# Supplementary material for: NHC-catalyzed enantioselective access to β-cyano carboxylic esters via in situ substrate alternation and release
Source: Nat Commun. 2023 Aug 12;14:4878. doi: 10.1038/s41467-023-40645-8 (PMC10423276; doi:10.1038/s41467-023-40645-8)
Supplement: Supplementary file 1 — Supplementary Information [file 41467_2023_40645_MOESM1_ESM.pdf]

Supplementary Information

NHC-Catalyzed Enantioselective Access to β-Cyano Carboxylic Esters via In Situ Substrate Alternation and Release

Qingyun Wang[1†], Shuquan Wu[2†], Juan Zou[3†], Xuyang Liang[1], Chengli Mou[3], Pengcheng Zheng[1]*, and Yonggui Robin Chi[1, 4]*

[1]National Key Laboratory of Green Pesticide, Key Laboratory of Green Pesticide and Agricultural Bioengineering, Ministry of Education, Center for R&D of Fine Chemicals, Guizhou University, Guiyang 550025, China.
[2]Guizhou Minzu University, Guiyang 550025, China
[3]Guizhou University of Traditional Chinese Medicine, Guiyang 550025, China
[4]School of Chemistry, Chemical Engineering and Biotechnology, Nanyang Technological University, Singapore 637371, Singapore.

Corresponding authors e-mails:
zhengpc1986@163.com
robinchi@ntu.edu.sg

[†]These authors contributed equally to this work.

Table of contents

I. Supplementary Notes S3

General information……………………………………………………………….….S3

II. Supplementary Methods S4

Preparation of substrates…………………………………………………………………S4

Initial studies and condition optimization for the synthesis of 3a………………………...S6

General procedure for the catalytic reactions………………………………………………S8

III. Supplementary Discussion..S10

Mechanistic study experiments: GC-MS semi-quantitative analysis of reactions...........S10

LC-HRMS analysis of reaction systems………………………………...…………….S11

IV. Characterization of substrates and products S15

V. Supplementary Figures S35

^{1}H NMR, ^{13}C NMR and ^{19}F NMR spectra…………………………………….…….S35

HPLC spectra…………………………………………………………...……………S106

HRMS spectra…………………………………………………………………...……..S142

X-Ray crystallography………………………………………………………………….S180

VI. Supplementary References S184

I. Supplementary Notes

General information

Commercially available materials and dry solvents purchased from Energy Chemical and Bidepharm were used as received. Unless otherwise specified, all reactions were prepared using 4 mL vial under N_2 atmosphere in glove-box from M Braun (UNILAB SP). Proton nuclear magnetic resonance (^{1}H NMR) spectra were recorded on a Bruker (AVANCE III HD 400 MHz) spectrometer. Chemical shifts were recorded in parts per million (ppm, δ) relative to tetramethylsilane (δ 0.00) or chloroform (δ = 7.26, singlet). ^{1}H NMR splitting patterns are designated as singlet (s), doublet (d), triplet (t), quartet (q), dd (doublet of doublets); m (multiplets), etc. All first-order splitting patterns were assigned on the basis of the appearance of the multiplet. Splitting patterns that could not be easily interpreted are designated as multiplet (m) or broad (br). Carbon nuclear magnetic resonance (^{13}C NMR) spectra were recorded on a Bruker (AVANCE III HD 101 MHz) spectrometer. Fluorine (^{19}F) nuclear magnetic resonance (^{19}F NMR) spectra were recorded on a Bruker (AVANCE III HD 376 MHz) spectrometer. The melting points (m.p.) of the title compounds were determined when left untouched on an XT-4-MP apparatus from Beijing Tech. Instrument Co. (Beijing, China). High resolution mass spectrometer analysis (HRMS) was performed on Waters Xevo G2-S QTOF mass spectrometer. The gas chromatography mass spectrometry (GC-MS) analyses were measured on Agilent systems, 7890B-5977B GC/MSD model. The liquid chromatography-high resolution mass spectrum (LC-HRMS) was performed on Waters Xevo G2-S QTOF system. Absolute configuration of the products was determined by X-ray crystallography (Bruker D8 quest). HPLC analyses were measured on Waters systems, Daicel Chemical Industries systems and Shimadzu systems with Empower3 system controller, Alliance 2695, and 2998 Diode Array Waters 2489 UV/Vis detector. Chiralcel brand chiral columns from Daicel Chemical Industries LC 20A QA&QC-HPLC were used with models IA, IG, IF, OD-H, AD-H, AS3RCD-BR007 or IF00CE-TE005 in 4.6 x 250 mm size. Optical rotations were measured on a Insmark IP-digi Polarimeter in a 1 dm cuvette at 25 °C. The concentration (c) is given in g/100 mL. Analytical thin-layer chromatography (TLC) was carried out pre-coated silica gel plate (0.2 mm thickness). Visualization was performed using a UV lamp.

II. Supplementary Methods

Preparation of substrates

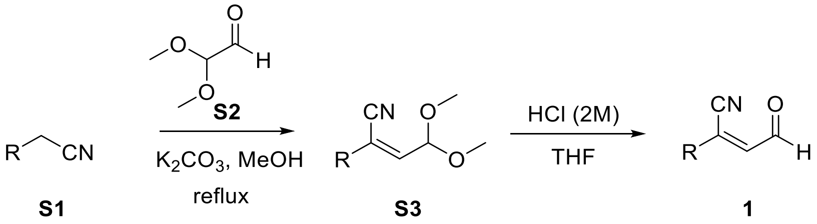

S1 (20.00 mmol, 1.0 eq) and **S2** (2.51 g, 3.62 mL, 24.00 mmol, 1.20 eq, purity: 60 wt. % in H_2O) were dissolved in MeOH (50.0 mL) at room temperature. After that, K_2CO_3 (4.15 g, 30.00 mmol, 1.50 eq) was added. The mixture was heated to reflux for 2 hrs. After completion of the reaction as monitored by TLC. The reaction system was cooled to room temperature and poured water (70.0 mL) into the mixture. The mixture was extracted with ethyl acetate (3×50.0 mL), the combined organic layers were washed with brine and dried over anhydrous sodium sulphate. The residual solvents were removed under reduced pressure to afford the product **S3** as colorless liquid without further purification.

To a stirred **S3** in THF (50.0 mL) was added 2M HCl (50.0 mL). The mixture was stirred at room temperature for 1h until material completion as monitored by TLC. The mixture was extracted with ethyl acetate (3×50.0 mL). The organic layers were washed with brine and treated with anhydrous sodium sulphate. The solvent was evaporated and the product **1** was purified by silica gel column chromatography using petroleum ether / ethyl acetate as eluent (10 / 1).[1]

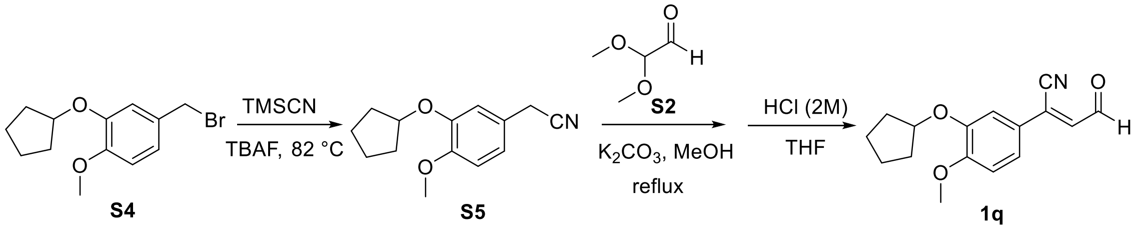

S4 (1.00 g, 3.51mmol) was dissolved in MeCN (50.0 mL), and TMSCN (Trimethylsilyl cyanide) (5.26 mmol) was added at room temperature. After that, TBAF (Tetrabutylammonium fluoride) (5.26 mmol) was added at same temperature then the reaction system was heated up to 82°C stirred for 5 hrs. After completion of the reaction as monitored by TLC. The mixture was rotary evaporated to remove solvent and unreacted TMSCN. Then the mixture was quenched with water and washed ethyl acetate (3×10.0 mL). The organic extracts were combined, dried over anhydrous sodium sulphate and concentrated. The crude product was purified by silica gel column chromatography using petroleum ether / ethyl acetate (10 / 1) as eluent to afford compound **S5**.[2]

Compound **1q** can be synthesized with the same method that compound **S1** to **1** from the compound **S5**.

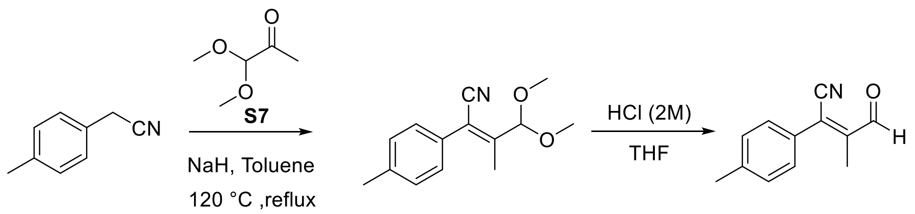

S6 **S8** **1x**

S6 (1.00 g, 7.62mmol) was dissolved in Toluene (30.0 mL), and NaH (15.25 mmol) was added slowly at 0°C. After 30min, **S7** (5.26 mmol) was added at same temperature then the reaction system was heated up to 120°C stirred for 6 hrs. After completion of the reaction as monitored by TLC. The mixture was quenched with water and washed with ethyl acetate (3×10.0 mL). The organic extracts were combined, dried over anhydrous sodium sulphate and concentrated. The crude product was purified by silica gel column chromatography using petroleum ether / ethyl acetate (20 / 1) as eluent to afford compound **S8**.[3]

Compound **1x** can be synthesized with the same method that compound **S3** to **1** from the compound **S8**.

Initial studies and condition optimization for the synthesis of 3a

Supplementary Table 1. The initial studies of nucleophile[a]

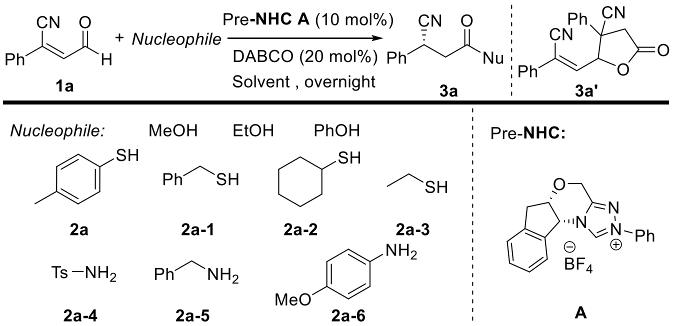

Entry	Nucleophile	NHC	Base	Solvent	Desired product **3a** [%][b]	Homo-coupling **3a'** [%][b]
1	**MeOH**	**A**	DABCO	THF	0	82
2	**MeOH**	**A**	DABCO	DCM	0	64
3	**MeOH**	**A**	DABCO	EA	0	72
4	**EtOH**	**A**	DABCO	THF	0	77
5	**EtOH**	**A**	DABCO	DCM	0	69
6	**EtOH**	**A**	DABCO	EA	0	70
7	**PhOH**	**A**	DABCO	THF	0	81
8	**PhOH**	**A**	DABCO	DCM	0	79
9	**PhOH**	**A**	DABCO	EA	0	66
10	**2a**	**A**	DABCO	THF	80	0
11	**2a**	**A**	DABCO	DCM	73	0
12	**2a**	**A**	DABCO	EA	78	0
13	**2a-1**	**A**	DABCO	THF	0	75
14	**2a-1**	**A**	DABCO	DCM	0	81
15	**2a-1**	**A**	DABCO	Toluene	0	80
16	**2a-2**	**A**	DABCO	THF	0	79
17	**2a-2**	**A**	DABCO	DCM	0	65
18	**2a-2**	**A**	DABCO	Toluene	0	83
19	**2a-3**	**A**	DABCO	THF	0	77
20	**2a-3**	**A**	DABCO	DCM	0	78
21	**2a-3**	**A**	DABCO	Toluene	0	81
22	**2a-4**	**A**	DABCO	THF	0	60
23	**2a-4**	**A**	DABCO	DCM	0	61
24	**2a-4**	**A**	DABCO	Toluene	0	67
25	**2a-5**	**A**	DABCO	THF	0	0
26	**2a-5**	**A**	DABCO	DCM	0	0
27	**2a-5**	**A**	DABCO	Toluene	0	0
28	**2a-6**	**A**	DABCO	THF	0	0
29	**2a-6**	**A**	DABCO	DCM	0	0
30	**2a-6**	**A**	DABCO	Toluene	0	0

[a]General conditions (unless otherwise specified): **1a** (0.10 mmol), nucleophiles (0.10 mmol), pre-**NHC A** (0.01 mmol), base (0.02 mmol), solvents (2.0 mL), rt, 12 hrs. [b]Isolated yields of **3a** and **3a'**. DABCO = 1,4-Diazabicyclo [2.2.2]octane.

Supplementary Table 2. The effects of catalysts, bases, solvents on the reaction outcome[a]

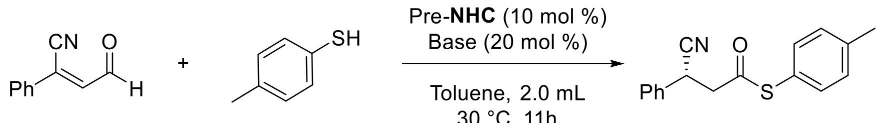

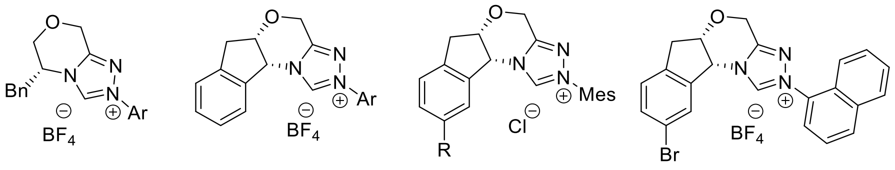

Entry	Pre-NHC	Base	Solvent	Yield (%)[b]	er[c]
1	**A**	K_2CO_3	THF	71	64:36
2	**B**	K_2CO_3	THF	85	55:45
3	**C**	K_2CO_3	THF	74	84:16
4	**D**	K_2CO_3	THF	64	62:38
5	**E**	K_2CO_3	THF	49	70:30
6	**F**	K_2CO_3	THF	57	58:42
7	**G**	K_2CO_3	THF	80	78:22
8	**H**	K_2CO_3	THF	69	80:20
9	**I**	K_2CO_3	THF	73	71:29
10	**J**	K_2CO_3	THF	74	81:19
11	**C**	Cs_2CO_3	THF	83	50:50
12	**C**	Na_2CO_3	THF	80	66:34
13	**C**	Et_3N	THF	62	71:29
14	**C**	DABCO	THF	88	85:15
15	**C**	DMAP	THF	76	88:12
16	**C**	DIPEA	THF	79	68:32
17	**C**	Quinecldine	THF	80	94:6
18	**C**	DABCO	DCM	73	89:11
19	**C**	DABCO	EtOAc	78	94:6
20	**C**	DABCO	MTBE	56	91:9
21	**C**	DABCO	Toluene	81	94:6
22	**C**	DABCO	Xylene	86	91:9
23[d]	**C**	DABCO	Toluene	83	95:5

[a]General conditions (unless otherwise specified): **1a** (0.10 mmol), **2a** (0.12 mmol), pre-**NHCs** (0.01 mmol), bases (0.02 mmol), solvents (2.0 mL), 30 °C, 11 hrs. [b]Isolated yield of **3a**. [c]The er values were determined via HPLC on chiral stationary phase. [d]Using 100 mg 4 Å MS as additive. DMAP = 4-Dimethylaminopyridine. DABCO = 1,4-Diazabicyclo [2.2.2]octane. DIEA = N,N-Diisopropylethylamine. Quinecldine= 1,4-ethanopiperidine.

General procedure for the catalytic reactions

General procedure for the catalytic reactions of enedial 1 and thiophenol 2 to synthesize product 3&4:

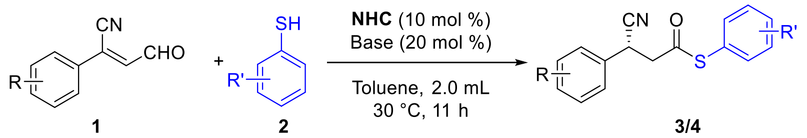

To a dry 4.0 mL vial equipped with a magnetic stir bar, β-cyano cinnamaldehyde **1** (0.10 mmol), aromatic thiol **2** (0.12 mmol), **NHC** (0.01 mmol) and base (0.02 mmol) were added. In glove-box, to the vial was added anhydrous toluene (2.0 mL), 4 Å MS (100 mg) follow by closure. The reaction mixture was then stirred at 30 ºC in oil bath till β-cyano cinnamaldehyde **1** was completely consumed (monitored by TLC). The mixture was concentrated under reduced pressure. The resulting crude residue was purified *via* column chromatography on silica gel (petroleum ether / ethyl acetate = 15 / 1) to afford the desired products **3/4**.

Synthetic transformations of chiral products:

Preparation of **(R)-Phenibut** and **(R)-Baclofen** from product **3a** and **3f**:

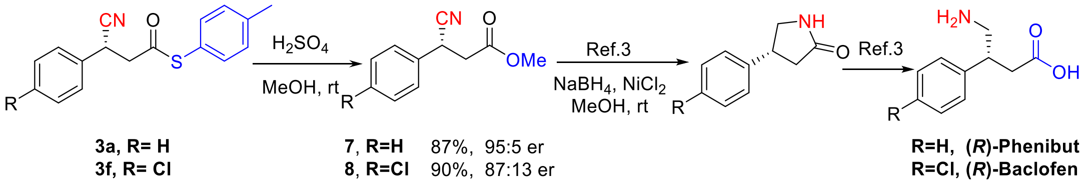

Compound **3a** (2.81 g, 10.0 mmol) or **3f** (3.16 g, 10.0 mmol) was dissolved in MeOH (15.0 mL) and 2 mL of H_2SO_4 (12 M) was added. The mixture was stirred at room temperature for over 12 hrs until the consumption of the **3a** or **3f** (monitored by TLC). The reaction was then cooled to 0 °C and quenched by the slow addition of water, extracted with ethyl acetate. The combined organic phases were dried over Na_2SO_4, concentrated under reduced pressure. The crude product was purified *via* SiO_2 flash chromatography (petroleum ether / ethyl acetate = 10 / 1) to afford **7** (1.64 g, 87% yield, 95:5 er) or **8** (2.00 g, 90% yield, 87:13 er) as yellow solid.[3-5] Compounds **7** and **8** can be converted to **(R)-Phenibut** and **(R)-Baclofen** according to literature procedure.[3-5]

Preparation of **(R)-Rolipram** from product **3q**:

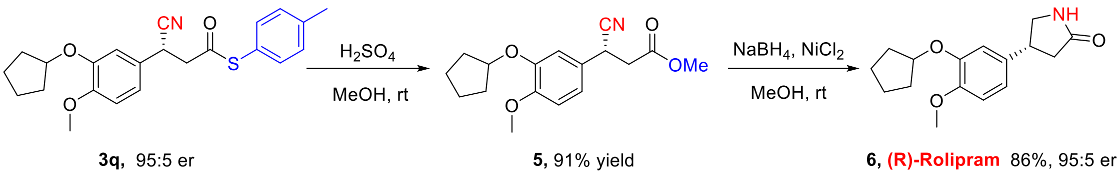

Compound **3q** (3.60 g, 13.5 mmol) was dissolved in MeOH (40.0 mL) and 3.0 mL of H_2SO_4 (12 M) was added. The mixture was stirred at room temperature for over 12 hrs until the consumption of the **3q** (monitored by TLC). The reaction was then cooled to 0 °C and quenched by the slow addition of water,

extracted with ethyl acetate. The combined organic phases were dried over Na_2SO_4, concentrated under reduced pressure. The crude product was purified *via* SiO_2 flash chromatography (petroleum ether / ethyl acetate = 10 / 1) to afford **5** (2.76 g, 91 % yield) as yellow solid.

Compound **5** (2.76 g, 8.25 mmol) was dissolved in MeOH (20.0 mL) and 5.0 equiv. of $NaBH_4$ (1.56 g, 41.21 mmol) was added. The mixture was stirred at room temperature then added 2.0 equiv. of $NiCl_2$ (2.14 g, 16.48 mmol), after that stirred at room temperature for 1h until the consumption of the **5** (monitored by TLC). The reaction was then cooled to 0 °C and quenched by the slow addition of a saturated NH_4Cl, extracted with dichloromethane. The combined organic phases were dried over Na_2SO_4, concentrated under reduced pressure. The crude product was purified *via* SiO_2 flash chromatography (dichloromethane / methanol = 20 / 1) to afford **(R)-Rolipram** (**6**) (2.16 g, 86% yield, 95:5 er) as white solid.[4-6]

III. Supplementary Discussion

Mechanistic study experiments: GC-MS semi-quantitative analysis of reactions

To a dry Schlenk tube equipped with a magnetic stir bar, **1a** (0.10 mmol), **2a** (0.12 mmol), triazolium salt **NHC-A** (0.01 mmol) and DABCO (0.02 mmol) were added. The vial was then sealed, purged and backfilled with N_2 three times in glovebox before adding anhydrous toluene (2.0 mL), using 100 mg 4 Å MS as additive. The reaction mixture was stirred in oil bath at 30 °C. At the same time, we prepared a methanol solution of mes-trimethoxybenzene as standard (10.00 mg mes-trimethoxybenzene in 25.0 mL MeOH). During the subsequent reaction process, 50.0 μL of the reaction system was taken out and diluted in 600.0 μL of methanol solution at regular intervals which was determined by Gas chromatography mass spectrometry (GC-MS). In particular, the initial concentration of **1a** was defined as the 100 % **1a** concentration (arb.units.), after the model reaction was complete, the concentration of product **3a** was defined as 100 % **3a** concentration (arb.units.). Meanwhile, the reaction in Table S1, entry 15 was also detected by GC-MS with same methods. Each experiment had been repeated three times under the same conditions. These numbers are semi-quantitative and can provide certain insights on the reaction mechanism.

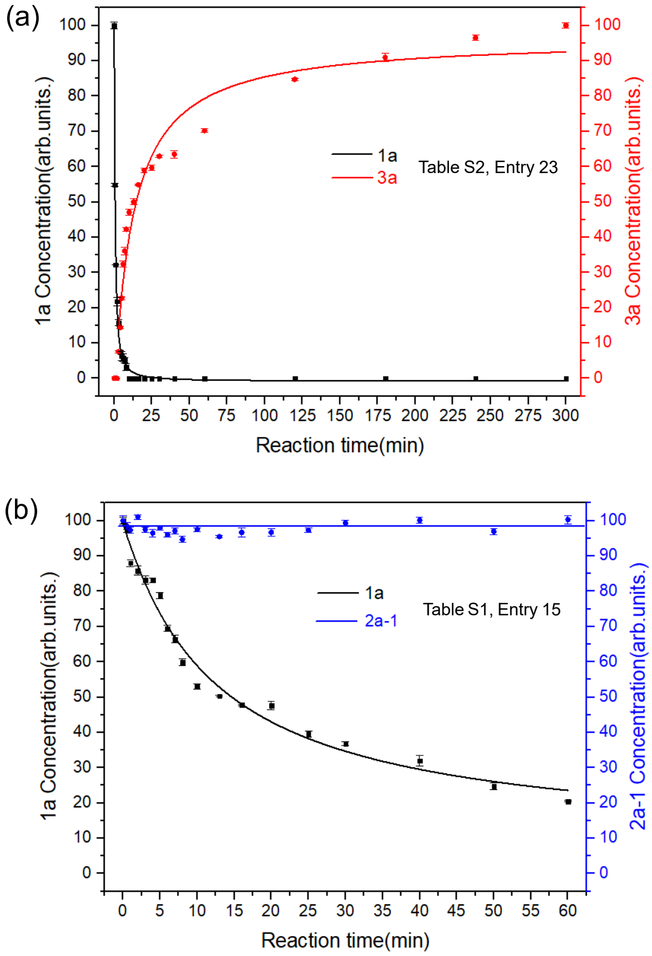

Supplementary Figure 1. GC-MS results of reaction systems. a. Reaction in Table S2, Entry 23. **b.** Reaction in Table S1, Entry 15

LC-HRMS analysis of reaction systems

To a dry Schlenk tube equipped with a magnetic stir bar, **1a** (0.10 mmol), triazolium salt **NHC-A** (0.01 mmol) and DBU (0.02 mmol) were added (Entry A). The vial was then sealed, purged and backfilled with N_2 three times in glovebox, using 100 mg 4 Å MS as additive and anhydrous toluene (2.0 mL) as solvent. The reaction mixture was then stirred at 30 °C in oil bath for 3 hours. After the indicated time, the supernatant of the reaction mixture was taken out and diluted. The crude mixture was subjected to liquid chromatography-high resolution mass spectrum (LC-HRMS) analysis. Meanwhile, the model reaction was also detected by LC-HRMS with same method (Entry B).

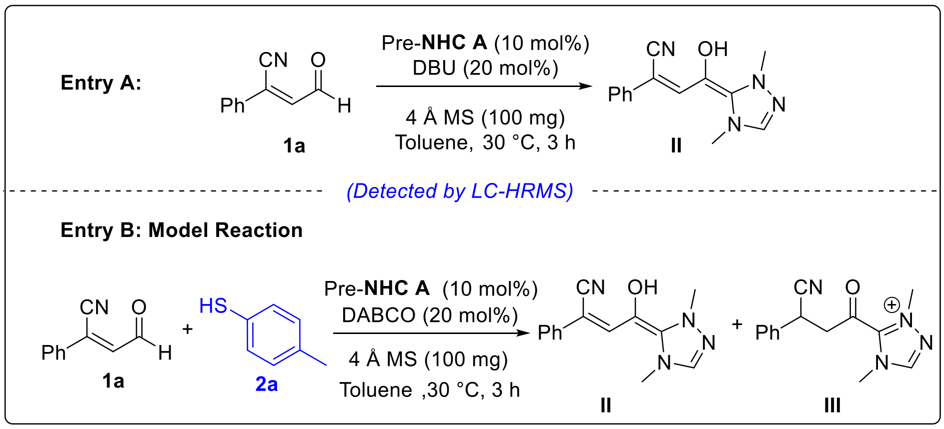

LC-HRMS results of Entry A:

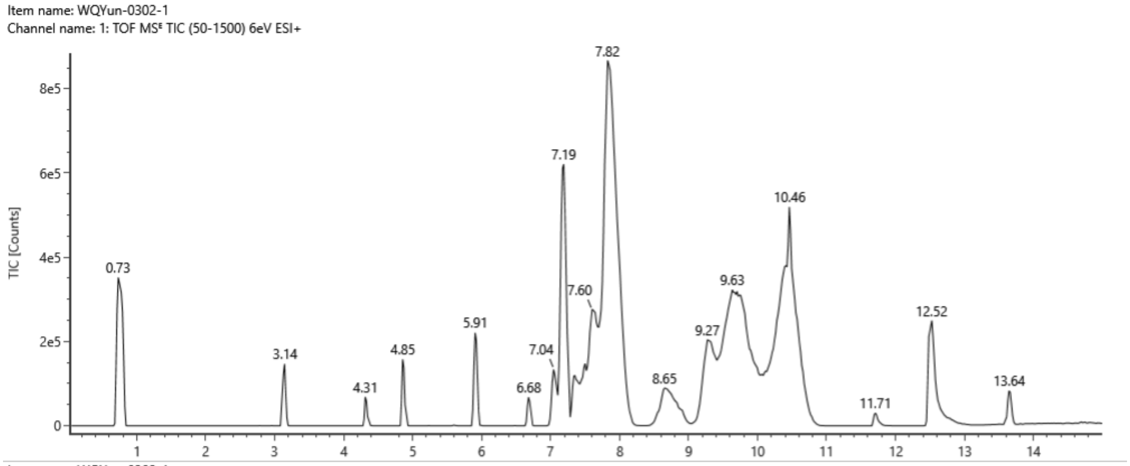

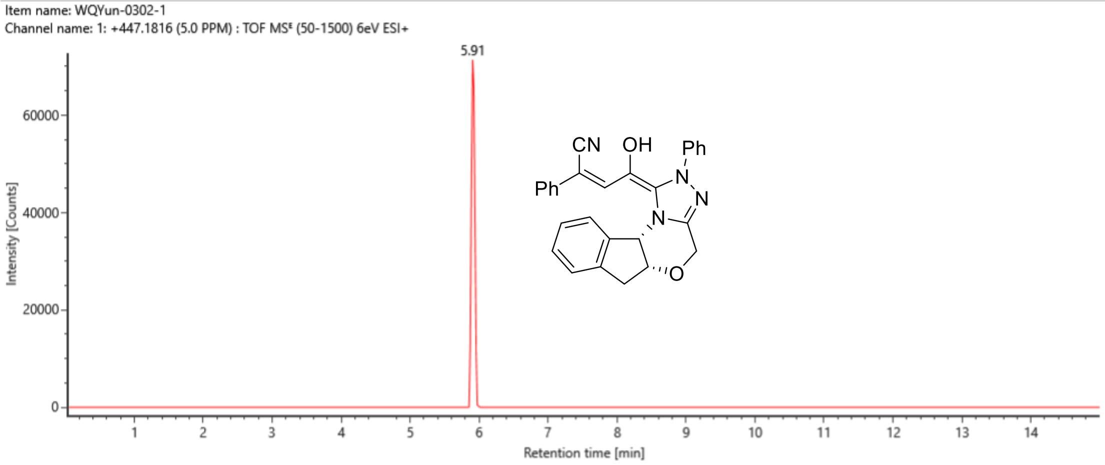

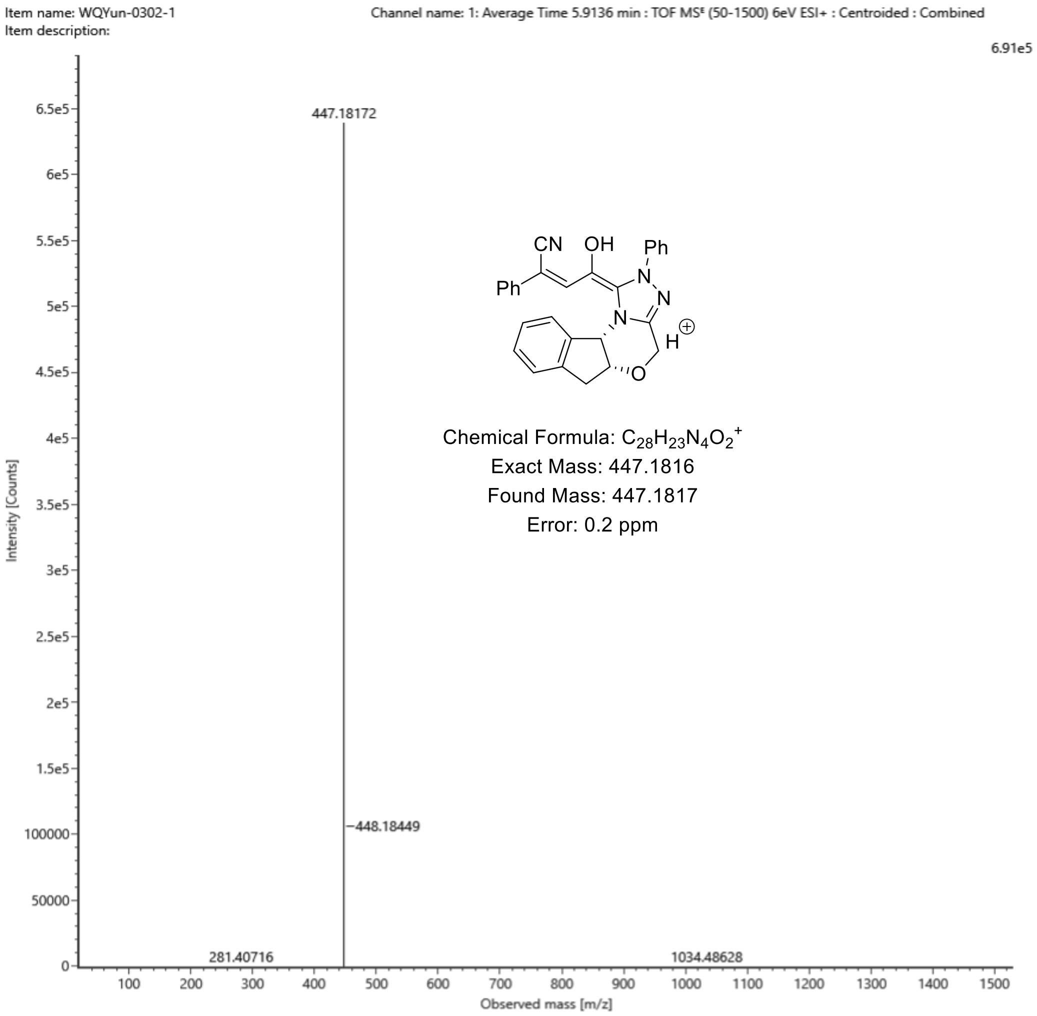

Supplementary Figure 2 LC-HRMS spectra and results of the reaction in Entry A

LC-HRMS results of Entry B:

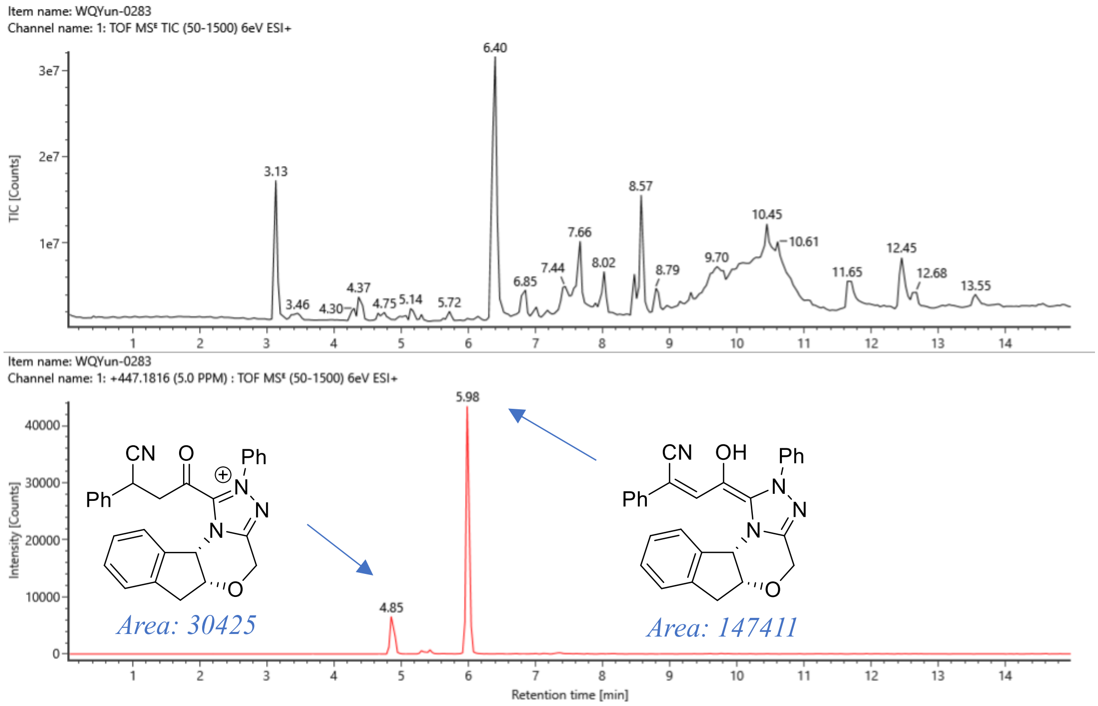

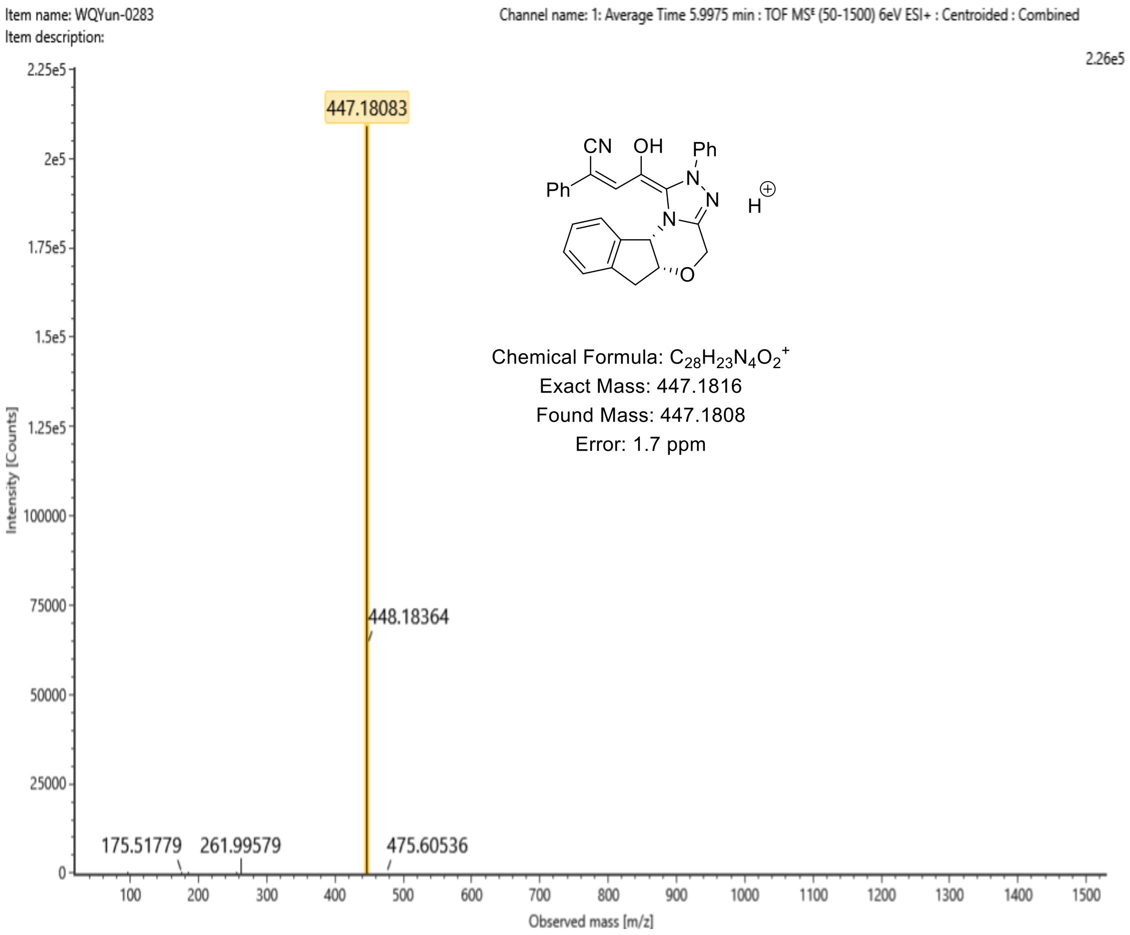
Item name: WQYun-0283
Item description:
Channel name: 1: Average Time 5.9975 min : TOF MS^E (50-1500) 6eV ESI+ : Centroided : Combined
2.26e5
447.18083
448.18364
175.51779
261.99579
475.60536
Intensity [Counts]
Observed mass [m/z]
CN
OH
Ph
Ph
N
N
N
O
H
Chemical Formula: C28H23N4O2+
Exact Mass: 447.1816
Found Mass: 447.1808
Error: 1.7 ppm

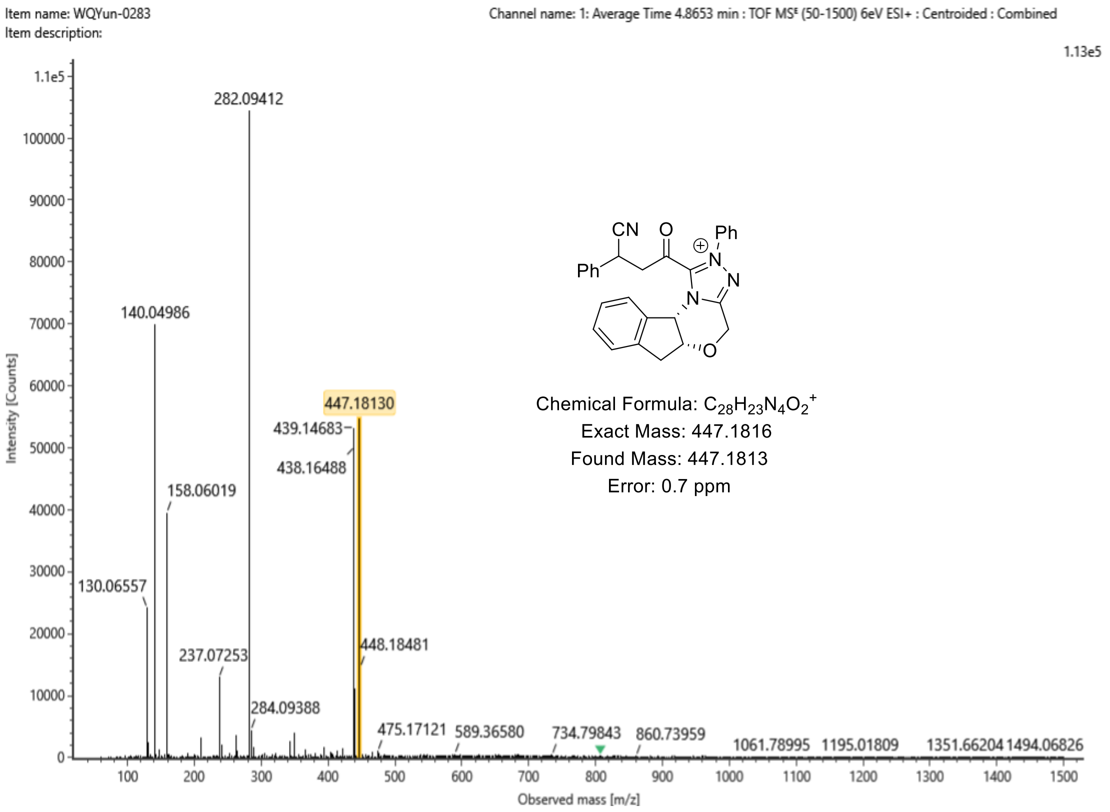
Item name: WQYun-0283
Item description:
Channel name: 1: Average Time 4.8653 min : TOF MS^E (50-1500) 6eV ESI+ : Centroided : Combined
1.13e5
282.09412
140.04986
447.18130
439.14683
438.16488
158.06019
130.06557
237.07253
448.18481
284.09388
475.17121
589.36580
734.79843
860.73959
1061.78995
1195.01809
1351.66204
1494.06826
Intensity [Counts]
Observed mass [m/z]
CN
O
Ph
Ph
N
N
N
O
Chemical Formula: C28H23N4O2+
Exact Mass: 447.1816
Found Mass: 447.1813
Error: 0.7 ppm

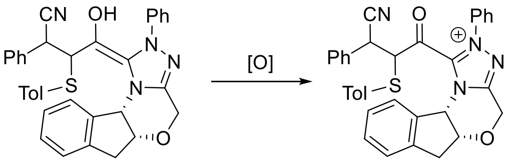

NHC-Thiol-click intermediate IV

cannot be detected

V

trace

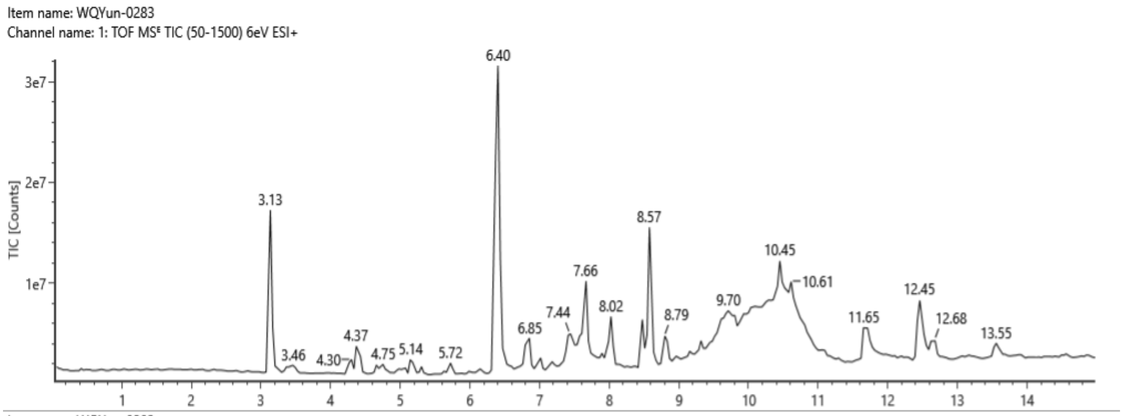

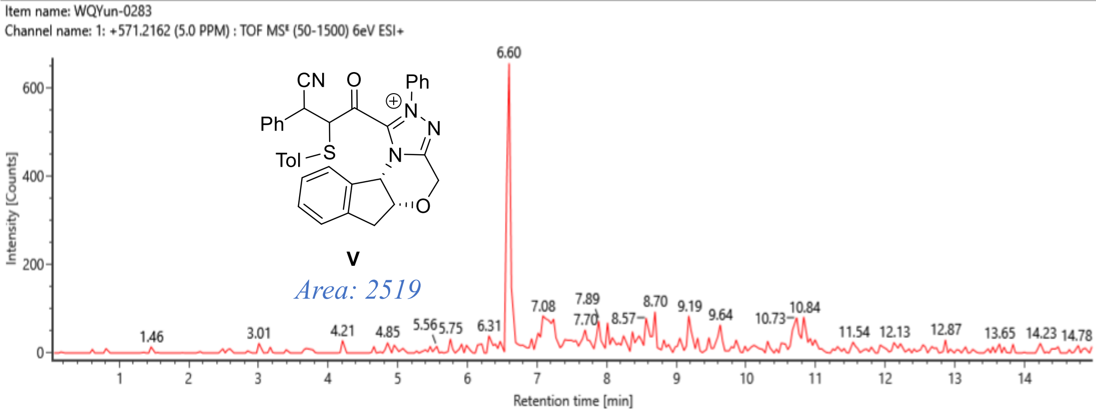

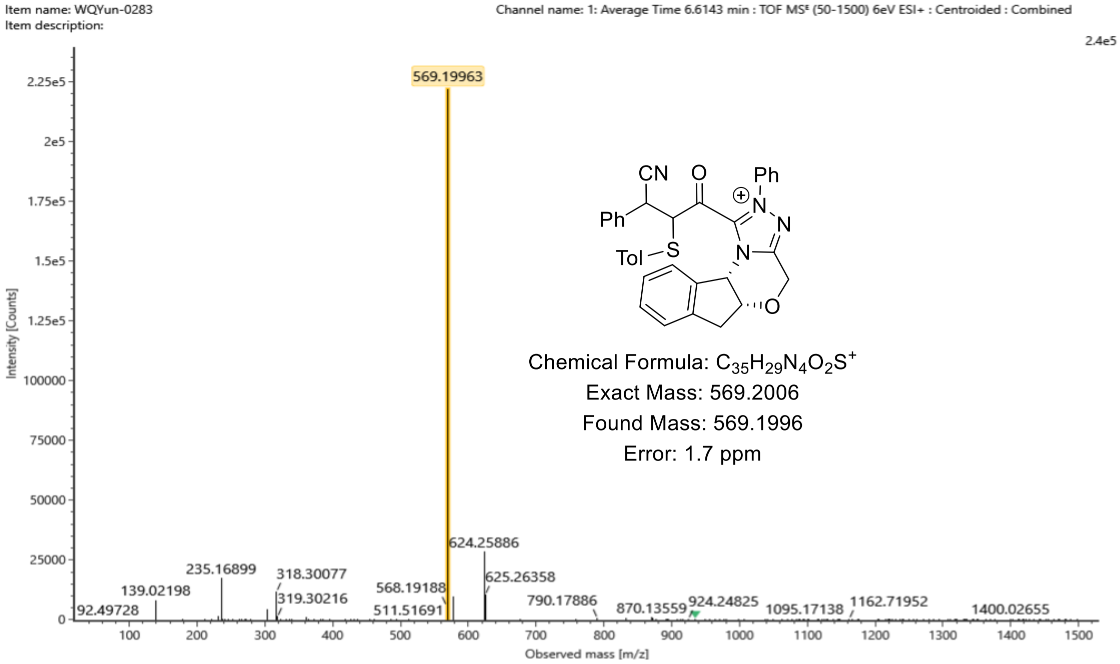

Supplementary Figure 3. LC-HRMS spectra and results of the reactions in Entry B

IV. Characterization of substrates and products

1. Characterization of substrates

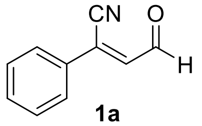
1a

(*Z*)-4-oxo-2-phenylbut-2-enenitrile (1a)

Purification by flash column chromatography on silica gel (petroleum ether / ethyl acetate = 15 / 1). Yellow solid, 71% yield, 3.55 g, m.p. 53-54 ºC.

^{1}H NMR (400 MHz, CDCl$_3$) δ 10.25 (d, *J* = 7.6 Hz, 1H), 7.79 – 7.75 (m, 2H), 7.59 – 7.51 (m, 3H), 7.06 (d, *J* = 7.6 Hz, 1H).

^{13}C NMR (101 MHz, CDCl$_3$) δ 188.8, 134.7, 131.5, 130.3, 129.4, 128.3, 125.8, 112.8.

HRMS (ESI, m/z): Mass calcd. for $C_{10}H_7NOH^+$ [M+H]$^+$, 158.0600, found 158.0608.

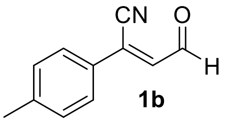
1b

(*Z*)-4-oxo-2-(*p*-tolyl)but-2-enenitrile (1b)

Purification by flash column chromatography on silica gel (petroleum ether / ethyl acetate = 15 / 1). Yellow solid, 85% yield, 4.25 g. m.p. 76-77 ºC.

^{1}H NMR (400 MHz, CDCl$_3$) δ 10.23 (d, *J* = 7.7 Hz, 1H), 7.66 (d, *J* = 8.3 Hz, 2H), 7.32 (d, *J* = 8.1 Hz, 2H), 7.01 (d, *J* = 7.6 Hz, 1H), 2.44 (s, 3H).

^{13}C NMR (101 MHz, CDCl$_3$) δ 190.3, 143.9, 134.9, 131.6, 130.4, 128.0, 127.1, 114.2, 21.6.

HRMS (ESI, m/z): Mass calcd. for $C_{11}H_{10}NO^+$ [M+H]$^+$, 172.0757; found 172.0760.

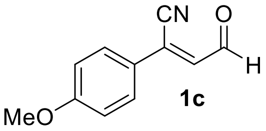
1c

(*Z*)-2-(4-methoxyphenyl)-4-oxobut-2-enenitrile (1c)

Purification by flash column chromatography on silica gel (petroleum ether / ethyl acetate = 10 / 1). Yellow solid, 90% yield, 4.5 g. m.p. 115-117 ºC.

^{1}H NMR (400 MHz, CDCl$_3$) δ 10.19 (d, *J* = 7.7 Hz, 1H), 7.72 (d, *J* = 8.9 Hz, 2H), 7.01 (d, *J* = 8.9 Hz, 2H), 6.94 (d, *J* = 7.8 Hz, 1H), 3.89 (s, 3H).

^{13}C NMR (101 MHz, CDCl$_3$) δ 190.2, 163.4, 133.4, 131.2, 129.0, 123.1, 115.1, 114.3, 55.7.

HRMS (ESI, m/z): Mass calcd. for $C_{11}H_{10}NO_2^+$ [M+H]$^+$, 188.0706; found 188.0712.

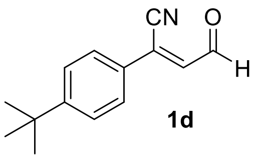
1d

(*Z*)-2-(4-(tert-butyl)phenyl)-4-oxobut-2-enenitrile (1d)

Purification by flash column chromatography on silica gel (petroleum ether / ethyl acetate = 5 / 1). Yellow solid, 91% yield, 4.6 g. m.p. 49-51 ºC.

^{1}H NMR (400 MHz, CDCl$_3$) δ 10.23 (d, *J* = 7.7 Hz, 1H), 7.71 (d, *J* = 8.6 Hz, 2H), 7.54 (d, *J* = 8.6 Hz, 2H), 7.04 (d, *J* = 7.7 Hz, 1H), 1.36 (s, 9H).

^{13}C NMR (101 MHz, CDCl$_3$) δ 190.2, 156.9, 135.0, 131.5, 127.9, 127.0, 126.7, 114.2, 35.2, 31.0.

HRMS (ESI, m/z): Mass calcd. for $C_{14}H_{16}NO^+$ [M+H]$^+$, 214.1226; found 214.1228.

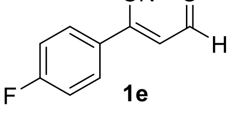
1e

(*Z*)-2-(4-fluorophenyl)-4-oxobut-2-enenitrile (1e)

Purification by flash column chromatography on silica gel (petroleum ether / ethyl acetate = 10 / 1). Yellow solid, 80% yield, 4.0 g, m.p. 74-75 ºC.

^{1}H NMR (400 MHz, $CDCl_3$) δ 10.23 (d, *J* = 7.6 Hz, 1H), 7.81 – 7.76 (m, 2H), 7.27 – 7.21 (m, 2H), 7.00 (d, *J* = 7.7 Hz, 1H).

^{13}C NMR (101 MHz, $CDCl_3$) δ 189.9, 165.2 (d, *J* = 256.3 Hz), 135.7 (d, *J* = 2.5 Hz), 130.4, 129.4 (d, *J* = 9.1 Hz), 127.0 (d, *J* = 3.6 Hz), 117.1 (d, *J* = 22.5 Hz), 113.9.

^{19}F NMR (376 MHz, $CDCl_3$) δ -104.87.

HRMS (ESI, m/z): Mass calcd. for $C_{10}H_7FNO^+$ $[M+H]^+$, 176.0506; found 176.0508.

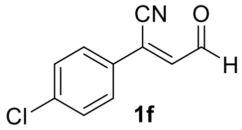

1f

(*Z*)-2-(4-chlorophenyl)-4-oxobut-2-enenitrile (1f)

Purification by flash column chromatography on silica gel (petroleum ether / ethyl acetate = 15 / 1). Yellow solid, 80% yield, 4.0 g, m.p. 99-100°C.

^{1}H NMR (400 MHz, $CDCl_3$) δ 10.24 (d, *J* = 7.6 Hz, 1H), 7.71 (d, *J* = 8.8 Hz, 2H), 7.51 (d, *J* = 8.8 Hz, 2H), 7.04 (d, *J* = 7.6 Hz, 1H).

^{13}C NMR (101 MHz, $CDCl_3$) δ 189.9, 139.3, 136.1, 130.3, 130.0, 129.1, 128.3, 113.8.

HRMS (ESI, m/z): Mass calcd. for $C_{10}H_7ClNO^+$ $[M+H]^+$, 192.0211; found 192.0212.

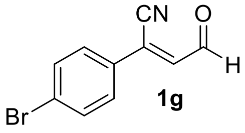

1g

(*Z*)-2-(4-bromophenyl)-4-oxobut-2-enenitrile (1g)

Purification by flash column chromatography on silica gel (petroleum ether / ethyl acetate = 10 / 1). Yellow solid, 79% yield, 3.9 g, m.p. 101-103 °C.

^{1}H NMR (400 MHz, $CDCl_3$) δ 10.24 (d, *J* = 7.6 Hz, 1H), 7.67 (d, *J* = 8.9 Hz, 2H), 7.63 (d, *J* = 8.9 Hz, 2H), 7.04 (d, *J* = 7.6 Hz, 1H).

^{13}C NMR (101 MHz, $CDCl_3$) δ 188.7, 135.0, 131.9, 129.3, 128.5, 127.3, 126.6, 112.6.

HRMS (ESI, m/z): Mass calcd. for $C_{10}H_7BrNO^+$ $[M+H]^+$, 235.9706; found 235.9705.

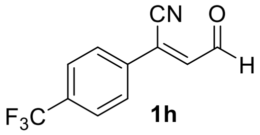

1h

(*Z*)-4-oxo-2-(4-(trifluoromethyl)phenyl)but-2-enenitrile (1h)

Purification by flash column chromatography on silica gel (petroleum ether / ethyl acetate = 10 / 1). Yellow solid, 83% yield, 4.2 g, m.p. 59-61 °C.

^{1}H NMR (400 MHz, $CDCl_3$) δ 10.25 (d, *J* = 7.5 Hz, 1H), 7.85 – 7.79 (m, 2H), 7.41 – 7.33 (m, 2H), 7.03 (d, *J* = 7.6 Hz, 1H).

^{13}C NMR (101 MHz, $CDCl_3$) δ 189.7, 152.2 (q, *J* = 2.04 Hz), 136.5, 130.0, 129.0, 128.9, 122.8 (q, *J* = 274.7 Hz), 121.5, 113.8.

^{19}F NMR (376 MHz, $CDCl_3$) δ -57.71.

HRMS (ESI, m/z): Mass calcd. for $C_{11}H_7F_3NO^+$ $[M+H]^+$, 226.0474; found 226.0474.

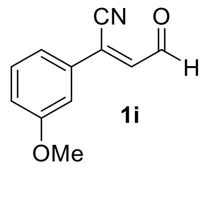

1i

(*Z*)-2-(3-methoxyphenyl)-4-oxobut-2-enenitrile (1i)

Purification by flash column chromatography on silica gel (petroleum ether / ethyl acetate = 5 / 1). Yellow solid, 87% yield, 4.4 g, m.p. 73-74 °C.

^{1}H NMR (400 MHz, $CDCl_3$) δ 10.25 (d, *J* = 7.7 Hz, 1H), 7.46 – 7.41 (m, 1H), 7.38 – 7.35 (m, 1H), 7.24 (s, 1H), 7.13 – 7.09 (m, 1H), 7.04 (d, *J* = 7.7 Hz, 1H), 3.88 (s, 3H).

^{13}C NMR (101 MHz, $CDCl_3$) δ 190.2, 160.3, 136.2, 131.9, 131.5, 130.7, 119.6, 118.6, 114.1, 112.2, 55.6.

HRMS (ESI, m/z): Mass calcd. for $C_{11}H_{10}NO_2^+$ [M+H]$^+$, 188.0706; found 188.0709.

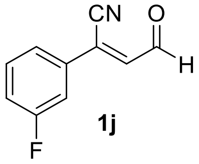

(*Z*)-2-(3-fluorophenyl)-4-oxobut-2-enenitrile (1j)

Purification by flash column chromatography on silica gel (petroleum ether / ethyl acetate = 15 / 1). Yellow solid, 80% yield, 4.0 g, m.p. 56-58 °C.

^{1}H NMR (400 MHz, $CDCl_3$) δ 10.25 (d, *J* = 7.5 Hz, 1H), 7.60 – 7.44 (m, 3H), 7.31 – 7.26 (m, 1H), 7.06 (d, *J* = 7.5 Hz, 1H).

^{13}C NMR (101 MHz, $CDCl_3$) δ 189.8, 163.0 (d, *J* = 249.6 Hz), 136.9, 132.8 (d, *J* = 7.9 Hz), 131.4 (d, *J* = 8.3 Hz), 130.2, 123.0 (d, *J* = 3.2 Hz), 119.8 (d, *J* = 21.4 Hz), 114.0 (d, *J* = 23.9 Hz), 113.7.

^{19}F NMR (376 MHz, $CDCl_3$) δ -110.05.

HRMS (ESI, m/z): Mass calcd. for $C_{10}H_7FNO^+$ [M+H]$^+$, 176.0506; found 176.0511.

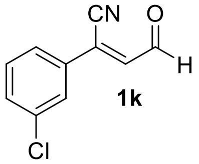

(*Z*)-2-(3-chlorophenyl)-4-oxobut-2-enenitrile (1k)

Purification by flash column chromatography on silica gel (petroleum ether / ethyl acetate = 15 / 1). Yellow solid, 53% yield, 2.7 g, m.p. 105-106 °C.

^{1}H NMR (400 MHz, $CDCl_3$) δ 10.24 (d, *J* = 7.5 Hz, 1H), 7.75 – 7.74 (m, 1H), 7.67 (d, *J* = 7.8 Hz, 1H), 7.57 – 7.54 (m, 1H), 7.49 (t, *J* = 7.9 Hz, 1H), 7.07 (d, *J* = 7.5 Hz, 1H).

^{13}C NMR (101 MHz, $CDCl_3$) δ 189.8, 137.0, 135.9, 132.7, 132.4, 130.9, 130.0, 127.0, 125.3, 113.7.

HRMS (ESI, m/z): Mass calcd. for $C_{10}H_7ClNO^+$ [M+H]$^+$, 192.0211; found 192.0217.

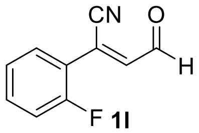

(*Z*)-2-(2-fluorophenyl)-4-oxobut-2-enenitrile (1l)

Purification by flash column chromatography on silica gel (petroleum ether / ethyl acetate = 15 / 1). Yellow solid, 77% yield, 3.9 g. m.p. 54-56 °C.

^{1}H NMR (400 MHz, $CDCl_3$) δ 10.27 (d, *J* = 7.6 Hz, 1H), 7.80 – 7.75 (m, 1H), 7.58 – 7.53 (m, 1H), 7.36 – 7.32 (m, 1H), 7.26 – 7.21 (m, 2H).

^{13}C NMR (101 MHz, $CDCl_3$) δ 190.5, 160.7 (d, *J* = 257.0 Hz), 140.6 (d, *J* = 13.4 Hz), 134.0 (d, *J* = 9.3 Hz), 130.5, 126.4 (d, *J* = 3.2 Hz), 125.3, 119.2 (d, *J* = 9.8 Hz), 117.2 (d, *J* = 22.3 Hz), 113.7.

^{19}F NMR (376 MHz, $CDCl_3$) δ -109.98.

HRMS (ESI, m/z): Mass calcd. for $C_{10}H_7FNO^+$ [M+H]$^+$, 176.0506; found 176.0509.

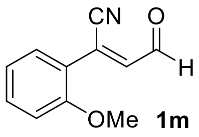

(*Z*)-2-(2-methoxyphenyl)-4-oxobut-2-enenitrile (1m)

Purification by flash column chromatography on silica gel (petroleum ether / ethyl acetate = 10 / 1). Yellow solid, 89% yield, 4.5 g. m.p. 79-81 °C.

^{1}H NMR (400 MHz, $CDCl_3$) δ 10.27 (d, *J* = 7.9 Hz, 1H), 7.69 (dd, *J* = 7.8, 1.7 Hz, 1H), 7.55 – 7.46 (m, 1H), 7.38 (d, *J* = 7.9 Hz, 1H), 7.14 – 7.05 (m, 1H), 7.02 (d, *J* = 8.4 Hz, 1H), 3.95 (s, 3H).

^{13}C NMR (101 MHz, CDCl$_3$) δ 191.7, 158.4, 139.8, 133.5, 130.8, 129.1, 121.3, 120.0, 114.5, 111.9, 55.8.

HRMS (ESI, m/z): Mass calcd. for $C_{11}H_{10}NO_2^+$ [M+H]$^+$, 188.0706; found 188.0707.

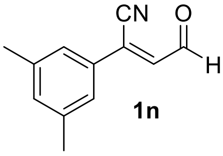

1n

(*Z*)-2-(3,5-dimethylphenyl)-4-oxobut-2-enenitrile (1n)

Purification by flash column chromatography on silica gel (petroleum ether / ethyl acetate = 5 / 1). Yellow solid, 92% yield, 4.6 g. m.p. 130-132 ºC.

^{1}H NMR (400 MHz, CDCl$_3$) δ 10.22 (d, *J* = 7.7 Hz, 1H), 7.36 (s, 2H), 7.19 (s, 1H), 7.02 (d, *J* = 7.7 Hz, 1H), 2.39 (s, 6H).

^{13}C NMR (101 MHz, CDCl$_3$) δ 190.2, 139.4, 135.6, 134.6, 131.9, 130.6, 124.9, 114.3, 21.2.

HRMS (ESI, m/z): Mass calcd. for $C_{12}H_{12}NO^+$ [M+H]$^+$, 186.0913; found 186.0915.

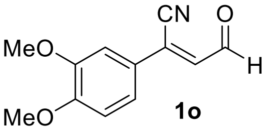

1o

(*Z*)-2-(3,4-dimethoxyphenyl)-4-oxobut-2-enenitrile (1o)

Purification by flash column chromatography on silica gel (petroleum ether / ethyl acetate = 5 / 1). Yellow solid, 97% yield, 4.9 g. m.p. 140-141 ºC.

^{1}H NMR (400 MHz, CDCl$_3$) δ 10.21 (d, *J* = 7.7 Hz, 1H), 7.45 (dd, *J* = 8.5, 2.3 Hz, 1H), 7.17 (d, *J* = 2.3 Hz, 1H), 6.96 (t, *J* = 8.4 Hz, 2H), 3.97 (s, 3H), 3.95 (s, 3H).

^{13}C NMR (101 MHz, CDCl$_3$) δ 190.2, 153.3, 149.7, 133.5, 131.3, 123.4, 121.9, 114.3, 111.4, 108.7, 56.2, 56.1.

HRMS (ESI, m/z): Mass calcd. for $C_{12}H_{14}NO_3^+$ [M+H]$^+$, 218.0812; found 218.0813.

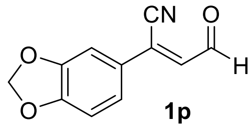

1p

(*Z*)-2-(benzo[d][1,3] dioxol-5-yl)-4-oxobut-2-enenitrile (1p)

Purification by flash column chromatography on silica gel (petroleum ether / ethyl acetate = 10 / 1). Yellow solid, 90% yield, 4.5 g. m.p. 173-175 ºC.

^{1}H NMR (400 MHz, CDCl$_3$) δ 10.20 (d, *J* = 7.6 Hz, 1H), 7.40 (dd, *J* = 8.2, 2.0 Hz, 1H), 7.16 (d, *J* = 1.9 Hz, 1H), 6.93 (d, *J* = 8.2 Hz, 1H), 6.89 (d, *J* = 7.7 Hz, 1H), 6.10 (s, 2H).

^{13}C NMR (101 MHz, CDCl$_3$) δ 190.2, 151.9, 149.1, 133.9, 131.1, 125.0, 123.8, 114.2, 109.1, 105.9, 102.4.

HRMS (ESI, m/z): Mass calcd. for $C_{11}H_8NO_3^+$ [M+H]$^+$, 202.0499; found 202.0499.

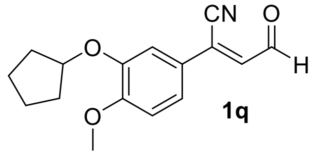

1q

(*Z*)-2-(3-(cyclopentyloxy)-4-methoxyphenyl)-4-oxobut-2-enenitrile (1q)

Purification by flash column chromatography on silica gel (petroleum ether / ethyl acetate = 10 / 1). Yellow solid, 87% yield, 4.4 g. m.p. 135-136 ºC.

^{1}H NMR (400 MHz, CDCl$_3$) δ 10.20 (d, *J* = 7.7 Hz, 1H), 7.41 (dd, *J* = 8.5, 2.3 Hz, 1H), 7.19 (d, *J* = 2.4 Hz, 1H), 6.94 (dd, *J* = 12.3, 8.2 Hz, 2H), 4.84 – 4.81 (m, 1H), 3.93 (s, 3H), 2.00 – 1.83 (m, 6H), 1.68 – 1.62 (m, 2H).

^{13}C NMR (101 MHz, CDCl$_3$) δ 190.3, 154.3, 148.4, 133.3, 131.5, 123.3, 121.6, 114.3, 112.0, 111.7, 80.9, 56.2, 32.8, 24.1.

HRMS (ESI, m/z): Mass calcd. for $C_{16}H_{17}NNaO_3^+$ [M+Na]$^+$, 294.1101; found 294.1101.

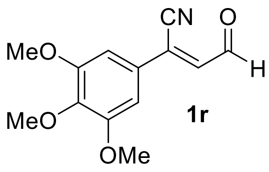

1r

(*Z*)-4-oxo-2-(3,4,5-trimethoxyphenyl)but-2-enenitrile (1r)

Purification by flash column chromatography on silica gel (petroleum ether / ethyl acetate = 8 / 1). Yellow solid, 90% yield, 4.5 g. m.p. 131-132 ºC.

^{1}H NMR (400 MHz, CDCl$_3$) δ 10.23 (d, *J* = 7.6 Hz, 1H), 7.00 (d, *J* = 7.7 Hz, 1H), 6.98 (s, 2H), 3.95 (s, 9H).

^{13}C NMR (101 MHz, CDCl$_3$) δ 190.1, 153.8, 142.2, 135.0, 131.3, 125.8, 114.2, 104.4, 61.2, 56.4.

HRMS (ESI, m/z): Mass calcd. for $C_{13}H_{14}NO_4^+$ [M+H]$^+$, 248.0917; found 248.0915.

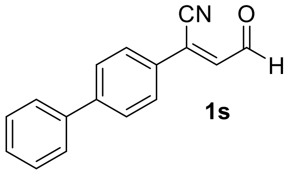

1s

(*Z*)-2-([1,1'-biphenyl]-4-yl)-4-oxobut-2-enenitrile (1s)

Purification by flash column chromatography on silica gel (petroleum ether / ethyl acetate = 10 / 1). Yellow solid, 83% yield, 4.2 g. m.p. 151-153 ºC.

^{1}H NMR (400 MHz, CDCl$_3$) δ 10.26 (d, *J* = 7.7 Hz, 1H), 7.84 (d, *J* = 8.6 Hz, 2H), 7.74 (d, *J* = 8.6 Hz, 2H), 7.67 – 7.60 (m, 2H), 7.52 – 7.40 (m, 3H), 7.08 (d, *J* = 7.6 Hz, 1H).

^{13}C NMR (101 MHz, CDCl$_3$) δ 190.1, 145.6, 139.1, 135.4, 131.2, 129.5, 129.1, 128.7, 128.1, 127.7, 127.2, 114.1.

HRMS (ESI, m/z): Mass calcd. for $C_{16}H_{12}NO^+$ [M+H]$^+$, 234.0913; found 234.0910.

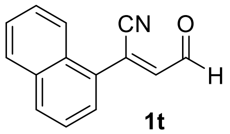

1t

(*Z*)-2-(naphthalen-1-yl)-4-oxobut-2-enenitrile (1t)

Purification by flash column chromatography on silica gel (petroleum ether / ethyl acetate = 10 / 1). Yellow solid, 80% yield, 4.0 g. m.p. 74-76 ºC.

^{1}H NMR (400 MHz, CDCl$_3$) δ 10.32 (d, *J* = 7.7 Hz, 1H), 8.08 (d, *J* = 8.4 Hz, 1H), 7.94 (d, *J* = 8.2 Hz, 1H), 7.90 – 7.87 (m, 1H), 7.59 – 7.53 (m, 3H), 7.50 – 7.46 (m, 1H), 6.88 (d, *J* = 7.7 Hz, 1H).

^{13}C NMR (101 MHz, CDCl$_3$) δ 188.3, 140.8, 132.2, 130.7, 129.4, 128.3, 128.0, 127.6, 126.4, 126.2, 125.5, 123.6, 122.1, 113.4.

HRMS (ESI, m/z): Mass calcd. for $C_{14}H_{10}NO^+$ [M+H]$^+$, 208.0757; found 208.0760.

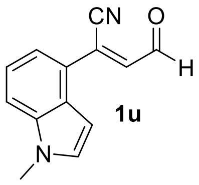

1u

(*Z*)-2-(1-methyl-1H-indol-4-yl)-4-oxobut-2-enenitrile (1u)

Purification by flash column chromatography on silica gel (petroleum ether / ethyl acetate = 6 / 1). Yellow solid, 74% yield, 3.7 g. m.p. 138-140 ºC.

^{1}H NMR (400 MHz, CDCl$_3$) δ 10.16 (d, *J* = 7.9 Hz, 1H), 7.86 (d, *J* = 7.9 Hz, 1H), 7.80 (s, 1H), 7.43 – 7.34 (m, 3H), 7.01 (d, *J* = 8.0 Hz, 1H), 3.90 (s, 3H).

^{13}C NMR (101 MHz, CDCl$_3$) δ 190.5, 138.8, 135.2, 129.0, 126.0, 124.4, 124.4, 123.1, 120.7, 114.9, 110.9, 110.5, 33.8.

HRMS (ESI, m/z): Mass calcd. for $C_{13}H_{10}N_2NaO^+$ [M+Na]$^+$, 233.0685; found 233.0683.

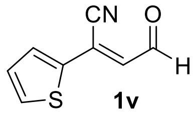

1v

(*E*)-4-oxo-2-(thiophen-2-yl)but-2-enenitrile (1v)

Purification by flash column chromatography on silica gel (petroleum ether / ethyl acetate = 5 / 1). Yellow solid, 70% yield, 3.5 g. m.p. 82-83 °C.

^{1}H NMR (400 MHz, CDCl$_3$) δ 10.14 (d, *J* = 7.6 Hz, 1H), 7.71 (dd, *J* = 3.8, 1.2 Hz, 1H), 7.62 (dd, *J* = 5.1, 1.2 Hz, 1H), 7.20 (dd, *J* = 5.1, 3.8 Hz, 1H), 6.81 (d, *J* = 7.7 Hz, 1H).

^{13}C NMR (101 MHz, CDCl$_3$) δ 189.4, 136.2, 132.9, 132.3, 129.2, 125.1, 113.2.

HRMS (ESI, m/z): Mass calcd. for $C_8H_6NOS^+$ [M+H]$^+$, 164.0165; found 164.0169.

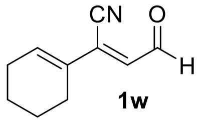

1w

(*Z*)-2-(cyclohex-1-en-1-yl)-4-oxobut-2-enenitrile (1w)

Purification by flash column chromatography on silica gel (petroleum ether / ethyl acetate = 10 / 1). Yellow solid, 75% yield, 3.8 g. m.p. 51-52 °C.

^{1}H NMR (400 MHz, CDCl$_3$) δ 10.14 (d, *J* = 7.8 Hz, 1H), 6.96 (t, *J* = 4.3 Hz, 1H), 6.45 (dd, *J* = 7.8, 1.0 Hz, 1H), 2.39 – 2.35 (m, 2H), 2.21 – 2.18 (m, 2H), 1.80 – 1.75 (m, 2H), 1.70 – 1.65 (m, 2H).

^{13}C NMR (101 MHz, CDCl$_3$) δ 190.8, 141.6, 133.3, 132.5, 132.2, 113.4, 26.7, 24.6, 21.7, 21.2.

HRMS (ESI, m/z): Mass calcd. for $C_{10}H_{12}NO^+$ [M+H]$^+$, 162.0913; found 162.0916.

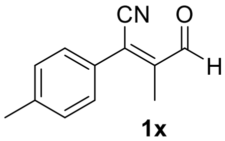

1x

(*Z*)-3-methyl-4-oxo-2-(p-tolyl)but-2-enenitrile (1x)

Purification by flash column chromatography on silica gel (petroleum ether / ethyl acetate = 20 / 1). Yellow oil, 81% yield, 1.61 g.

^{1}H NMR (400 MHz, CDCl$_3$) δ 10.35 (s, 0.85H, major), 9.73 (s, 0.15H, minor), 7.43 (d, J = 8.3 Hz, 2H), 7.34 – 7.28 (m, 2H), 2.43 (s, 2.49H, major), 2.42 (s, 0.57H, minor), 2.27 (s, 0.42H, minor), 2.08 (s, 2.63H, major).

^{13}C NMR (101 MHz, CDCl$_3$) δ 190.6, 147.6, 140.8, 129.5, 129.1, 128.8, 128.3, 115.2, 20.9, 12.5.

HRMS (ESI, m/z): Mass calcd. for $C_{12}H_{12}NO^+$ [M+H]$^+$, 186.0841; found 186.0913

2. Characterization of products

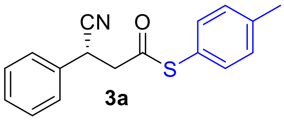
3a

***S*-(*p*-tolyl) (*R*)-3-cyano-3-phenylpropanethioate (3a)**

Purification by flash column chromatography on silica gel (petroleum ether / ethyl acetate = 10 / 1). Yellow solid, 83% yield, 23.4 mg, m.p. 94-96 °C.

$[\alpha]^{25}_D$ = -15.1 (*c* = 0.4 in $CHCl_3$).

^{1}H NMR (400 MHz, $CDCl_3$) δ 7.45 – 7.34 (m, 5H), 7.31 – 7.21 (m, 4H), 4.39 (t, *J* = 7.3 Hz, 1H), 3.34 (dd, *J* = 16.1, 7.8 Hz, 1H), 3.17 (dd, *J* = 16.2, 7.0 Hz, 1H), 2.40 (s, 3H).

^{13}C NMR (101 MHz, $CDCl_3$) δ 193.9, 140.4, 134.5, 134.1, 130.3, 129.4, 128.7, 127.5, 122.9, 119.6, 47.9, 33.1, 21.4.

HRMS (ESI, m/z): Mass calcd. for $C_{17}H_{16}NOS^+$ $[M+H]^+$, 282.0947; found 282.0943.

HPLC analysis (Chiralcel IA; 25 °C, IPA/Hexane = 05/95, 0.5 mL/min, 254 nm), Rt_1 (major) = 36.2 min, Rt_2 (minor) = 42.8 min; 95:5 er.

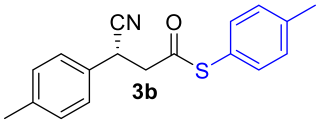
3b

***S*-(*p*-tolyl) (*R*)-3-cyano-3-(*p*-tolyl)propanethioate (3b)**

Purification by flash column chromatography on silica gel (petroleum ether / ethyl acetate = 10 / 1). White solid, 85% yield, 25.1 mg, m.p. 99-101 °C.

$[\alpha]^{25}_D$ = -61.3 (*c* = 0.6 in $CHCl_3$).

^{1}H NMR (400 MHz, $CDCl_3$) δ 7.29 – 7.21 (m, 8H), 4.35 (t, *J* = 7.4 Hz, 1H), 3.32 (dd, *J* = 16.2, 7.8 Hz, 1H), 3.15 (dd, *J* = 16.2, 7.0 Hz, 1H), 2.41 (s, 3H), 2.39 (s, 3H).

^{13}C NMR (101 MHz, $CDCl_3$) δ 194.0, 140.4, 138.6, 134.5, 131.1, 130.3, 130.0, 127.3, 122.9, 119.8, 48.0, 32.7, 21.4, 21.2.

HRMS (ESI, m/z): Mass calcd. for $C_{18}H_{18}NOS^+$ $[M+H]^+$, 318.0923; found 318.0927.

HPLC analysis (Chiralcel IA; 25 °C, IPA/Hexane = 05/95, 0.6 mL/min, 254 nm), Rt_1 (major) = 27.4 min, Rt_2 (minor) = 30.1 min; 96:4 er.

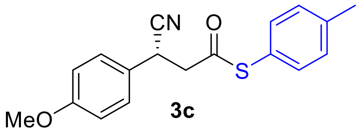
3c

***S*-(*p*-tolyl) (*R*)-3-cyano-3-(4-methoxyphenyl)propanethioate (3c)**

Purification by flash column chromatography on silica gel (petroleum ether / ethyl acetate = 10 / 1). White solid, 92% yield, 28.6 mg, m.p. 168-170 °C.;

$[\alpha]^{25}_D$ = -71.5 (*c* = 0.6 in $CHCl_3$).

^{1}H NMR (400 MHz, $CDCl_3$) δ 7.29 (d, *J* = 8.6 Hz, 2H), 7.28 – 7.18 (m, 4H), 6.93 (d, *J* = 8.7 Hz, 2H), 4.34 (t, *J* = 7.3 Hz, 1H), 3.84 (s, 3H), 3.31 (dd, *J* = 16.1, 7.5 Hz, 1H), 3.14 (dd, *J* = 16.1, 7.2 Hz, 1H), 2.40 (s, 3H).

^{13}C NMR (101 MHz, $CDCl_3$) δ 194.0, 159.7, 140.3, 134.4, 130.3, 128.7, 126.0, 122.9, 119.8, 114.6, 55.4, 48.1, 32.3, 21.4.

HRMS (ESI, m/z): Mass calcd. for $C_{18}H_{17}NNaO_2S^+$ $[M+Na]^+$, 334.0872; found 334.0872.

HPLC analysis (Chiralcel IA; 25 °C, IPA/Hexane = 05/95, 0.6 mL/min, 254 nm), Rt_1 (major) = 44.2

min, Rt$_2$ (minor) = 47.3 min; 96:4 er.

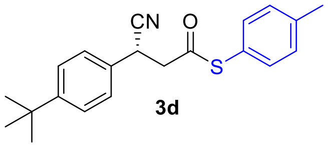

***S*-(*p*-tolyl) (*R*)-3-(4-(tert-butyl)phenyl)-3-cyanopropanethioate (3d)**

Purification by flash column chromatography on silica gel (petroleum ether / ethyl acetate = 10 / 1). White solid, 85% yield, 28.7mg, m.p. 81-82 °C.

[α]$^{25}_{D}$ = -37.0 (*c* = 0.6 in $CHCl_3$).

^{1}H NMR (400 MHz, $CDCl_3$) δ 7.43 (d, *J* = 8.4 Hz, 2H), 7.33 – 7.24 (m, 6H), 4.36 (dd, *J* = 8.1, 6.7 Hz, 1H), 3.34 (dd, *J* = 16.2, 8.1 Hz, 1H), 3.16 (dd, *J* = 16.2, 6.7 Hz, 1H), 2.41 (s, 3H), 1.35 (s, 9H).

^{13}C NMR (101 MHz, $CDCl_3$) δ 194.0, 151.8, 140.3, 134.5, 131.0, 130.3, 127.1, 126.3, 122.9, 119.7, 47.9, 34.7, 32.6, 31.3, 21.4.

HRMS (ESI, m/z): Mass calcd. for $C_{21}H_{23}NNaOS^+$ [M+Na]$^+$, 360.1393; found 360.1393.

HPLC analysis (Chiralcel OD-H; 25 °C, IPA/Hexane = 10/90, 0.5 mL/min, 254 nm), Rt$_1$ (major) = 44.4 min, Rt$_2$ (minor) = 59.3 min; 97:3 er.

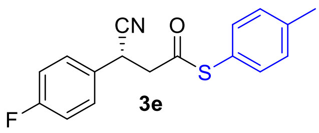

***S*-(*p*-tolyl) (*R*)-3-cyano-3-(4-fluorophenyl)propanethioate (3e)**

Purification by flash column chromatography on silica gel (petroleum ether / ethyl acetate = 10 / 1). Yellow oil, 89% yield, 26.6 mg.

[α]$^{25}_{D}$ = -35.9 (*c* = 0.5 in $CHCl_3$).

^{1}H NMR (400 MHz, $CDCl_3$) δ 7.37 – 7.31 (m, 2H), 7.23 (s, 4H), 7.12 – 7.04 (m, 2H), 4.35 (t, *J* = 7.3 Hz, 1H), 3.30 (dd, *J* = 16.1, 7.2 Hz, 1H), 3.13 (dd, *J* = 16.2, 7.4 Hz, 1H), 2.38 (s, 3H).

^{13}C NMR (101 MHz, $CDCl_3$) δ 193.8, 162.7 (d, *J* = 248.5 Hz), 140.5, 134.4, 130.3, 129.9 (d, *J* = 3.5 Hz), 129.4 (d, *J* = 8.4 Hz), 122.7, 119.4, 116.4 (d, *J* = 22.0 Hz), 47.8, 32.3, 21.4.

^{19}F NMR (377 MHz, $CDCl_3$) δ -112.65.

HRMS (ESI, m/z): Mass calcd. for $C_{17}H_{14}FNNaOS^+$ [M+Na]$^+$, 322.0672; found 322.0668.

HPLC analysis (Chiralcel AD-H; 25 °C, IPA/Hexane = 05/95, 0.5 mL/min, 254 nm), Rt$_1$ (major) = 41.8 min, Rt$_2$ (minor) = 51.7 min; 92:8 er.

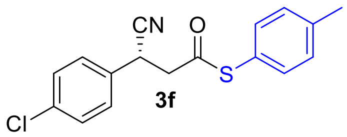

***S*-(*p*-tolyl) (*R*)-3-(4-chlorophenyl)-3-cyanopropanethioate (3f)**

Purification by flash column chromatography on silica gel (petroleum ether / ethyl acetate = 10 / 1). Yellow solid, 84% yield, 26.5 mg, m.p. 104-106 °C.

[α]$^{25}_{D}$ = -11.6 (*c* = 0.4 in $CHCl_3$).

^{1}H NMR (400 MHz, $CDCl_3$) δ 7.37 (d, *J* = 8.5 Hz, 2H), 7.30 (d, *J* = 8.5 Hz, 2H), 7.23 (s, 4H), 4.35 (t, *J* = 7.3 Hz, 1H), 3.30 (dd, *J* = 16.2, 7.2 Hz, 1H), 3.13 (dd, *J* = 16.2, 7.3 Hz, 1H), 2.38 (s, 3H).

^{13}C NMR (101 MHz, $CDCl_3$) δ 193.7, 140.5, 134.8, 134.4, 132.6, 130.3, 129.5, 128.9, 122.7, 119.2, 47.7, 32.4, 21.4.

HRMS (ESI, m/z): Mass calcd. for $C_{17}H_{14}ClNNaOS^+$ [M+Na]$^+$, 338.0377; found 338.0377.

HPLC analysis (Chiralcel IA; 25 °C, IPA/Hexane = 05/95, 0.6 mL/min, 254 nm), Rt_1 (major) = 36.0 min, Rt_2 (minor) = 45.6 min; 86:14 er.

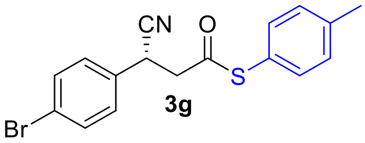

S-(*p*-tolyl) (*R*)-3-(4-bromophenyl)-3-cyanopropanethioate (3g)

Purification by flash column chromatography on silica gel (petroleum ether / ethyl acetate = 15 / 1). Yellow solid, 88% yield, 31.7 mg, m.p. 94-96 °C.

$[\alpha]^{25}_D$ = -76.3 (*c* = 0.5 in $CHCl_3$).

^{1}H NMR (400 MHz, $CDCl_3$) δ 7.55 (d, *J* = 8.5 Hz, 2H), 7.28 (d, *J* = 2.9 Hz, 6H), 4.36 (t, *J* = 7.2 Hz, 1H), 3.33 (dd, *J* = 16.3, 7.2 Hz, 1H), 3.15 (dd, *J* = 16.3, 7.3 Hz, 1H), 2.40 (s, 3H).

^{13}C NMR (101 MHz, $CDCl_3$) δ 193.7, 140.5, 134.4, 133.1, 132.5, 130.3, 129.2, 122.9, 122.6, 119.1, 47.6, 32.5, 21.4.

HRMS (ESI, m/z): Mass calcd. for $C_{17}H_{14}BrNNaOS^+$ $[M+Na]^+$, 381.9872; found 381.9869.

HPLC analysis (Chiralcel IA; 25 °C, IPA/Hexane = 05/95, 0.6 mL/min, 254 nm), Rt_1 (major) = 42.6 min, Rt_2 (minor) = 52.7 min; 80:20 er.

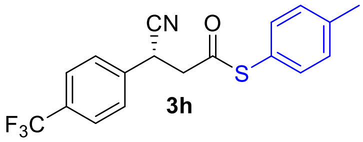

S-(*p*-tolyl) (*R*)-3-cyano-3-(4-(trifluoromethyl)phenyl)propanethioate (3h)

Purification by flash column chromatography on silica gel (petroleum ether / ethyl acetate = 3 / 1). Yellow oil, 72% yield, 25.2 mg.

$[\alpha]^{25}_D$ = -12.1 (*c* = 0.4 in $CHCl_3$).

^{1}H NMR (400 MHz, $CDCl_3$) δ 7.43 (d, *J* = 8.7 Hz, 2H), 7.27 (d, *J* = 11.2 Hz, 6H), 4.41 (t, *J* = 7.3 Hz, 1H), 3.35 (dd, *J* = 16.3, 7.3 Hz, 1H), 3.18 (dd, *J* = 16.3, 7.3 Hz, 1H), 2.41 (s, 3H).

^{13}C NMR (101 MHz, $CDCl_3$) δ 193.8, 149.3 (q, *J* = 1.9 Hz), 140.5, 134.4, 132.8, 130.3, 129.2, 120.4 (q, *J* = 257.8 Hz), 122.6, 121.8, 119.2, 47.6, 32.4, 21.4.

^{19}F NMR (377 MHz, $CDCl_3$) δ -57.87.

HRMS (ESI, m/z): Mass calcd. for $C_{18}H_{14}F_3NNaOS^+$ $[M+Na]^+$, 372.0640; found 372.0639.

HPLC analysis (Chiralcel IA; 25 °C, IPA/Hexane = 05/95, 0.6 mL/min, 254 nm), Rt_1 (major) = 29.8 min, Rt_2 (minor) = 36.5 min; 77:23 er.

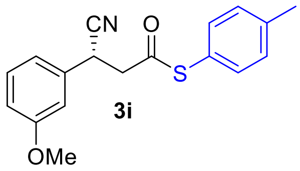

S-(*p*-tolyl) (*R*)-3-cyano-3-(3-methoxyphenyl)propanethioate (3i)

Purification by flash column chromatography on silica gel (petroleum ether / ethyl acetate = 10 / 1). White solid, 91% yield, 28.3 mg, m.p. 107-108 °C.

$[\alpha]^{25}_D$ = -21.0 (*c* = 0.4 in $CHCl_3$).

^{1}H NMR (400 MHz, $CDCl_3$) δ 7.32 – 7.20 (m, 5H), 6.96 – 6.85 (m, 3H), 4.32 (dd, *J* = 7.9, 6.8 Hz, 1H), 3.81 (s, 3H), 3.30 (dd, *J* = 16.1, 7.9 Hz, 1H), 3.13 (dd, *J* = 16.1, 6.8 Hz, 1H), 2.37 (s, 3H).

^{13}C NMR (101 MHz, CDCl$_3$) δ 193.9, 160.2, 140.4, 135.5, 134.5, 130.4, 130.3, 122.9, 119.6, 119.5, 114.2, 113.1, 55.4, 47.9, 33.1, 21.4.

HRMS (ESI, m/z): Mass calcd. for $C_{18}H_{17}NNaO_2S^+$ [M+Na]$^+$, 334.0872; found 334.0868.

HPLC analysis (Chiralcel IA; 25 °C, IPA/Hexane = 10/90, 0.6 mL/min, 254 nm), Rt$_1$ (major) = 29.2 min, Rt$_2$ (minor) = 31.2 min; 95:5 er.

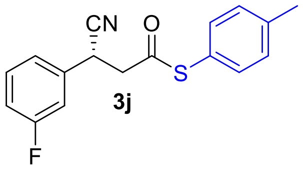

3j

***S*-(*p*-tolyl) (*R*)-3-cyano-3-(3-fluorophenyl)propanethioate (3j)**

Purification by flash column chromatography on silica gel (petroleum ether / ethyl acetate = 10 / 1). Yellow oil, 85% yield, 25.4 mg.

[α]$^{25}_{D}$ = -26.5 (*c* = 0.8 in CHCl$_3$).

^{1}H NMR (400 MHz, CDCl$_3$) δ 7.40 – 7.35 (m, 1H), 7.24 (s, 4H), 7.17 – 7.14 (m, 1H), 7.11 – 7.04 (m, 2H), 4.37 (t, *J* = 7.2 Hz, 1H), 3.32 (dd, *J* = 16.2, 7.4 Hz, 1H), 3.15 (dd, *J* = 16.2, 7.1 Hz, 1H), 2.38 (s, 3H).

^{13}C NMR (101 MHz, CDCl$_3$) δ 193.7, 163.0 (d, *J* = 244.4 Hz), 140.5, 136.4 (d, *J* = 7.5 Hz), 134.4, 131.0 (d, *J* = 8.3 Hz), 130.3, 123.2 (d, *J* = 3.0 Hz), 122.7, 119.0, 115.8 (d, *J* = 20.9 Hz), 114.8 (d, *J* = 22.9 Hz). 47.6, 32.7, 21.4.

^{19}F NMR (377 MHz, CDCl$_3$) δ -110.93.

HRMS (ESI, m/z): Mass calcd. for $C_{17}H_{14}FNNaOS^+$ [M+Na]$^+$, 322.0672; found 322.0671.

HPLC analysis (Chiralcel IA; 25 °C, IPA/Hexane = 05/95, 0.6 mL/min, 254 nm), Rt$_1$ (major) = 39.2 min, Rt$_2$ (minor) = 45.6 min; 92:8 er.

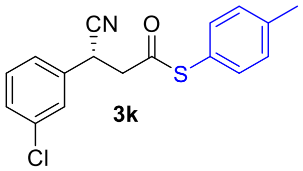

3k

***S*-(*p*-tolyl) (*R*)-3-(3-chlorophenyl)-3-cyanopropanethioate (3k)**

Purification by flash column chromatography on silica gel (petroleum ether / ethyl acetate = 10 / 1). Yellow solid, 85% yield, 28.6 mg, m.p. 99-101 °C.

[α]$^{25}_{D}$ = -9.8 (*c* = 0.4 in CHCl$_3$).

^{1}H NMR (400 MHz, CDCl$_3$) δ 7.41 – 7.32 (m, 3H), 7.30 – 7.24 (m, 5H), 4.37 (t, *J* = 7.3 Hz, 1H), 3.34 (dd, *J* = 16.2, 7.4 Hz, 1H), 3.16 (dd, *J* = 16.2, 7.1 Hz, 1H), 2.41 (s, 3H).

^{13}C NMR (101 MHz, CDCl$_3$) δ 193.7, 140.5, 136.0, 135.2, 134.5, 130.6, 130.3, 129.0, 127.8, 125.7, 122.7, 119.0, 47.6, 32.7, 21.4.

HRMS (ESI, m/z): Mass calcd. for $C_{17}H_{14}ClNNaOS^+$ [M+Na]$^+$, 338.0377; found 338.0369.

HPLC analysis (Chiralcel IA; 25 °C, IPA/Hexane = 05/95, 0.6 mL/min, 254 nm), Rt$_1$ (major) = 36.0 min, Rt$_2$ (minor) = 45.6 min; 86:14 er.

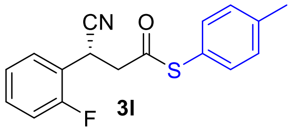

3l

***S*-(*p*-tolyl) (*R*)-3-cyano-3-(2-fluorophenyl)propanethioate (3l)**

Purification by flash column chromatography on silica gel (petroleum ether / ethyl acetate = 15 / 1). Yellow oil, 93% yield, 27.8 mg.

[α]$^{25}_{D}$ = -42.6 (*c* = 0.6 in CHCl$_3$).

^{1}H NMR (400 MHz, CDCl$_3$) δ 7.46 (td, *J* = 7.6, 1.8 Hz, 1H), 7.39 – 7.33 (m, 1H), 7.26 – 7.16 (m, 5H), 7.14 – 7.09 (m, 1H), 4.57 (dd, *J* = 8.2, 6.2 Hz, 1H), 3.31 (dd, *J* = 16.3, 8.2 Hz, 1H), 3.20 (dd, *J* = 16.3, 6.2 Hz, 1H), 2.37 (s, 3H).

^{13}C NMR (101 MHz, CDCl$_3$) δ 193.5, 160.0 (d, *J* = 248.7 Hz), 140.4, 134.5, 130.8 (d, *J* = 8.3 Hz), 130.3, 129.5 (d, *J* = 3.1 Hz), 125.0 (d, *J* = 3.6 Hz), 122.8, 121.3 (d, *J* = 13.7 Hz), 118.5, 116.2 (d, *J* = 21.0 Hz), 45.8, 27.6, 21.4.

^{19}F NMR (377 MHz, CDCl$_3$) δ -116.94.

HRMS (ESI, m/z): Mass calcd. for $C_{17}H_{14}FNNaOS^+$ $[M+Na]^+$, 322.0672; found 322.0668.

HPLC analysis (Chiralcel IA; 25 °C, IPA/Hexane = 05/95, 0.6 mL/min, 254 nm), Rt$_1$ (major) = 48.8 min, Rt$_2$ (minor) = 65.7 min; 91:9 er.

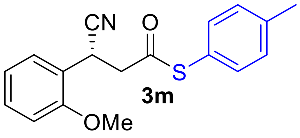

S-(*p*-tolyl) (*R*)-3-cyano-3-(2-methoxyphenyl)propanethioate (3m)

Purification by flash column chromatography on silica gel (petroleum ether / ethyl acetate = 10 / 1). White solid, 88% yield, 27.4 mg, m.p. 108-109 °C.

[α]$^{25}_{D}$ = -31.8 (*c* = 0.4 in CHCl$_3$).

^{1}H NMR (400 MHz, CDCl$_3$) δ 7.38 (dd, *J* = 7.6, 1.7 Hz, 1H), 7.32 (ddd, *J* = 8.2, 7.5, 1.7 Hz, 1H), 7.26 (d, *J* = 8.2 Hz, 2H), 7.21 (d, *J* = 8.2 Hz, 2H), 6.97 (td, *J* = 7.5, 1.1 Hz, 1H), 6.91 (dd, *J* = 8.3, 1.1 Hz, 1H), 4.59 (dd, *J* = 8.3, 6.0 Hz, 1H), 3.87 (s, 3H), 3.28 – 3.16 (m, 2H), 2.36 (s, 3H).

^{13}C NMR (101 MHz, CDCl$_3$) δ 194.2, 156.3, 140.1, 134.5, 130.2, 130.1, 129.0, 123.2, 122.1, 121.1, 119.6, 111.0, 55.6, 45.5, 28.5, 21.4.

HRMS (ESI, m/z): Mass calcd. for $C_{18}H_{17}NNaO_2S^+$ $[M+Na]^+$, 334.0872; found 334.0884.

HPLC analysis (Chiralcel OD-H; 25 °C, IPA/Hexane = 15/85, 0.6 mL/min, 254 nm), Rt$_1$ (major) = 49.2 min, Rt$_2$ (minor) = 79.0 min; 90:10 er.

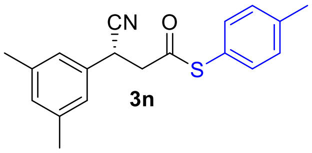

S-(*p*-tolyl) (*R*)-3-cyano-3-(3,5-dimethylphenyl)propanethioate (3n)

Purification by flash column chromatography on silica gel (petroleum ether / ethyl acetate = 10 / 1). Yellow solid, 82% yield, 25.4 mg, m.p. 103-104 °C.

[α]$^{25}_{D}$ = -37.1 (*c* = 0.8 in CHCl$_3$).

^{1}H NMR (400 MHz, CDCl$_3$) δ 7.27 – 7.21 (m, 4H), 6.96 (d, *J* = 7.3 Hz, 3H), 4.27 (dd, *J* = 8.0, 6.7 Hz, 1H), 3.28 (dd, *J* = 16.1, 8.1 Hz, 1H), 3.10 (dd, *J* = 16.1, 6.6 Hz, 1H), 2.38 (s, 3H), 2.32 (s, 6H).

^{13}C NMR (101 MHz, CDCl$_3$) δ 194.0, 140.3, 139.1, 134.5, 134.0, 130.2, 125.2, 123.0, 119.8, 48.0, 33.0, 21.4, 21.3.

HRMS (ESI, m/z): Mass calcd. for $C_{19}H_{19}NNaOS^+$ $[M+Na]^+$, 332.1080; found 332.1078.

HPLC analysis (Chiralcel IF; 25 °C, IPA/Hexane = 05/95, 0.5 mL/min, 254 nm), Rt$_1$ (major) = 35.7 min, Rt$_2$ (minor) = 43.2 min; 96:4 er.

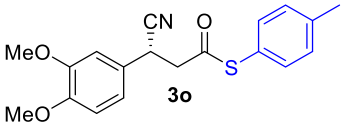

S-(p-tolyl) (R)-3-cyano-3-(3,4-dimethoxyphenyl)propanethioate (3o)

Purification by flash column chromatography on silica gel (petroleum ether / ethyl acetate = 5 / 1). Yellow solid, 90% yield, 30.7 mg, m.p. 121-122 ºC.

[α]^{25}D = -23.1 (*c* = 0.6 in $CHCl_3$).

^{1}H NMR (400 MHz, $CDCl_3$) δ 7.25 (s, 4H), 6.95 – 6.83 (m, 3H), 4.33 (t, *J* = 7.3 Hz, 1H), 3.91 (d, *J* = 1.7 Hz, 6H), 3.32 (dd, *J* = 16.0, 7.5 Hz, 1H), 3.15 (dd, *J* = 16.1, 7.2 Hz, 1H), 2.40 (s, 3H).

^{13}C NMR (101 MHz, $CDCl_3$) δ 194.0, 149.4, 149.2, 140.4, 134.4, 130.3, 126.4, 122.9, 119.8, 119.8, 111.5, 110.3, 56.0, 56.0, 48.0, 32.7, 21.4.

HRMS (ESI, m/z): Mass calcd. for $C_{19}H_{19}NNaO_3S$+ [M+Na]$^+$, 364.0978; found 364.0980.

HPLC analysis (Chiralcel IA; 25 ºC, IPA/Hexane = 05/95, 0.6 mL/min, 254 nm), Rt_1 (major) = 70.8 min, Rt_2 (minor) = 77.4 min; 97:3 er.

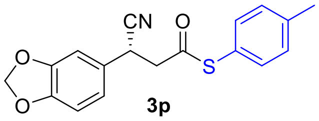

S-(p-tolyl) (R)-3-(benzo[d][1,3]dioxol-5-yl)-3-cyanopropanethioate (3p)

Purification by flash column chromatography on silica gel (petroleum ether / ethyl acetate = 10 / 1). Yellow solid, 86% yield, 28.0 mg, m.p. 125-127 ºC.

[α]^{25}D = -36.0 (*c* = 0.4 in $CHCl_3$).

^{1}H NMR (400 MHz, $CDCl_3$) δ 7.26 – 7.20 (m, 4H), 6.88 – 6.70 (m, 3H), 5.99 (s, 2H), 4.27 (t, *J* = 7.3 Hz, 1H), 3.27 (dd, *J* = 16.1, 7.4 Hz, 1H), 3.10 (dd, *J* = 16.1, 7.2 Hz, 1H), 2.38 (s, 3H).

^{13}C NMR (101 MHz, $CDCl_3$) δ 193.9, 148.4, 147.9, 140.4, 134.4, 130.3, 127.6, 122.9, 121.1, 119.6, 108.8, 107.8, 101.6, 48.0, 32.8, 21.4.

HRMS (ESI, m/z): Mass calcd. for $C_{18}H_{15}NNaO_3S^+$ [M+Na]$^+$, 348.0665; found 348.0664.

HPLC analysis (Chiralcel IA; 25 ºC, IPA/Hexane = 05/95, 0.5 mL/min, 254 nm), Rt_1 (major) = 87.9 min, Rt_2 (minor) = 95.7 min; 96:4 er.

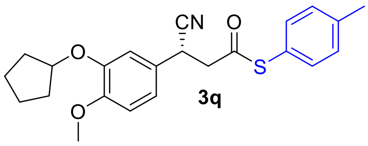

S-(p-tolyl) (R)-3-cyano-3-(3-(cyclopentyloxy)-4-methoxyphenyl) propanethioate (3q)

Purification by flash column chromatography on silica gel (petroleum ether / ethyl acetate = 10 / 1). Yellow solid, 81% yield, 32.0 mg, m.p. 107-108 ºC.

[α]^{25}D = -61.9 (*c* = 0.4 in $CHCl_3$).

^{1}H NMR (400 MHz, $CDCl_3$) δ 7.26 – 7.20 (m, 4H), 6.90 – 6.80 (m, 3H), 4.79 – 4.74 (m, 1H), 4.29 (t, *J* = 7.3 Hz, 1H), 3.85 (s, 3H), 3.28 (dd, *J* = 16.0, 7.4 Hz, 1H), 3.12 (dd, *J* = 16.0, 7.3 Hz, 1H), 2.38 (s, 3H), 1.98 – 1.81 (m, 6H), 1.64 – 1.57 (m, 2H).

^{13}C NMR (101 MHz, $CDCl_3$) δ 194.0, 150.2, 148.1, 140.3, 134.4, 130.2, 126.2, 122.9, 119.8, 119.6, 113.9, 112.2, 80.6, 56.1, 48.1, 32.8, 32.7, 32.7, 24.1, 21.4.

HRMS (ESI, m/z): Mass calcd. for $C_{23}H_{25}NNaO_3S^+$ [M+Na]$^+$, 418.1447; found 418.1447.

HPLC analysis (Chiralcel IA; 25 °C, IPA/Hexane = 05/95, 0.6 mL/min, 254 nm), Rt_1 (major) = 49.4 min, Rt_2 (minor) = 40.0 min; 96:4 er.

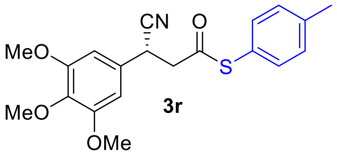

3r

***S*-(*p*-tolyl) (*R*)-3-cyano-3-(3,4,5-trimethoxyphenyl)propanethioate (3r)**

Purification by flash column chromatography on silica gel (petroleum ether / ethyl acetate = 5 / 1). Yellow solid, 94% yield, 34.9 mg, m.p. 116-118 °C.

[α]$^{25}_{D}$ = -184.3 (*c* = 0.4 in $CHCl_3$).

^{1}H NMR (400 MHz, $CDCl_3$) δ 7.29 – 7.24 (m, 4H), 6.56 (s, 2H), 4.33 (t, *J* = 7.3 Hz, 1H), 3.89 (s, 6H), 3.87 (s, 3H), 3.33 (dd, *J* = 16.1, 7.7 Hz, 1H), 3.17 (dd, *J* = 16.1, 7.0 Hz, 1H), 2.40 (s, 3H).

^{13}C NMR (101 MHz, $CDCl_3$) δ 194.0, 153.8, 140.5, 138.0, 134.4, 130.3, 129.6, 122.8, 119.5, 104.4, 60.9, 56.3, 48.0, 33.3, 21.4.

HRMS (ESI, m/z): $C_{20}H_{21}NNaO_4S^+$ $[M+Na]^+$, 394.1083; found 394.1092.

HPLC analysis (Chiralcel IB; 25 °C, IPA/Hexane = 10/90, 0.8 mL/min, 254 nm), Rt_1 (major) = 32.8 min, Rt_2 (minor) = 53.6 min; 96:4 er.

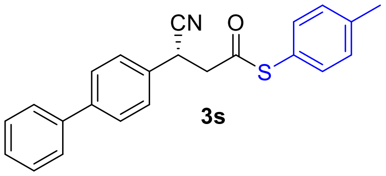

3s

***S*-(*p*-tolyl) (*R*)-3-([1,1'-biphenyl]-4-yl)-3-cyanopropanethioate (3s)**

Purification by flash column chromatography on silica gel (petroleum ether / ethyl acetate = 10 / 1). Yellow solid, 92% yield, 32.9 mg, m.p. 97-98 °C.

[α]$^{25}_{D}$ = -46.3 (*c* = 0.4 in $CHCl_3$).

^{1}H NMR (400 MHz, $CDCl_3$) δ 7.65 – 7.54 (m, 4H), 7.49 – 7.35 (m, 5H), 7.27 – 7.22 (m, 4H), 4.41 (t, *J* = 7.3 Hz, 1H), 3.36 (dd, *J* = 16.2, 7.6 Hz, 1H), 3.19 (dd, *J* = 16.2, 7.0 Hz, 1H), 2.38 (s, 3H).

^{13}C NMR (101 MHz, $CDCl_3$) δ 193.9, 141.7, 140.4, 140.1, 134.5, 133.0, 130.3, 128.9, 128.0, 127.9, 127.8, 127.1, 122.9, 119.5, 47.9, 32.7, 21.4.

HRMS (ESI, m/z): Mass calcd. for $C_{23}H_{19}NNaOS^+$ $[M+Na]^+$, 380.1080; found 380.1074.

HPLC analysis (Chiralcel OD-H; 25 °C, IPA/Hexane = 20/80, 0.6 mL/min, 254 nm), Rt_1 (major) = 31.5 min, Rt_2 (minor) = 47.8 min; 96:4 er.

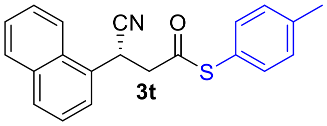

3t

***S*-(*p*-tolyl) (*R*)-3-cyano-3-(naphthalen-1-yl)propanethioate (3t)**

Purification by flash column chromatography on silica gel (petroleum ether / ethyl acetate = 10 / 1). Yellow solid, 90% yield, 29.7 mg, m.p. 84-85 °C.

[α]$^{25}_{D}$ = -16.3 (*c* = 0.4 in $CHCl_3$).

^{1}H NMR (400 MHz, $CDCl_3$) δ 7.97 – 7.84 (m, 3H), 7.75 – 7.69 (m, 1H), 7.63 – 7.48 (m, 3H), 7.28 – 7.22 (m, 4H), 5.12 (dd, *J* = 9.2, 5.0 Hz, 1H), 3.42 (dd, *J* = 16.3, 9.2 Hz, 1H), 3.28 (dd, *J* = 16.3, 5.0 Hz, 1H), 2.38 (s, 3H).

^{13}C NMR (101 MHz, CDCl$_3$) δ 194.2, 140.4, 134.5, 134.1, 130.3, 129.7, 129.6, 129.5, 127.4, 126.4, 126.0, 125.5, 122.9, 121.9, 119.6, 47.1, 30.3, 21.4.

HRMS (ESI, m/z): Mass calcd. for $C_{21}H_{17}NNaOS^+$ [M+Na]$^+$, 354.0923; found 354.0917.

HPLC analysis (Chiralcel IG; 25 °C, IPA/Hexane = 10/90, 0.6 mL/min, 254 nm), Rt$_1$ (major) = 61.9 min, Rt$_2$ (minor) = 67.1 min; 94:6 er.

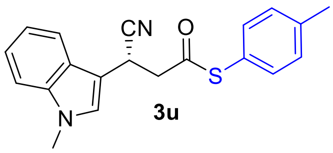

3u

***S*-(*p*-tolyl) (*R*)-3-cyano-3-(1-methyl-1H-indol-3-yl)propanethioate (3u)**

Purification by flash column chromatography on silica gel (petroleum ether / ethyl acetate = 5 / 1). Brown solid, 86% yield, 28.8 mg, m.p. 127-128 °C.

[α]$^{25}_{D}$ = -90.5 (*c* = 0.4 in CHCl$_3$).

^{1}H NMR (400 MHz, CDCl$_3$) δ 7.62 (dt, *J* = 8.0, 1.0 Hz, 1H), 7.35 – 7.26 (m, 2H), 7.24 – 7.15 (m, 5H), 7.10 (s, 1H), 4.62 (t, *J* = 6.8 Hz, 1H), 3.75 (s, 3H), 3.35 (dd, *J* = 16.1, 7.7 Hz, 1H), 3.28 (dd, *J* = 16.2, 6.7 Hz, 1H), 2.36 (s, 3H).

^{13}C NMR (101 MHz, CDCl$_3$) δ 194.3, 140.2, 137.3, 134.5, 130.2, 127.5, 125.4, 123.2, 122.5, 120.0, 119.8, 118.5, 109.9, 107.2, 46.9, 32.9, 24.9, 21.4.

HRMS (ESI, m/z): Mass calcd. for $C_{20}H_{18}N_2NaOS^+$ [M+Na]$^+$, 357.1032; found 357.1031.

HPLC analysis (Chiralcel AS-H; 25 °C, IPA/Hexane = 30/70, 0.6 mL/min, 254 nm), Rt$_1$ (major) = 50.4 min, Rt$_2$ (minor) = 58.2min; 96:4 er.

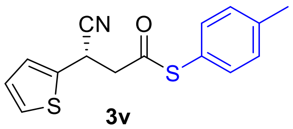

3v

***S*-(*p*-tolyl) (*S*)-3-cyano-3-(thiophen-2-yl)propanethioate (3v)**

Purification by flash column chromatography on silica gel (petroleum ether / ethyl acetate =5 / 1). Yellow oil, 83% yield, 23.9 mg.

[α]$^{25}_{D}$ = -8.7 (*c* = 0.4 in CHCl$_3$).

^{1}H NMR (400 MHz, CDCl$_3$) δ 7.31 – 7.22 (m, 5H), 7.10 (dt, *J* = 3.6, 1.1 Hz, 1H), 6.99 (dd, *J* = 5.2, 3.6 Hz, 1H), 4.65 (td, *J* = 7.3, 0.8 Hz, 1H), 3.36 (dd, *J* = 16.2, 7.3 Hz, 1H), 3.24 (dd, *J* = 16.3, 7.1 Hz, 1H), 2.38 (s, 3H).

^{13}C NMR (101 MHz, CDCl$_3$) δ 193.5, 140.4, 135.8, 134.4, 130.3, 127.3, 127.0, 126.2, 122.8, 118.7, 48.0, 28.3, 21.4.

HRMS (ESI, m/z): Mass calcd. for $C_{15}H_{13}NNaOS_2^+$ [M+Na]$^+$, 310.0331; found 310.0330.

HPLC analysis (Chiralcel IA; 25 °C, IPA/Hexane = 05/95, 0.5 mL/min, 254 nm), Rt$_1$ (major) = 42.9 min, Rt$_2$ (minor) = 46.3 min; 75:25 er.

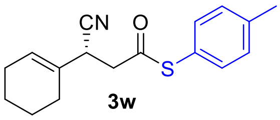

3w

***S*-(*p*-tolyl) (*R*)-3-cyano-3-(cyclohex-1-en-1-yl)propanethioate (3w)**

Purification by flash column chromatography on silica gel (petroleum ether / ethyl acetate =5 / 1). Yellow soild, 47% yield, 13.4 mg, m.p. 68-70 °C.

[α]$^{25}_{D}$ = -11.4 (*c* = 0.4 in CHCl$_3$).

^{1}H NMR (400 MHz, $CDCl_3$) δ 7.30 – 7.21 (m, 4H), 5.82 (d, *J* = 1.1 Hz, 1H), 3.67 (t, *J* = 7.4 Hz, 1H), 3.05 (dd, *J* = 15.8, 7.7 Hz, 1H), 2.98 (dd, *J* = 15.8, 7.0 Hz, 1H), 2.38 (s, 3H), 2.14 – 1.97 (m, 4H), 1.73 – 1.66 (m, 2H), 1.61 – 1.55 (m, 2H).

^{13}C NMR (101 MHz, $CDCl_3$) δ 194.1, 140.3, 134.4, 130.2, 130.0, 127.8, 123.1, 119.3, 44.2, 35.0, 25.9, 25.2, 22.4, 21.7, 21.4.

HRMS (ESI, m/z): Mass calcd. for $C_{17}H_{19}NNaOS^+$ $[M+Na]^+$, 308.1080; found 308.1078.

HPLC analysis (Chiralcel AS3RCD-BR007; 25 °C, Water (20mM, Ammonium bicarbonate)/Acetonitrile =30/70, 0.6 mL/min, 254 nm), Rt_1 (major) = 7.8 min, Rt_2 (minor) = 7.0 min; 80:20 er.

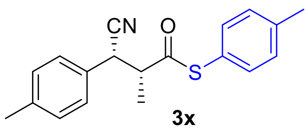

***S*-(*p*-tolyl) (2*R*,3*R*)-3-cyano-2-methyl-3-(*p*-tolyl)propanethioate (3x)**

Purification by flash column chromatography on silica gel (petroleum ether / ethyl acetate =10 / 1). Yellow soild, 79% yield, 24.3 mg, m.p. 121-123 °C, > 20:1 dr.

$[\alpha]^{25}_D$ = -16.5 (*c* = 0.5 in $CHCl_3$).

^{1}H NMR (400 MHz, $CDCl_3$) δ 7.24 – 7.15 (m, 6H), 7.09 (d, *J* = 8.2 Hz, 2H), 4.19 (d, *J* = 8.5 Hz, 1H), 3.18 – 3.10 (m, 1H), 2.37 (s, 3H), 2.36 (s, 3H), 1.51 (d, *J* = 7.0 Hz, 3H).

^{13}C NMR (101 MHz, $CDCl_3$) δ 196.3, 137.8, 136.1, 132.0, 128.2, 127.8, 127.4, 125.6, 120.7, 116.7, 50.1, 37.9, 19.0, 18.8, 13.7.

HRMS (ESI, m/z): Mass calcd. for $C_{19}H_{20}NOS^+$ $[M+H]^+$, 310.1260; found 310.1247

HPLC analysis (Chiralcel IF00CE-TE005; 35 °C, N-Hexane/Ethanol/Diethylamine =70/30/0.1, 1.0 mL/min, 254 nm), Rt_1 (major) = 4.9 min, Rt_2 (minor) = 5.5 min; 90:10 er.

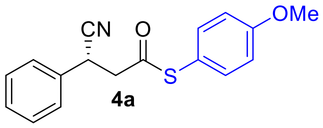

***S*-(4-methoxyphenyl) (*R*)-3-cyano-3-phenylpropanethioate (4a)**

Purification by flash column chromatography on silica gel (petroleum ether / ethyl acetate = 5 / 1). Yellow solid, 88% yield, 26.2 mg, m.p. 112-114°C.

$[\alpha]^{25}_D$ = -46.6 (*c* = 0.6 in $CHCl_3$).

^{1}H NMR (400 MHz, $CDCl_3$) δ 7.45 – 7.36 (m, 5H), 7.30 – 7.27 (m, 2H), 6.96 (d, *J* = 8.8 Hz, 2H), 4.39 (t, *J* = 7.3 Hz, 1H), 3.85 (s, 3H), 3.33 (dd, *J* = 16.1, 7.8 Hz, 1H), 3.16 (dd, *J* = 16.2, 6.9 Hz, 1H).

^{13}C NMR (101 MHz, $CDCl_3$) δ 194.5, 161.0, 136.1, 134.1, 129.4, 128.7, 127.5, 119.6, 117.0, 115.1, 55.4, 47.8, 33.1.

HRMS (ESI, m/z): Mass calcd. for $C_{17}H_{16}NO_2S^+$ $[M+Na]^+$, 298.0896; found 298.0902.

HPLC analysis (Chiralcel IA; 25 °C, IPA/Hexane = 05/95, 0.6 mL/min, 254 nm), Rt_1 (major) = 57.9 min, Rt_2 (minor) = 67.7 min; 96:4 er.

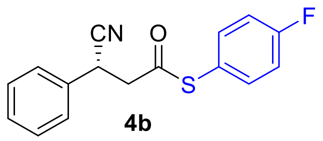

4b

***S*-(4-fluorophenyl) (*R*)-3-cyano-3-phenylpropanethioate (4b)**

Purification by flash column chromatography on silica gel (petroleum ether / ethyl acetate = 10 / 1). Yellow solid, 87% yield, 24.8 mg, m.p. 83-84 °C.

[α]$^{25}_{D}$ = -38.7 (*c* = 0.6 in $CHCl_3$).

^{1}H NMR (400 MHz, $CDCl_3$) δ 7.46 – 7.32 (m, 7H), 7.19 – 7.10 (m, 2H), 4.39 (dd, *J* = 7.9, 6.8 Hz, 1H), 3.36 (dd, *J* = 16.2, 8.0 Hz, 1H), 3.18 (dd, *J* = 16.2, 6.8 Hz, 1H).

^{13}C NMR (101 MHz, $CDCl_3$) δ 193.4, 163.8 (d, *J* = 251.1 Hz), 136.6 (d, *J* = 8.7 Hz), 133.9, 129.4, 128.8, 127.5, 121.6 (d, *J* = 3.5 Hz), 119.5, 116.8 (d, *J* = 3.5 Hz), 48.0, 33.1.

^{19}F NMR (377 MHz, $CDCl_3$) δ -110.05.

HRMS (ESI, m/z): Mass calcd. for $C_{16}H_{12}FNNaOS^+$ $[M+Na]^+$, 308.0516; found 308.0515.

HPLC analysis (Chiralcel IA; 25 °C, IPA/Hexane = 05/95, 0.7 mL/min, 254 nm), Rt_1 (major) = 30.6 min, Rt_2 (minor) = 35.6 min; 93:7 er.

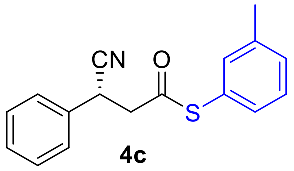

4c

***S*-(m-tolyl) (*R*)-3-cyano-3-phenylpropanethioate (4c)**

Purification by flash column chromatography on silica gel (petroleum ether / ethyl acetate = 10 / 1). Yellow solid, 86% yield, 24.2 mg, m.p. 95-97 °C.

[α]$^{25}_{D}$ = -78.2 (*c* = 0.6 in $CHCl_3$).

^{1}H NMR (400 MHz, $CDCl_3$) δ 7.44 – 7.32 (m, 5H), 7.34 – 7.26 (m, 1H), 7.27 – 7.20 (m, 1H), 7.21 – 7.12 (m, 2H), 4.36 (dd, *J* = 7.9, 6.8 Hz, 1H), 3.31 (dd, *J* = 16.1, 7.9 Hz, 1H), 3.14 (dd, *J* = 16.2, 6.8 Hz, 1H), 2.36 (s, 3H).

^{13}C NMR (101 MHz, $CDCl_3$) δ 193.6, 139.4, 135.0, 134.1, 131.5, 130.8, 129.4, 129.2, 128.7, 127.5, 126.0, 119.5, 48.0, 33.1, 21.3.

HRMS (ESI, m/z): Mass calcd. for $C_{17}H_{15}NNaOS^+$ $[M+Na]^+$, 304.0767; found 304.0763.

HPLC analysis (Chiralcel IA; 25 °C, IPA/Hexane = 05/95, 0.6 mL/min, 254 nm), Rt_1 (major) = 28.3 min, Rt_2 (minor) = 34.6 min; 95:5 er.

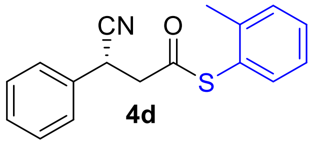

4d

***S*-(o-tolyl) (*R*)-3-cyano-3-phenylpropanethioate (4d)**

Purification by flash column chromatography on silica gel (petroleum ether / ethyl acetate = 10 / 1). Yellow solid, 81% yield, 22.8 mg, m.p. 98-99 °C.

[α]$^{25}_{D}$ = -10.6 (*c* = 0.4 in $CHCl_3$).

^{1}H NMR (400 MHz, $CDCl_3$) δ 7.46 – 7.32 (m, 8H), 7.27 – 7.22 (m, 1H), 4.40 (t, *J* = 7.4 Hz, 1H), 3.36 (dd, *J* = 16.0, 7.7 Hz, 1H), 3.20 (dd, *J* = 15.9, 7.2 Hz, 1H), 2.26 (s, 3H).

^{13}C NMR (101 MHz, $CDCl_3$) δ 192.9, 142.1, 135.8, 134.0, 131.0, 130.6, 129.4, 128.7, 127.5, 126.8, 119.5, 47.9, 33.2, 20.6.

HRMS (ESI, m/z): Mass calcd. for $C_{17}H_{15}NNaOS^+$ $[M+Na]^+$, 304.0767; found 304.0766.

HPLC analysis (Chiralcel IA; 25 °C, IPA/Hexane = 05/95, 0.6 mL/min, 254 nm), Rt_1 (major) = 27.7 min, Rt_2 (minor) = 30.3 min; 94:6 er.

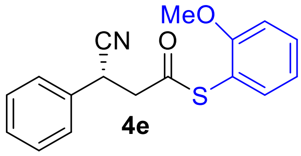
4e

***S*-(2-methoxyphenyl) (*R*)-3-cyano-3-phenylpropanethioate (4e)**

Purification by flash column chromatography on silica gel (petroleum ether / ethyl acetate = 5 / 1). Yellow solid, 74% yield, 22.0 mg, m.p. 104-106°C.

[α]$^{25}_{D}$ = -41.1 (*c* = 0.5 in $CHCl_3$).

^{1}H NMR (400 MHz, $CDCl_3$) δ 7.48 – 7.34 (m, 7H), 7.04 – 6.97 (m, 2H), 4.40 (t, *J* = 7.3 Hz, 1H), 3.84 (s, 3H), 3.36 (dd, *J* = 16.2, 7.7 Hz, 1H), 3.19 (dd, *J* = 16.2, 6.9 Hz, 1H).

^{13}C NMR (101 MHz, $CDCl_3$) δ 192.7, 159.2, 136.6, 134.2, 132.3, 129.3, 128.6, 127.5, 121.2, 119.6, 114.6, 111.7, 56.0, 47.7, 33.0.

HRMS (ESI, m/z): Mass calcd. for $C_{17}H_{15}NNaO_2S^+$ $[M+Na]^+$, 320.0716; found 320.0728.

HPLC analysis (Chiralcel IA; 25 °C, IPA/Hexane = 05/95, 0.6 mL/min, 254 nm), Rt_1 (major) = 44.3 min, Rt_2 (minor) = 51.2 min; 94:6 er.

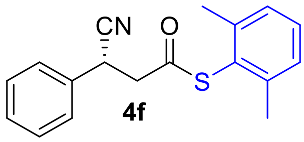
4f

***S*-(2,6-dimethylphenyl) (*R*)-3-cyano-3-phenylpropanethioate (4f)**

Purification by flash column chromatography on silica gel (petroleum ether / ethyl acetate = 6 / 1). Yellow solid, 78% yield, 23.0 mg, m.p. 88-90 °C.

[α]$^{25}_{D}$ = -28.2 (*c* = 0.8 in $CHCl_3$).

^{1}H NMR (400 MHz, $CDCl_3$) δ 7.46 – 7.36 (m, 5H), 7.29 – 7.24 (m, 1H), 7.17 (d, *J* = 7.5 Hz, 2H), 4.40 (t, *J* = 7.5 Hz, 1H), 3.36 (dd, *J* = 15.8, 7.5 Hz, 1H), 3.23 (dd, *J* = 15.8, 7.4 Hz, 1H), 2.27 (s, 6H).

^{13}C NMR (101 MHz, $CDCl_3$) δ 192.4, 142.8, 134.0, 130.4, 129.4, 128.7, 128.4, 127.6, 125.9, 119.6, 47.9, 33.2, 21.6.

HRMS (ESI, m/z): Mass calcd. for $C_{18}H_{17}NNaOS^+$ $[M+Na]^+$, 318.0923; found 318.0929.

HPLC analysis (Chiralcel IA; 25 °C, IPA/Hexane = 05/95, 0.6 mL/min, 254 nm), Rt_1 (major) = 42.7 min, Rt_2 (minor) = 50.1 min; 94:6 er.

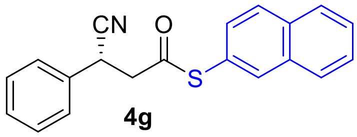
4g

***S*-(naphthalen-2-yl) (*R*)-3-cyano-3-phenylpropanethioate (4g)**

Purification by flash column chromatography on silica gel (petroleum ether / ethyl acetate = 15 / 1). White solid, 81% yield, 25.7 mg, m.p. 104-105 °C.

[α]$^{25}_{D}$ = -50.2 (*c* = 0.8 in $CHCl_3$).

^{1}H NMR (400 MHz, $CDCl_3$) δ 7.94 – 7.84 (m, 4H), 7.61 – 7.54 (m, 2H), 7.46 – 7.38 (m, 6H), 4.42 (dd, *J* = 7.8, 6.9 Hz, 1H), 3.41 (dd, *J* = 16.2, 7.9 Hz, 1H), 3.23 (dd, *J* = 16.2, 6.8 Hz, 1H).

^{13}C NMR (101 MHz, $CDCl_3$) δ 193.6, 134.6, 134.1, 133.5, 133.5, 130.6, 129.4, 129.1, 128.7, 128.1, 127.9, 127.5, 127.5, 126.8, 123.7, 119.6, 48.1, 33.1.

HRMS (ESI, m/z): Mass calcd. for $C_{20}H_{15}NNaOS^+$ $[M+Na]^+$, 340.0767; found 340.0768.

HPLC analysis (Chiralcel IA; 25 °C, IPA/Hexane = 05/95, 0.6 mL/min, 254 nm), Rt_1 (major) = 64.5 min, Rt_2 (minor) = 71.9 min; 94:6 er.

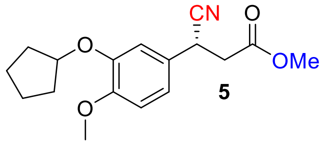

5

methyl (*R*)-3-cyano-3-(3-(cyclopentyloxy)-4- methoxyphenyl) propanoate (5)

Purification by flash column chromatography on silica gel (petroleum ether / ethyl acetate = 10 / 1). White solid, 91% yield, 2.51 g, m.p. 98-99 ºC.

^{1}H NMR (400 MHz, CDCl$_3$) δ 6.90 – 6.81 (m, 3H), 4.80 – 4.76 (m, 1H), 4.23 (dd, *J* = 8.0, 6.9 Hz, 1H), 3.83 (s, 3H), 3.71 (s, 3H), 3.00 (dd, *J* = 16.5, 8.1 Hz, 1H), 2.82 (dd, *J* = 16.5, 6.8 Hz, 1H), 1.98 – 1.80 (m, 6H), 1.67 – 1.56 (m, 2H).

^{13}C NMR (101 MHz, CDCl$_3$) δ 169.8, 150.1, 148.1, 126.6, 120.2, 119.5, 113.8, 112.2, 80.6, 56.1, 52.3, 40.0, 32.8, 32.8, 32.7, 24.1.

HRMS (ESI, m/z): Mass calcd. for $C_{17}H_{21}NNaO_4^+$ [M+Na]$^+$, 326.1363; found 326.1364.

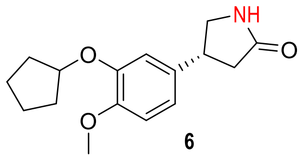

6

(*R*)-Rolipram (6)

Purification by flash column chromatography on silica gel (Dichloromethane / Methanol = 20 / 1). white solid, 86% yield, 2.2 g, m.p. 133-134 ºC.

$[\alpha]^{25}_{D}$ = -30.7 (*c* = 0.6 in CHCl$_3$).

^{1}H NMR (400 MHz, CDCl$_3$) δ 6.84 – 6.75 (m, 3H), 6.63 (s, 1H), 4.79 – 4.75 (m, 1H), 3.83 (s, 3H), 3.76 (t, *J* = 8.8 Hz, 1H), 3.67 – 3.59 (m, 1H), 3.39 (dd, *J* = 9.5, 7.3 Hz, 1H), 2.71 (dd, *J* = 16.9, 8.9 Hz, 1H), 2.47 (dd, *J* = 16.9, 8.9 Hz, 1H), 1.95 – 1.81 (m, 6H), 1.67 – 1.56 (m, 2H).

^{13}C NMR (101 MHz, CDCl$_3$) δ 177.9, 149.2, 147.9, 134.6, 118.8, 113.9, 112.2, 80.6, 56.2, 49.8, 40.0, 38.2, 32.8, 24.0.

HRMS (ESI, m/z): Mass calcd. for $C_{16}H_{21}NNaO_3^+$ [M+Na]$^+$, 298.1414; found 298.1415.

HPLC analysis (Chiralcel IA; 25 ºC, IPA/Hexane = 05/95, 0.5 mL/min, 254 nm), Rt$_1$ (major) = 18.1 min, Rt$_2$ (minor) = 22.5 min; 95:5 er.

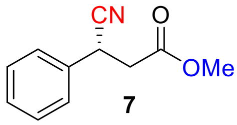

7

methyl (*R*)-3-cyano-3-phenylpropanoate (7)

Purification by flash column chromatography on silica gel (petroleum ether / ethyl acetate = 5 / 1). White solid, 87% yield, 56.9 mg, m.p. 92-94 ºC.

$[\alpha]^{25}_{D}$ = 15.6 (*c* = 0.6 in CHCl$_3$).

^{1}H NMR (400 MHz, CDCl$_3$) δ 7.40 – 7.32 (m, 5H), 4.29 (dd, *J* = 8.4, 6.5 Hz, 1H), 3.69 (s, 3H), 3.00 (dd, *J* = 16.7, 8.4 Hz, 1H), 2.84 (dd, *J* = 16.7, 6.6 Hz, 1H).

^{13}C NMR (101 MHz, CDCl$_3$) δ 169.7, 134.5, 129.3, 128.6, 127.4, 120.0, 52.3, 39.7, 33.1.

HRMS (ESI, m/z): Mass calcd. for $C_{11}H_{11}NNaO_2^+$ [M+Na]$^+$, 212.0682; found 212.0685.

HPLC analysis (Chiralcel AD-H; 25 ºC, IPA/Hexane = 05/95, 0.5 mL/min, 254 nm), Rt$_1$ (major) = 33.8 min, Rt$_2$ (minor) = 37.2 min; 95:5 er.

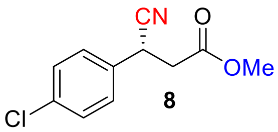

8

methyl (*R*)-3-(4-chlorophenyl)-3-cyanopropanoate (8)

Purification by flash column chromatography on silica gel (petroleum ether / ethyl acetate = 5 / 1). Yellow solid, 90% yield, 77.8 mg, m.p. 93-95 °C.

$[\alpha]^{25}_{D}$ = 10.2 (c = 0.6 in $CHCl_3$).

^{1}H NMR (400 MHz, $CDCl_3$) δ 7.41 – 7.35 (m, 2H), 7.34 – 7.29 (m, 2H), 4.28 (t, J = 7.4 Hz, 1H), 3.72 (s, 3H), 3.02 (dd, J = 16.7, 7.8 Hz, 1H), 2.83 (dd, J = 16.7, 7.1 Hz, 1H).

^{13}C NMR (101 MHz, $CDCl_3$) δ 169.4, 134.8, 132.9, 129.5, 128.8, 119.5, 52.5, 39.7, 32.6.

HRMS (ESI, m/z): Mass calcd. for $C_{11}H_{10}ClNNaO_2^+$ $[M+Na]^+$, 246.0292; found 246.0288.

HPLC analysis (Chiralcel AD-H; 25 °C, IPA/Hexane = 05/95, 0.5 mL/min, 254 nm), Rt_1 (major) = 33.9 min, Rt_2 (minor) = 40.1 min; 87:13 er.

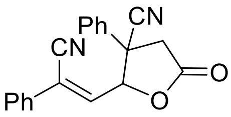

3a'

(*Z*)-2-(2-cyano-2-phenylvinyl)-5-oxo-3-phenyltetrahydrofuran -3-carbonitrile (3a')

Purification by flash column chromatography on silica gel (petroleum ether / ethyl acetate = 5 / 1). Yellow solid, 88% yield, 27.7 mg, m.p. 107-109 °C.

^{1}H NMR (400 MHz, $CDCl_3$) δ 7.62 – 7.43 (m, 10H), 6.96 (d, J = 9.2 Hz, 1H), 5.58 (d, J = 9.2 Hz, 1H), 3.45 (s, 2H).

^{13}C NMR (101 MHz, $CDCl_3$) δ 170.1, 133.6, 131.2, 131.0, 130.9, 130.2, 130.1, 129.4, 126.6, 126.0, 123.2, 117.8, 114.3, 84.0, 51.6, 41.3.

HRMS (ESI, m/z): Mass calcd. for $C_{20}H_{14}N_2NaO_2^+$ $[M+Na]^+$, 337.0947; found 337.0948.

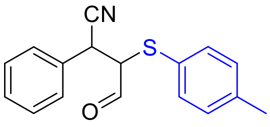

I

4-oxo-2-phenyl-3-(*p*-tolylthio)butanenitrile (I)

Purification by flash column chromatography on silica gel (petroleum ether / ethyl acetate = 5 / 1). Yellow oil, 36% yield, 10.1 mg. 2:1 dr (reaction mixture)

^{1}H NMR (400 MHz, $CDCl_3$) δ 9.66 (d, J = 1.5 Hz, 0.3H, minor), δ 9.41 (d, J = 1.5 Hz, 0.7H, major), 7.40 – 7.31 (m, 6H), 7.18 – 7.08 (m, 3H), 4.18 (t, J = 9.0 Hz, 1H), 3.95 (dd, J = 8.13, 1.5 Hz, 0.3H, minor), 3.89 (dd, J = 9.8, 1.5 Hz, 0.7H, major), 2.36 (s, 2H, major), 2.32 (s, 1H, minor).

^{13}C NMR (101 MHz, $CDCl_3$) δ 188.4, 140.6, 136.0, 133.0, 130.4, 129.2, 128.9, 128.6, 124.5, 119.0, 59.8, 36.9, 21.3.

HRMS (ESI, m/z): Mass calcd. for $C_{17}H_{15}NNaOS^+$ $[M+Na]^+$, 304.0767; found 304.0762.

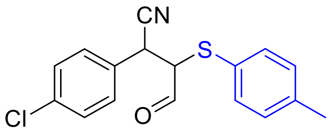

I'

2-(4-chlorophenyl)-4-oxo-3-(*p*-tolylthio)butanenitrile (I')

Purification by flash column chromatography on silica gel (petroleum ether / ethyl acetate = 5 / 1). Yellow soild, 42% yield, 18.2 mg. m.p. 110-111 °C. 2:1 dr (reaction mixture)

^{1}H NMR (400 MHz, $CDCl_3$) δ 9.66 (d, J = 1.3 Hz, 0.3H, minor), δ 9.42 (d, J = 1.3 Hz, 0.7H, major), 7.42 – 7.35 (m, 2H), 7.32 – 7.27 (m, 3H), 7.18 – 7.16 (m, 2H), 7.12 – 7.10 (m, 1H), 4.16 (dd, J = 9.0, 3.9

Hz, 1H), 3.95 (dd, *J* = 8.13, 1.3 Hz, 0.3H, minor), 3.86 (dd, *J* = 9.8, 1.3 Hz, 0.7H, major), 2.36 (s, 2H, major), 2.33 (s, 1H, minor).

^{13}C NMR (101 MHz, $CDCl_3$) δ 188.3, 140.8, 136.1, 134.6, 131.5, 130.5, 130.0, 129.4, 124.1, 118.7, 59.6, 36.3, 21.3.

HRMS (ESI, m/z): Mass calcd. for $C_{17}H_{14}ClNNaOS^+$ $[M+Na]^+$, 338.0377; found 338.0371.

V. Supplementary Figures

^{1}H NMR, ^{13}C NMR and ^{19}F NMR spectra

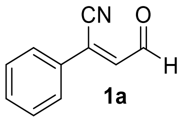

1a

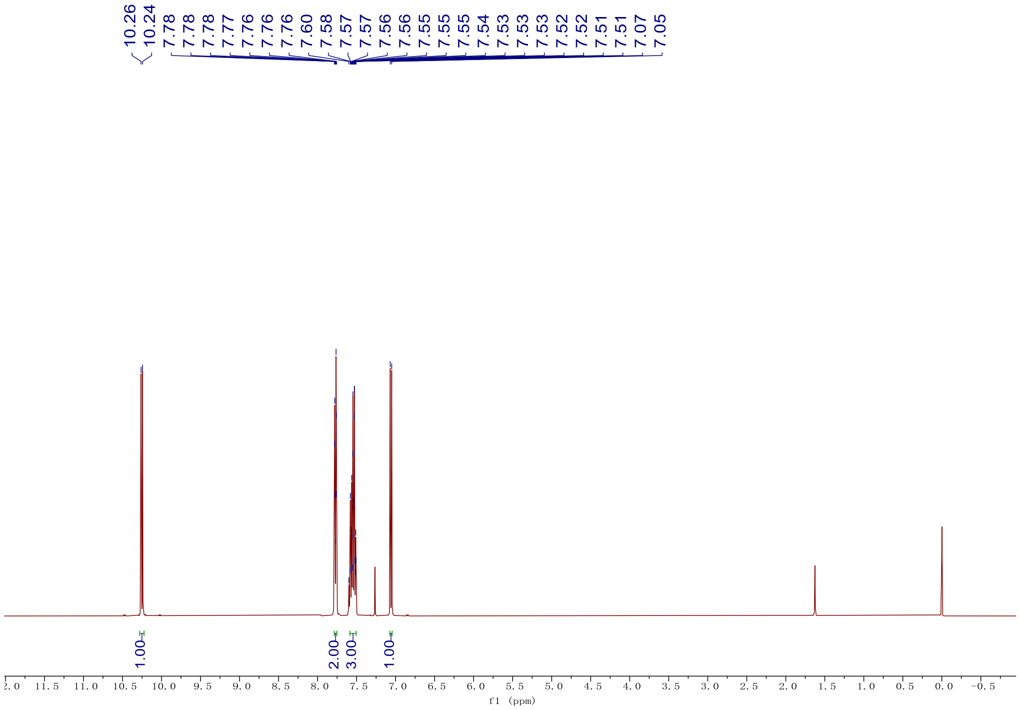

Supplementary Figure 4. ^{1}H NMR (400 MHz, $CDCl_3$)

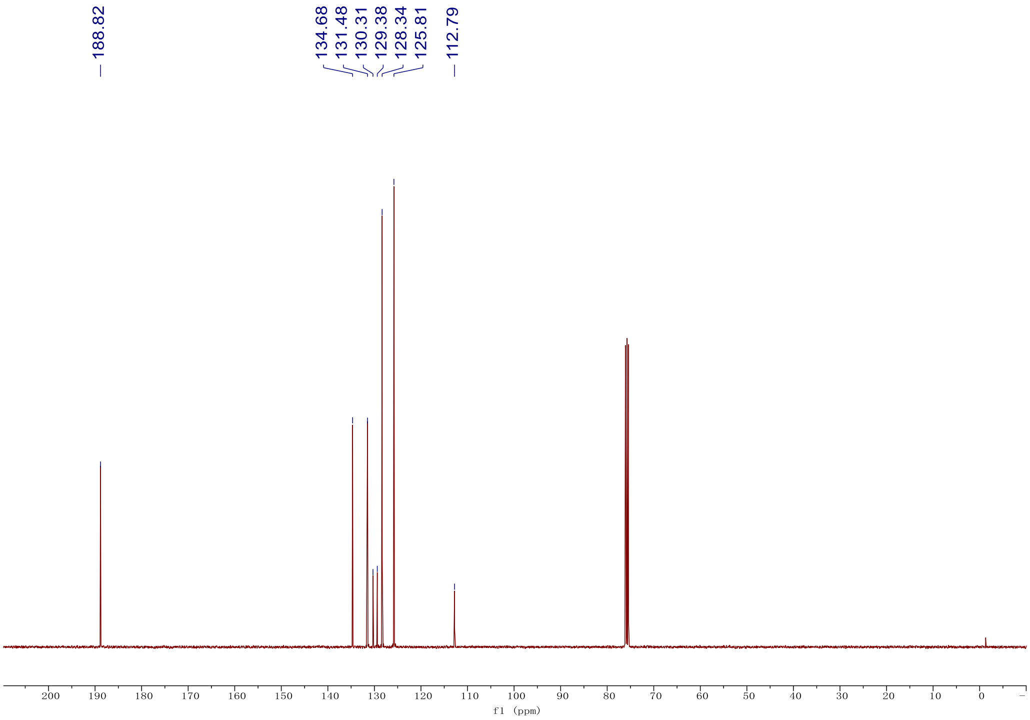

Supplementary Figure 5. ^{13}C NMR (101 MHz, $CDCl_3$)

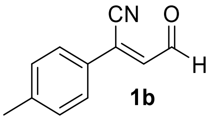

1b

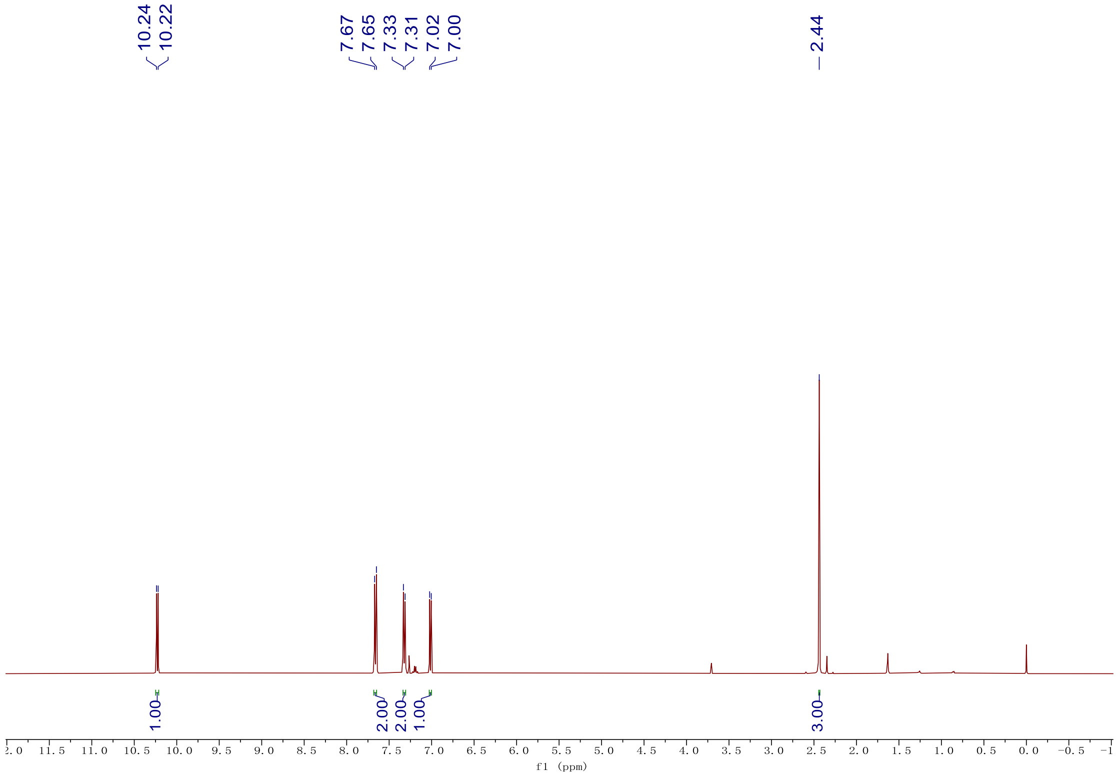

<u>Supplementary Figure 6. ^{1}H NMR (400 MHz, $CDCl_3$)</u>

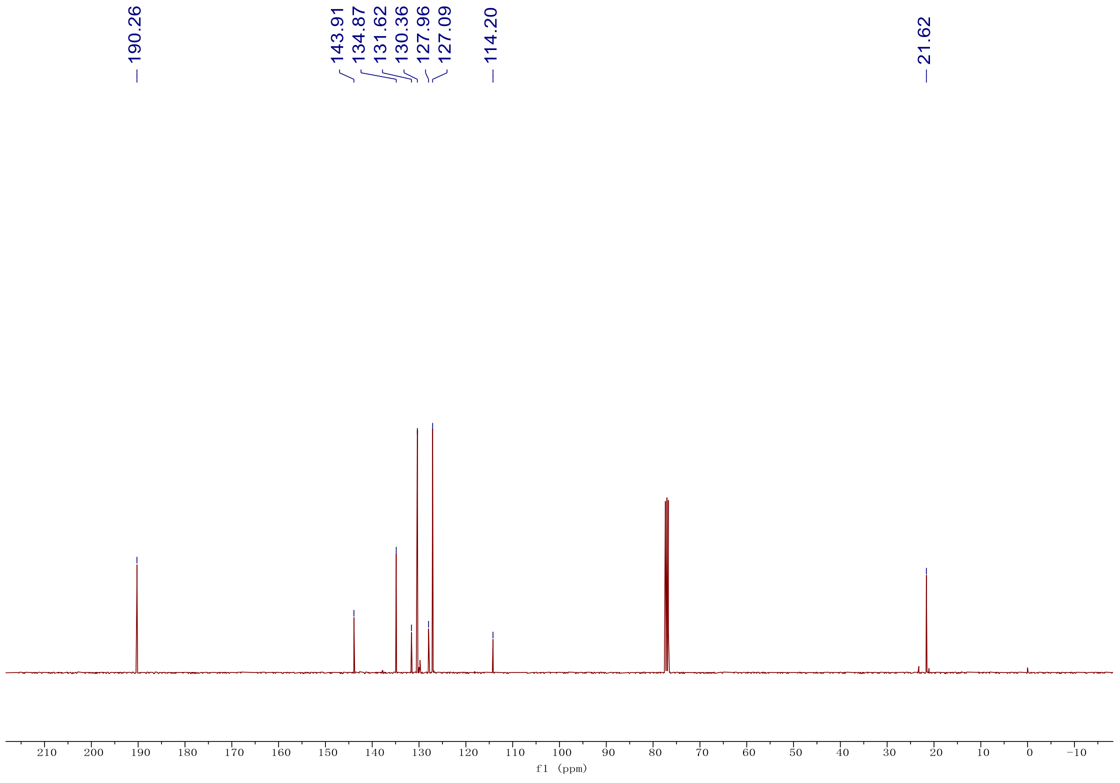

<u>Supplementary Figure 7. ^{13}C NMR (101 MHz, $CDCl_3$)</u>

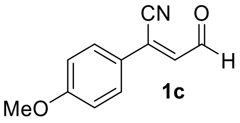

1c

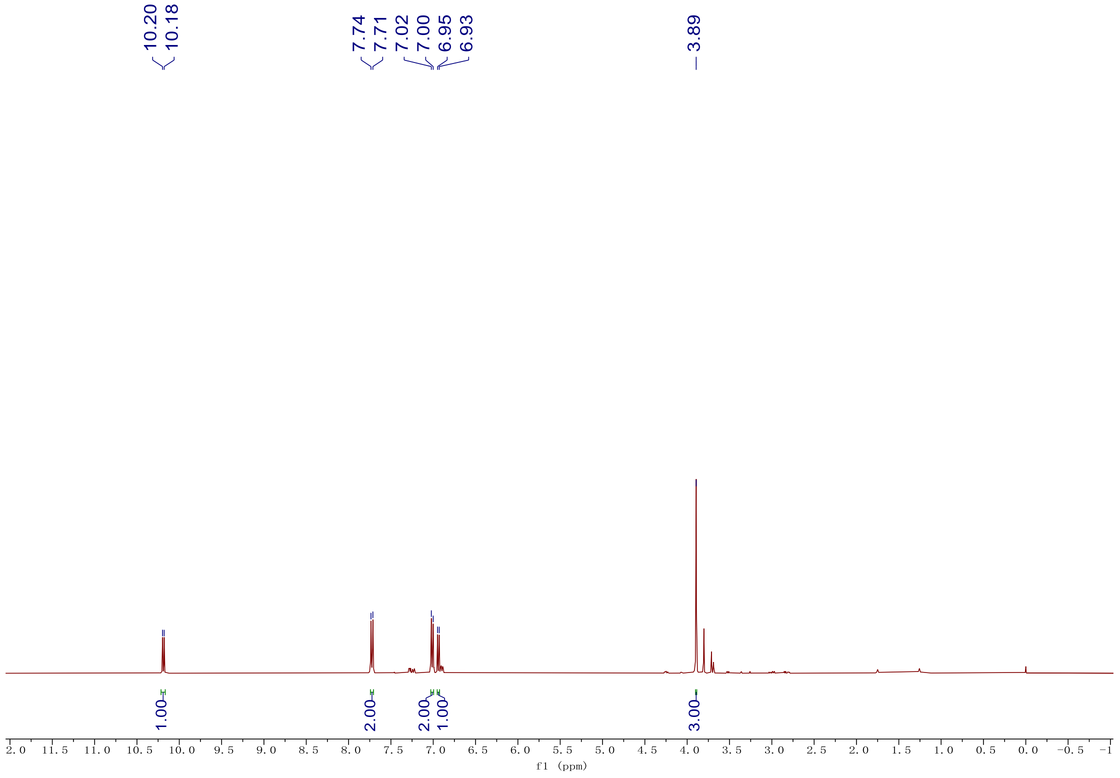

Supplementary Figure 8. ^{1}H NMR (400 MHz, CDCl$_3$)

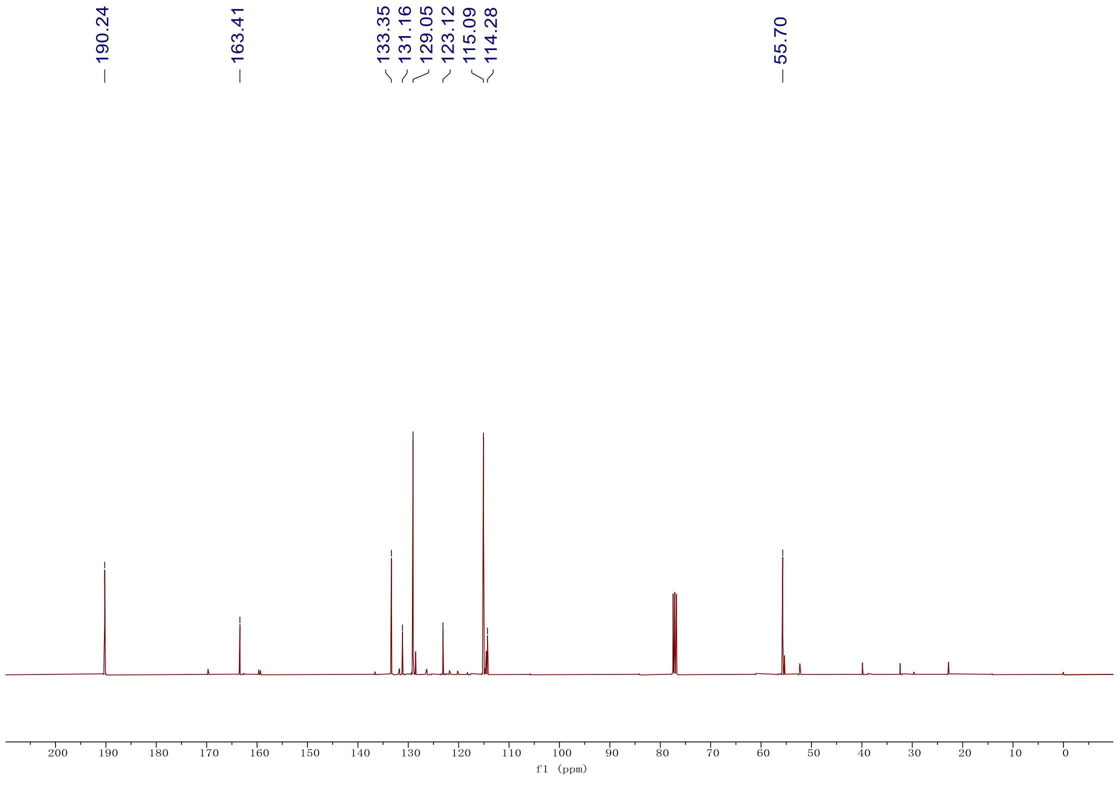

Supplementary Figure 9. ^{13}C NMR (101 MHz, CDCl$_3$)

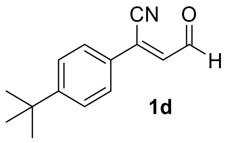

1d

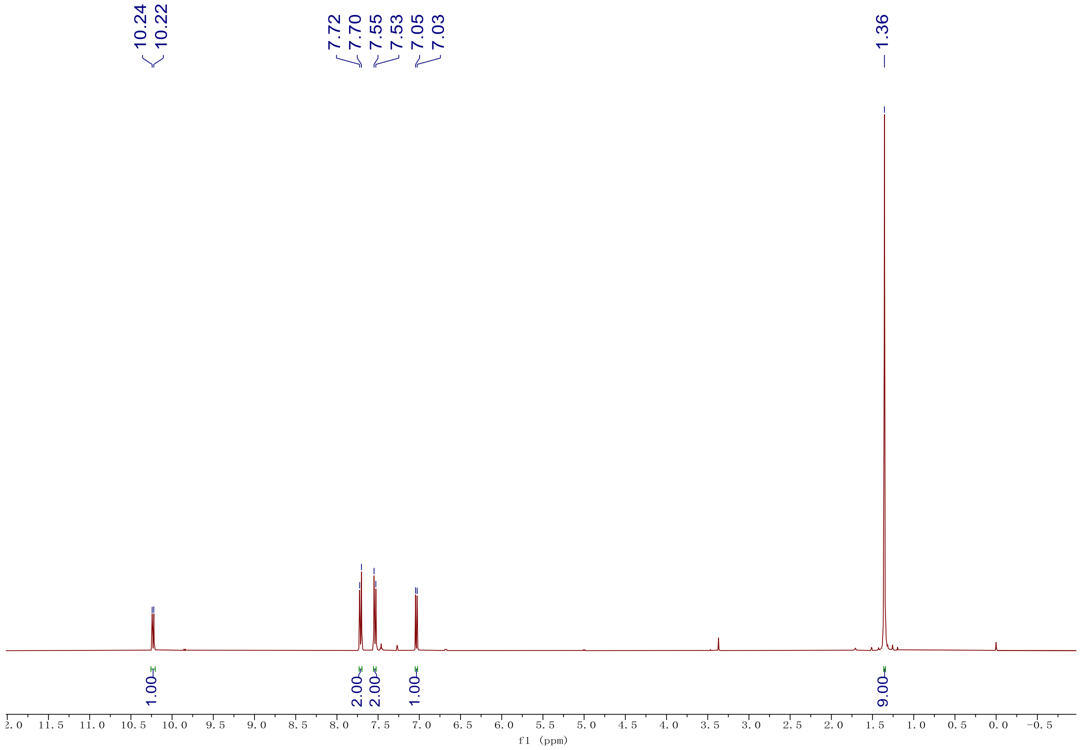

Supplementary Figure 10. ^{1}H NMR (400 MHz, CDCl$_3$)

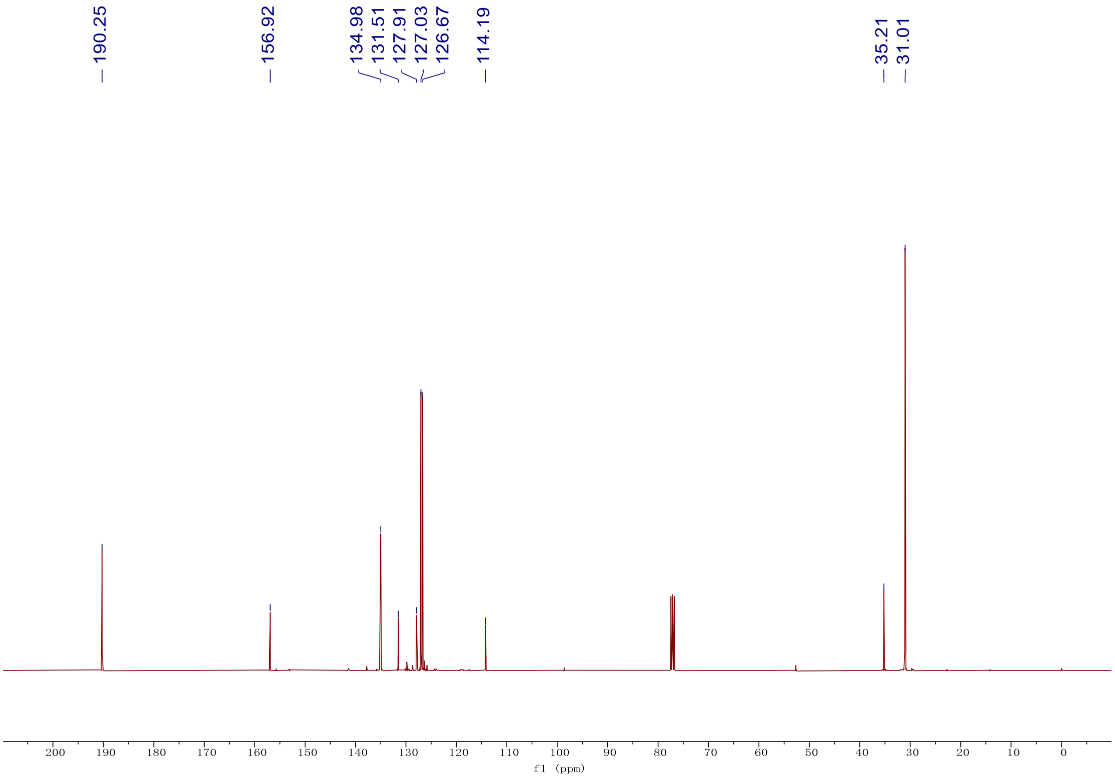

Supplementary Figure 11. ^{13}C NMR (101 MHz, CDCl$_3$)

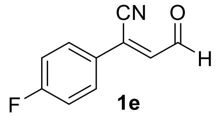

1e

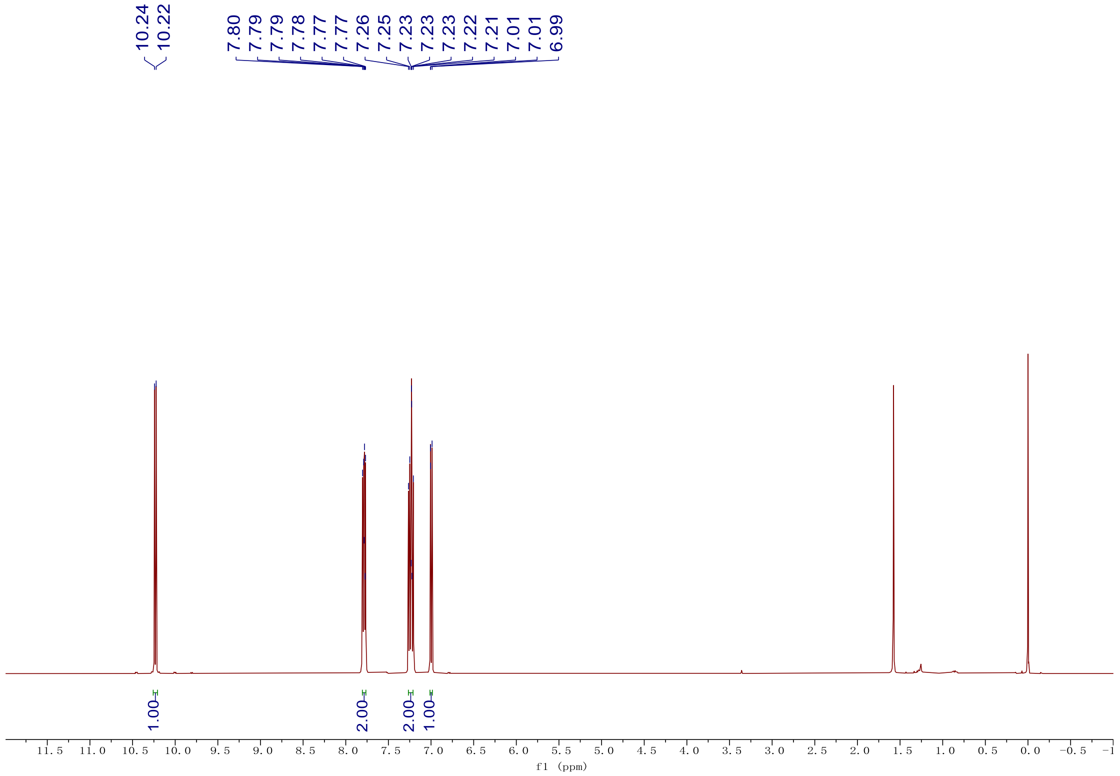

Supplementary Figure 12. ^{1}H NMR (400 MHz, $CDCl_3$)

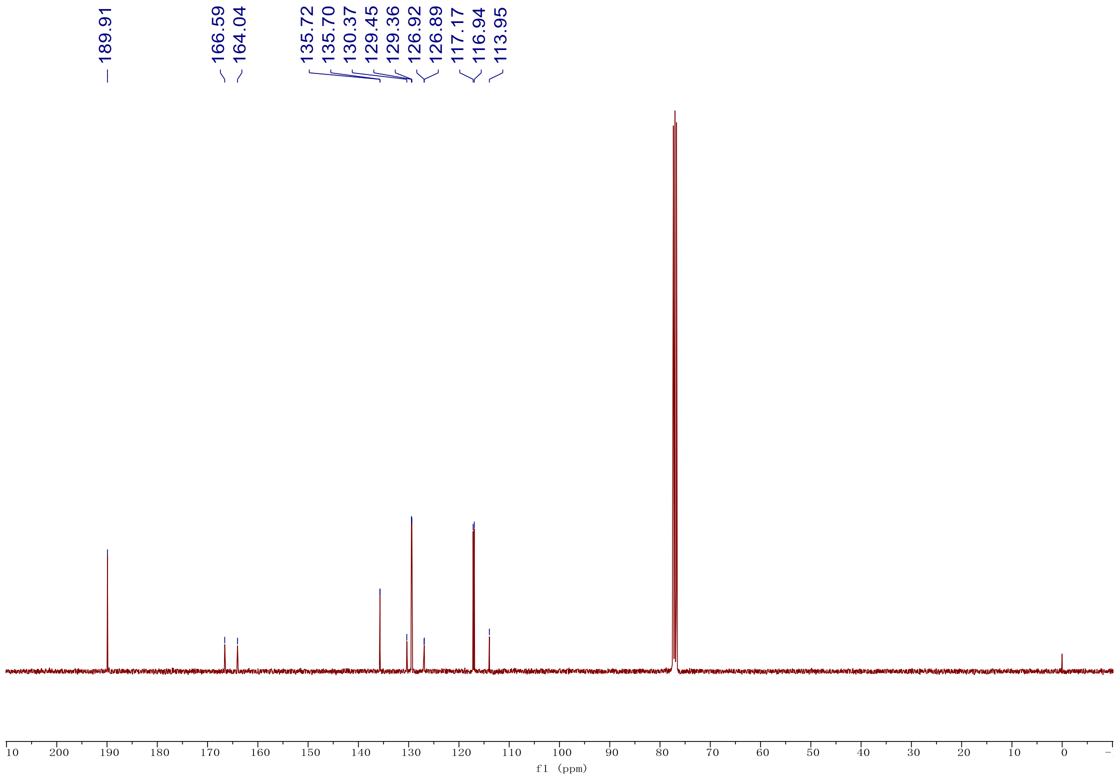

Supplementary Figure 13. ^{13}C NMR (101 MHz, $CDCl_3$)

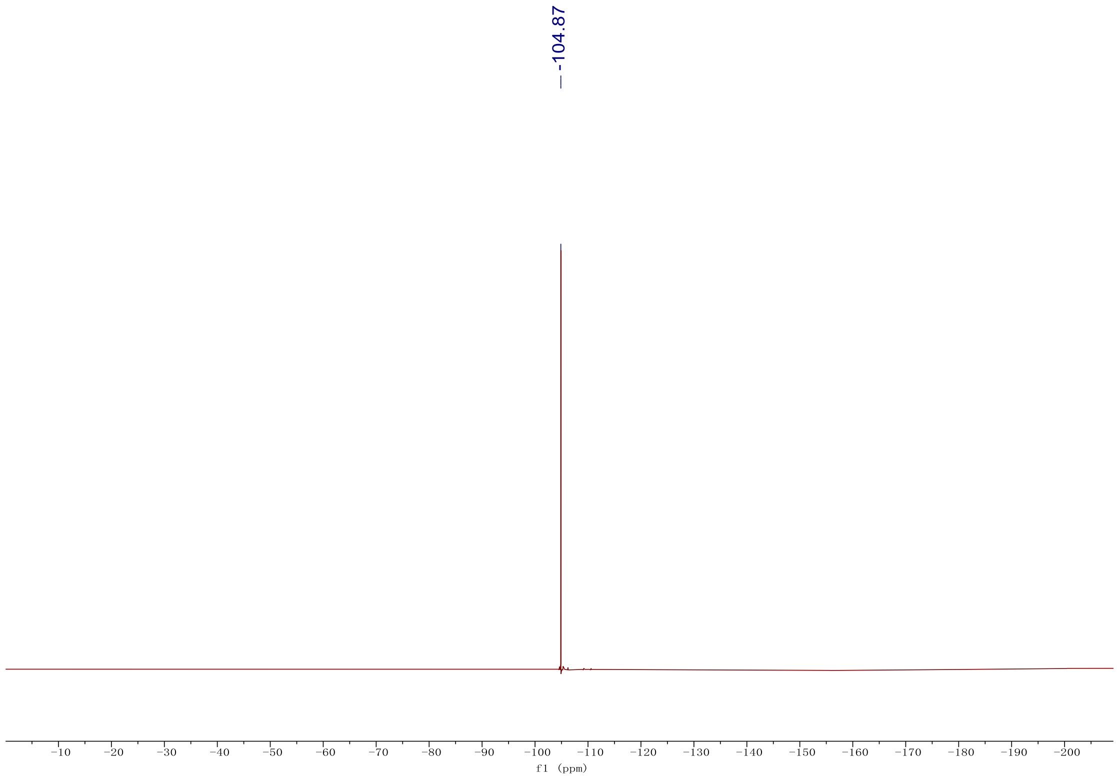

Supplementary Figure 14. ^{19}F NMR (376 MHz, $CDCl_3$)

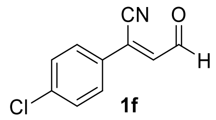

1f

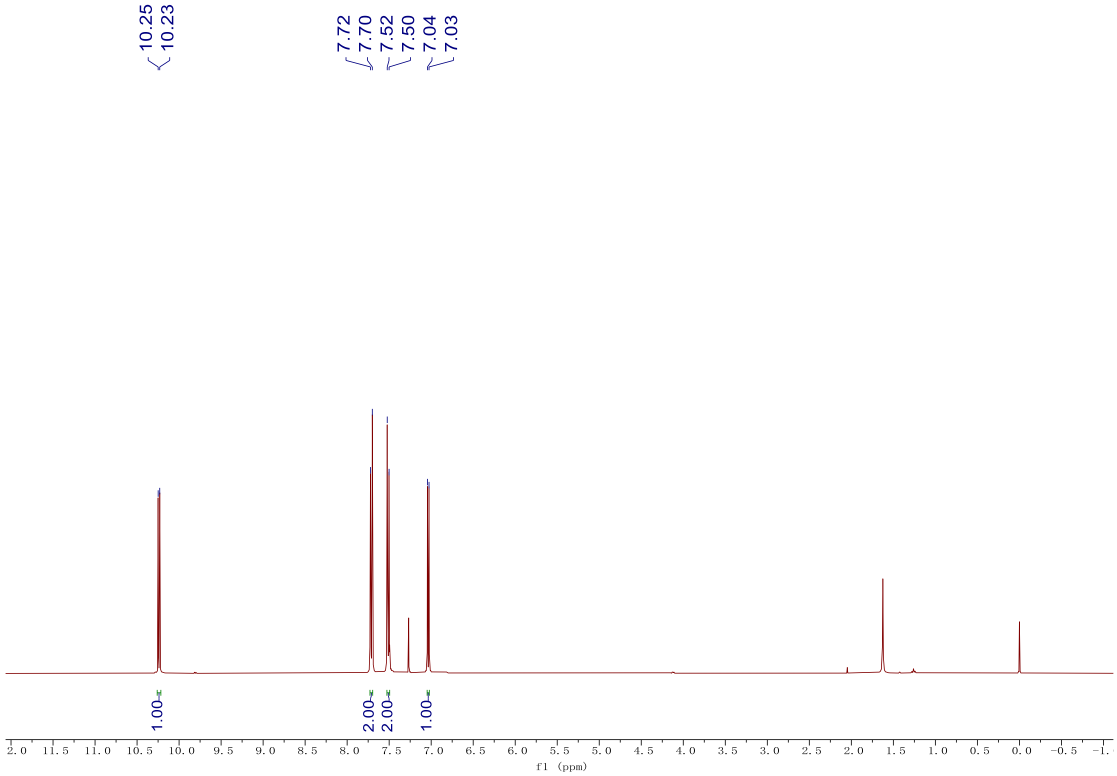

Supplementary Figure 15. ^{1}H NMR (400 MHz, CDCl$_3$)

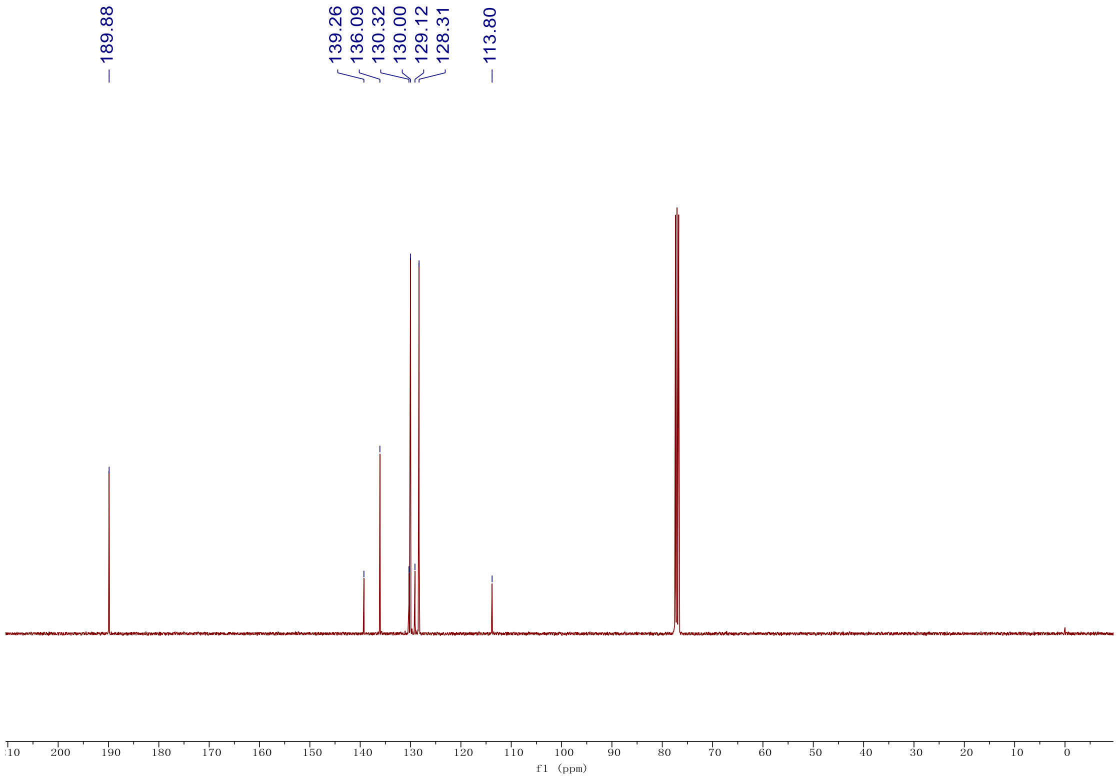

Supplementary Figure 16. ^{13}C NMR (101 MHz, CDCl$_3$)

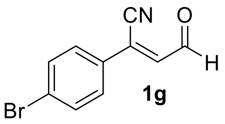

1g

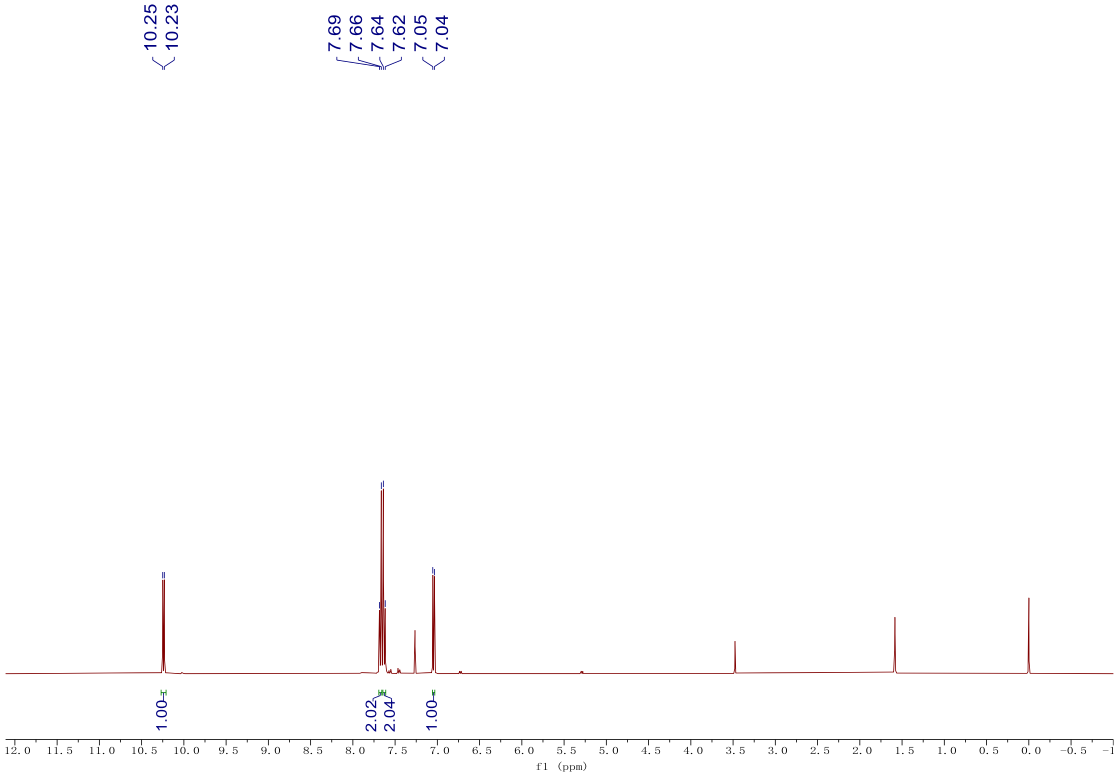

Supplementary Figure 17. ^{1}H NMR (400 MHz, $CDCl_3$)

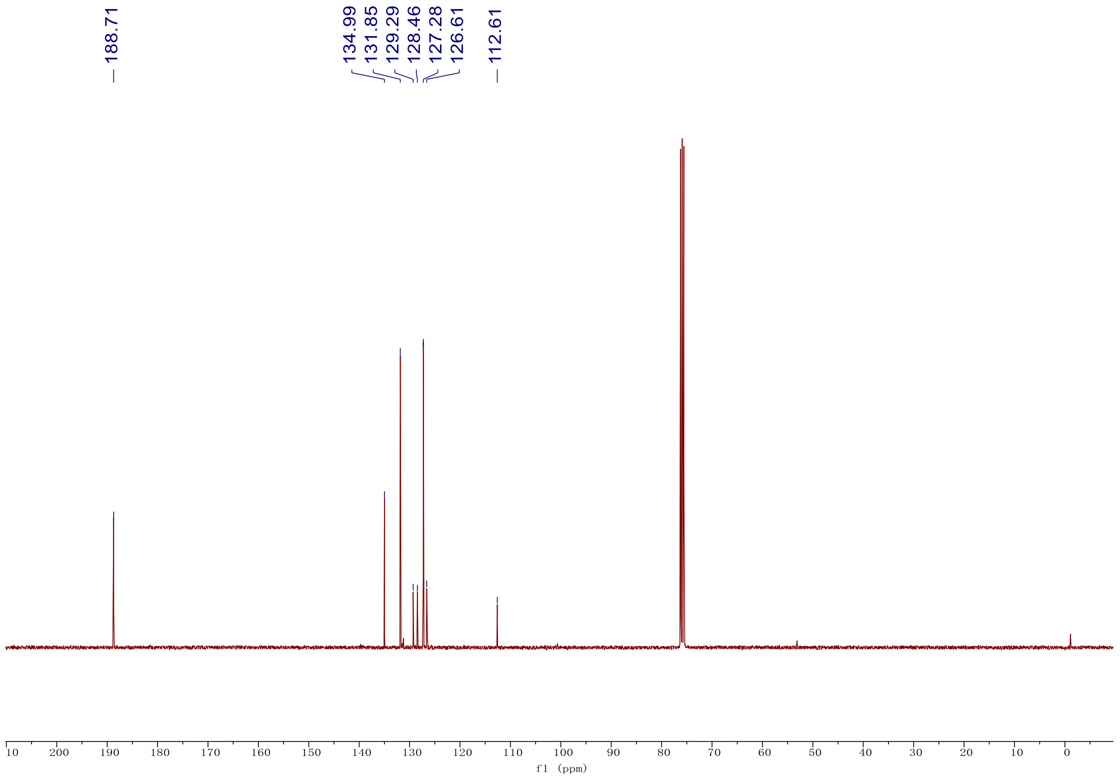

Supplementary Figure 18. ^{13}C NMR (101 MHz, $CDCl_3$)

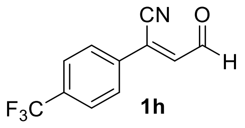

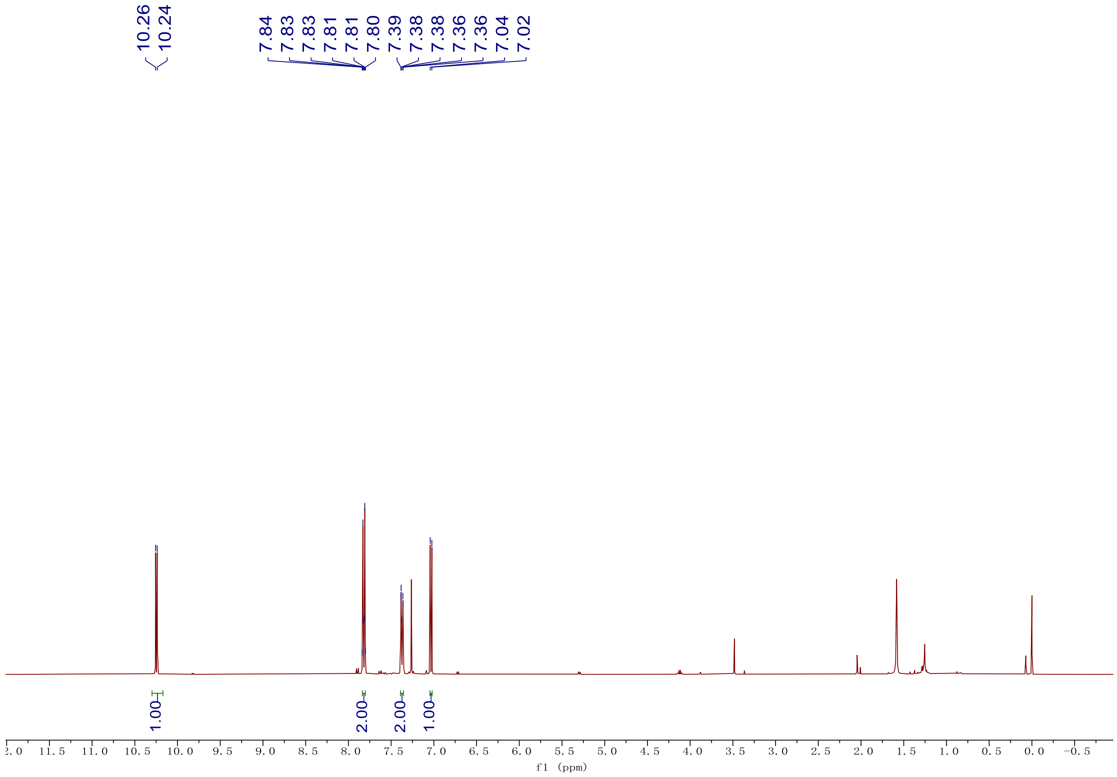

Supplementary Figure 19. ^{1}H NMR (400 MHz, CDCl$_3$)

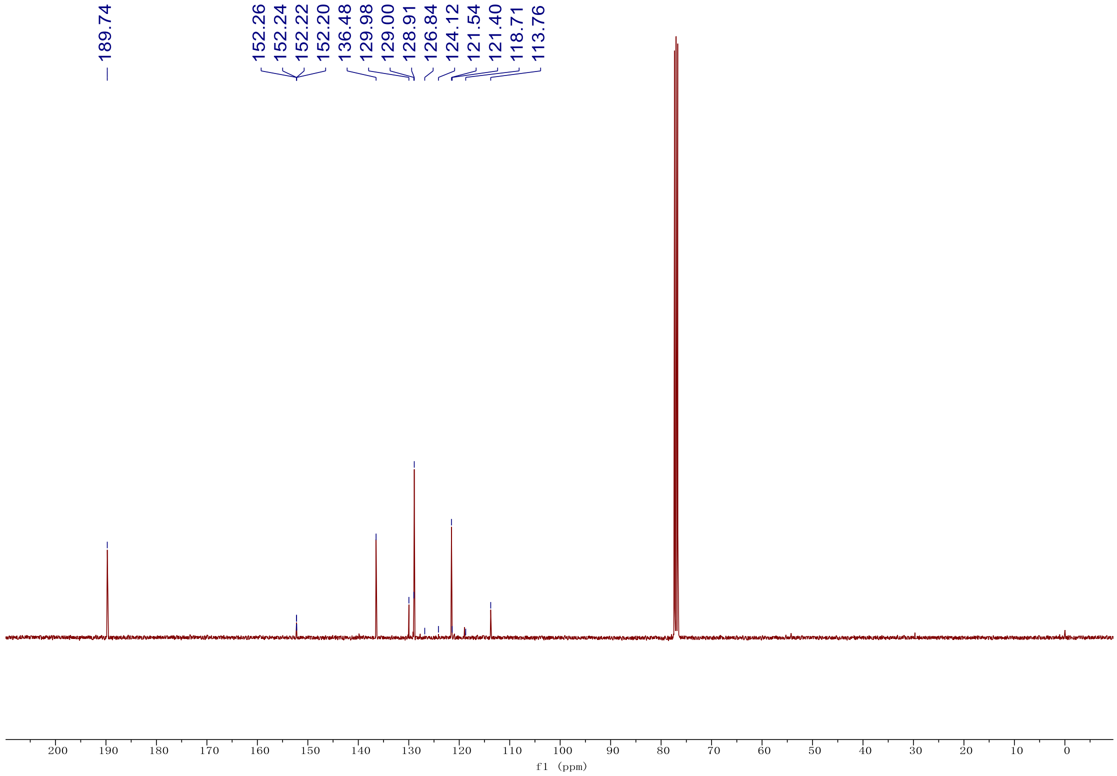

Supplementary Figure 20. ^{13}C NMR (101 MHz, CDCl$_3$)

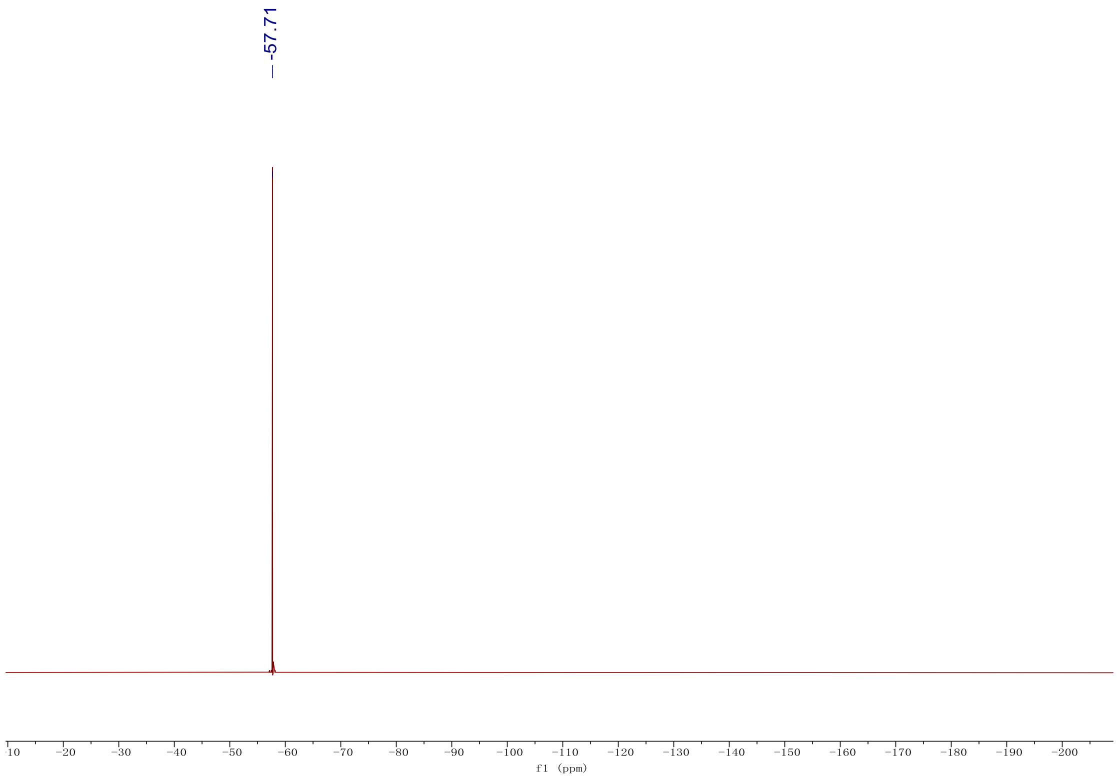

Supplementary Figure 21. ^{19}F NMR (376 MHz, $CDCl_3$)

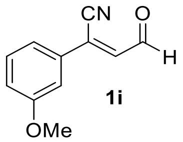

1i

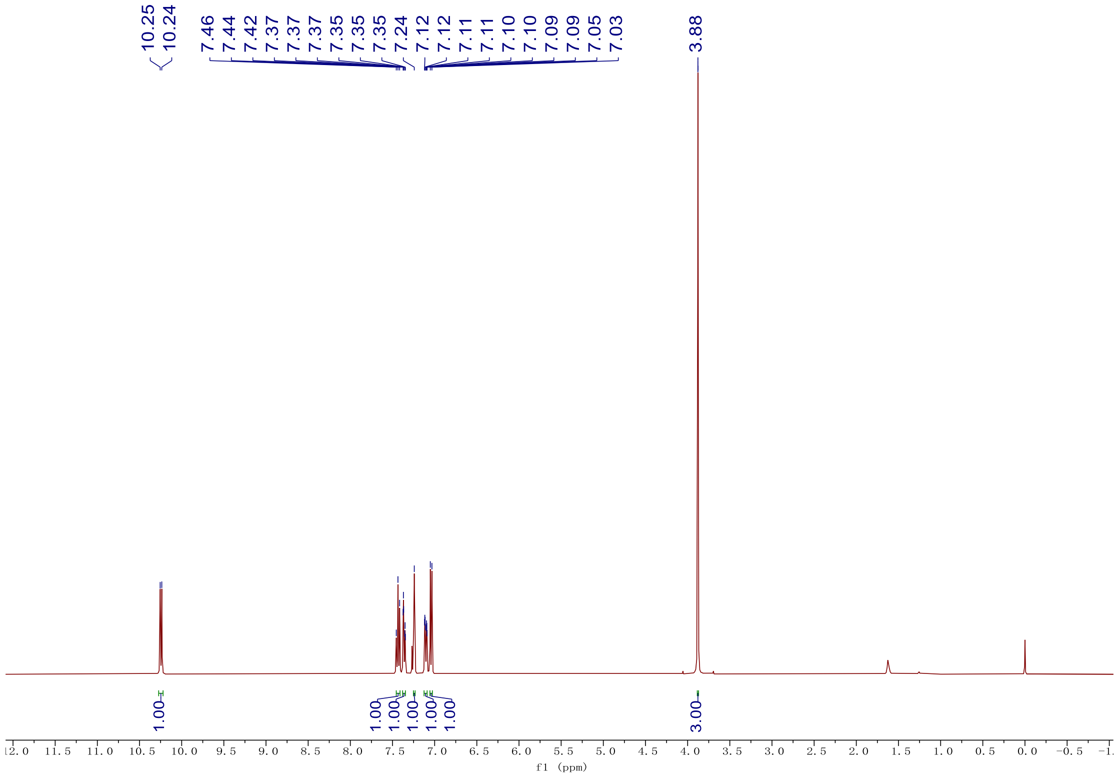

Supplementary Figure 22. ^{1}H NMR (400 MHz, $CDCl_3$)

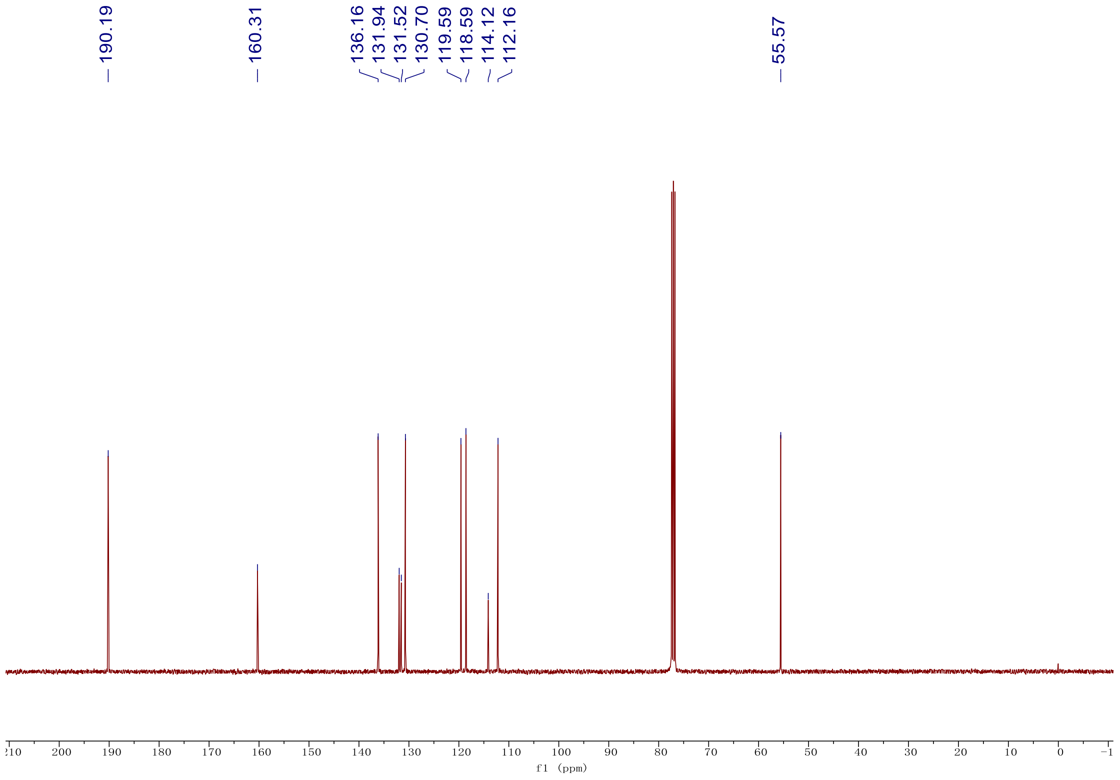

Supplementary Figure 23. ^{13}C NMR (101 MHz, $CDCl_3$)

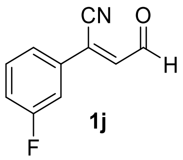

1j

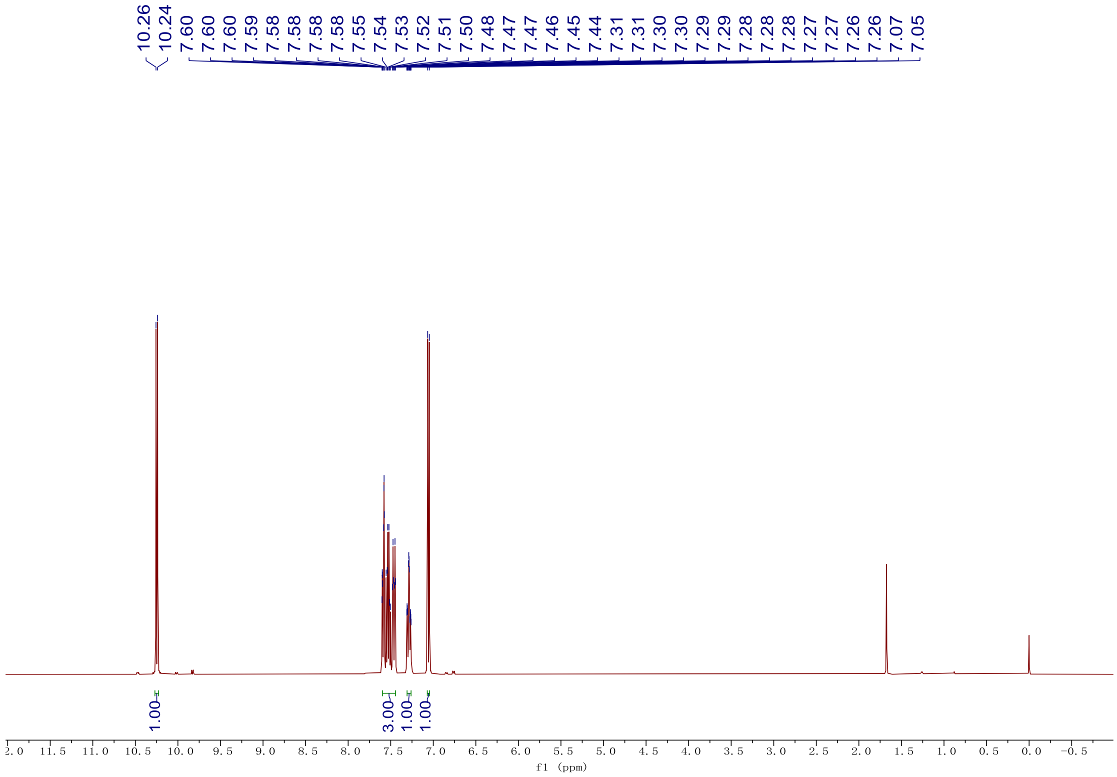

Supplementary Figure 24. ^{1}H NMR (400 MHz, CDCl$_3$)

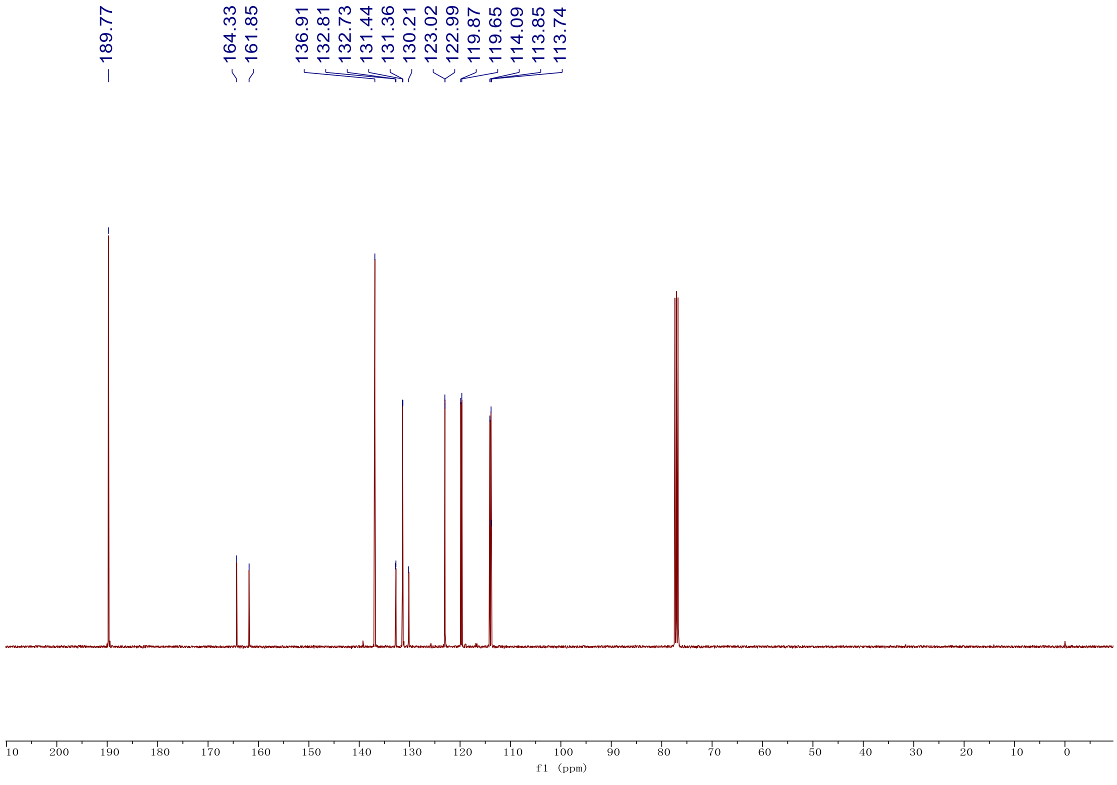

Supplementary Figure 25. ^{13}C NMR (101 MHz, CDCl$_3$)

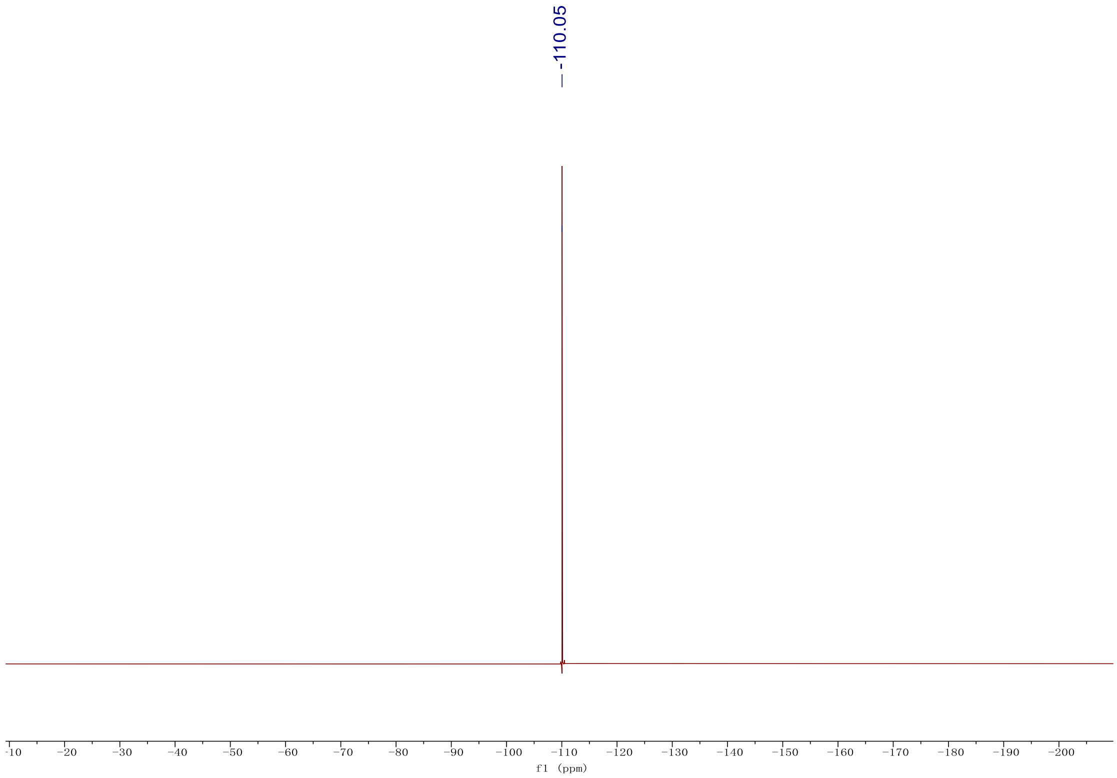

Supplementary Figure 26. ^{19}F NMR (376 MHz, $CDCl_3$)

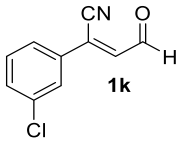

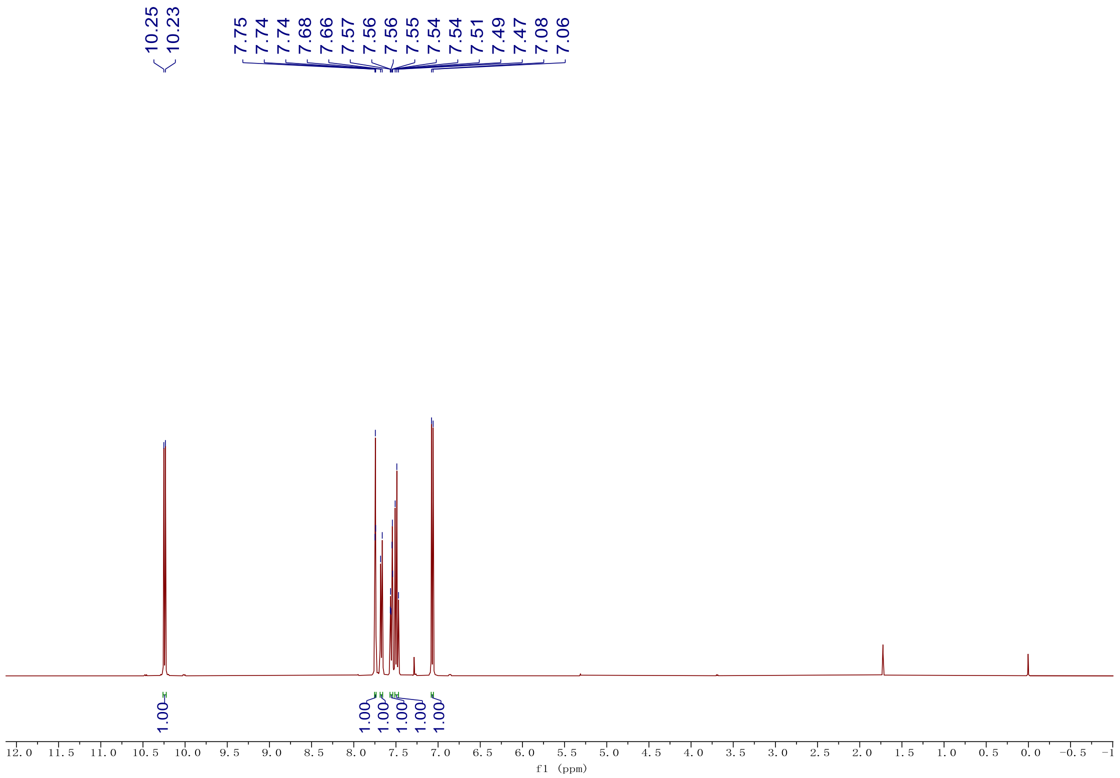

Supplementary Figure 27. ^{1}H NMR (400 MHz, $CDCl_3$)

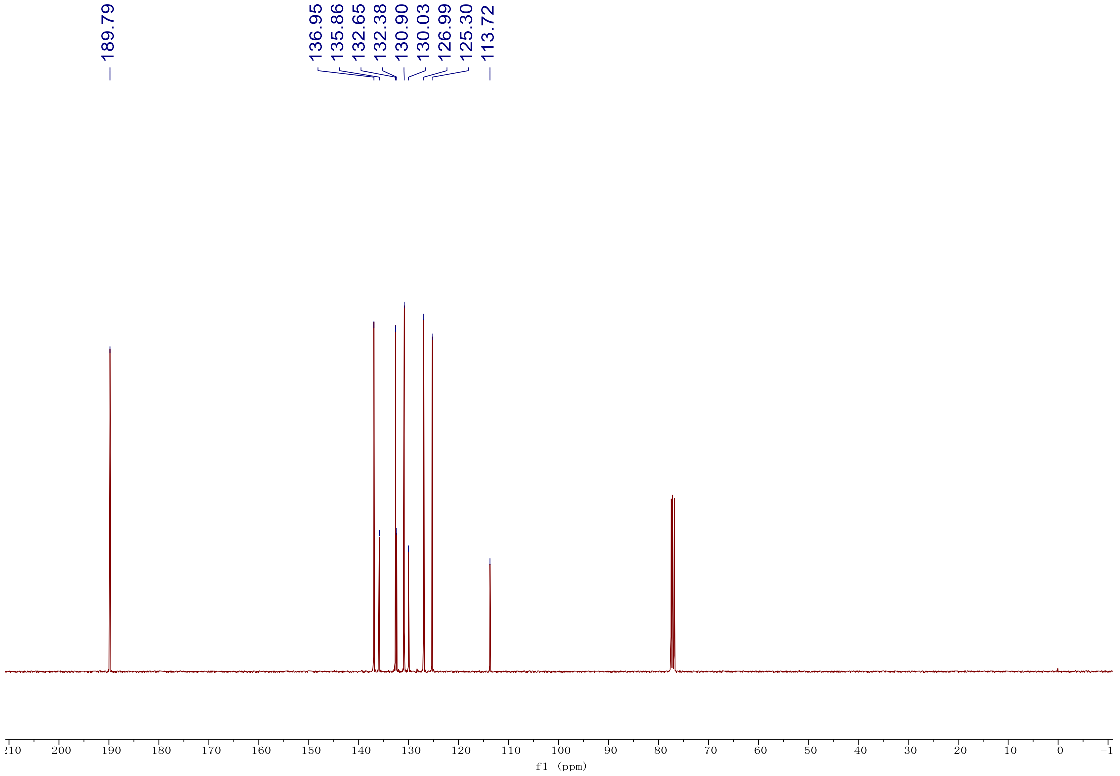

Supplementary Figure 28. ^{13}C NMR (101 MHz, $CDCl_3$)

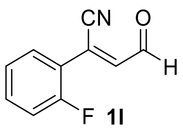

1l

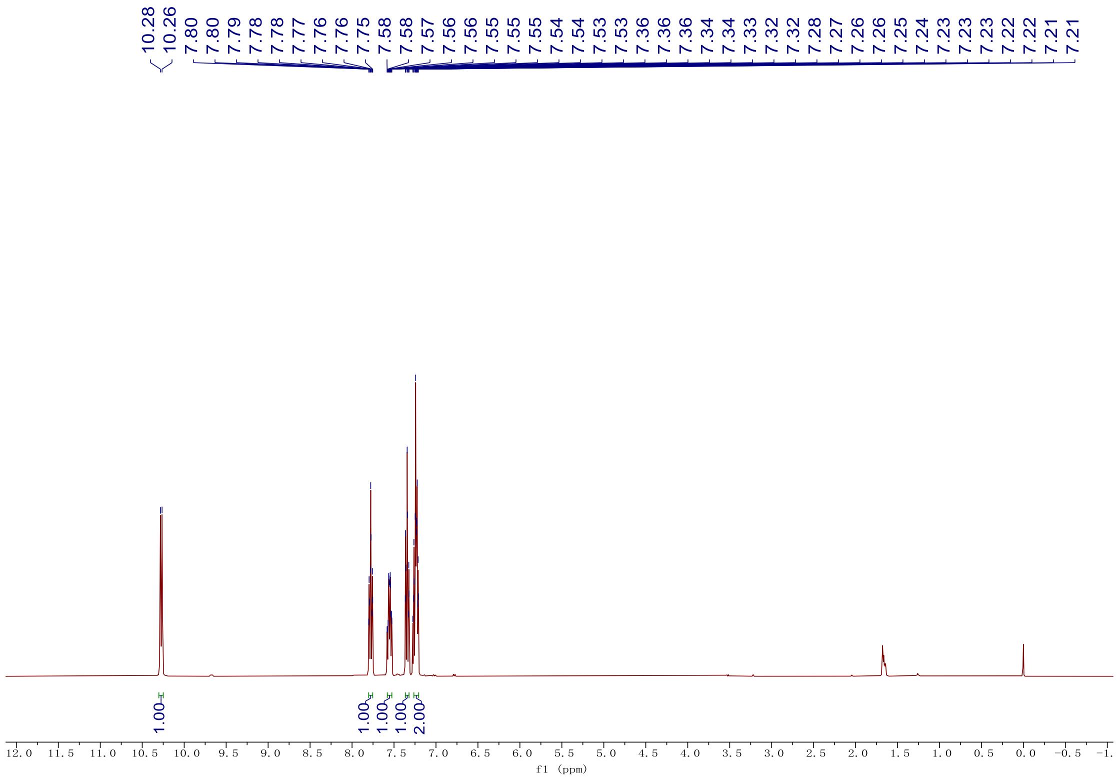

Supplementary Figure 29. ^{1}H NMR (400 MHz, $CDCl_3$)

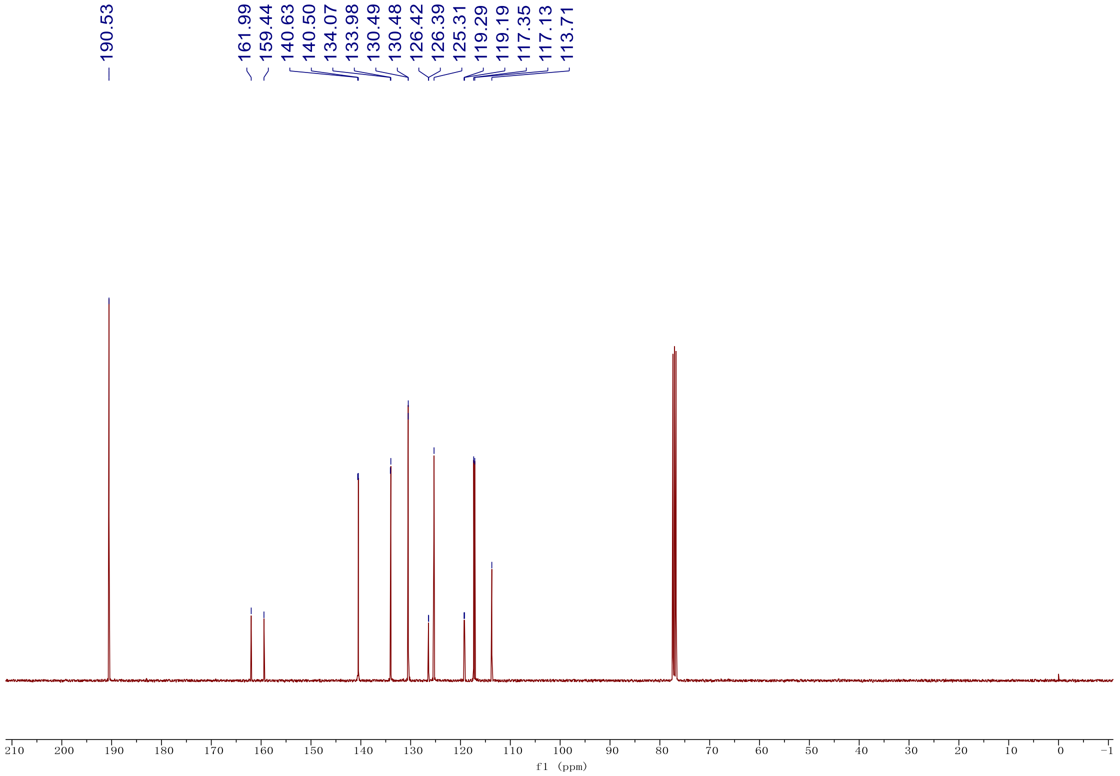

Supplementary Figure 30. ^{13}C NMR (101 MHz, $CDCl_3$)

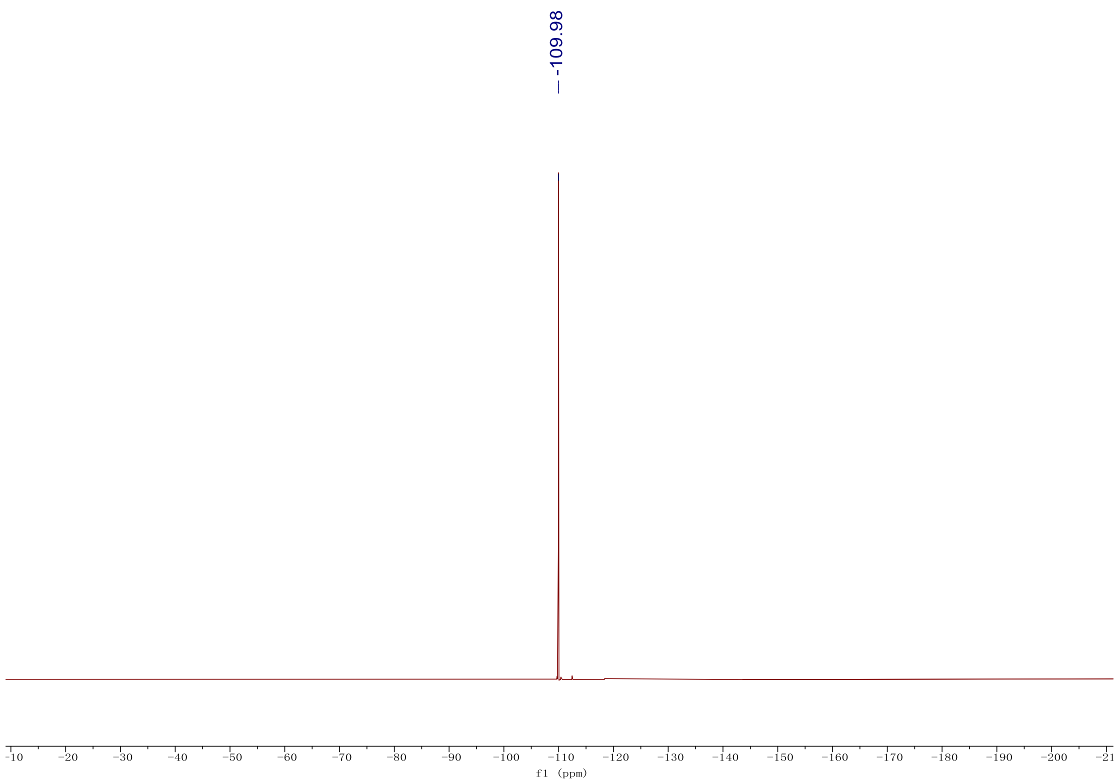

Supplementary Figure 31. ^{19}F NMR (376 MHz, $CDCl_3$)

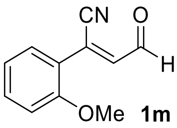

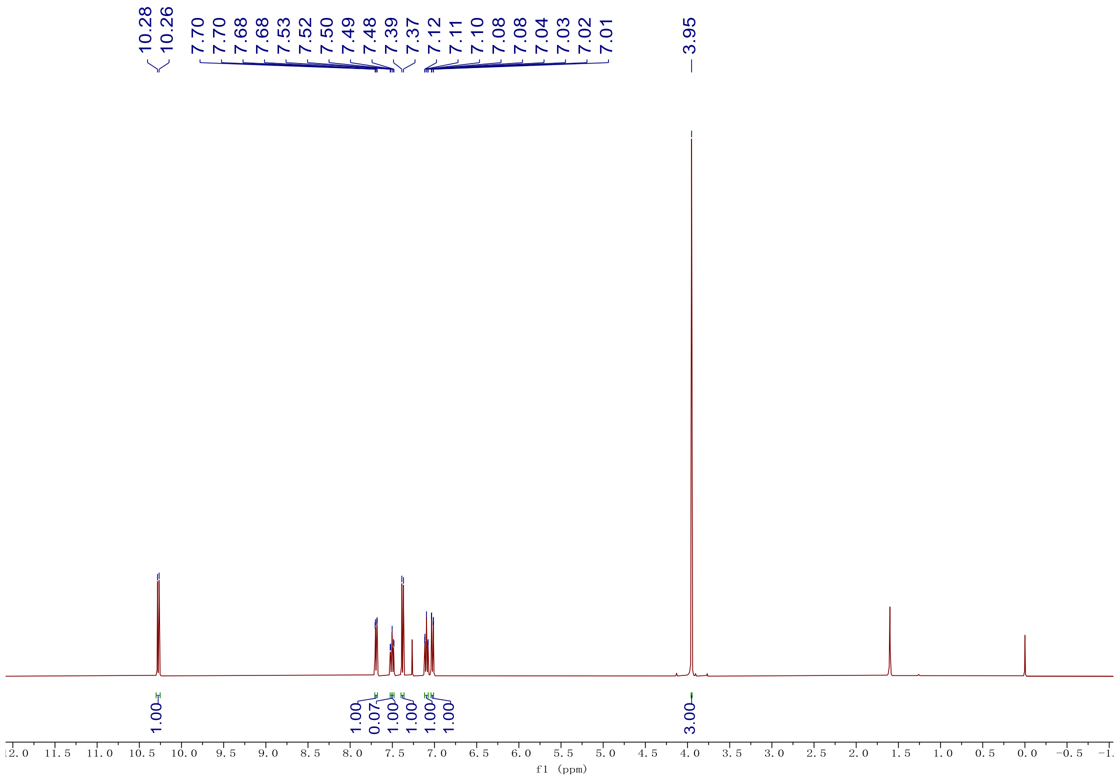

Supplementary Figure 32. ^{1}H NMR (400 MHz, $CDCl_3$)

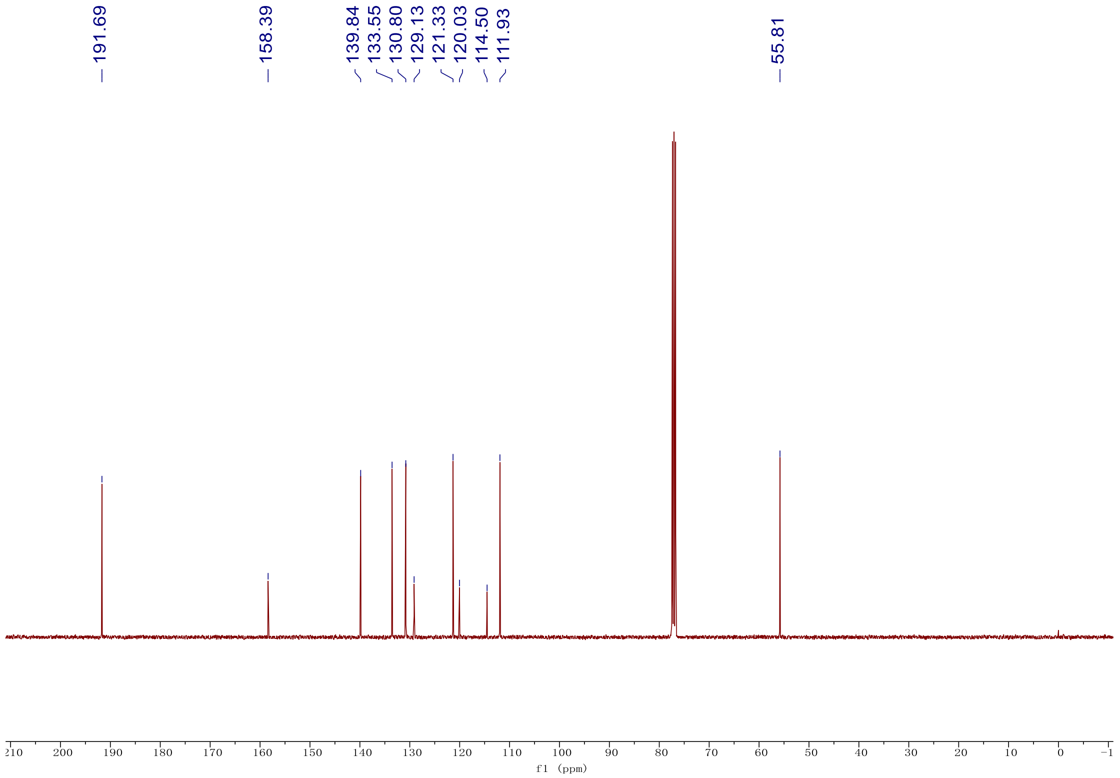

Supplementary Figure 33. ^{13}C NMR (101 MHz, $CDCl_3$)

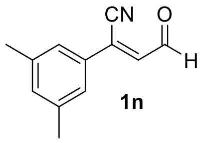

1n

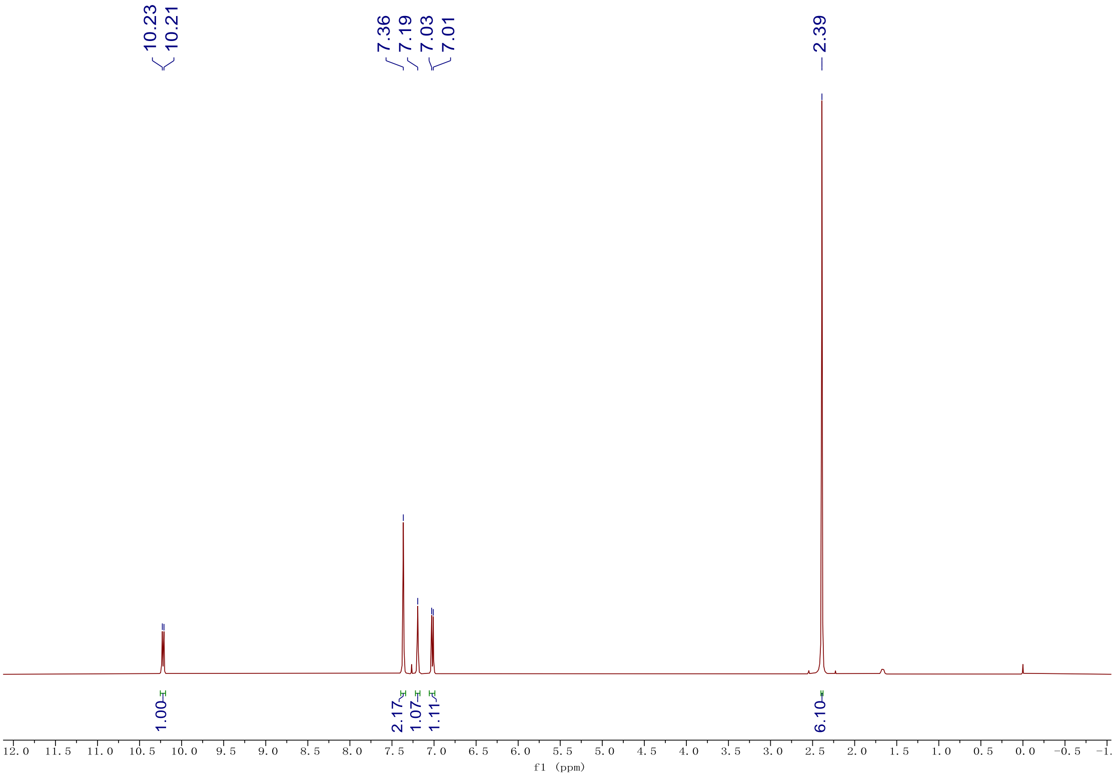

Supplementary Figure 34. ^{1}H NMR (400 MHz, $CDCl_3$)

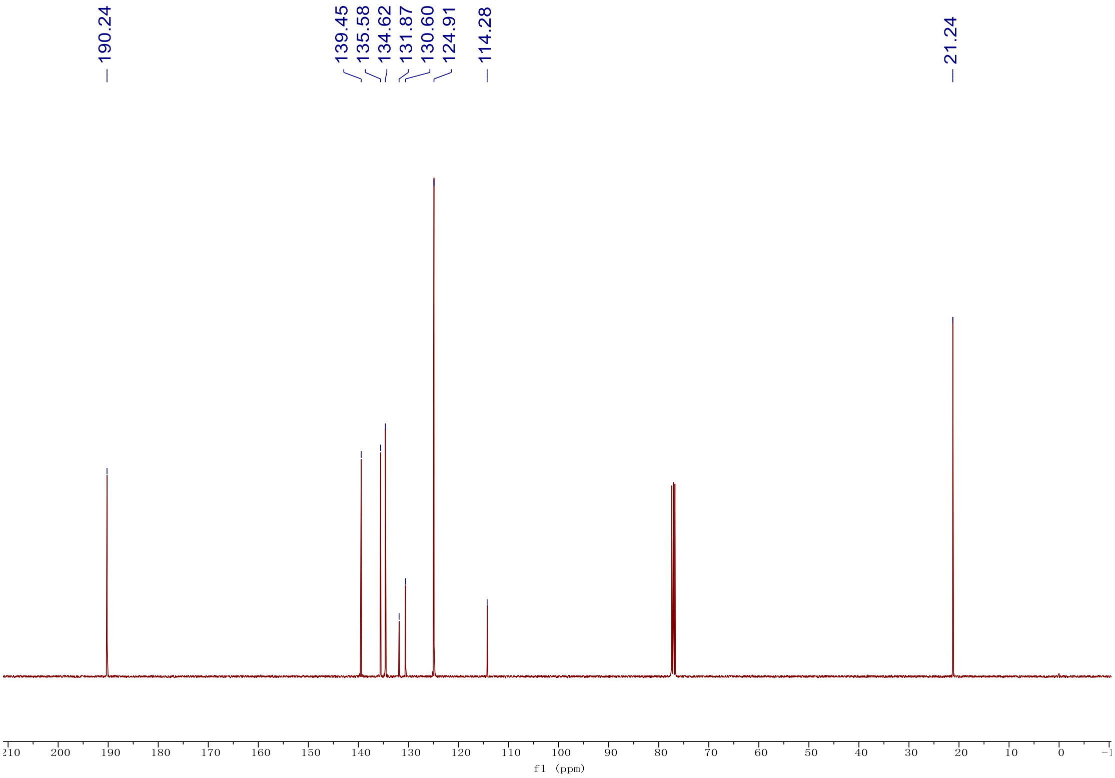

Supplementary Figure 35. ^{13}C NMR (101 MHz, $CDCl_3$)

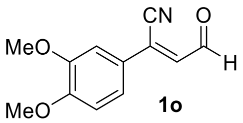

1o

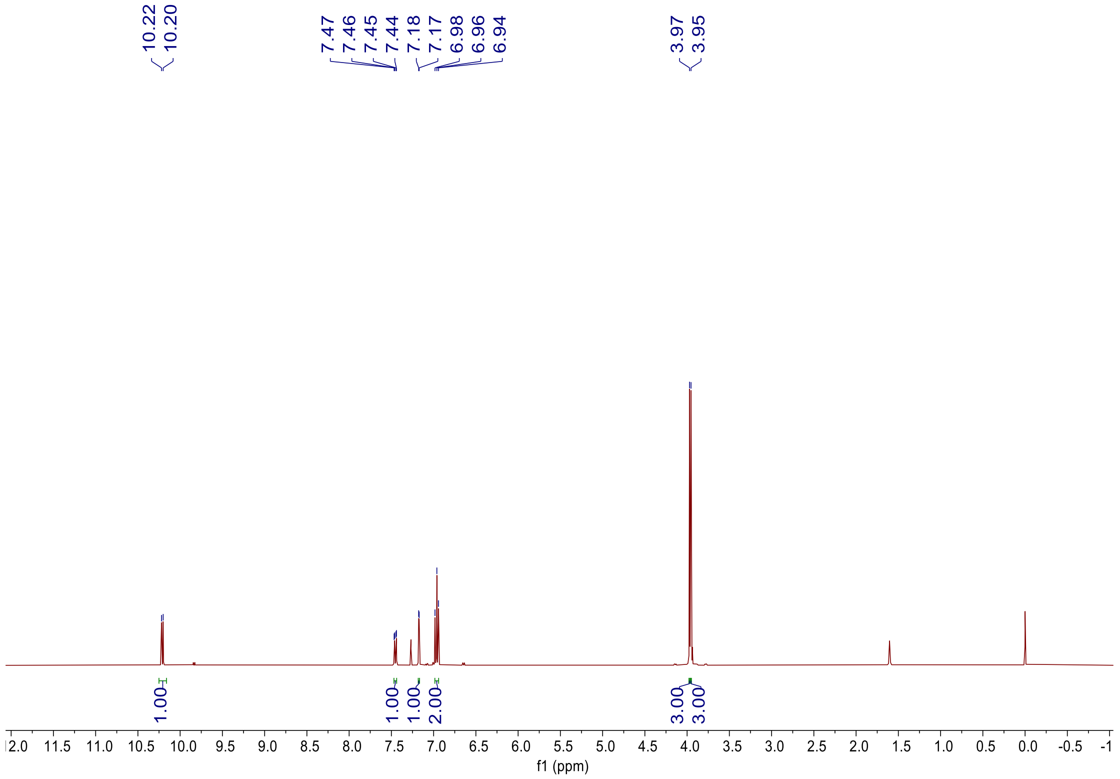

Supplementary Figure 36. ^{1}H NMR (400 MHz, $CDCl_3$)

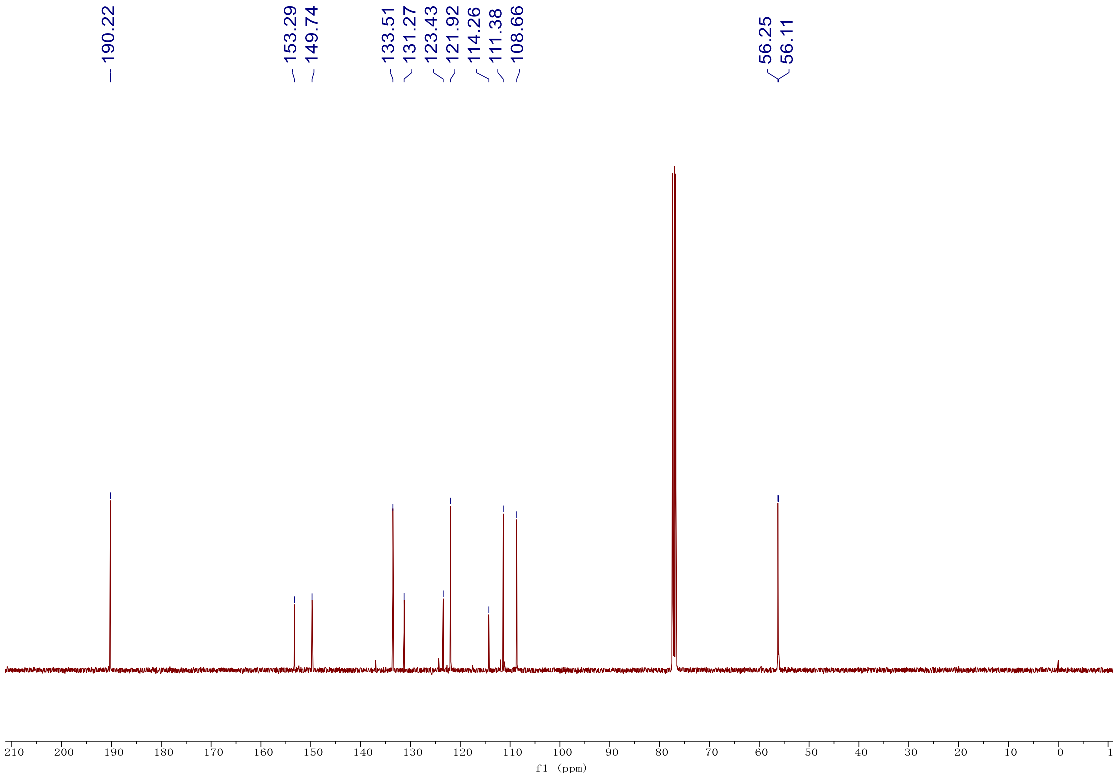

Supplementary Figure 37. ^{13}C NMR (101 MHz, $CDCl_3$)

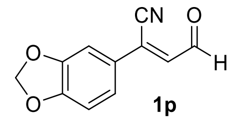

1p

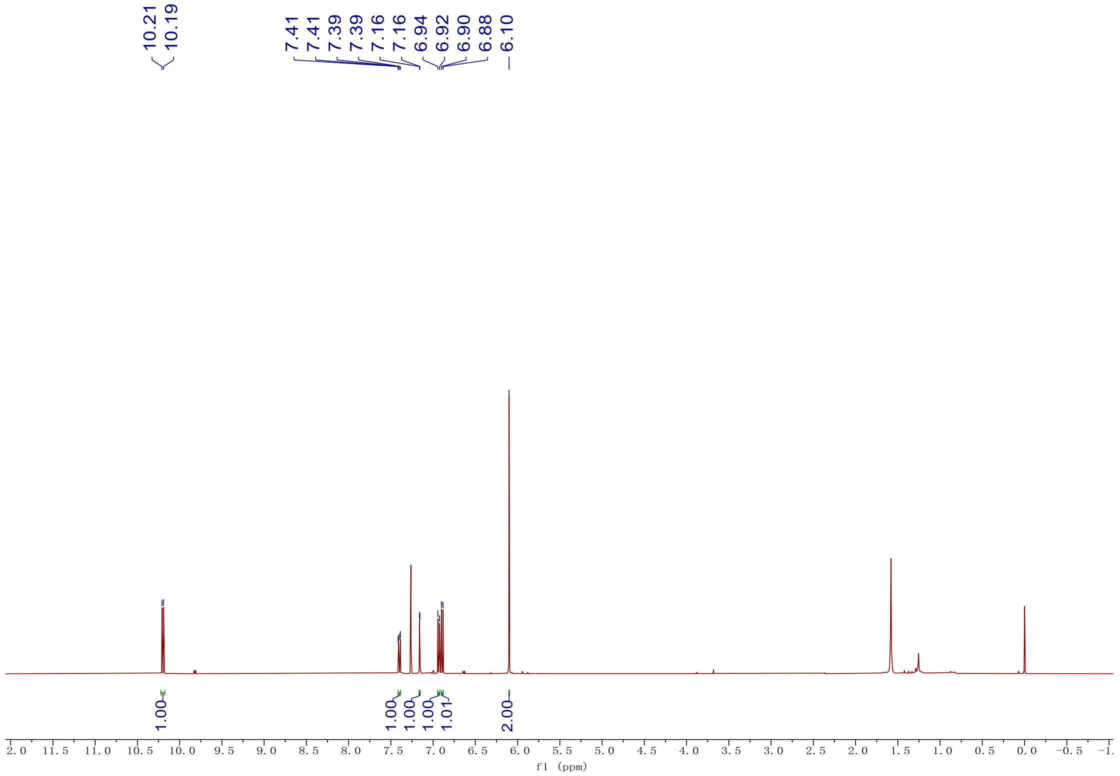

Supplementary Figure 38. ^{1}H NMR (400 MHz, $CDCl_3$)

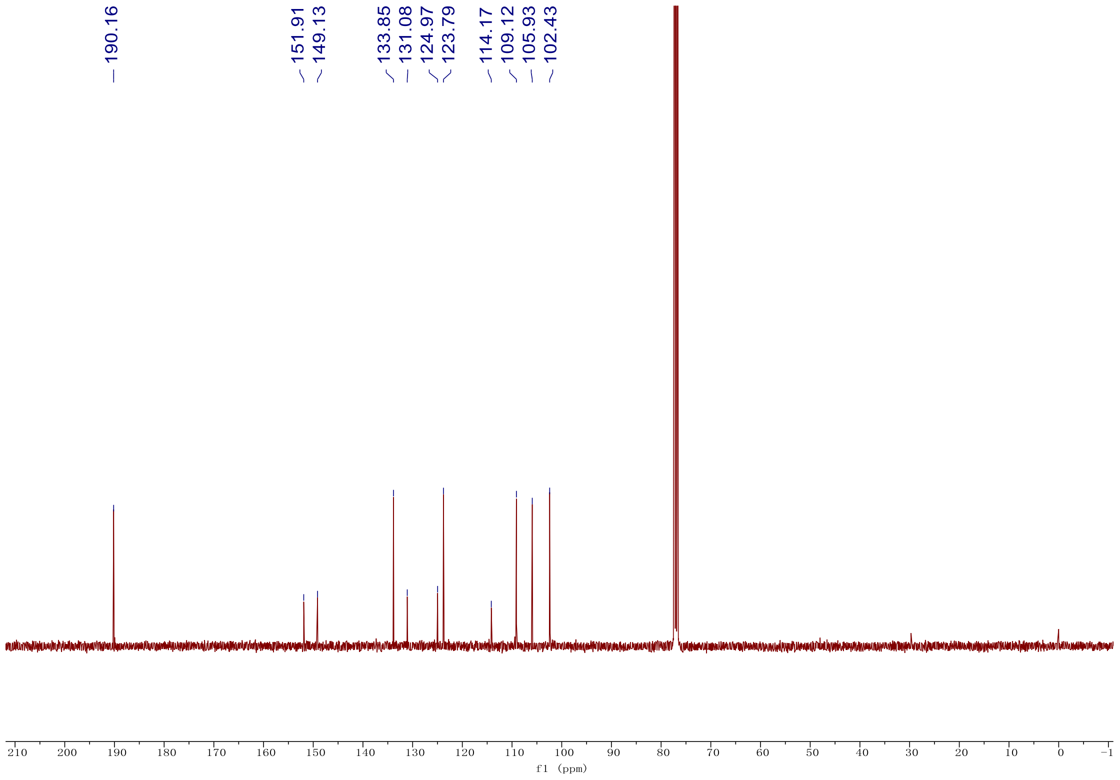

Supplementary Figure 39. ^{13}C NMR (101 MHz, $CDCl_3$)

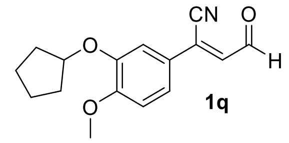

1q

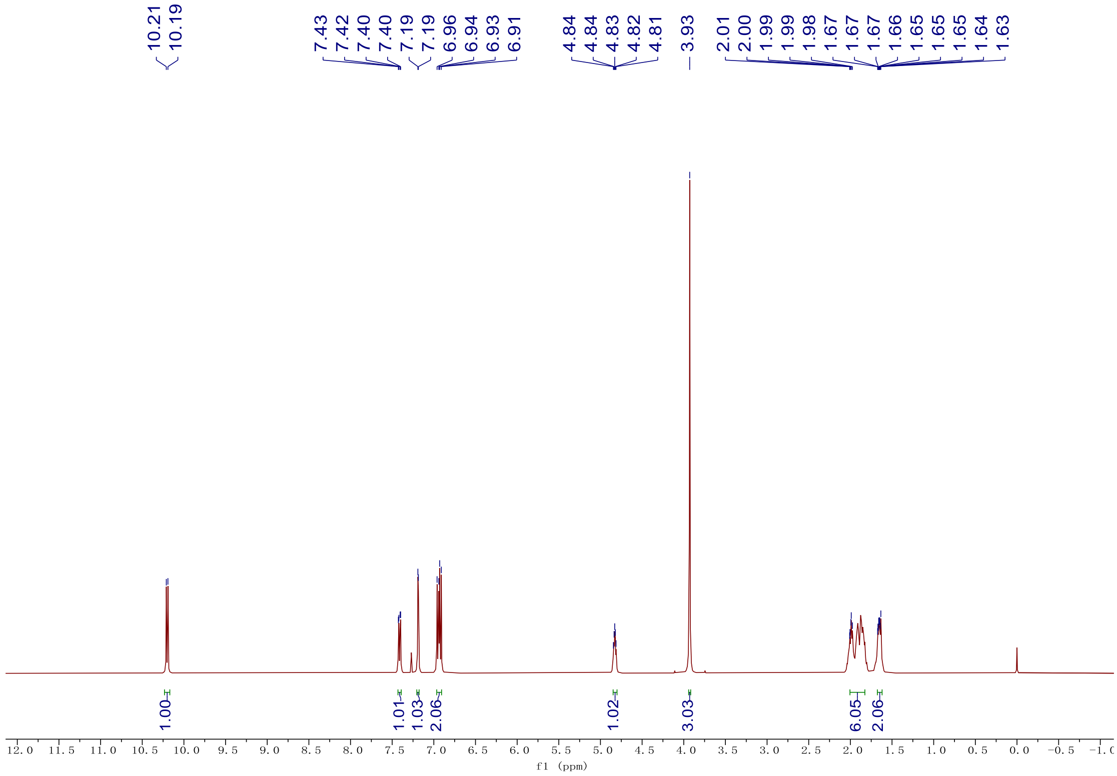

Supplementary Figure 40. ^{1}H NMR (400 MHz, $CDCl_3$)

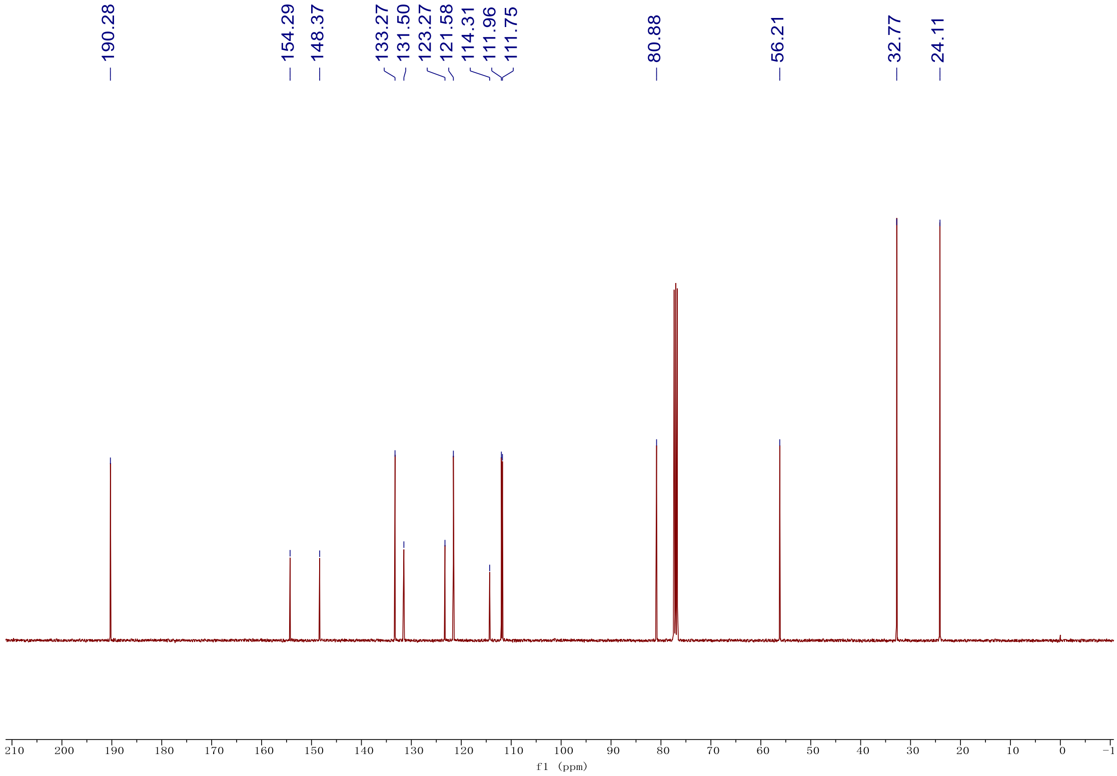

Supplementary Figure 41. ^{13}C NMR (101 MHz, $CDCl_3$)

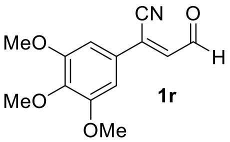

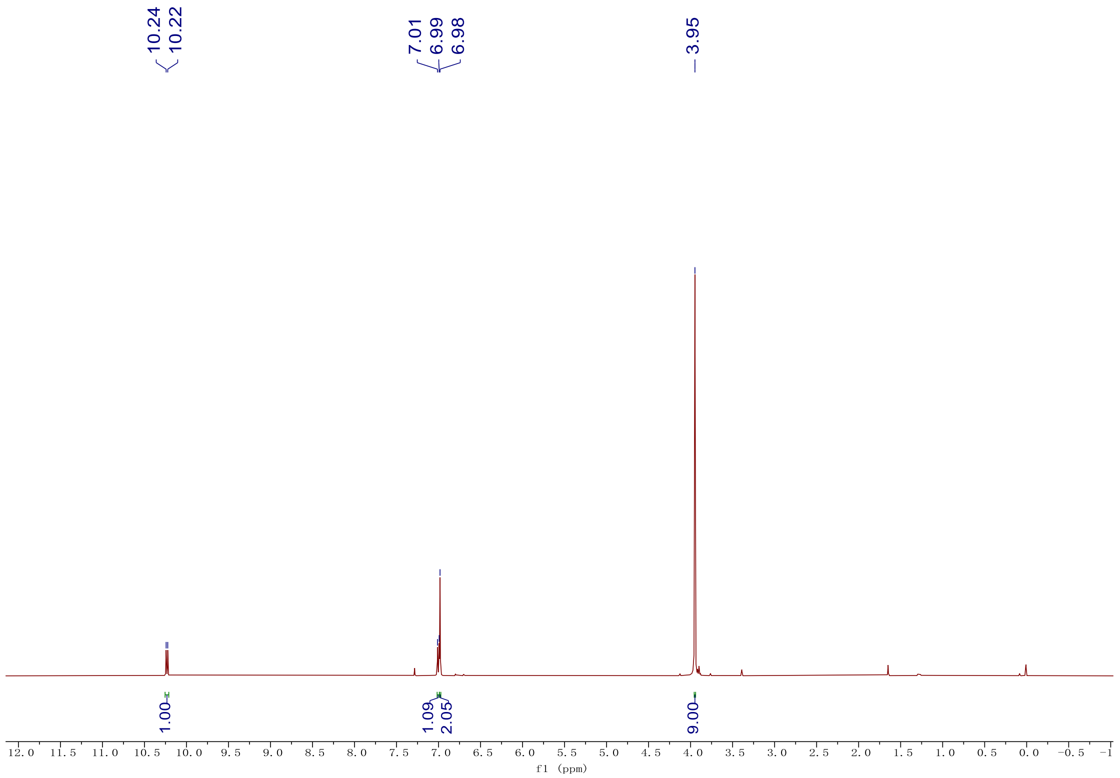

Supplementary Figure 42. ^{1}H NMR (400 MHz, CDCl$_3$)

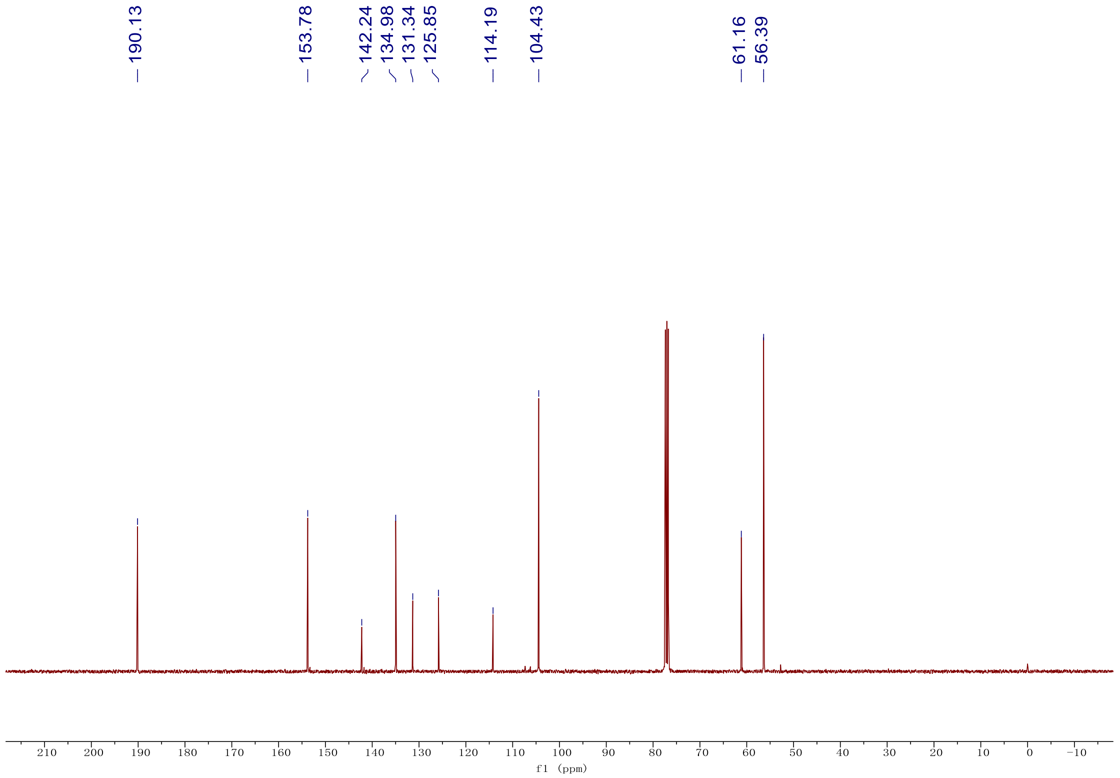

Supplementary Figure 43. ^{13}C NMR (101 MHz, CDCl$_3$)

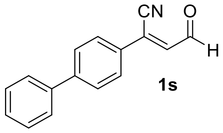

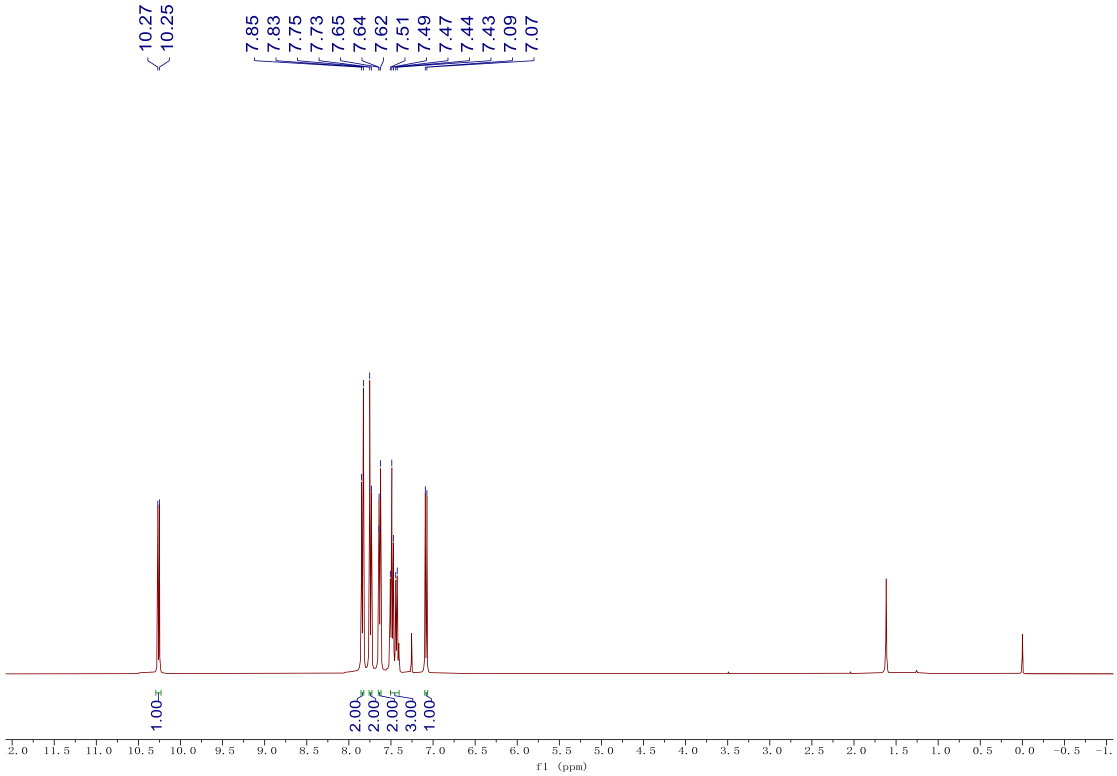

Supplementary Figure 44. ^{1}H NMR (400 MHz, $CDCl_3$)

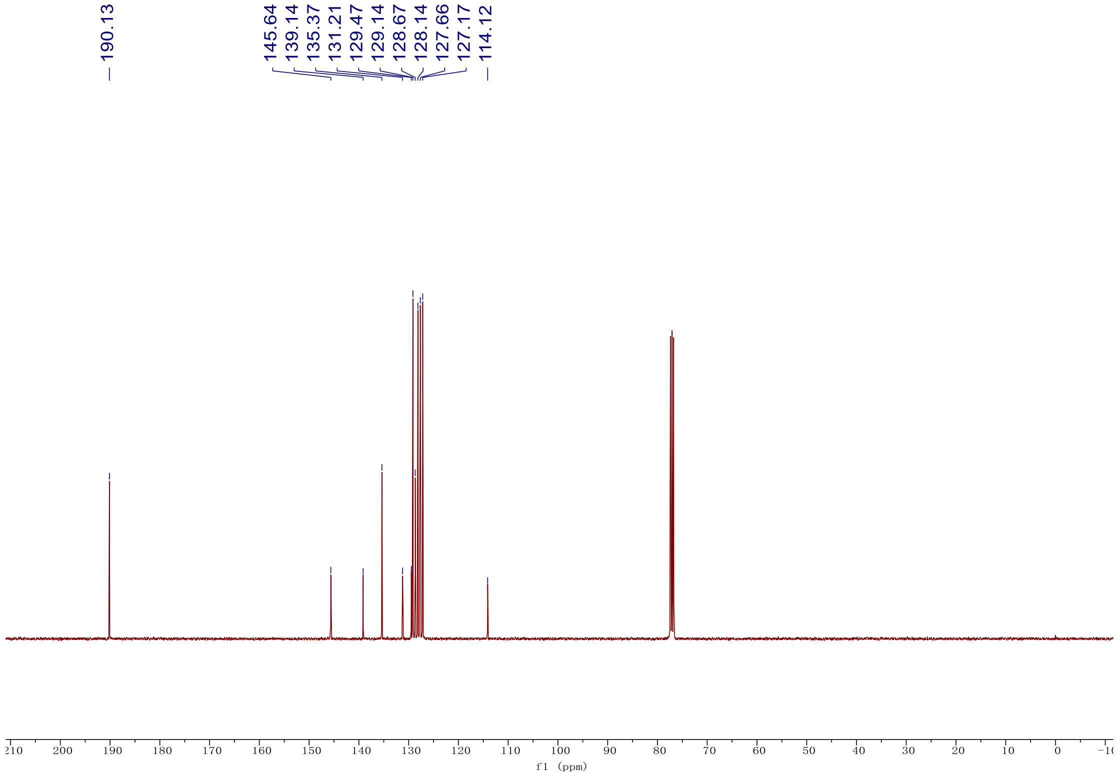

Supplementary Figure 45. ^{13}C NMR (101 MHz, $CDCl_3$)

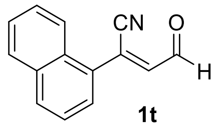

1t

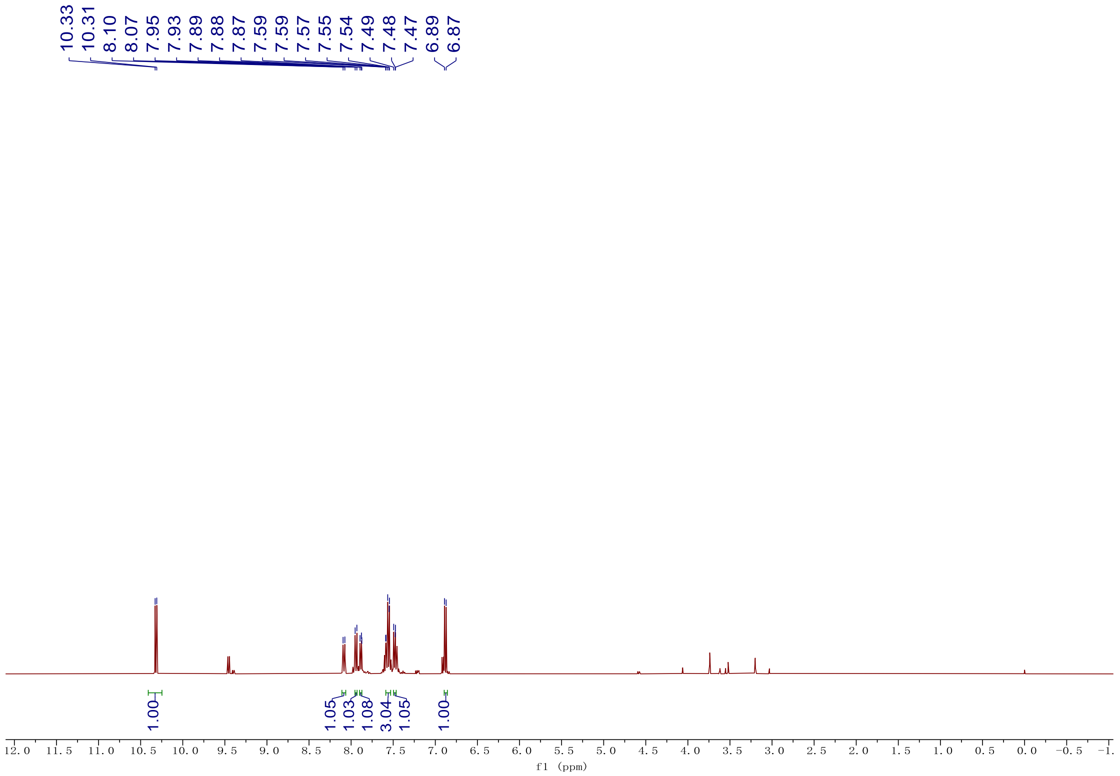

Supplementary Figure 46. ^{1}H NMR (400 MHz, $CDCl_3$)

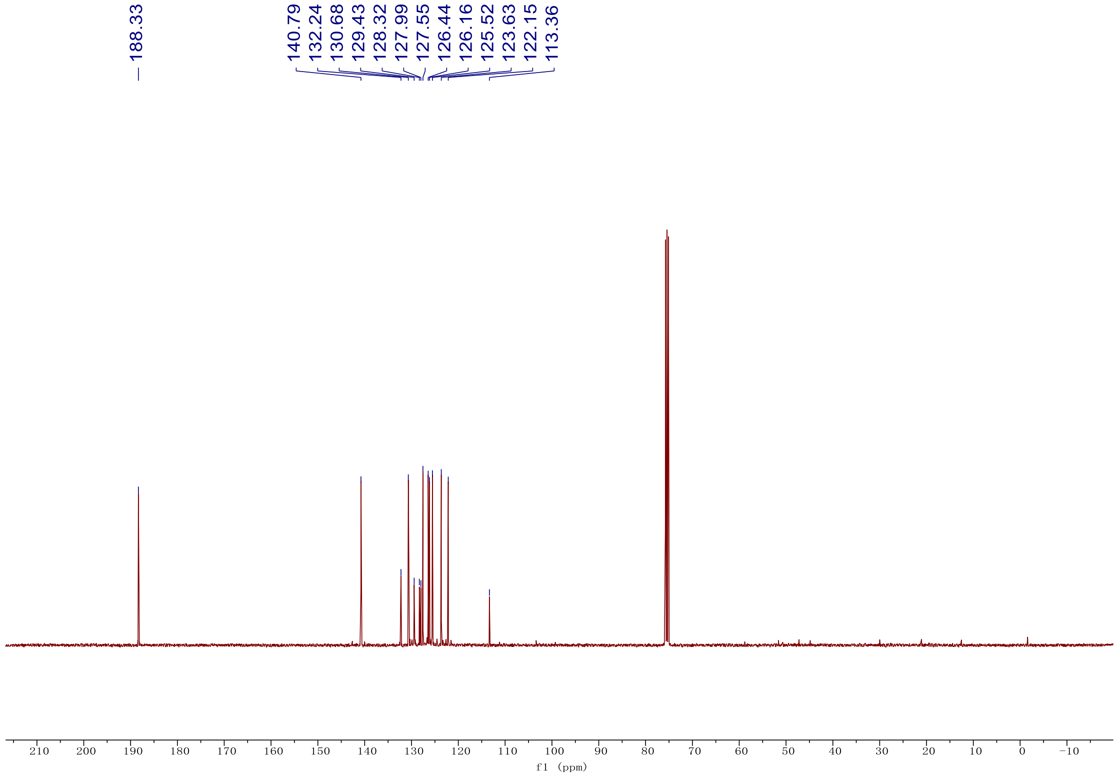

Supplementary Figure 47. ^{13}C NMR (101 MHz, $CDCl_3$)

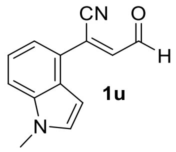

1u

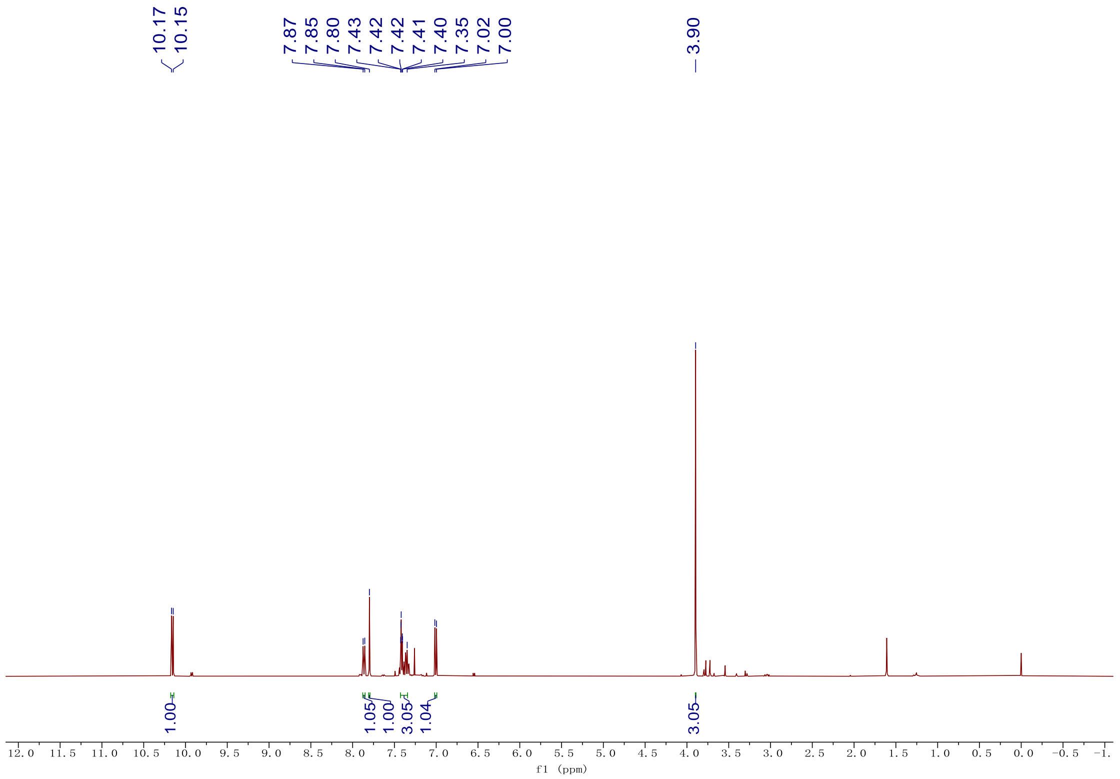

Supplementary Figure 48. ^{1}H NMR (400 MHz, $CDCl_3$)

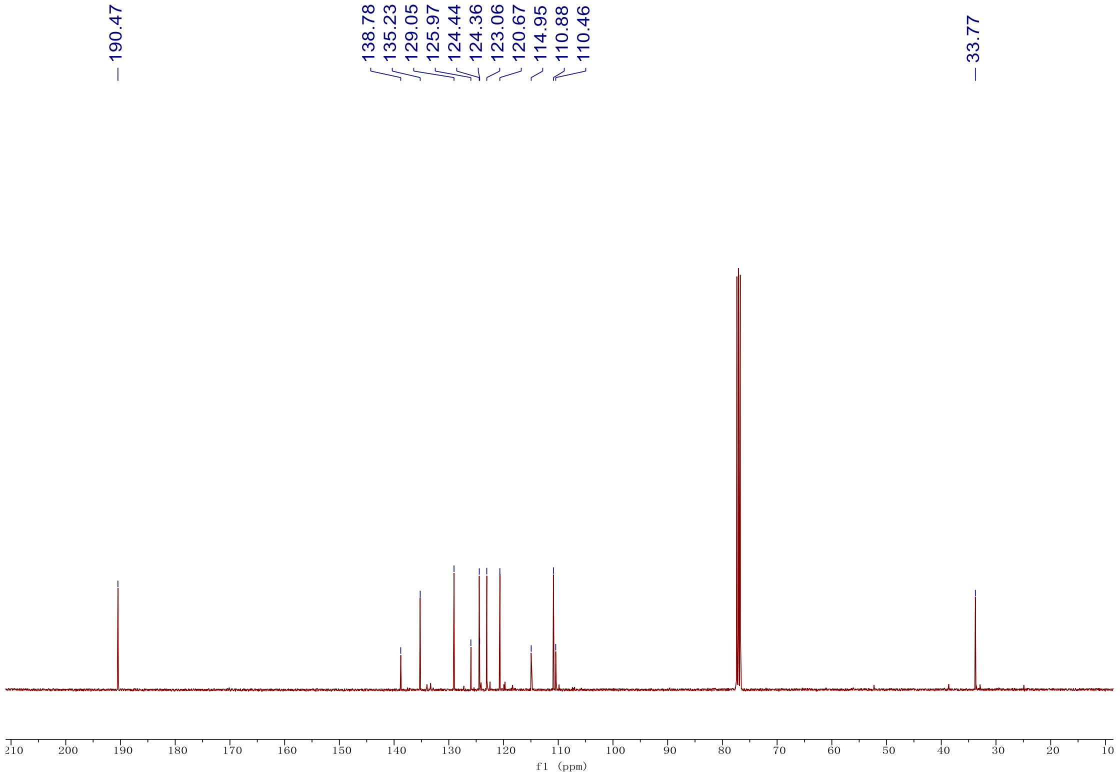

Supplementary Figure 49. ^{13}C NMR (101 MHz, $CDCl_3$)

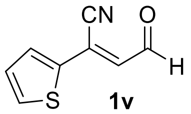

1v

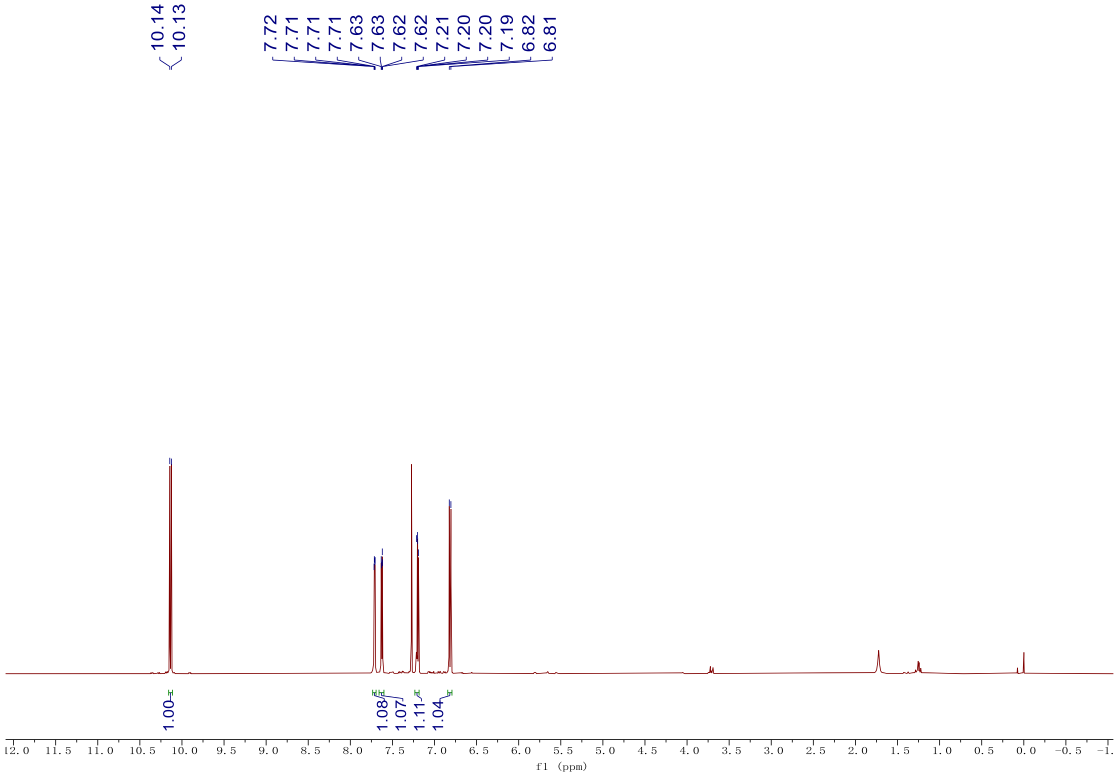

Supplementary Figure 50. ¹H NMR (400 MHz, CDCl₃)

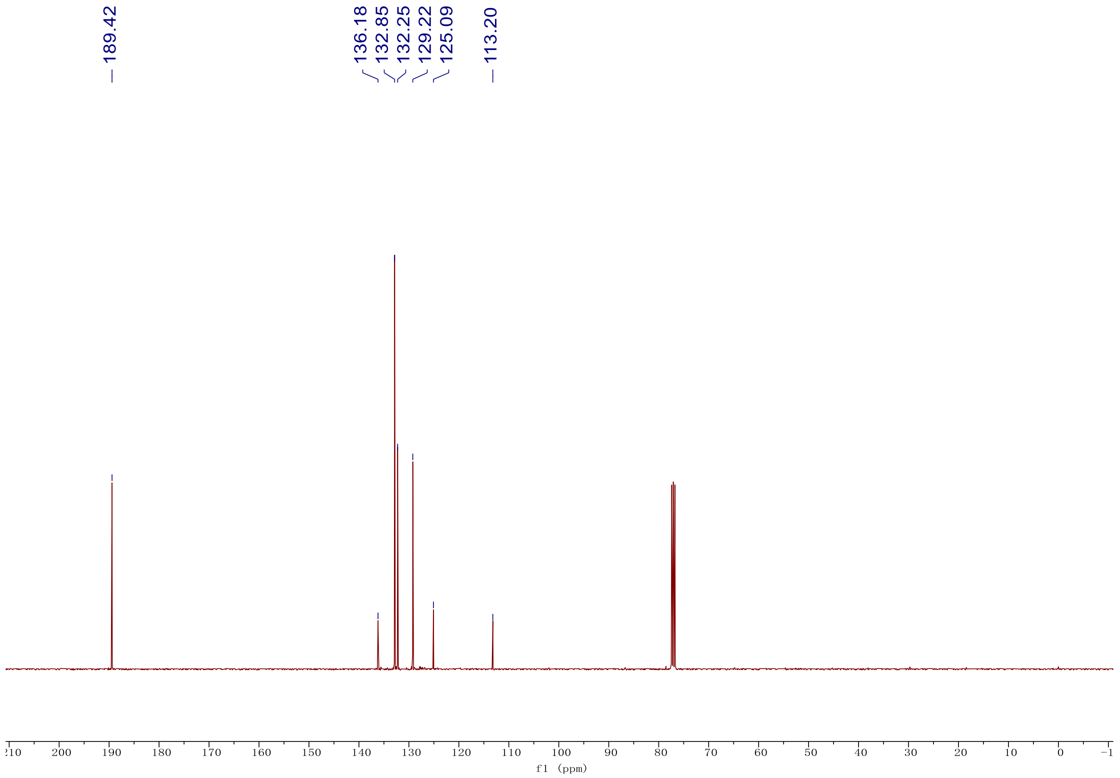

Supplementary Figure 51. ¹³C NMR (101 MHz, CDCl₃)

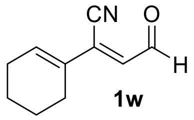

1w

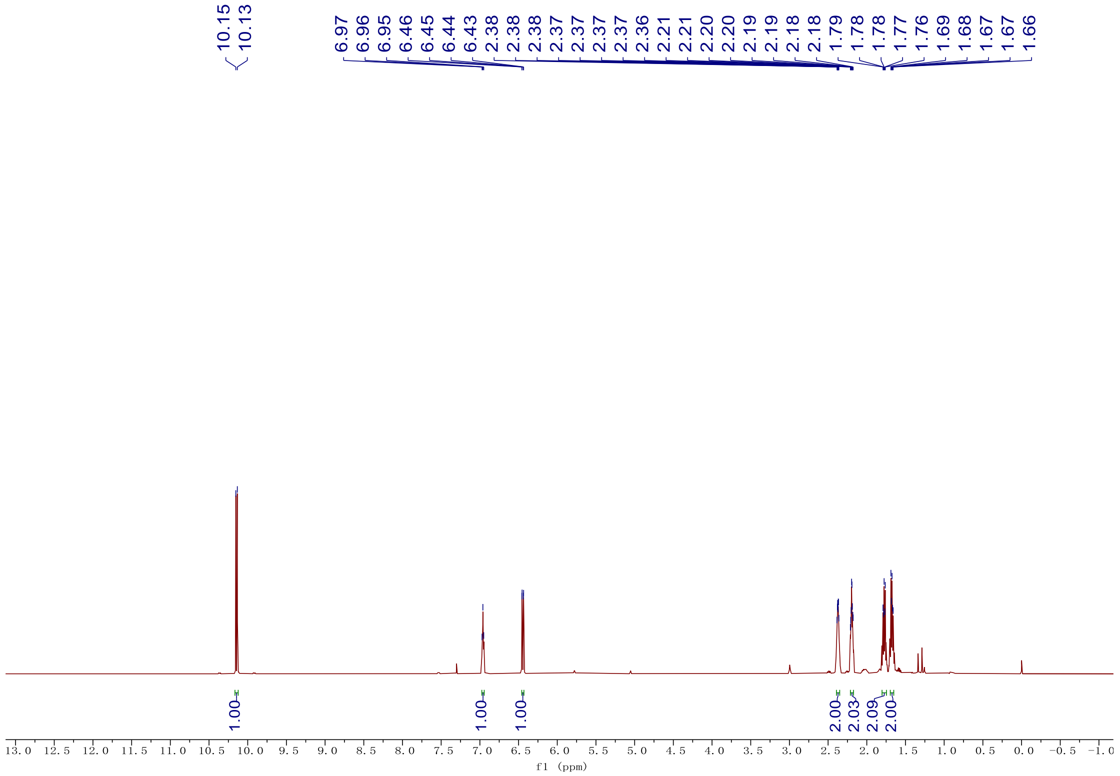

Supplementary Figure 52. ^{1}H NMR (400 MHz, $CDCl_3$)

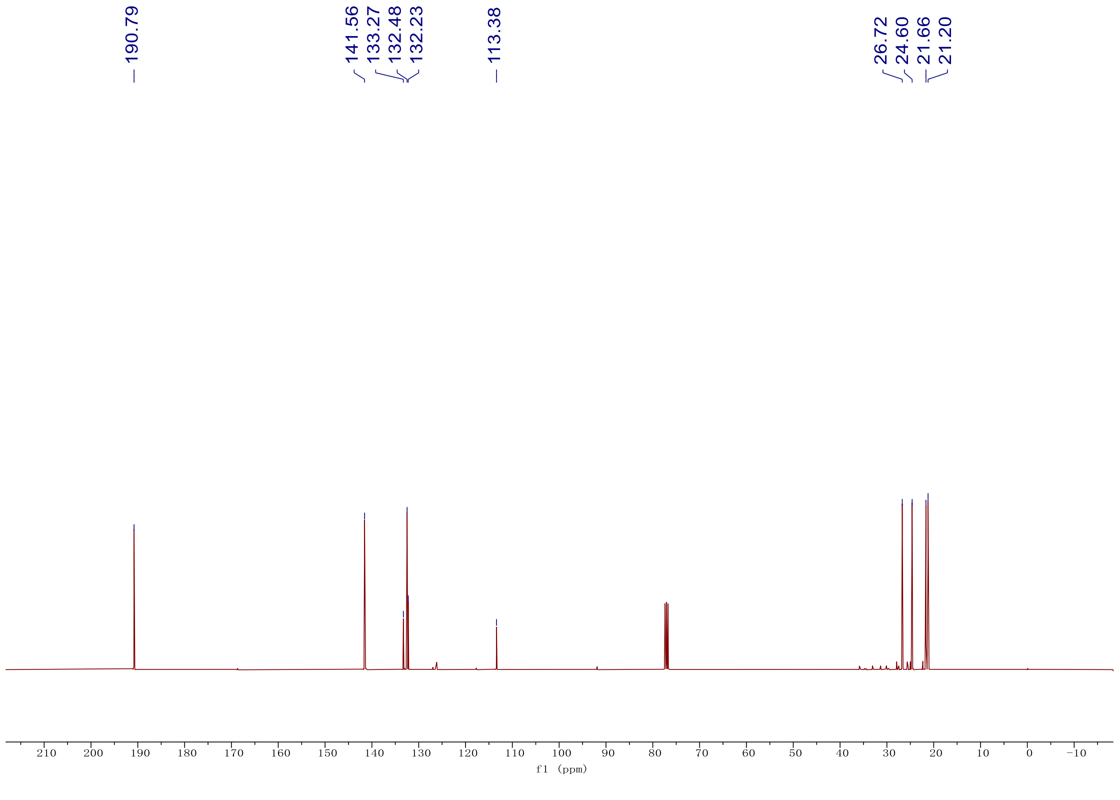

Supplementary Figure 53. ^{13}C NMR (101 MHz, $CDCl_3$)

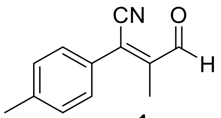

1x

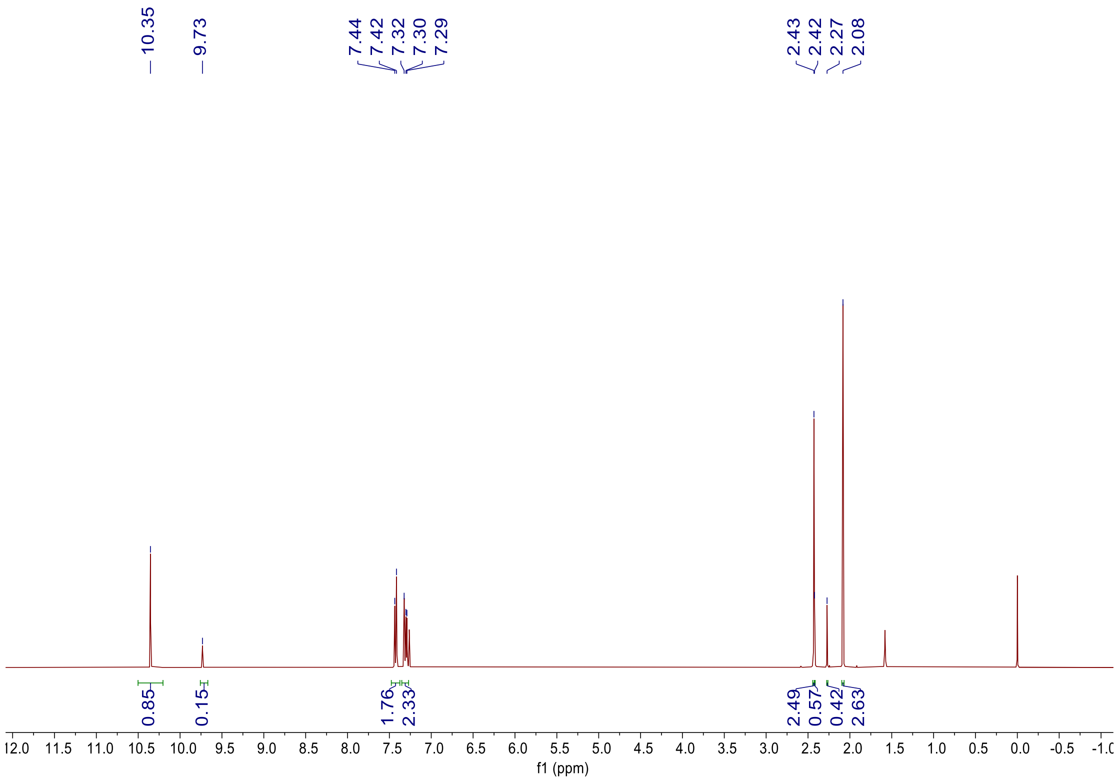

Supplementary Figure 54. ^{1}H NMR (400 MHz, $CDCl_3$)

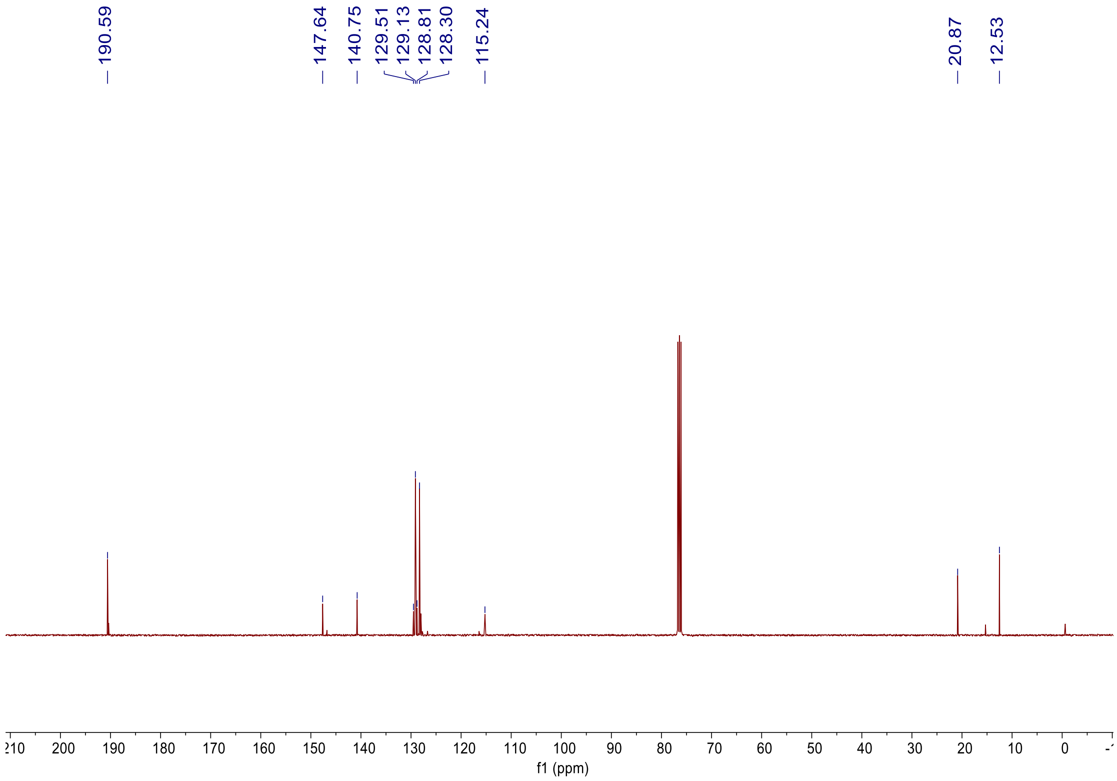

Supplementary Figure 55. ^{13}C NMR (101 MHz, $CDCl_3$)

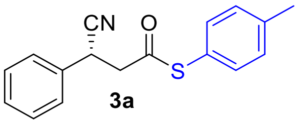

3a

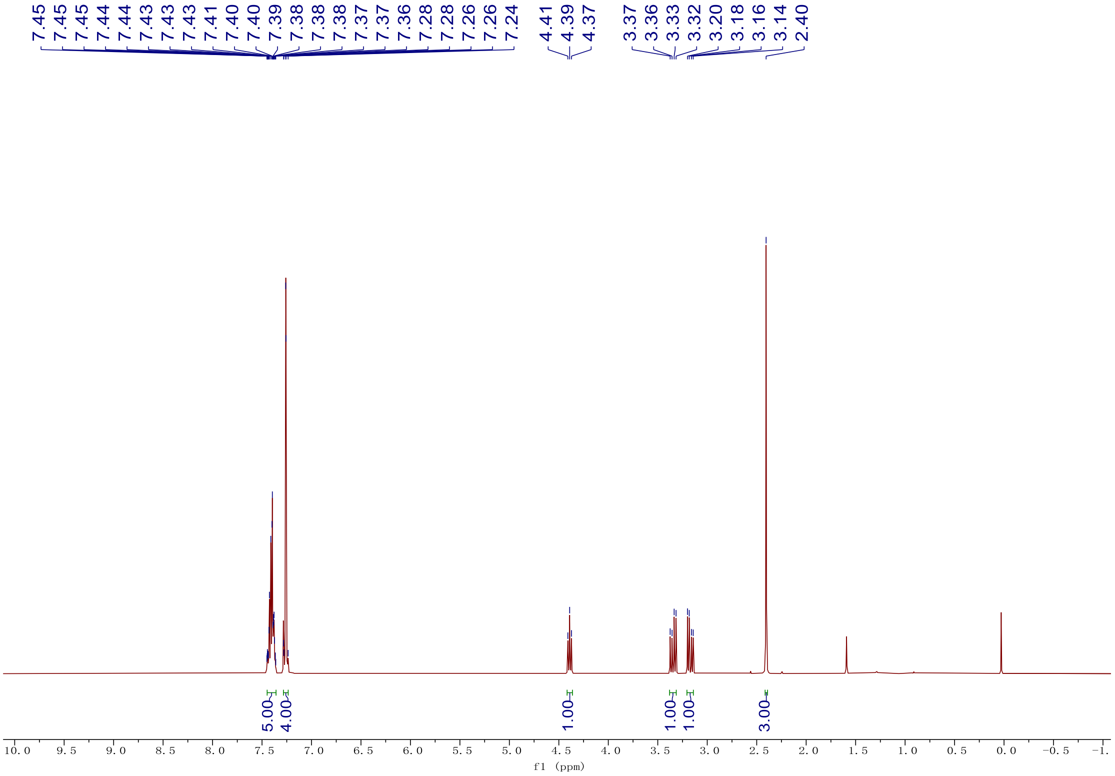

Supplementary Figure 56. ^{1}H NMR (400 MHz, $CDCl_3$)

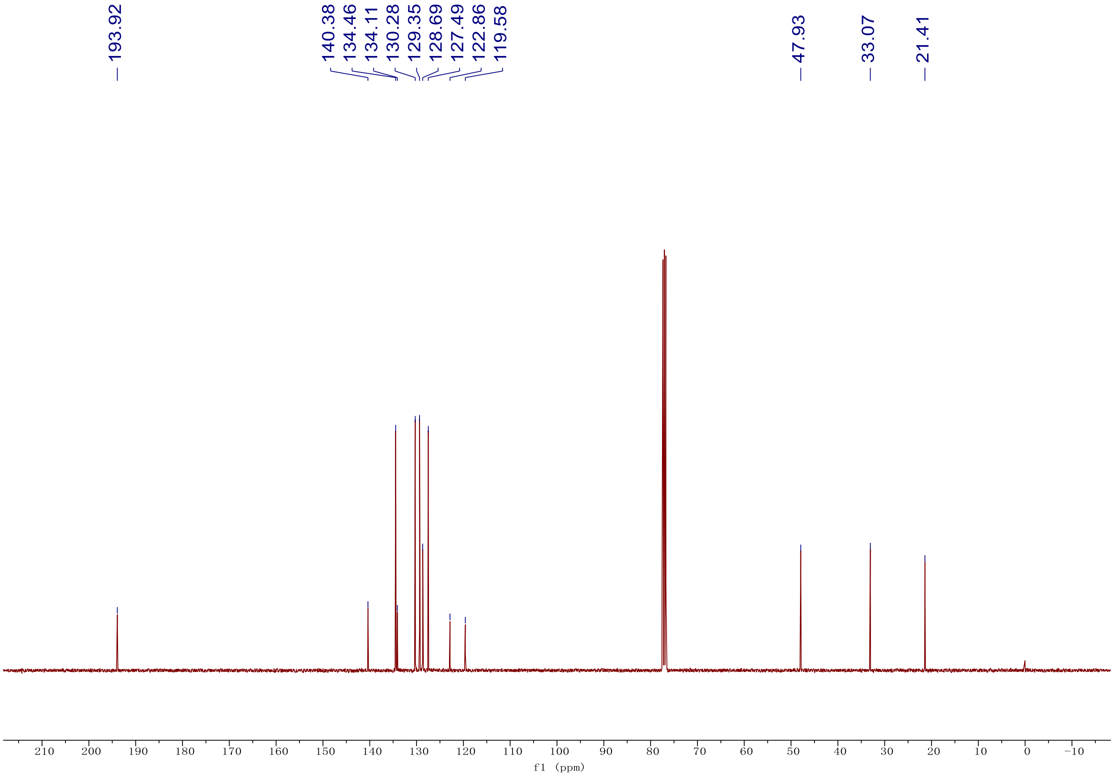

Supplementary Figure 57. ^{13}C NMR (101 MHz, $CDCl_3$)

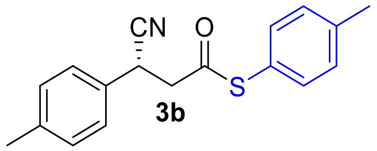

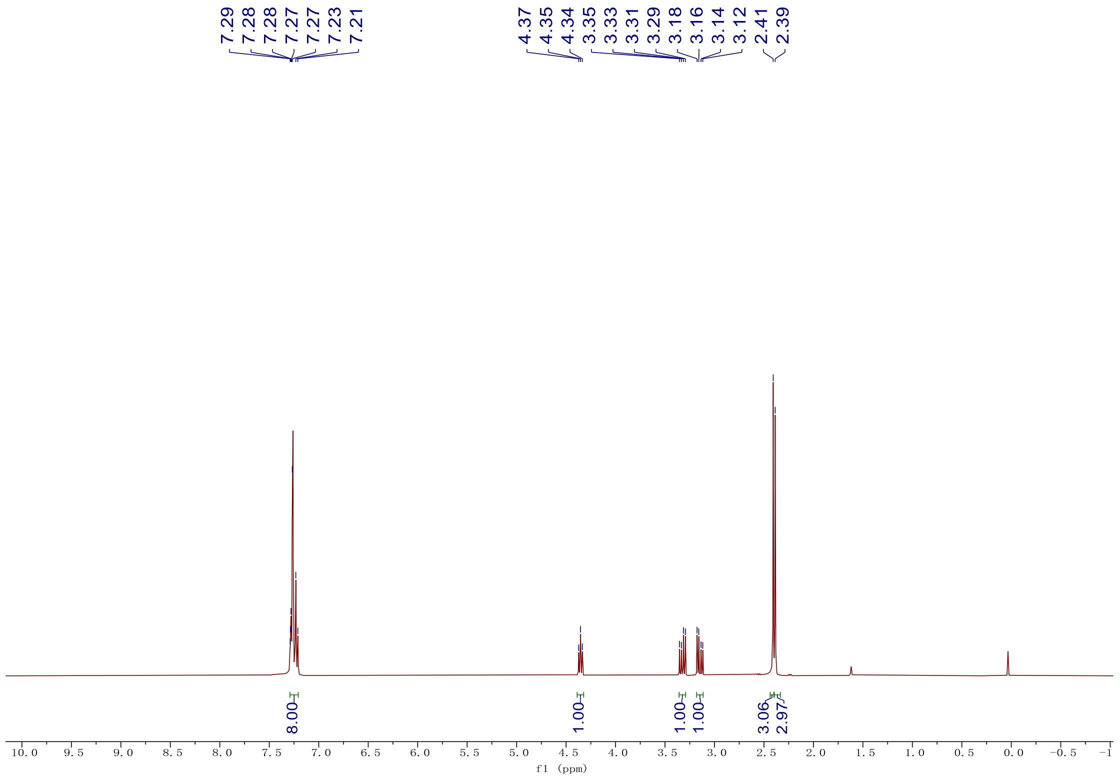

Supplementary Figure 58. ^{1}H NMR (400 MHz, CDCl$_3$)

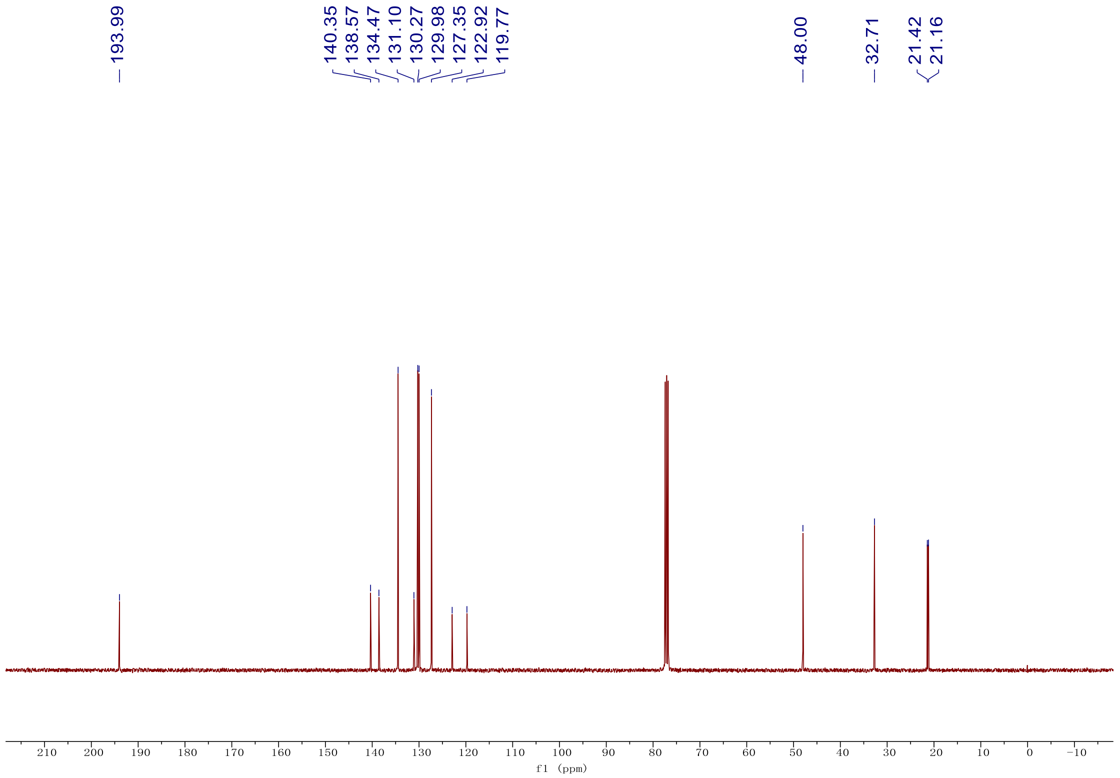

Supplementary Figure 59. ^{13}C NMR (101 MHz, CDCl$_3$)

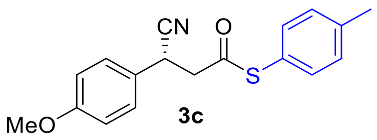

3c

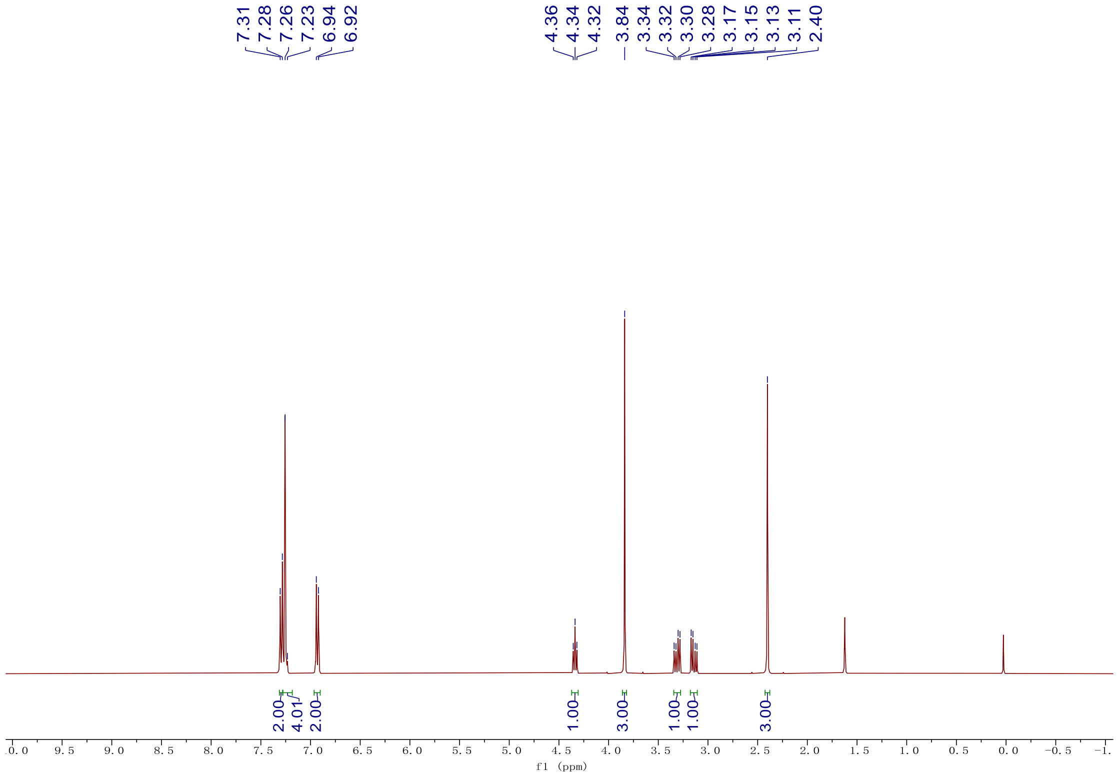

Supplementary Figure 60. ^{1}H NMR (400 MHz, $CDCl_3$)

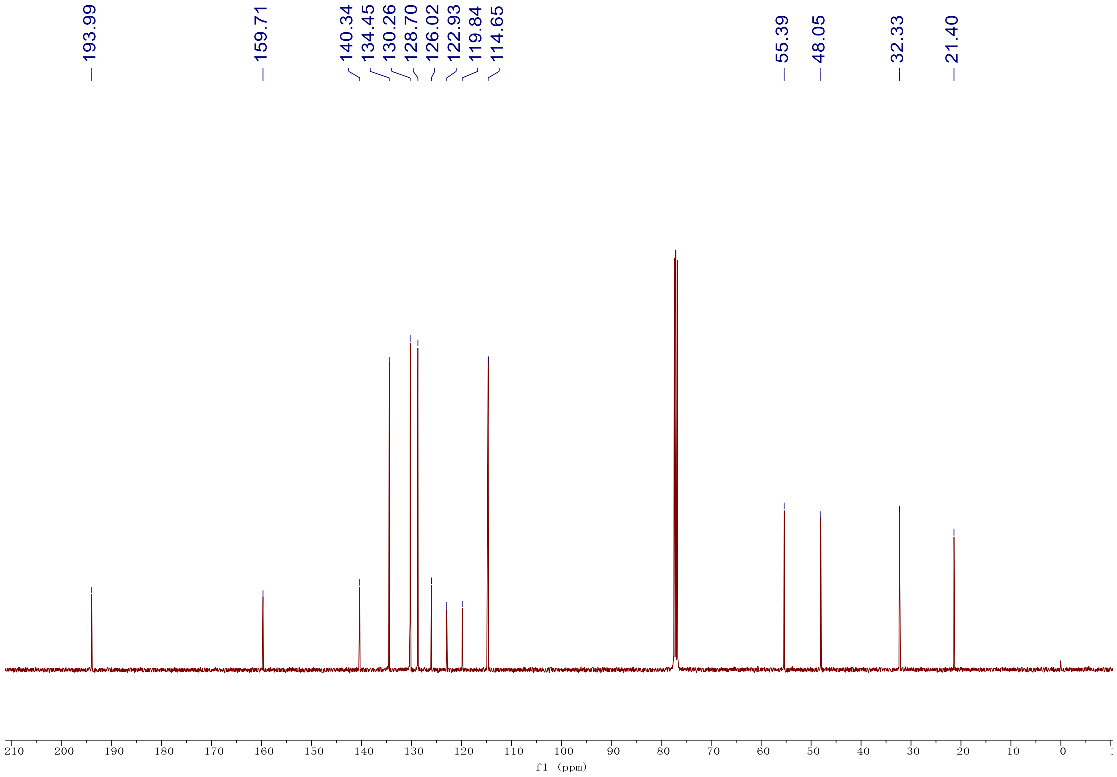

Supplementary Figure 61. ^{13}C NMR (101 MHz, $CDCl_3$)

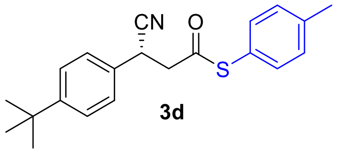

3d

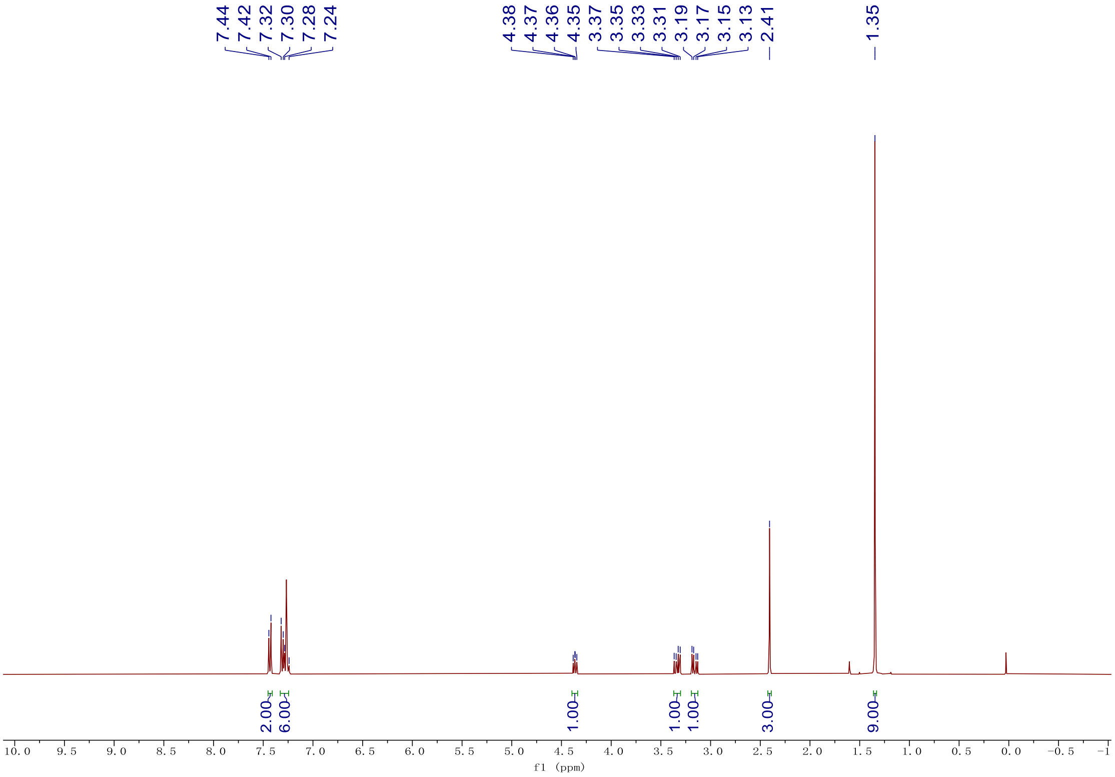

Supplementary Figure 62. ^{1}H NMR (400 MHz, $CDCl_3$)

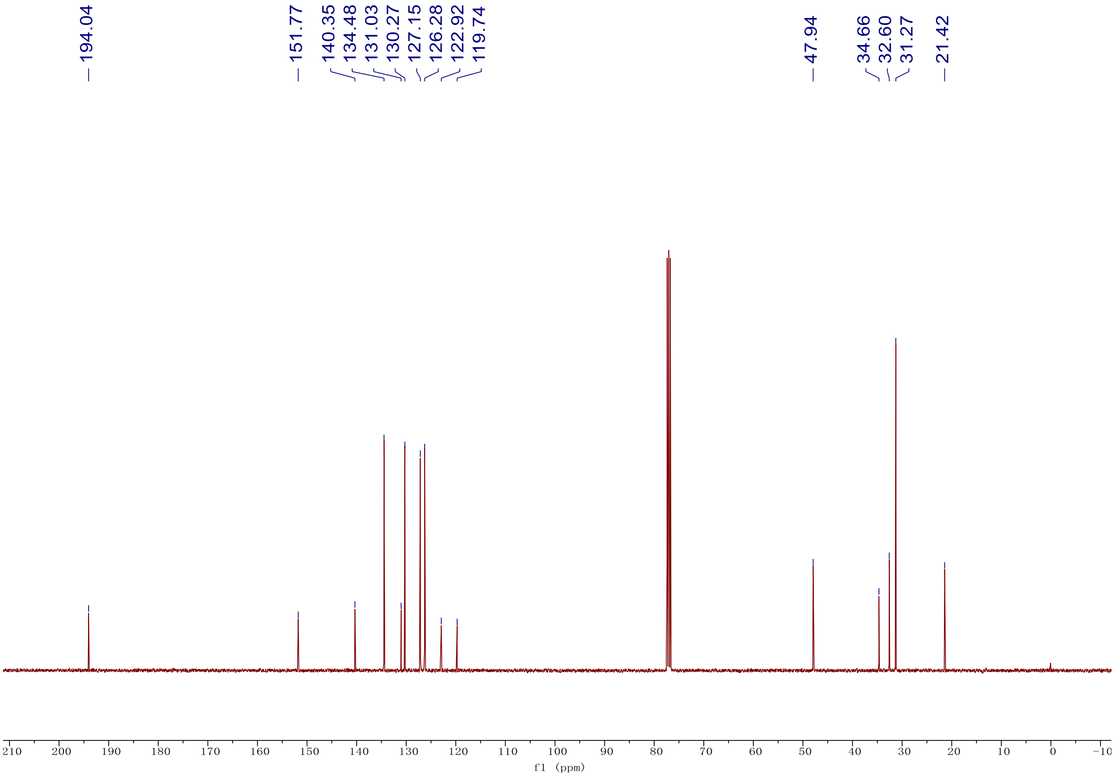

Supplementary Figure 63. ^{13}C NMR (101 MHz, $CDCl_3$)

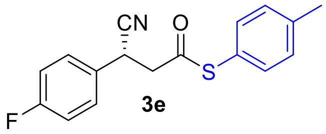

3e

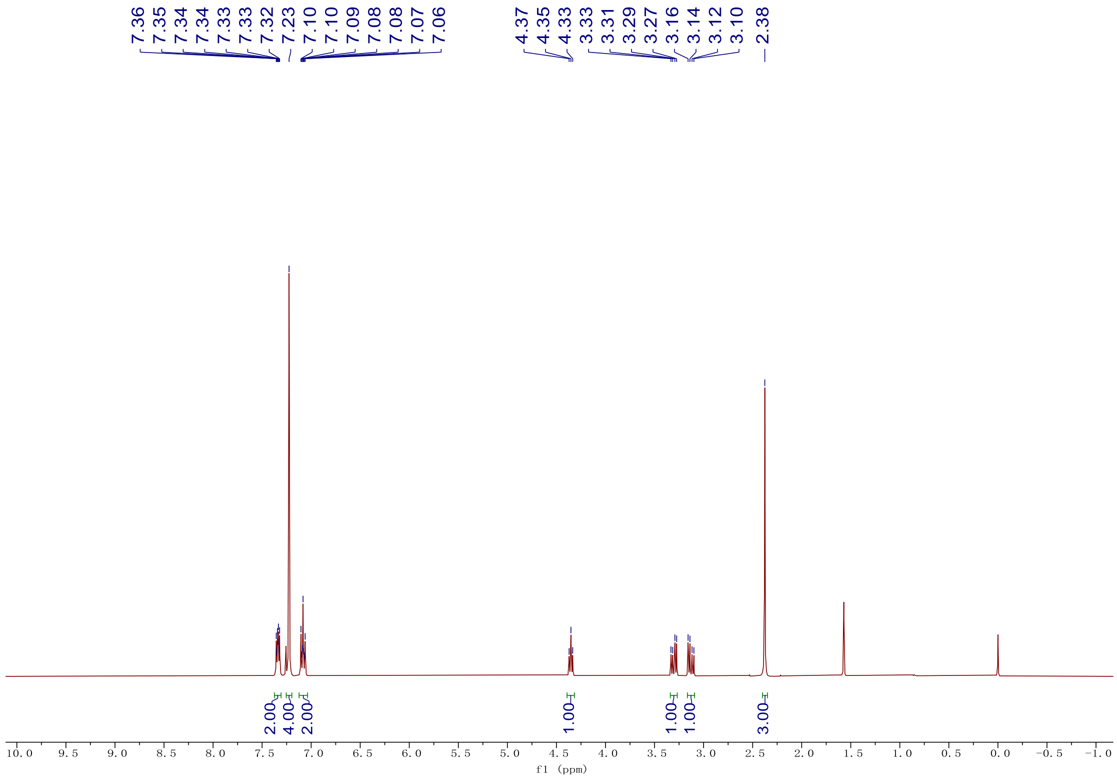

Supplementary Figure 64. ^{1}H NMR (400 MHz, $CDCl_3$)

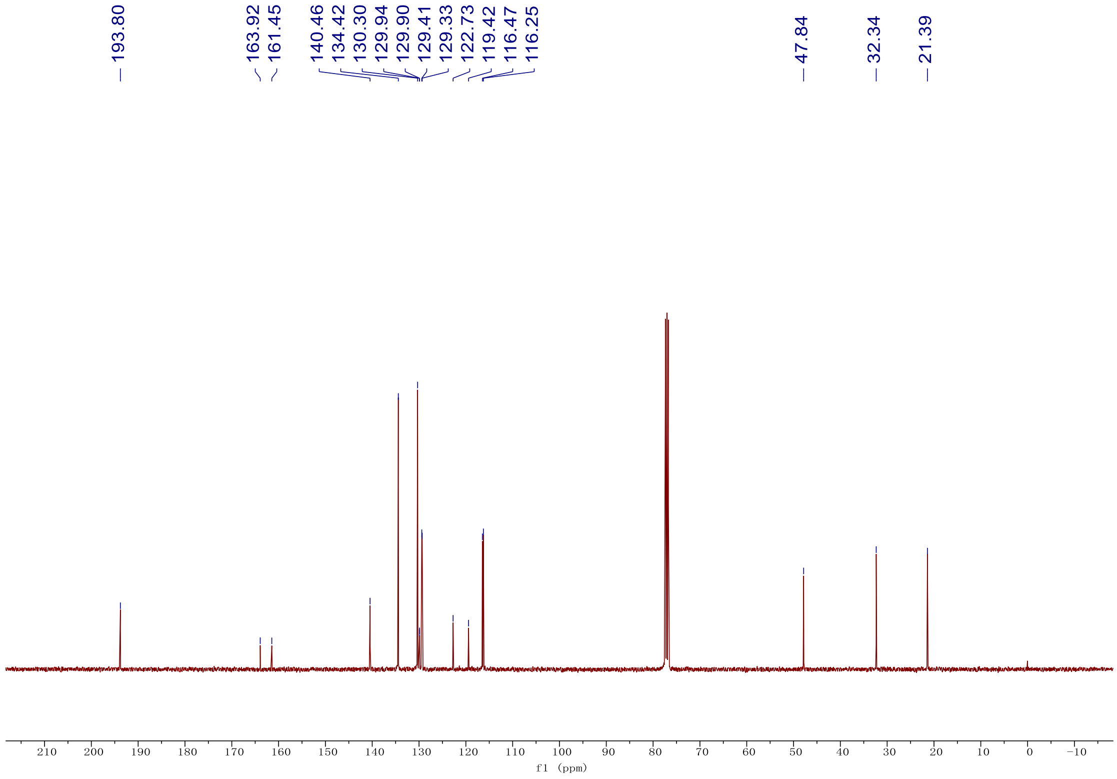

Supplementary Figure 65. ^{13}C NMR (101 MHz, $CDCl_3$)

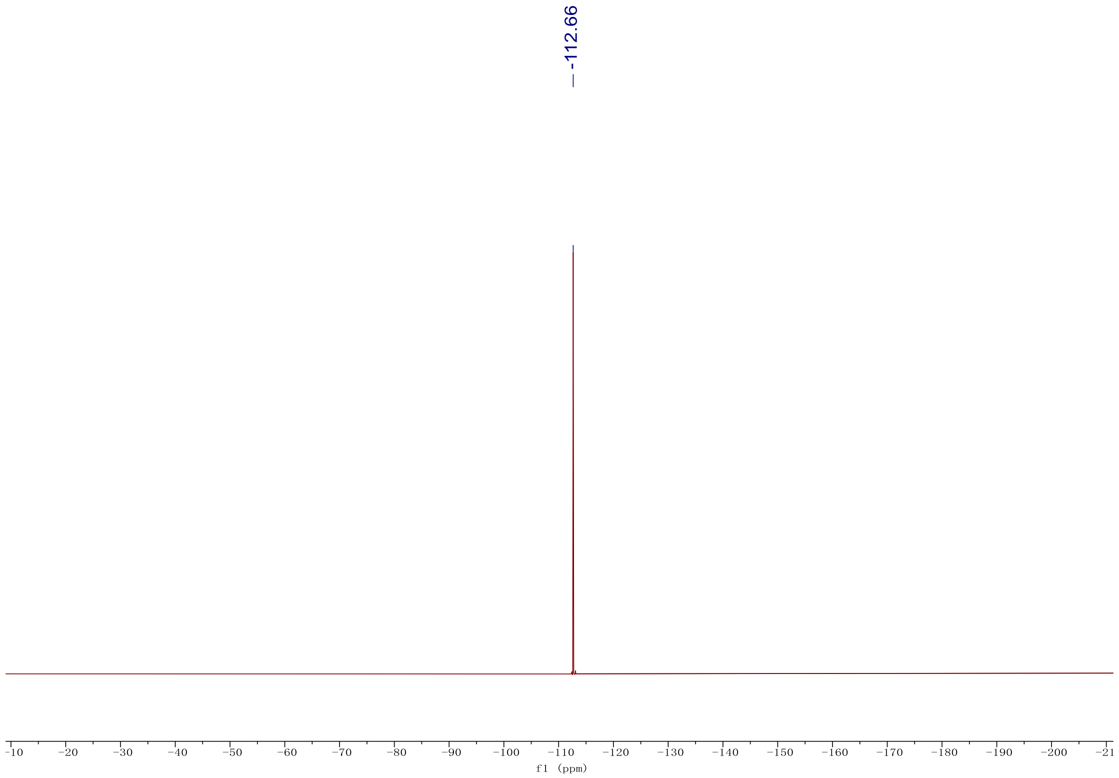

Supplementary Figure 66. ^{19}F NMR (376 MHz, $CDCl_3$)

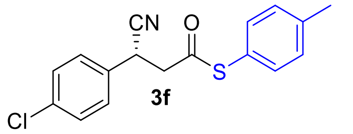

3f

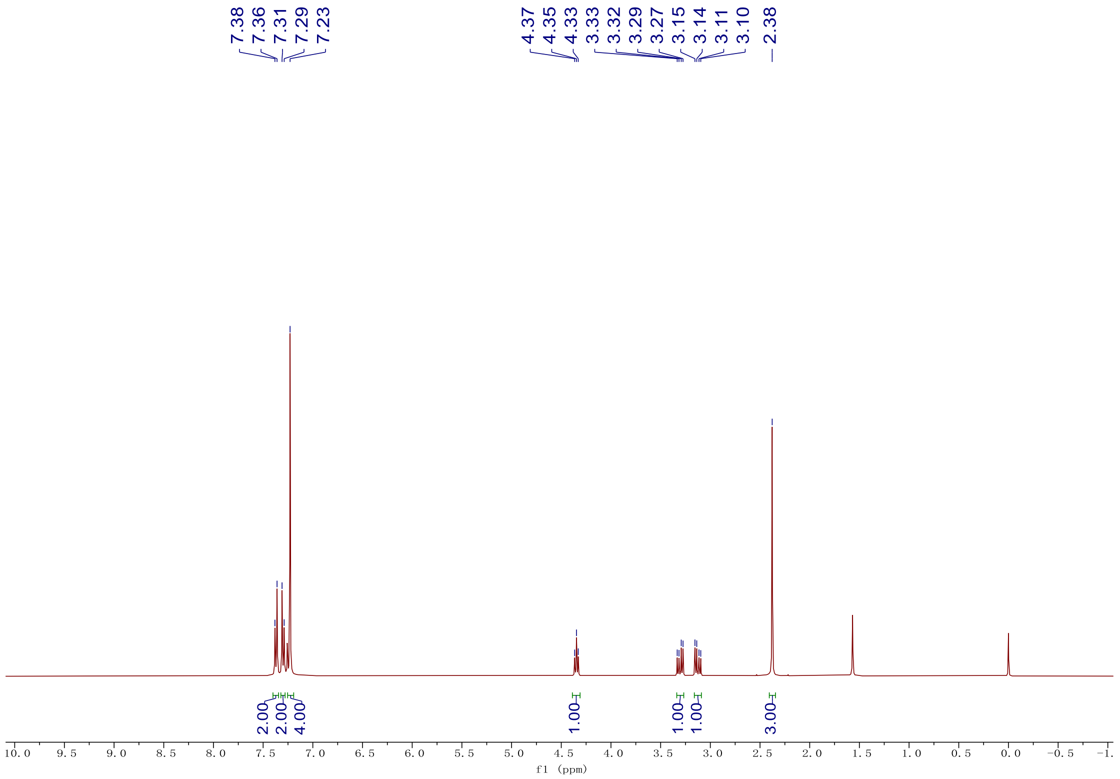

Supplementary Figure 67. ^{1}H NMR (400 MHz, $CDCl_3$)

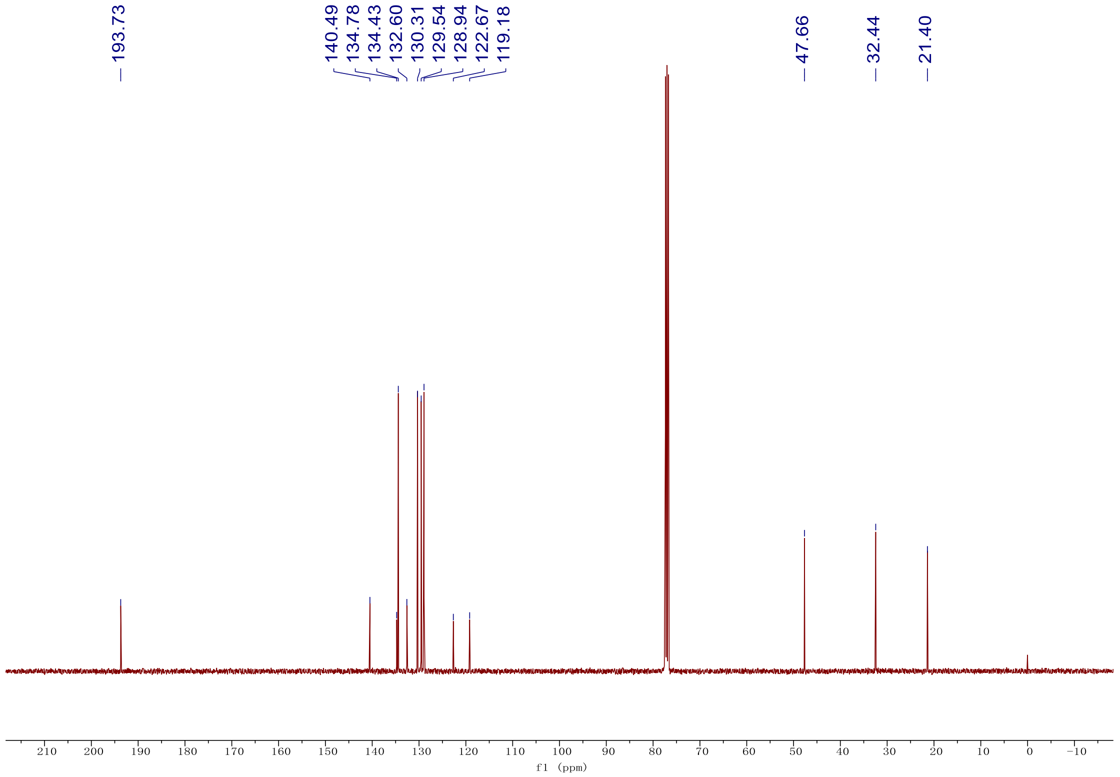

Supplementary Figure 68. ^{13}C NMR (101 MHz, $CDCl_3$)

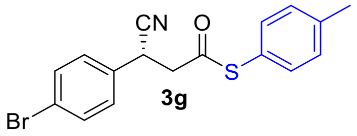

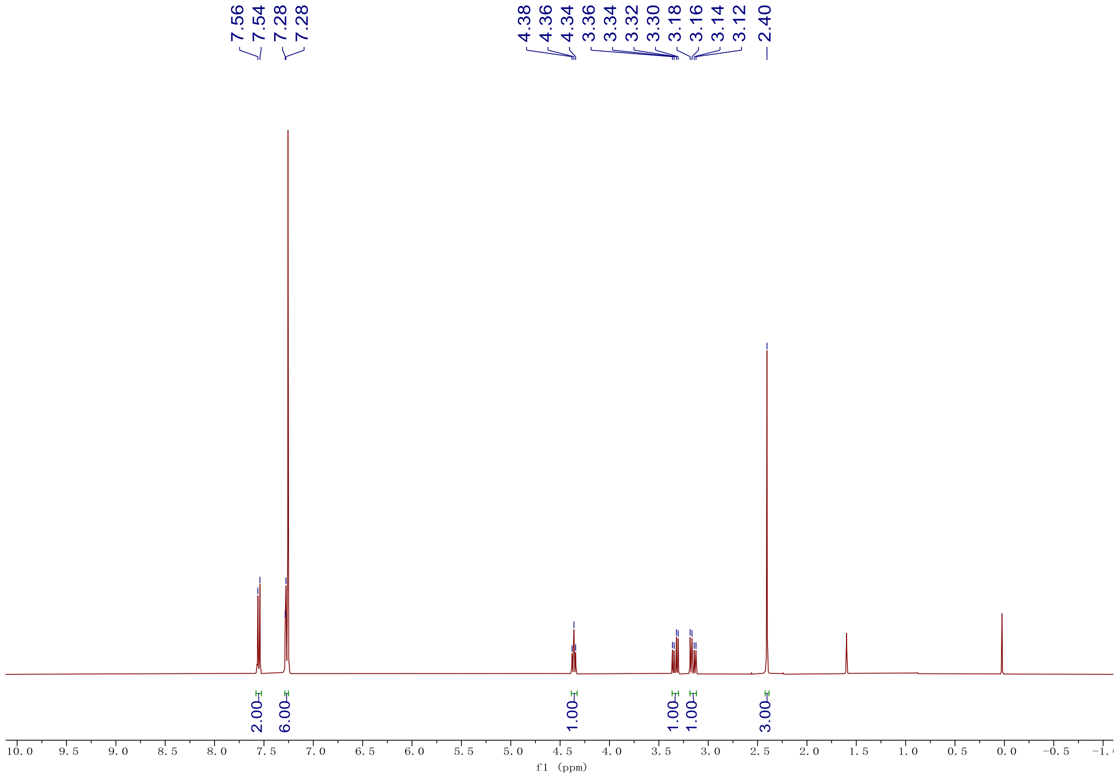

Supplementary Figure 69. ^{1}H NMR (400 MHz, $CDCl_3$)

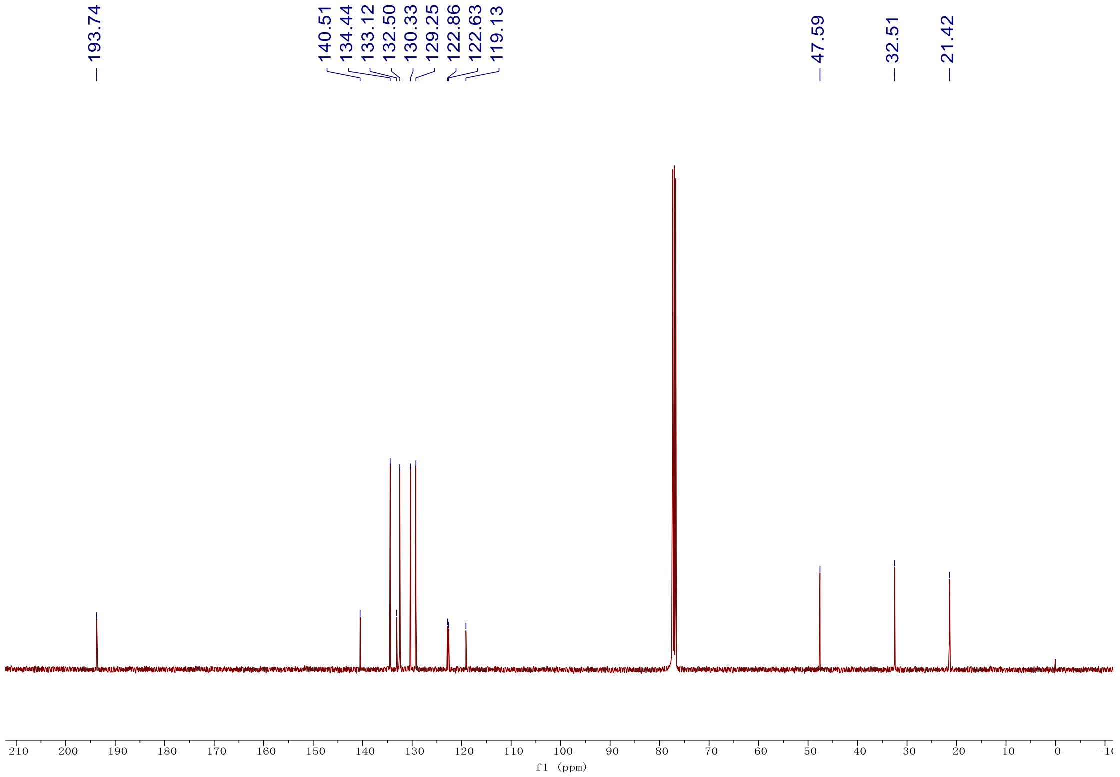

Supplementary Figure 70. ^{13}C NMR (101 MHz, $CDCl_3$)

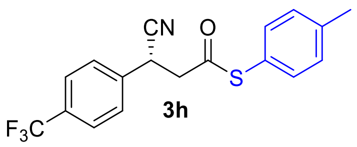

3h

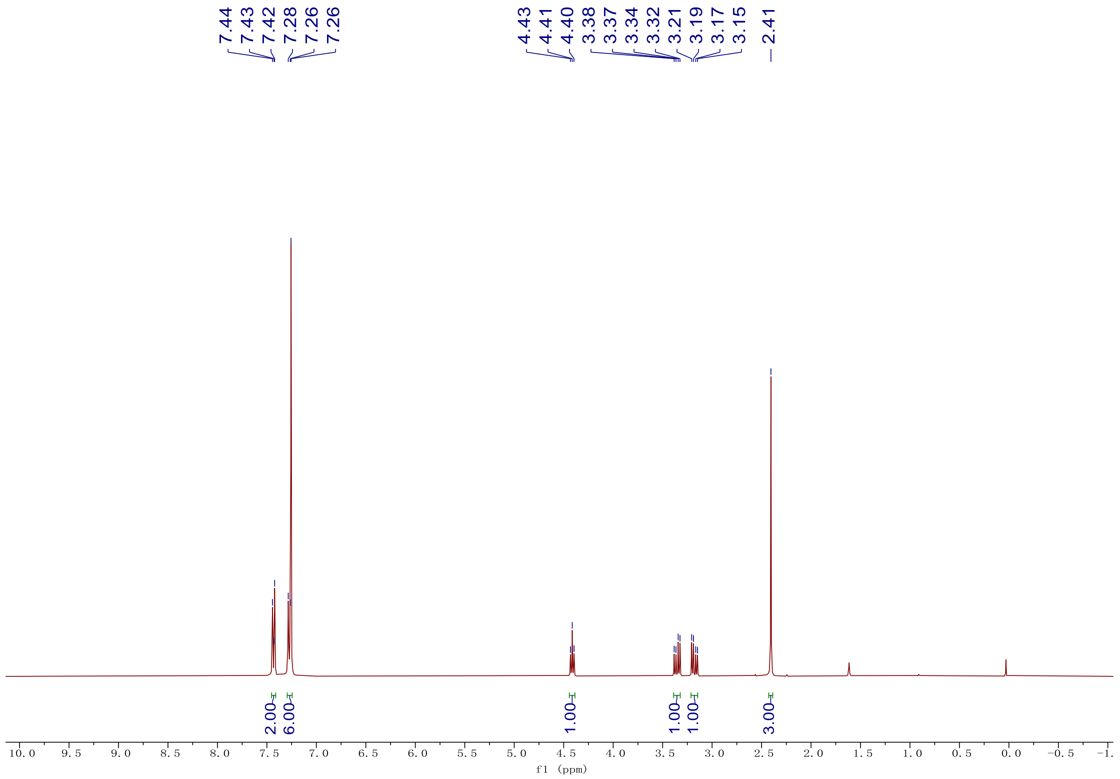

Supplementary Figure 71. ^{1}H NMR (400 MHz, $CDCl_3$)

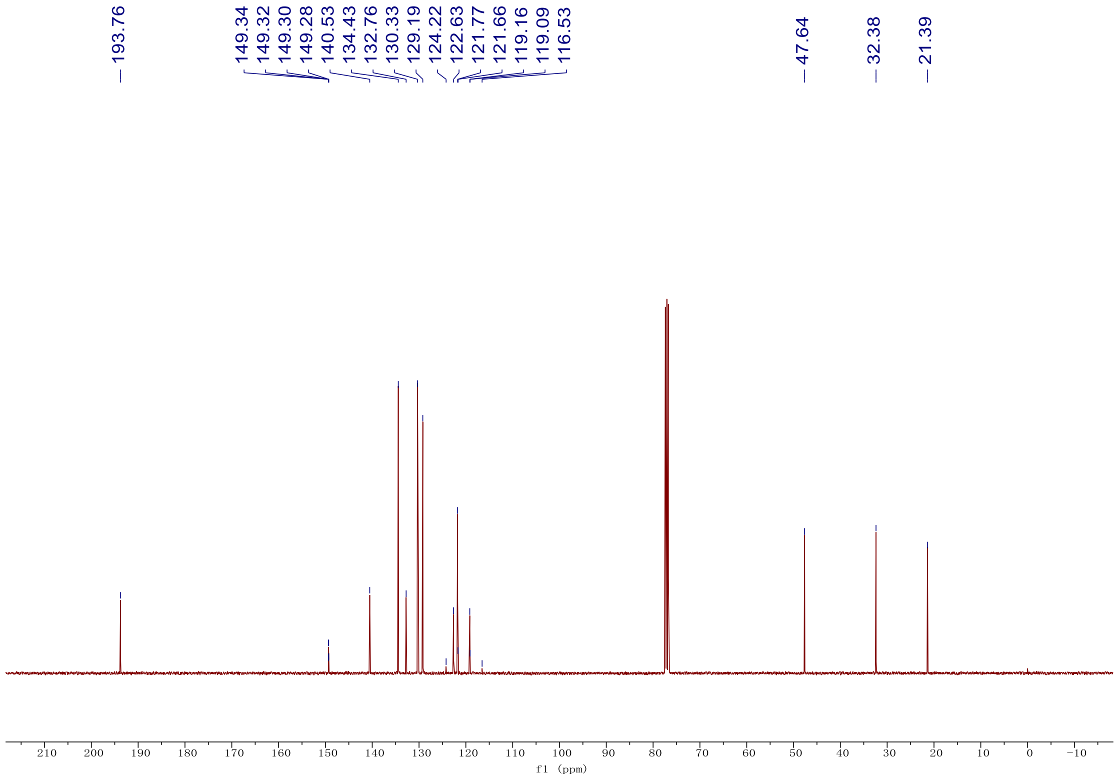

Supplementary Figure 72. ^{13}C NMR (101 MHz, $CDCl_3$)

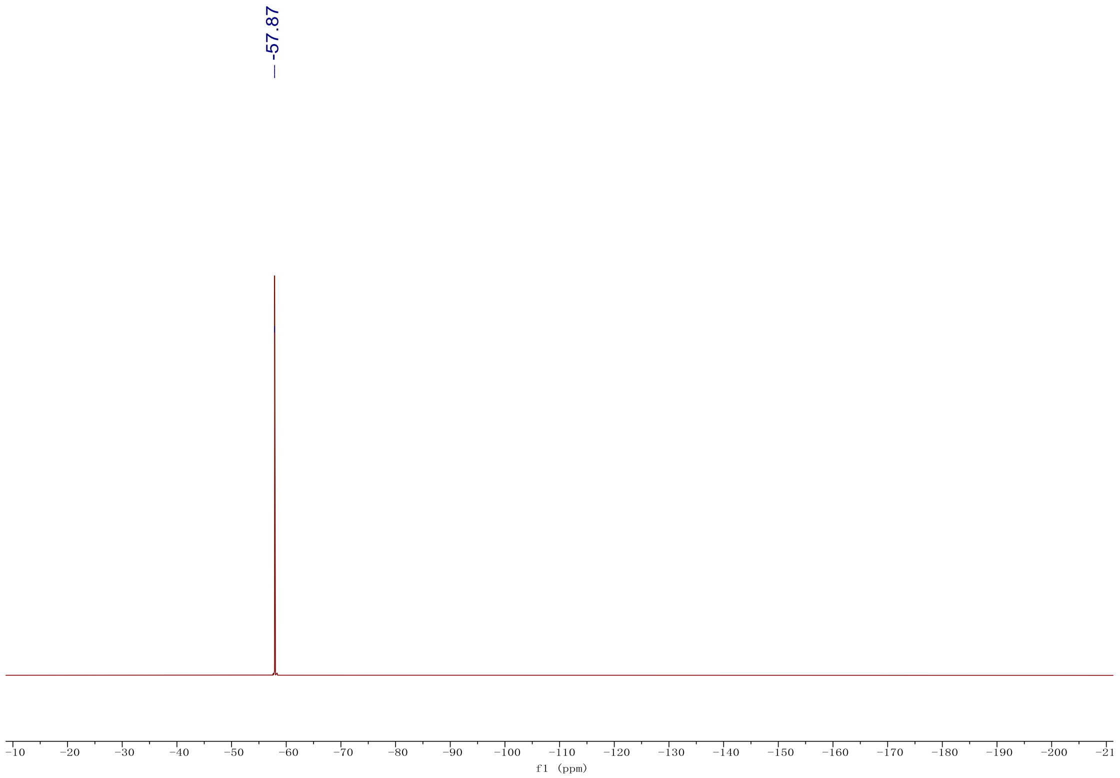

Supplementary Figure 73. ^{19}F NMR (376 MHz, $CDCl_3$)

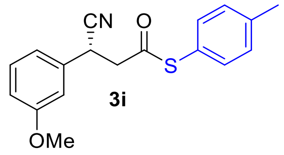

3i

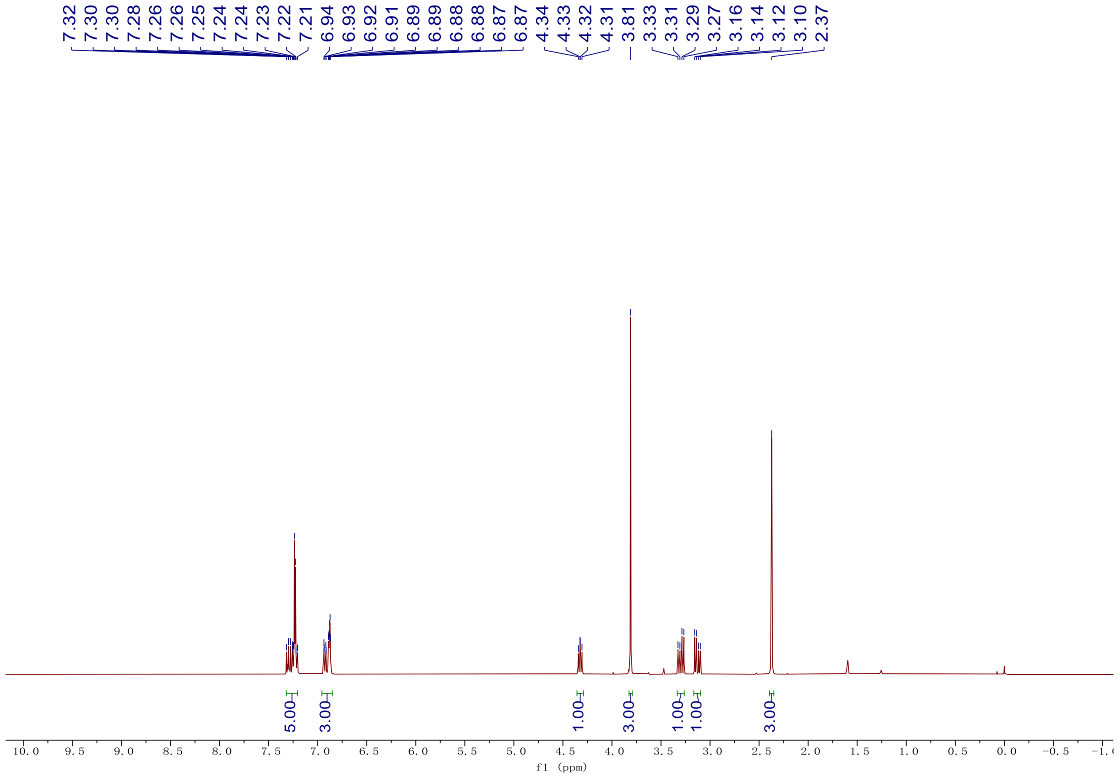

Supplementary Figure 74. ^{1}H NMR (400 MHz, $CDCl_3$)

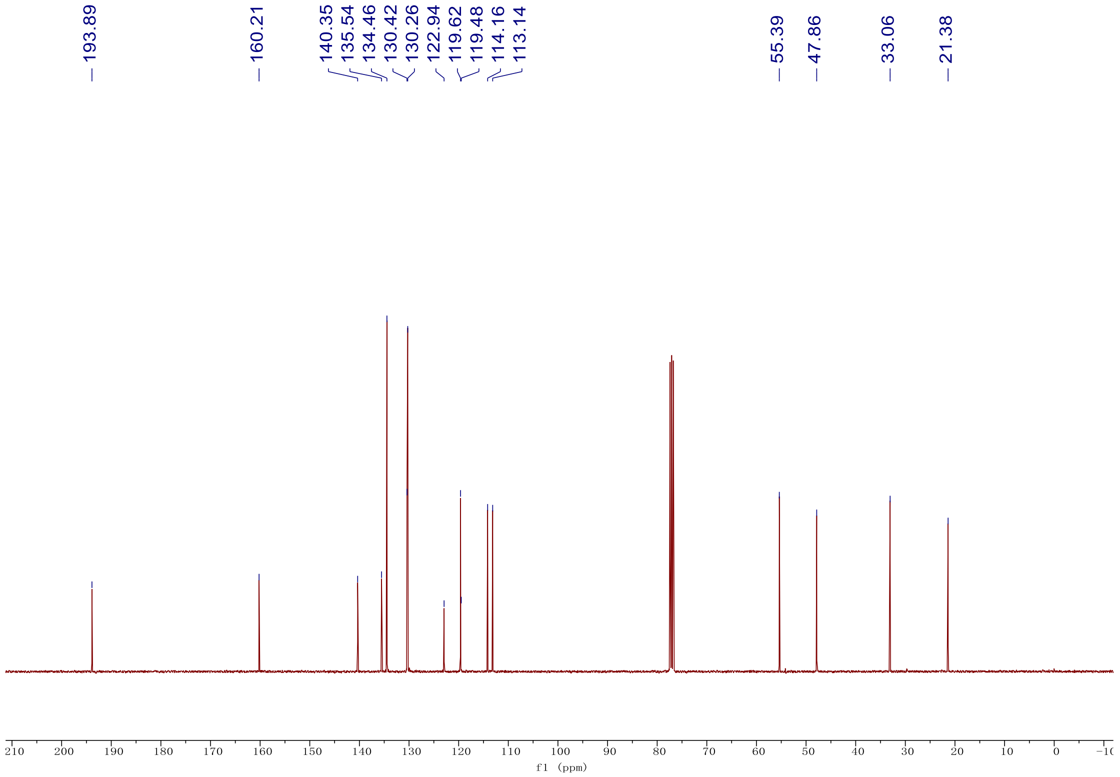

Supplementary Figure 75. ^{13}C NMR (101 MHz, $CDCl_3$)

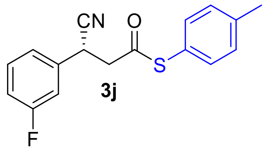

3j

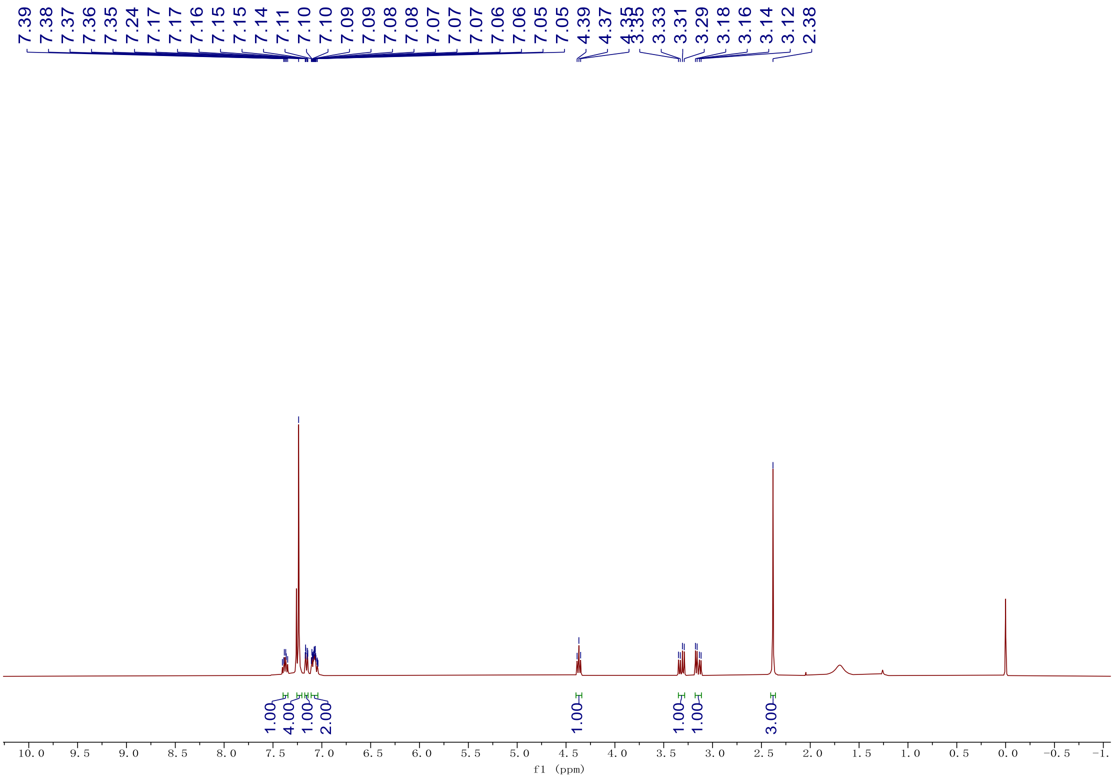

Supplementary Figure 76. ^{1}H NMR (400 MHz, CDCl$_3$)

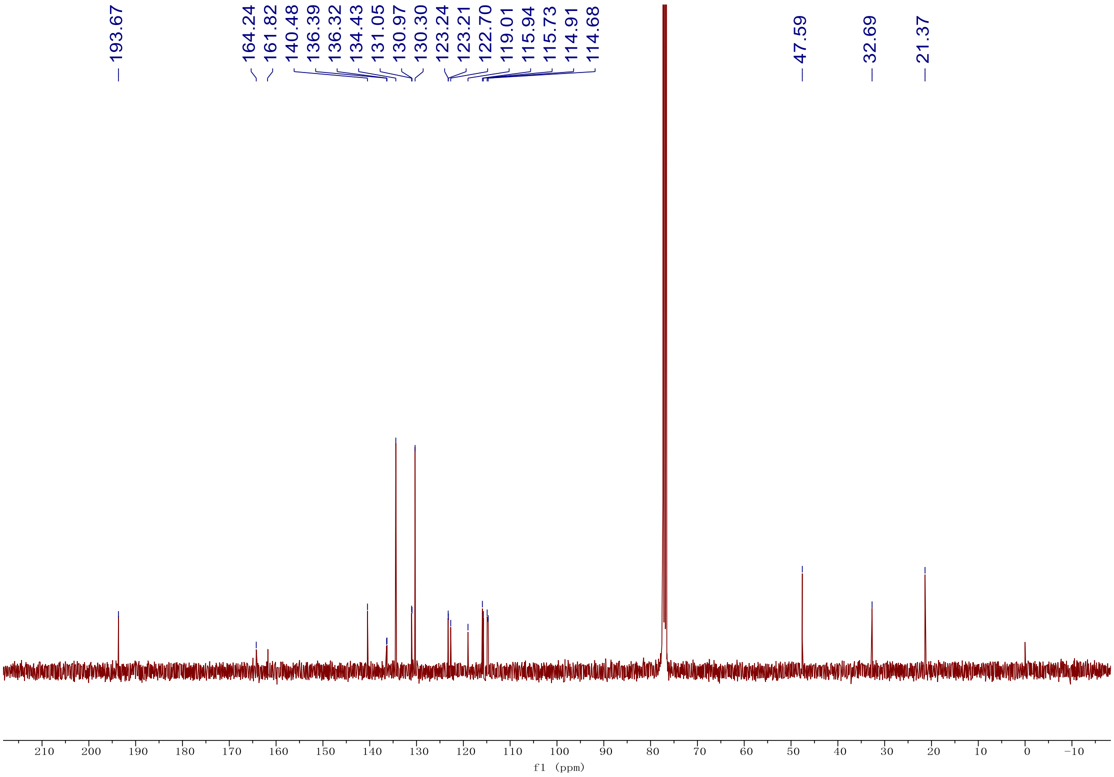

Supplementary Figure 77. ^{13}C NMR (101 MHz, CDCl$_3$)

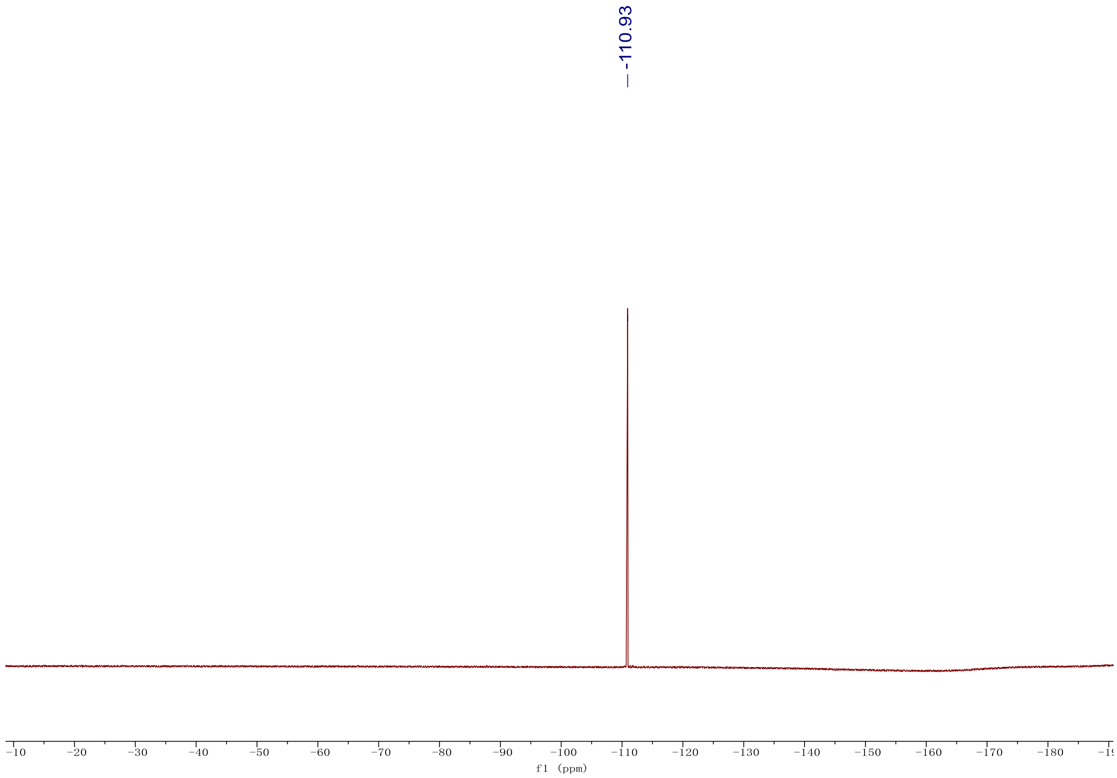

Supplementary Figure 78. ^{19}F NMR (376 MHz, $CDCl_3$)

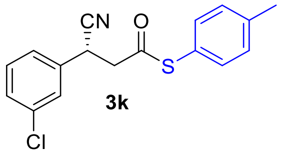

3k

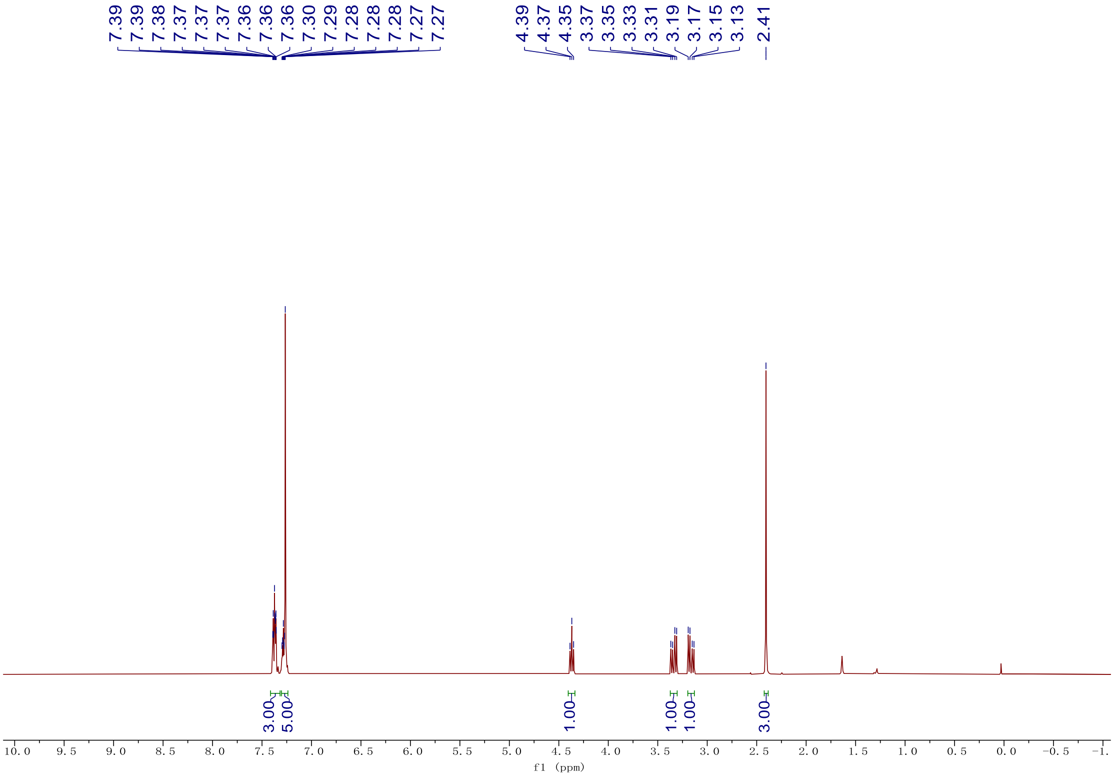

Supplementary Figure 79. ^{1}H NMR (400 MHz, CDCl$_3$)

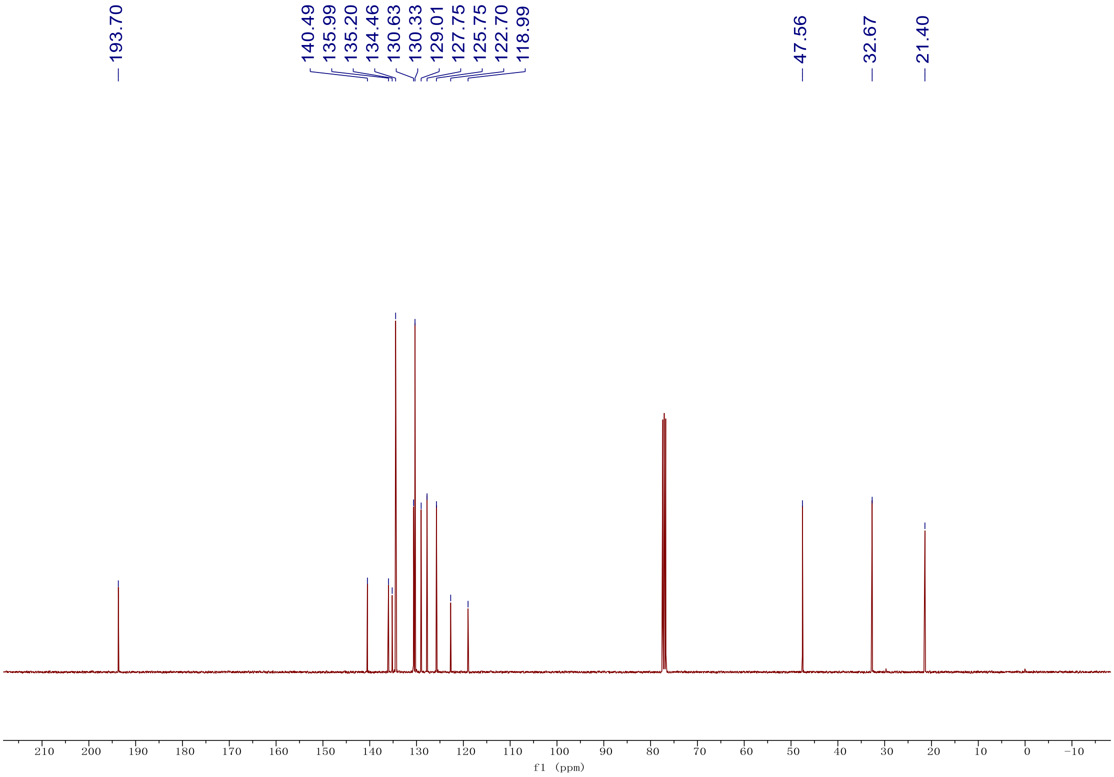

Supplementary Figure 80. ^{13}C NMR (101 MHz, CDCl$_3$)

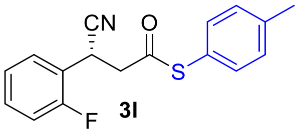

3l

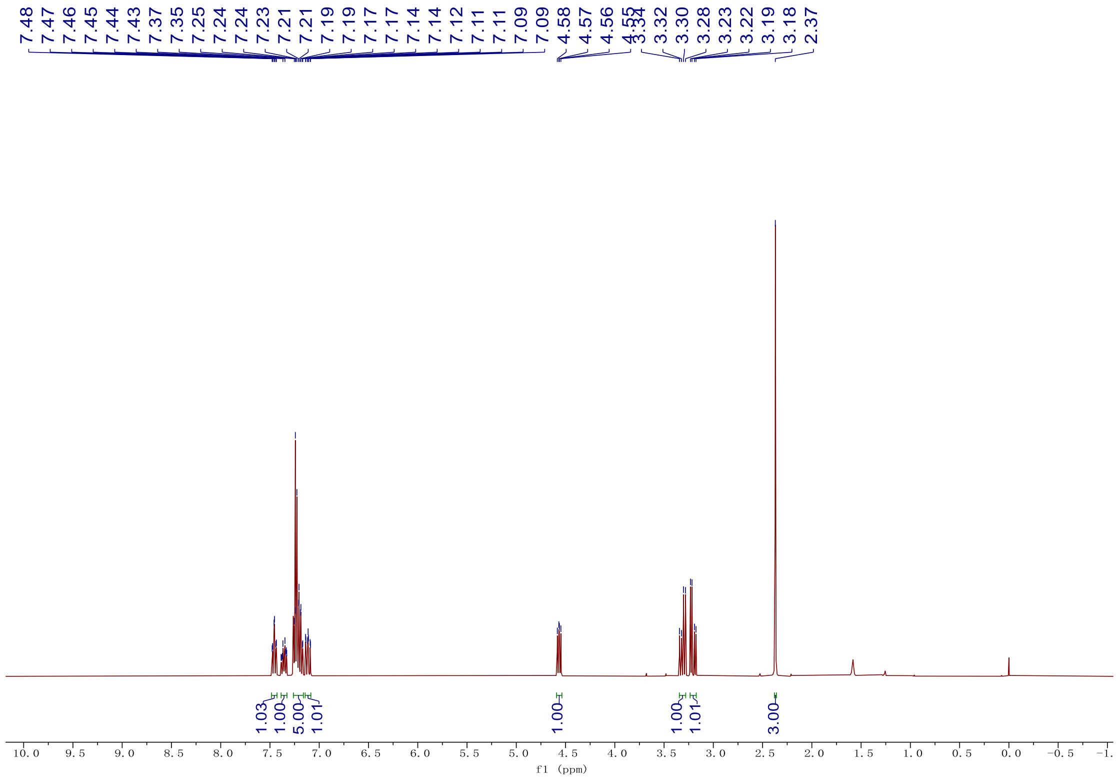

Supplementary Figure 81. ^{1}H NMR (400 MHz, $CDCl_3$)

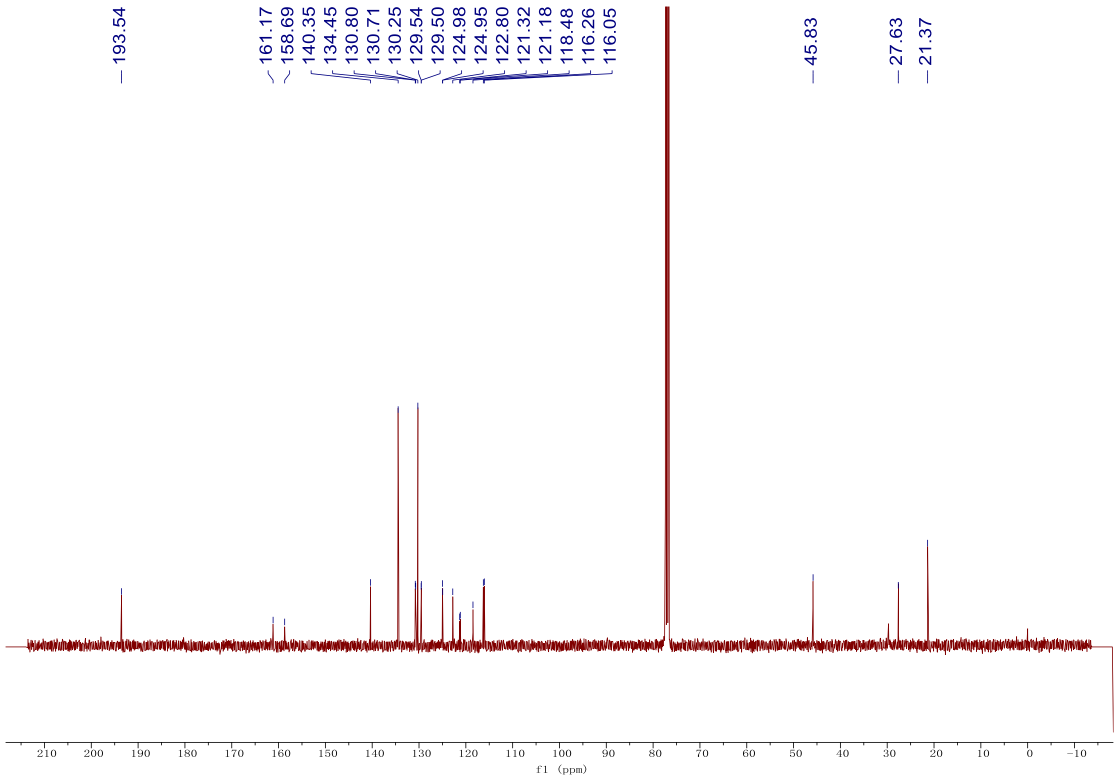

Supplementary Figure 82. ^{13}C NMR (101 MHz, $CDCl_3$)

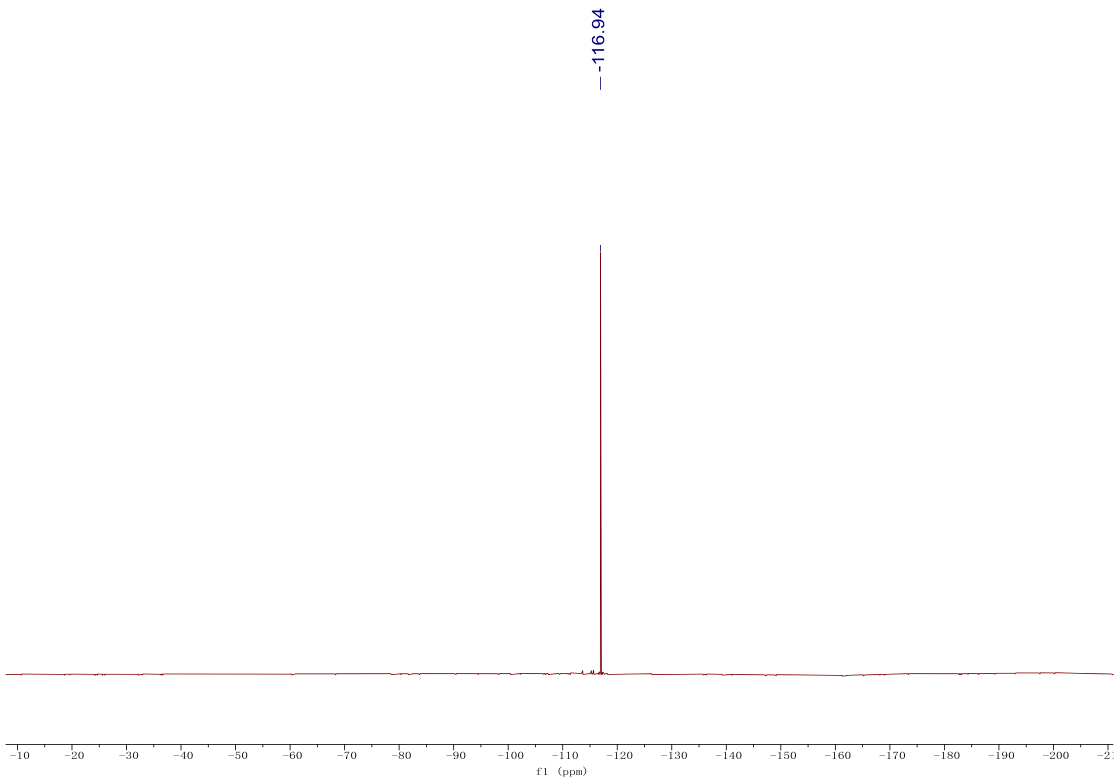

Supplementary Figure 83. ^{19}F NMR (376 MHz, $CDCl_3$)

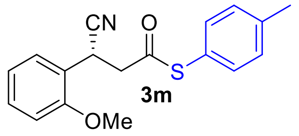

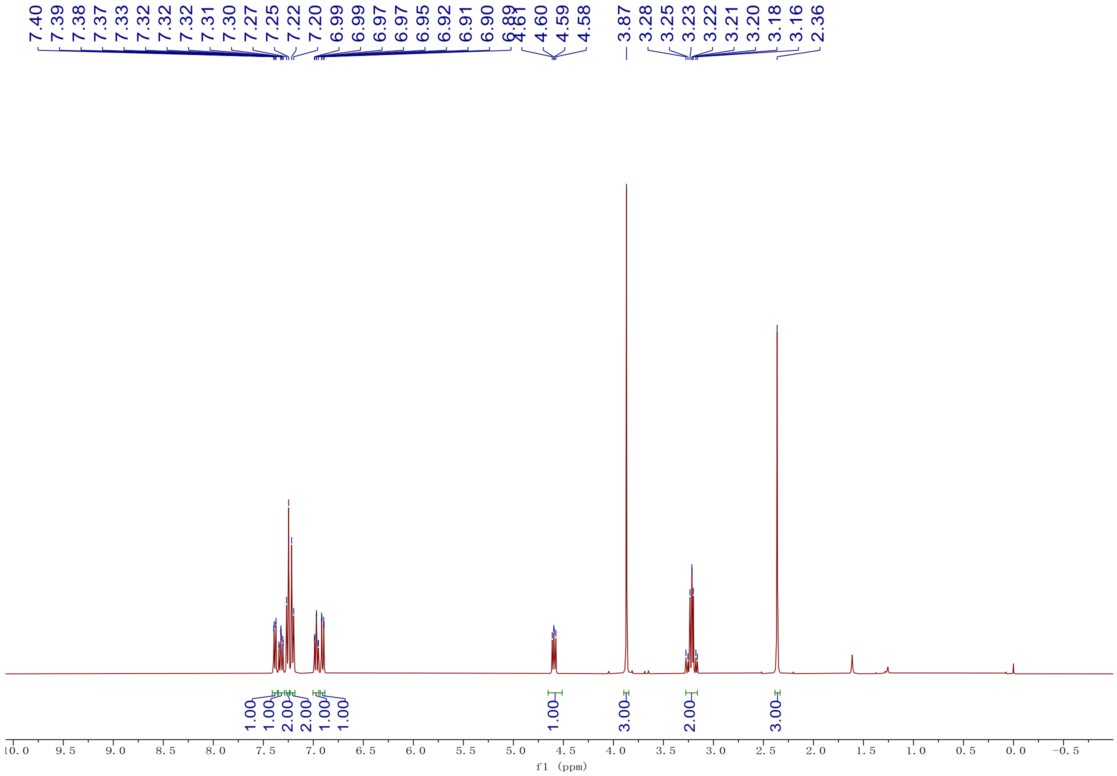

Supplementary Figure 84. ^{1}H NMR (400 MHz, CDCl$_3$)

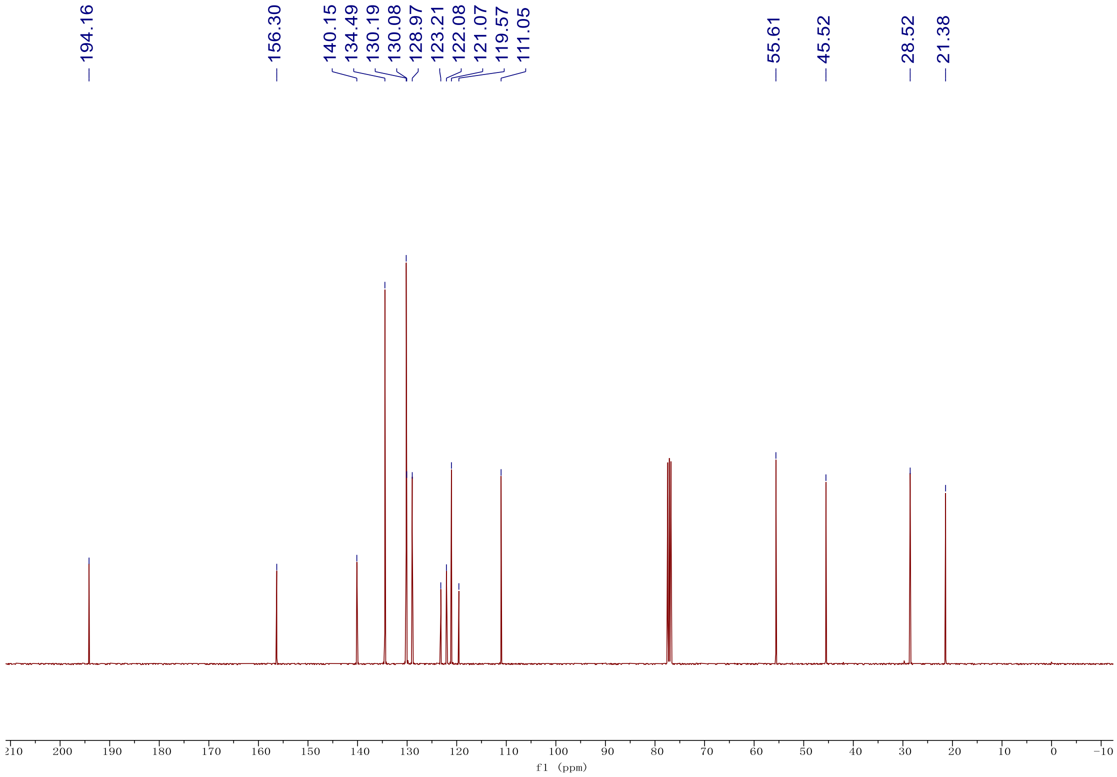

Supplementary Figure 85. ^{13}C NMR (101 MHz, CDCl$_3$)

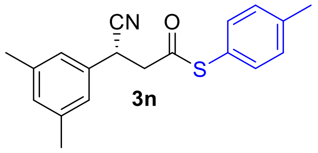

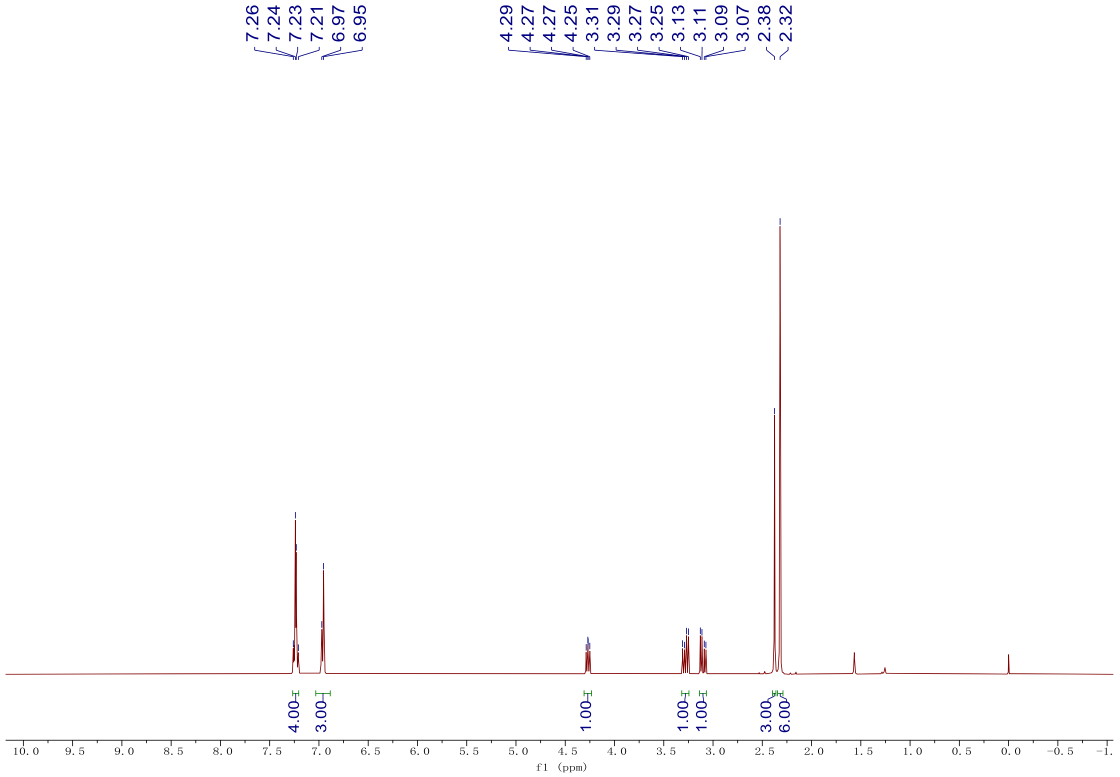

Supplementary Figure 86. ^{1}H NMR (400 MHz, CDCl$_3$)

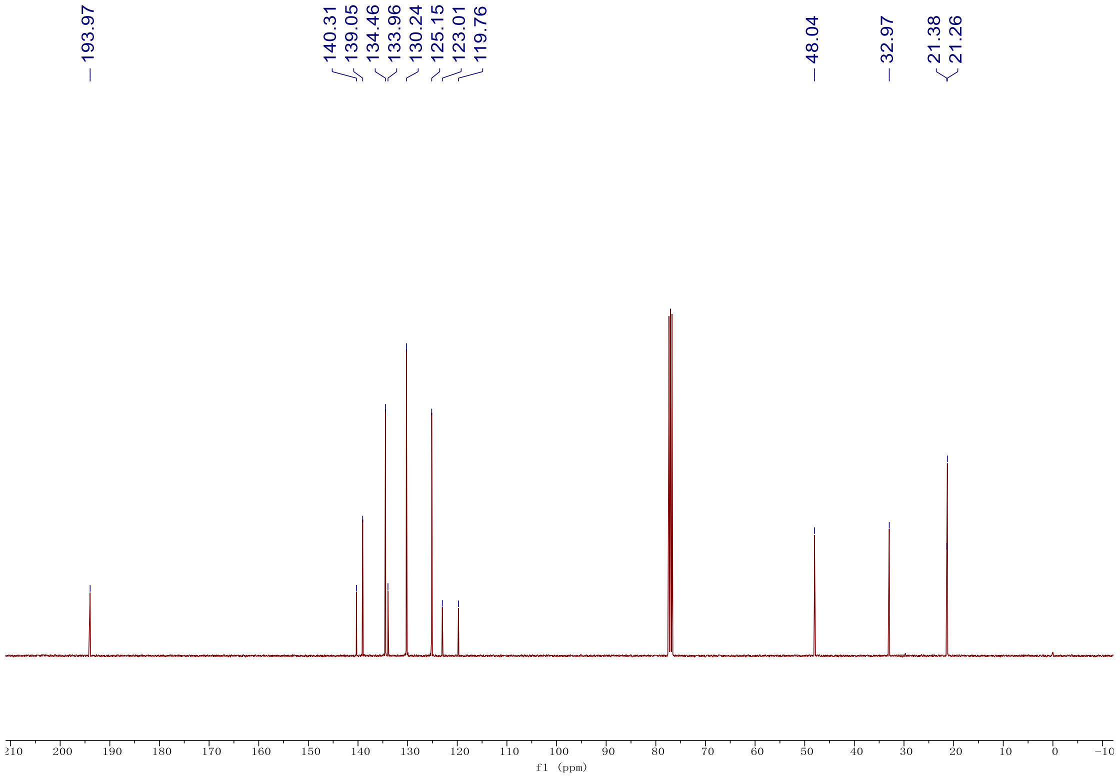

Supplementary Figure 87. ^{13}C NMR (101 MHz, CDCl$_3$)

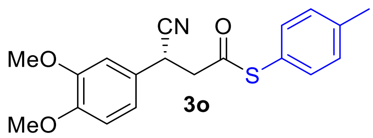

3o

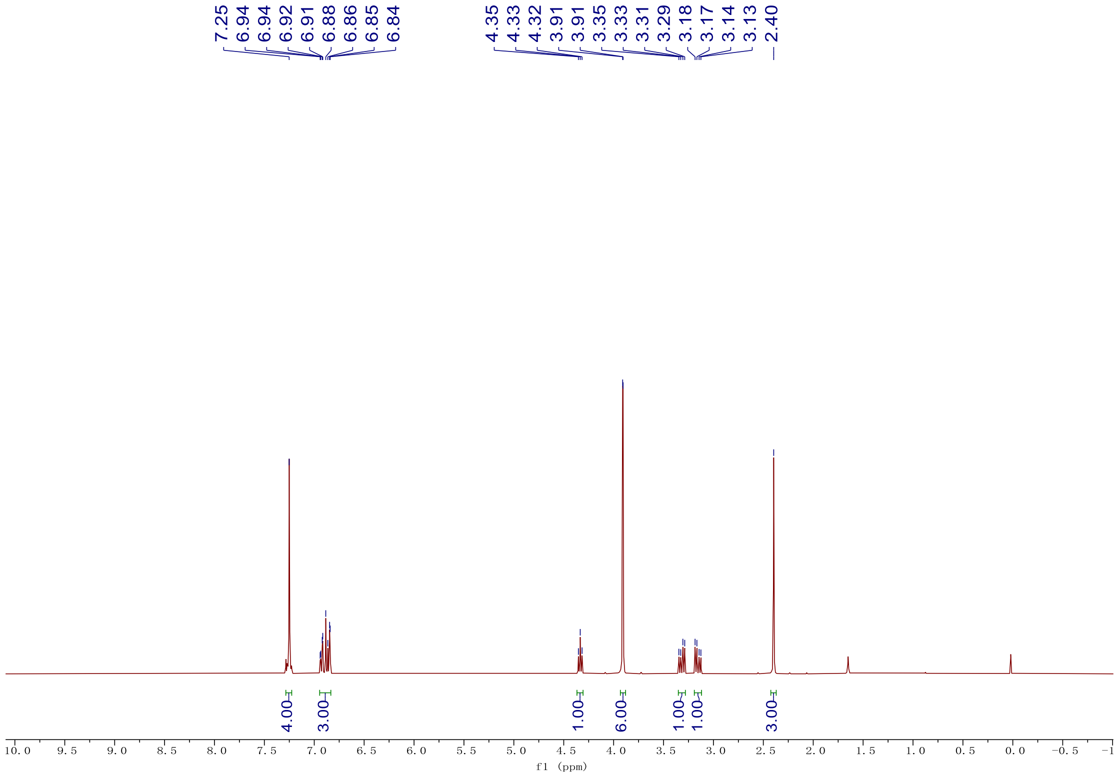

Supplementary Figure 88. ^{1}H NMR (400 MHz, CDCl$_3$)

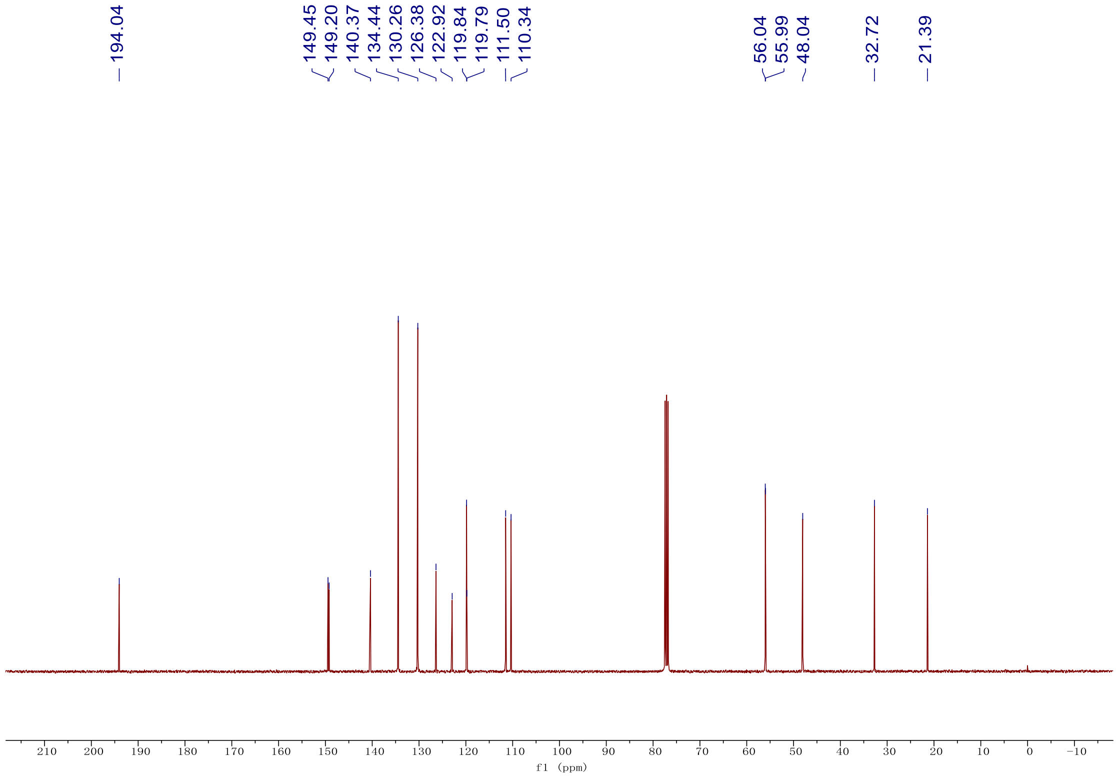

Supplementary Figure 89. ^{13}C NMR (101 MHz, CDCl$_3$)

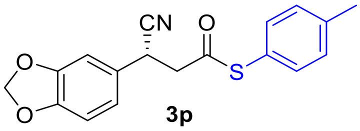

3p

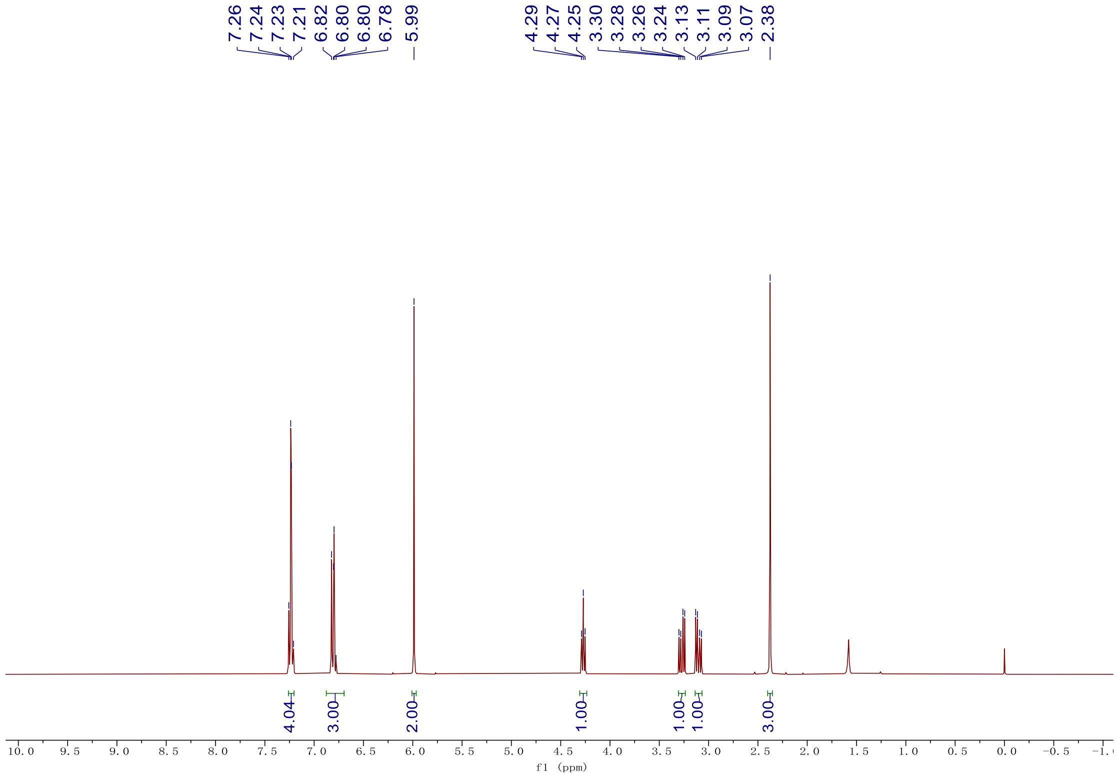

Supplementary Figure 90. ^{1}H NMR (400 MHz, $CDCl_3$)

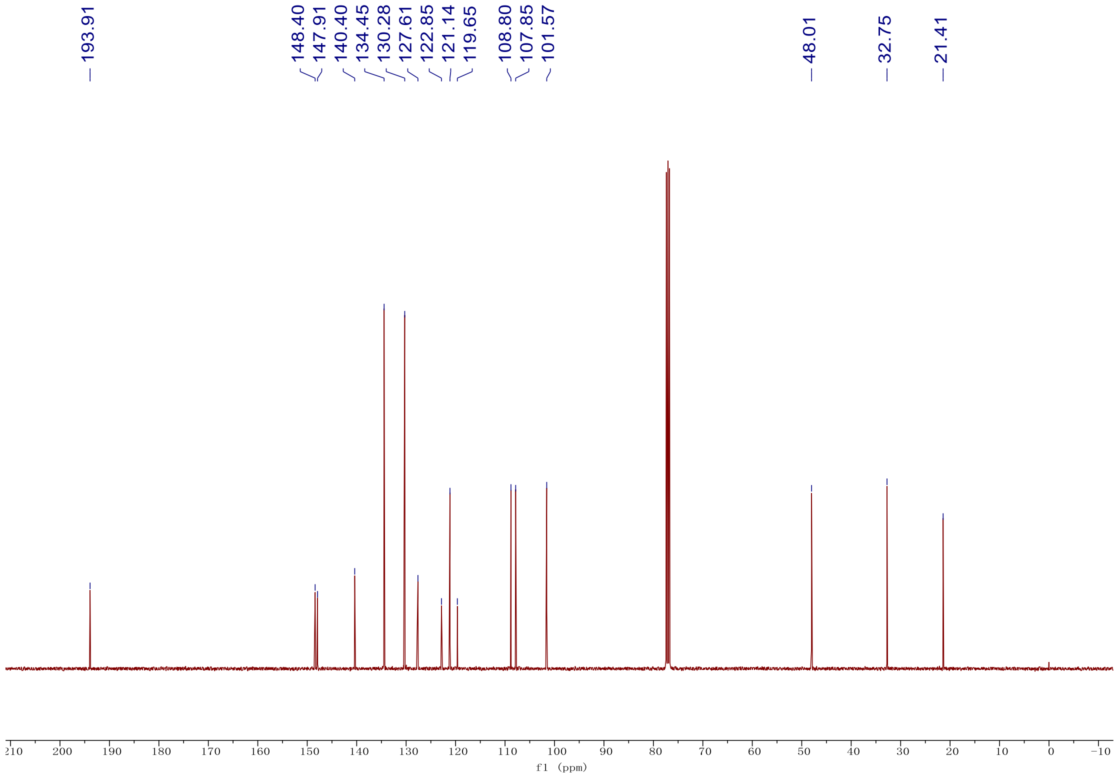

Supplementary Figure 91. ^{13}C NMR (101 MHz, $CDCl_3$)

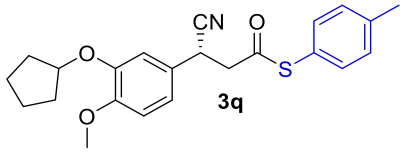

3q

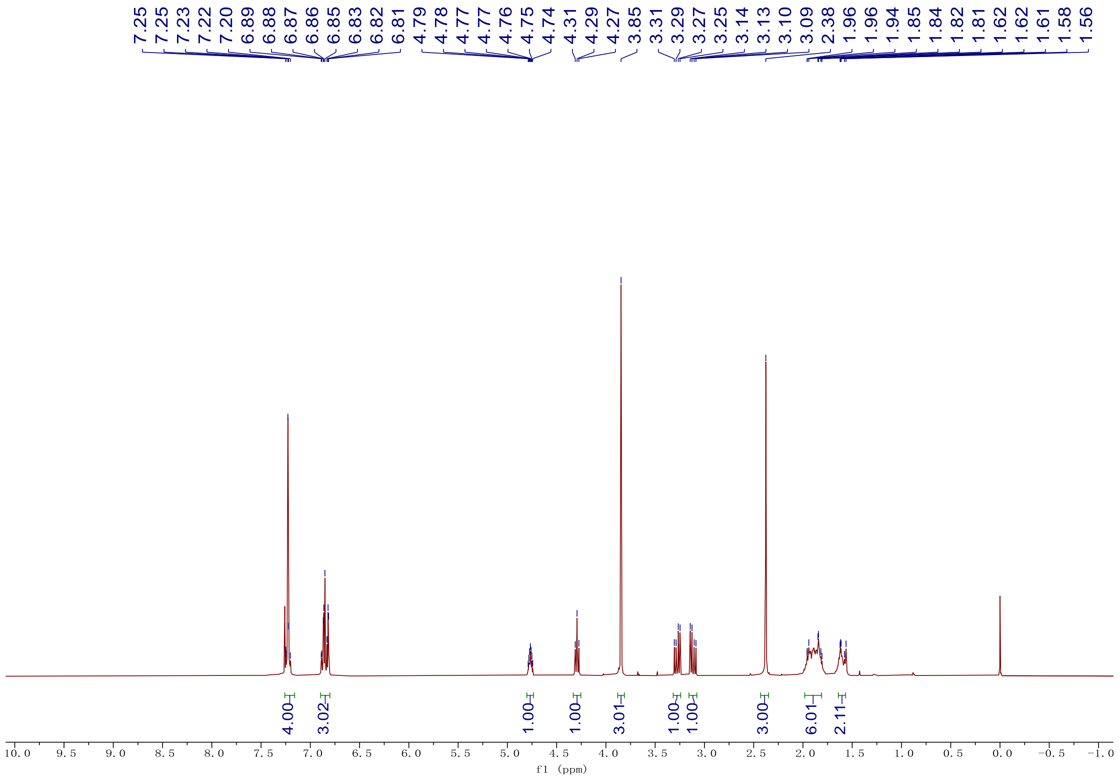

Supplementary Figure 92. ^{1}H NMR (400 MHz, $CDCl_3$)

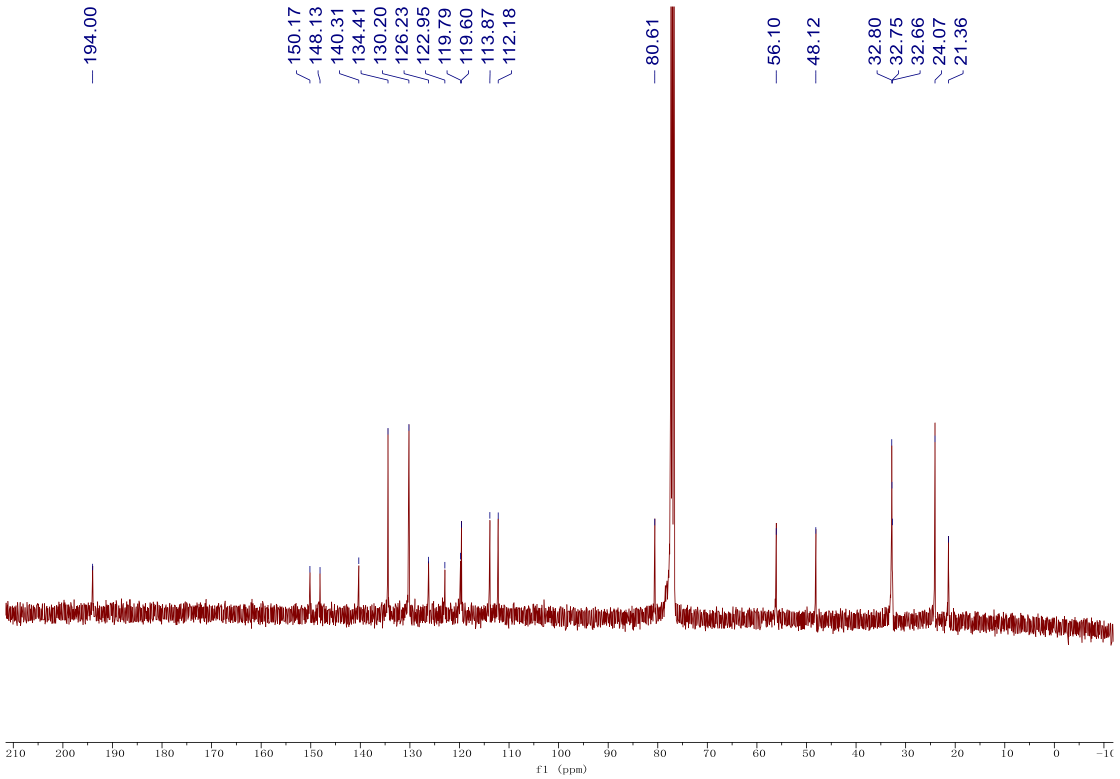

Supplementary Figure 93. ^{13}C NMR (101 MHz, $CDCl_3$)

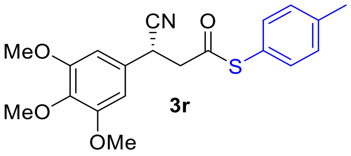

3r

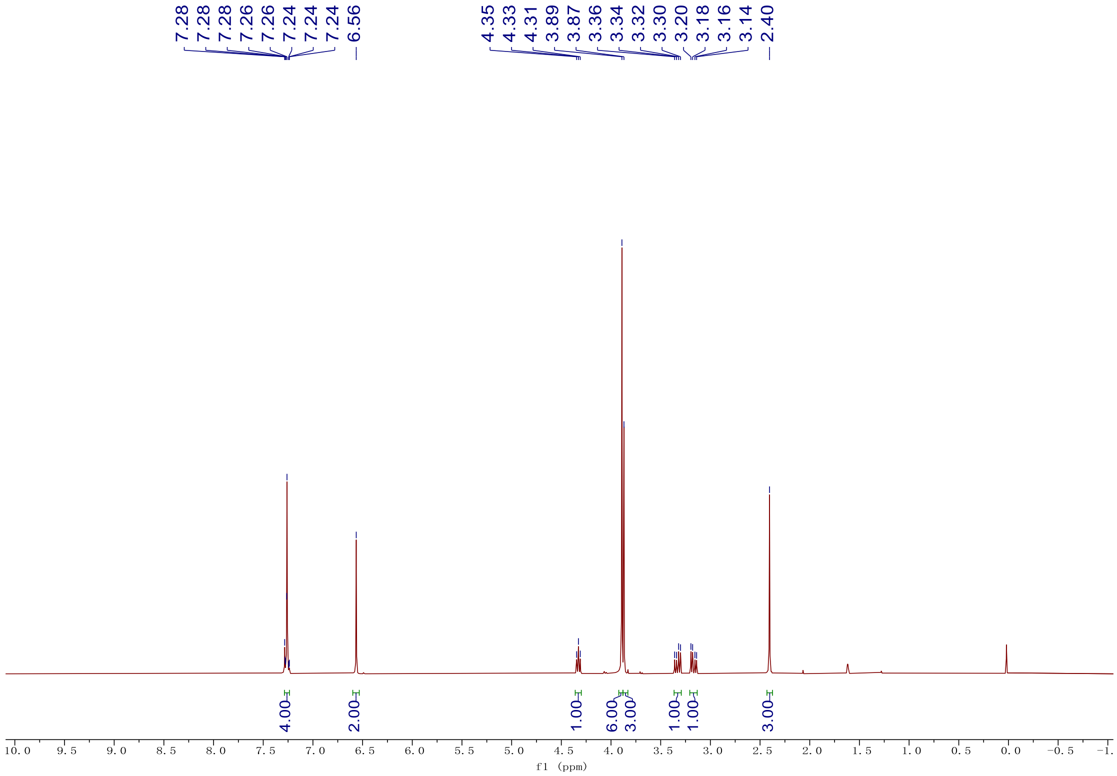

Supplementary Figure 94. ^{1}H NMR (400 MHz, $CDCl_3$)

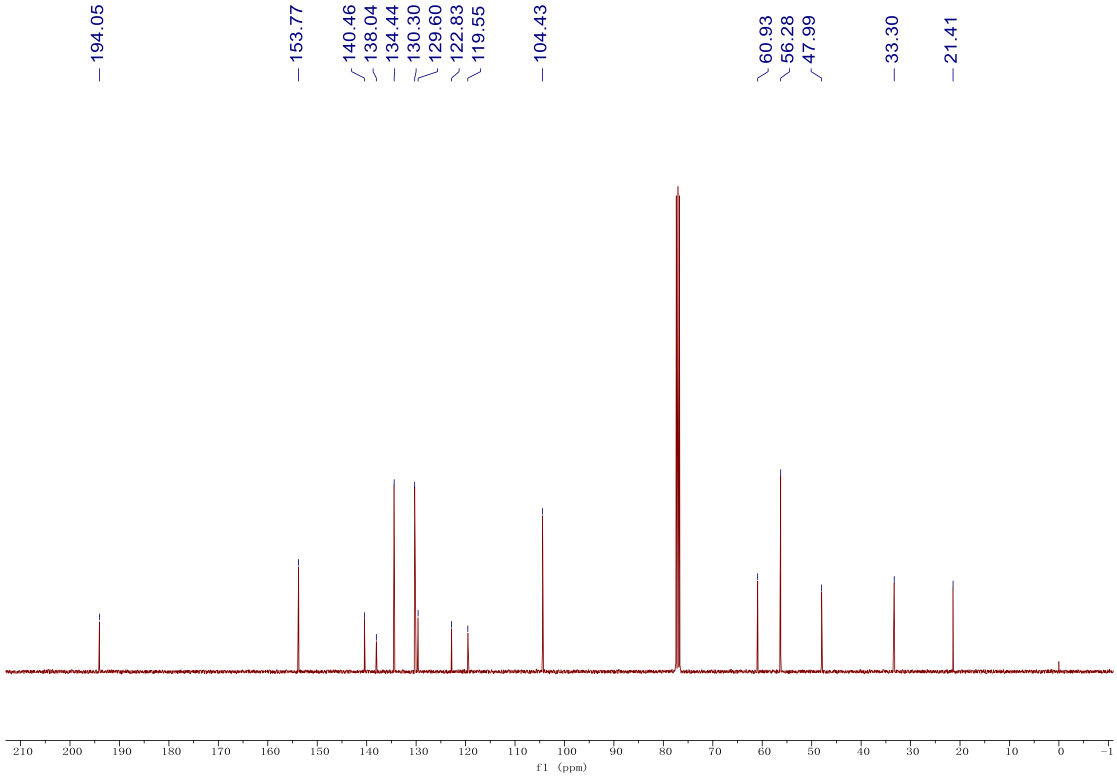

Supplementary Figure 95. ^{13}C NMR (101 MHz, $CDCl_3$)

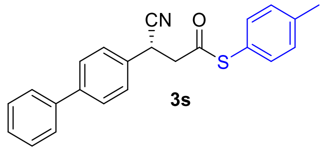

3s

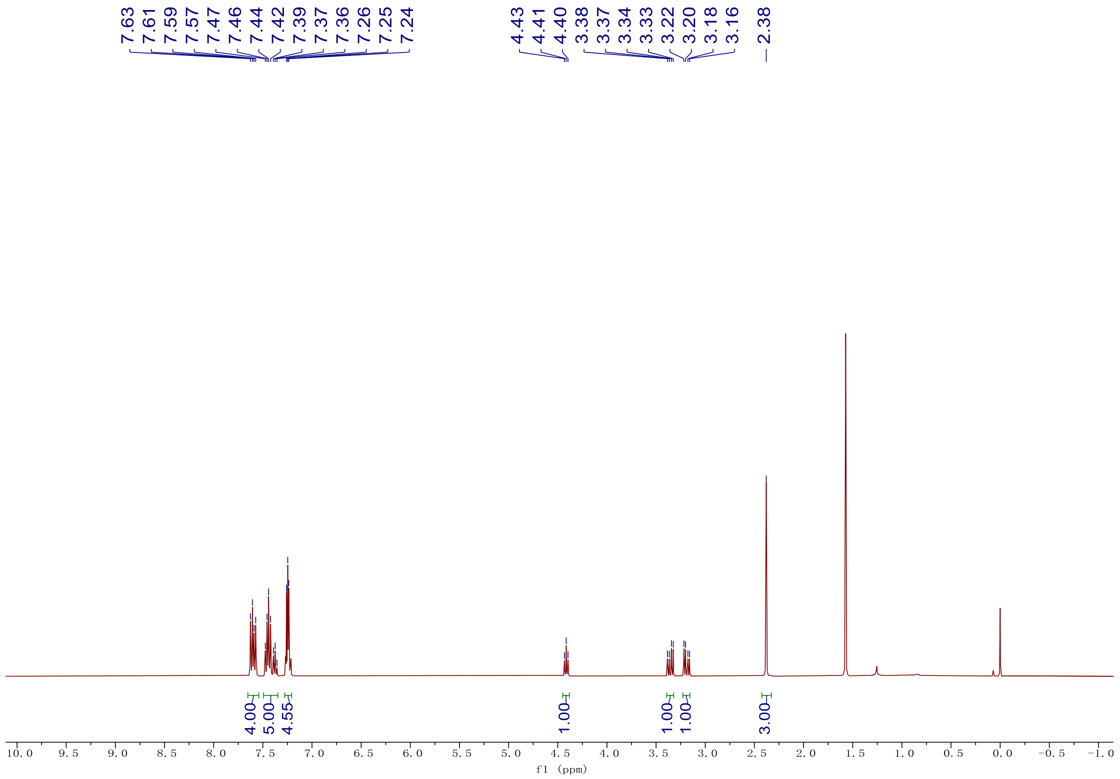

Supplementary Figure 96. ¹H NMR (400 MHz, CDCl₃)

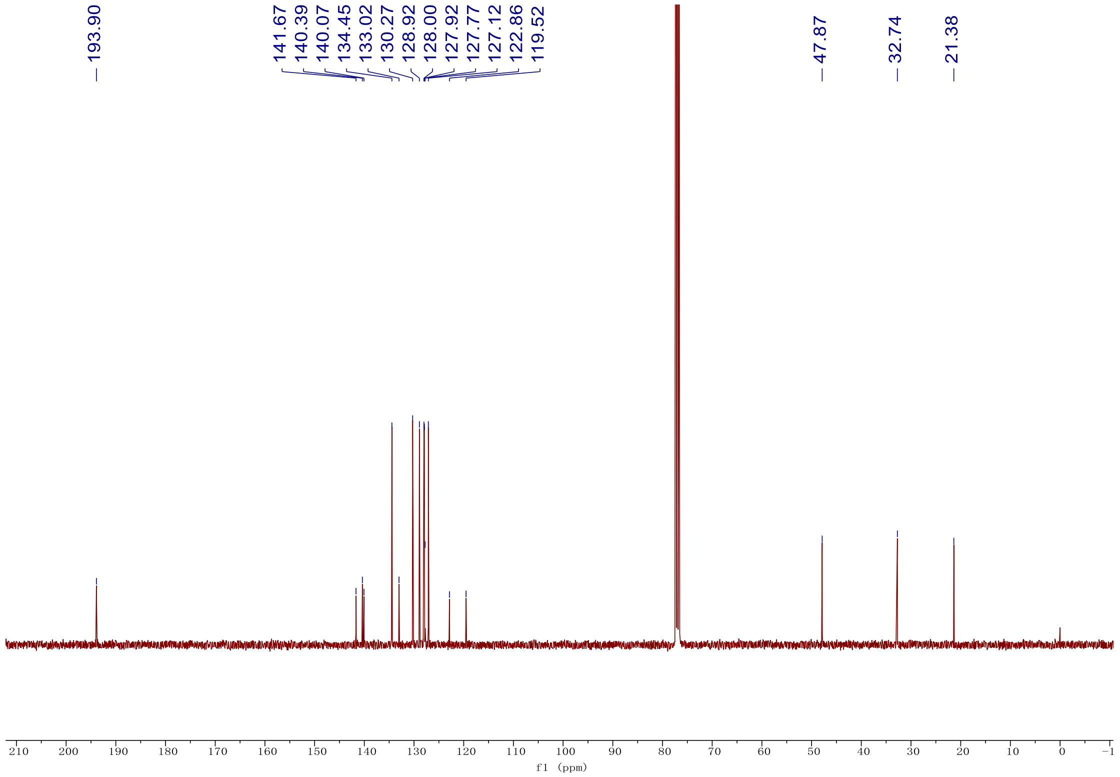

Supplementary Figure 97. ^{13}C NMR (101 MHz, $CDCl_3$)

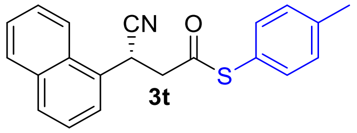

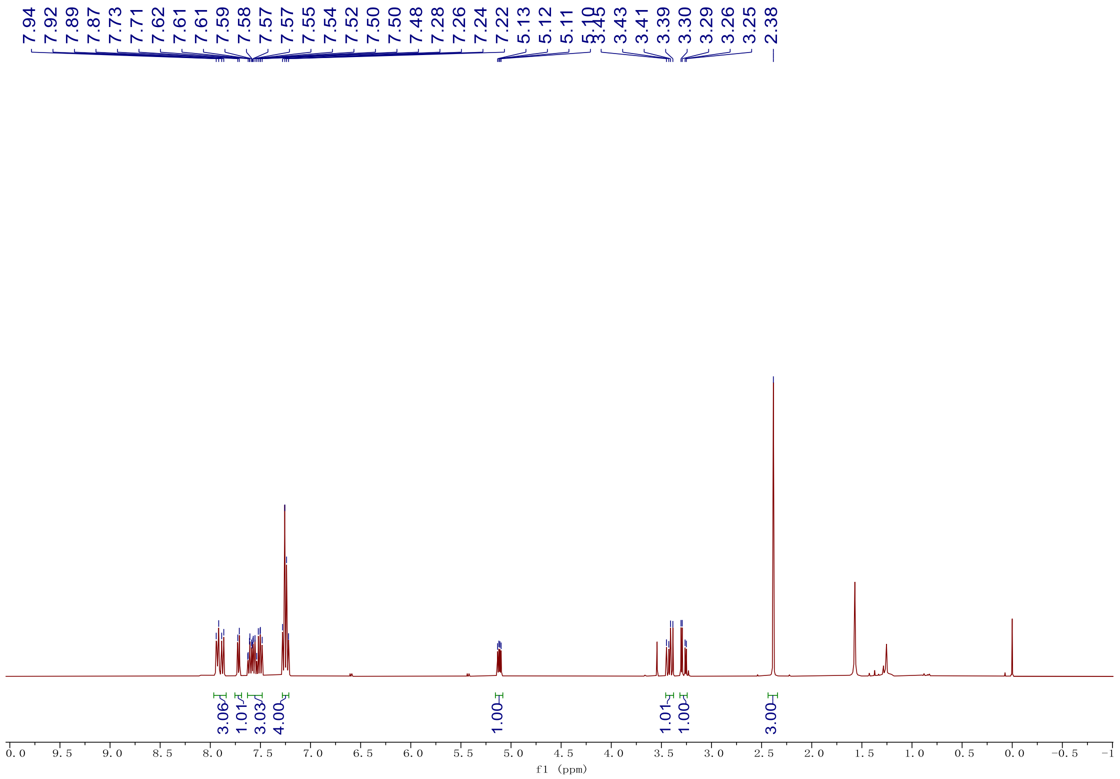

Supplementary Figure 98. ^{1}H NMR (400 MHz, CDCl$_3$)

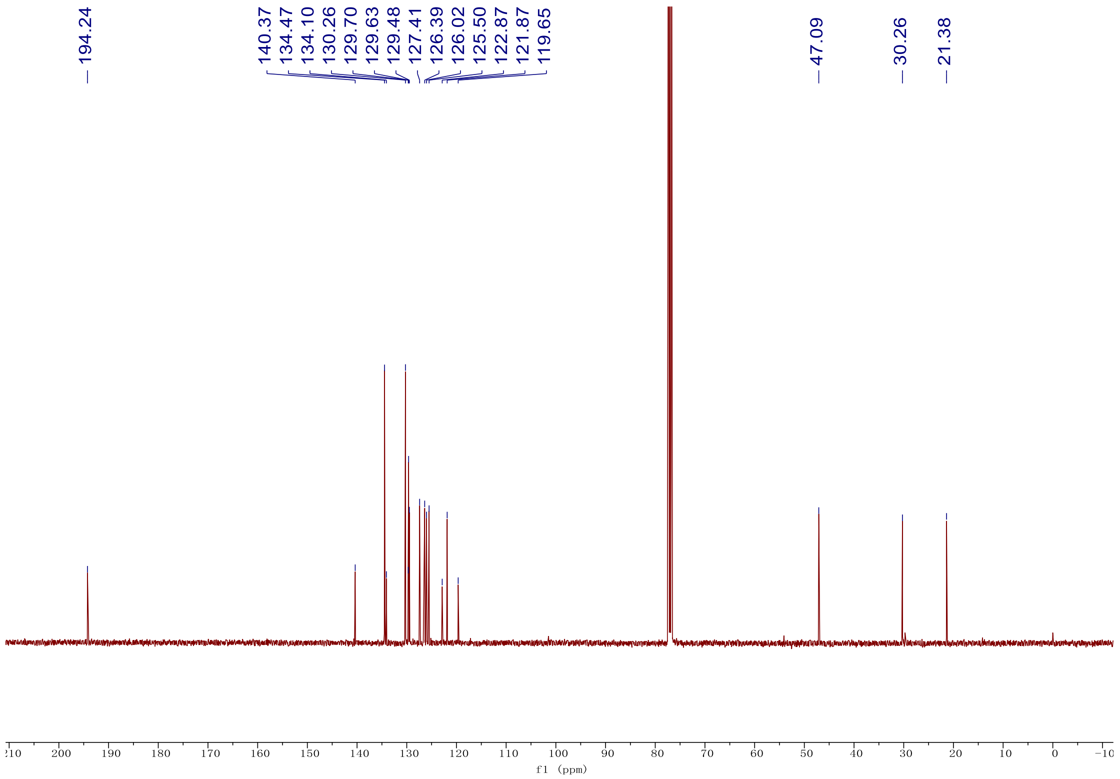

Supplementary Figure 99. ^{13}C NMR (101 MHz, CDCl$_3$)

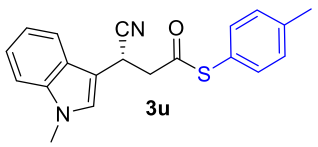

3u

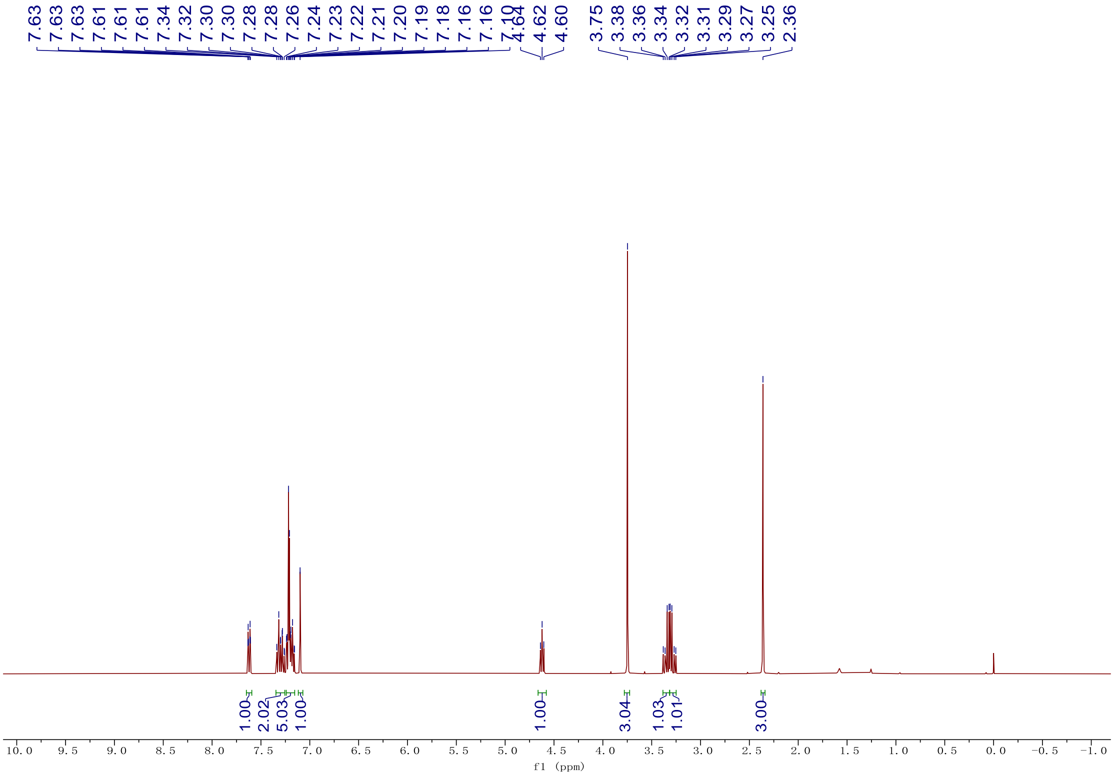

Supplementary Figure 100. ^{1}H NMR (400 MHz, CDCl$_3$)

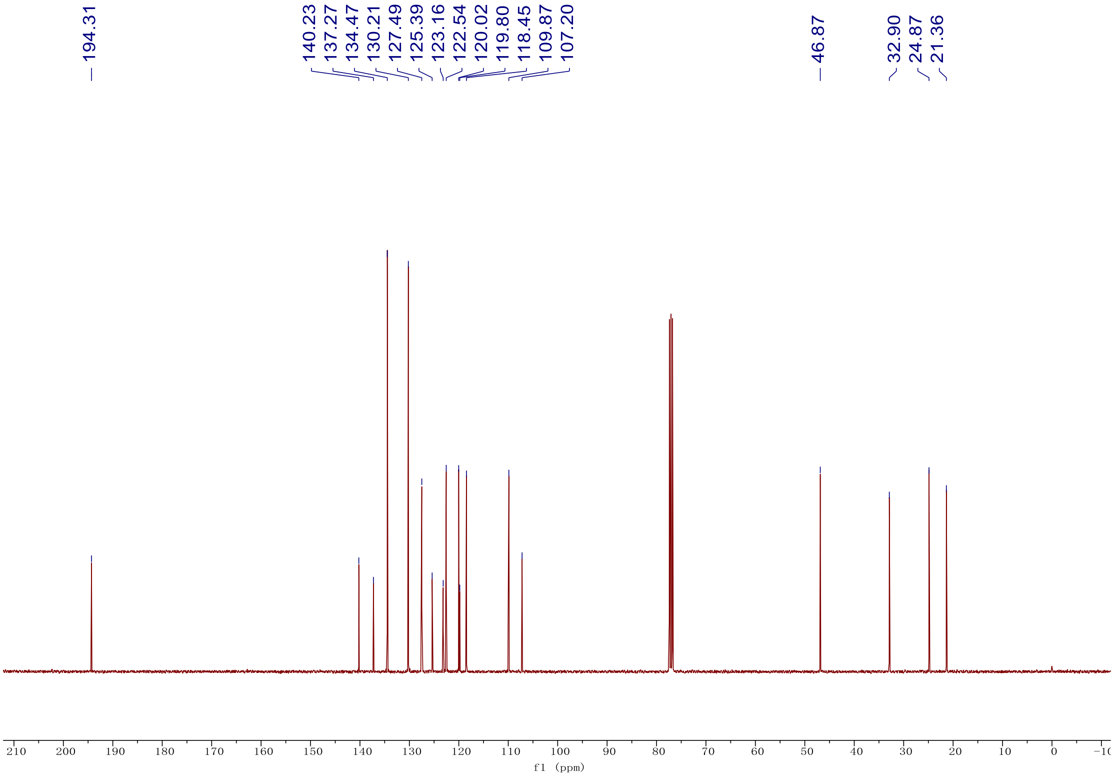

Supplementary Figure 101. ^{13}C NMR (101 MHz, CDCl$_3$)

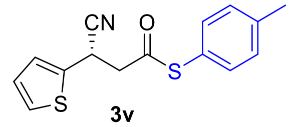

3v

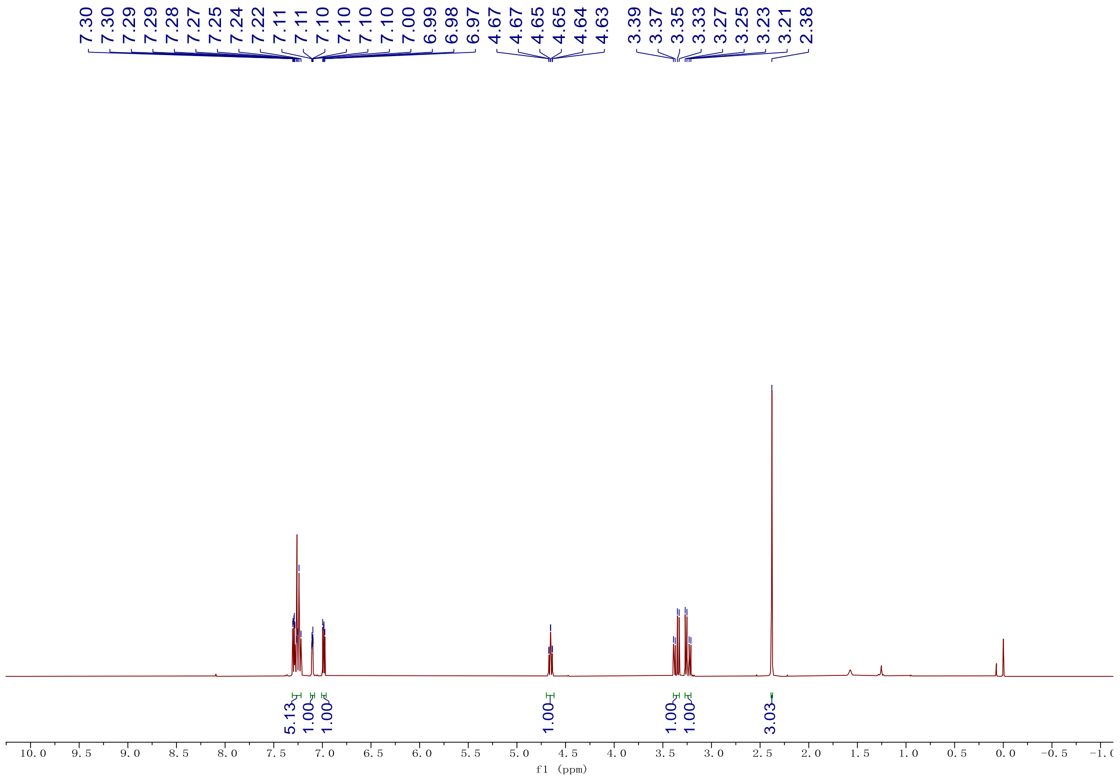

<u>**Supplementary Figure 102. ^{1}H NMR (400 MHz, $CDCl_3$)**</u>

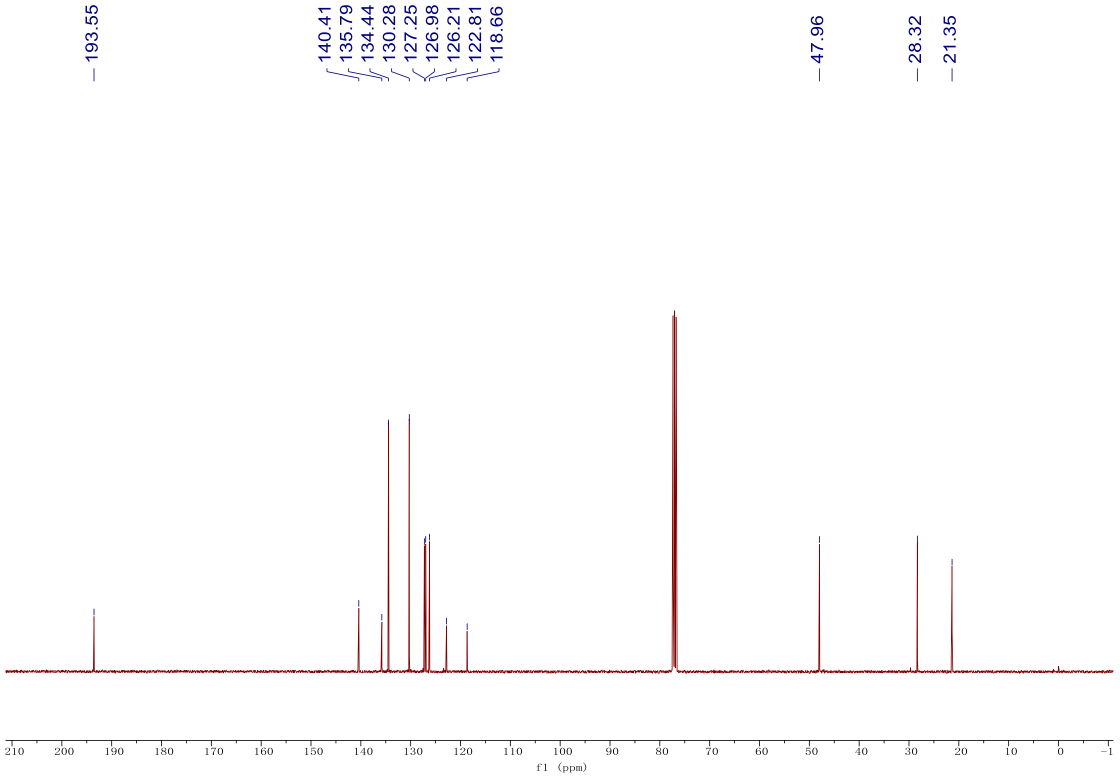

<u>**Supplementary Figure 103. ^{13}C NMR (101 MHz, $CDCl_3$)**</u>

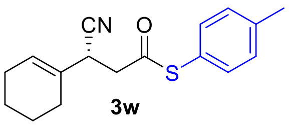

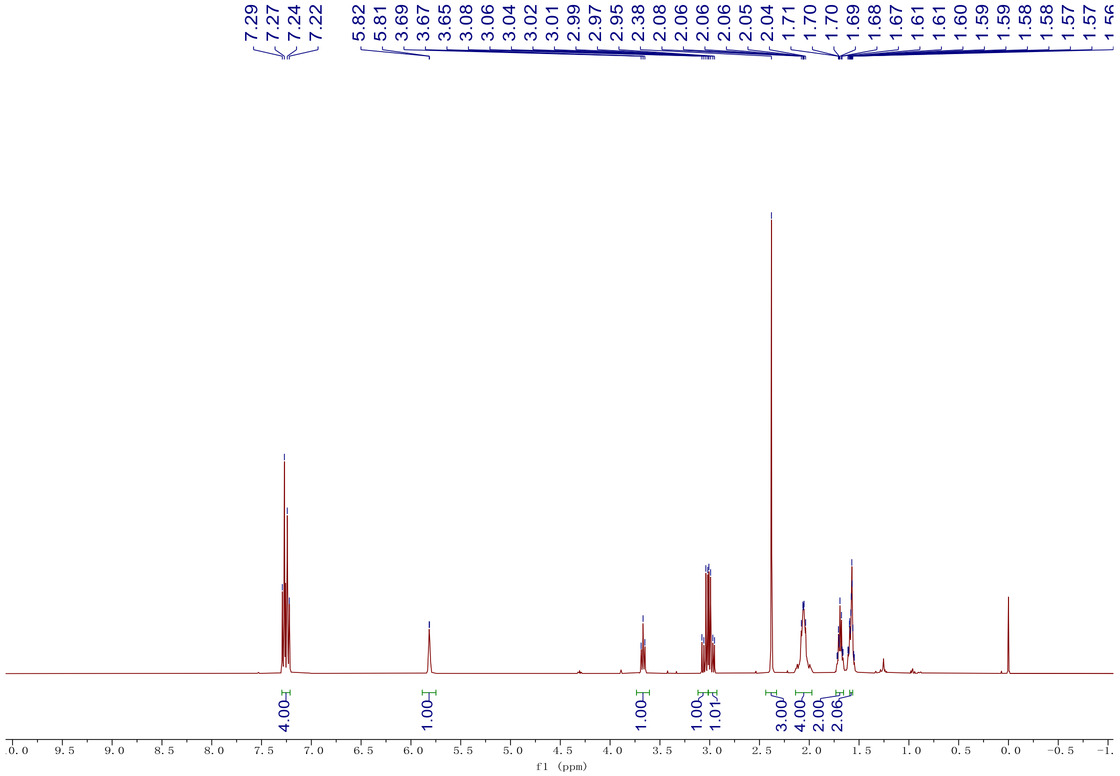

Supplementary Figure 104. ^{1}H NMR (400 MHz, $CDCl_3$)

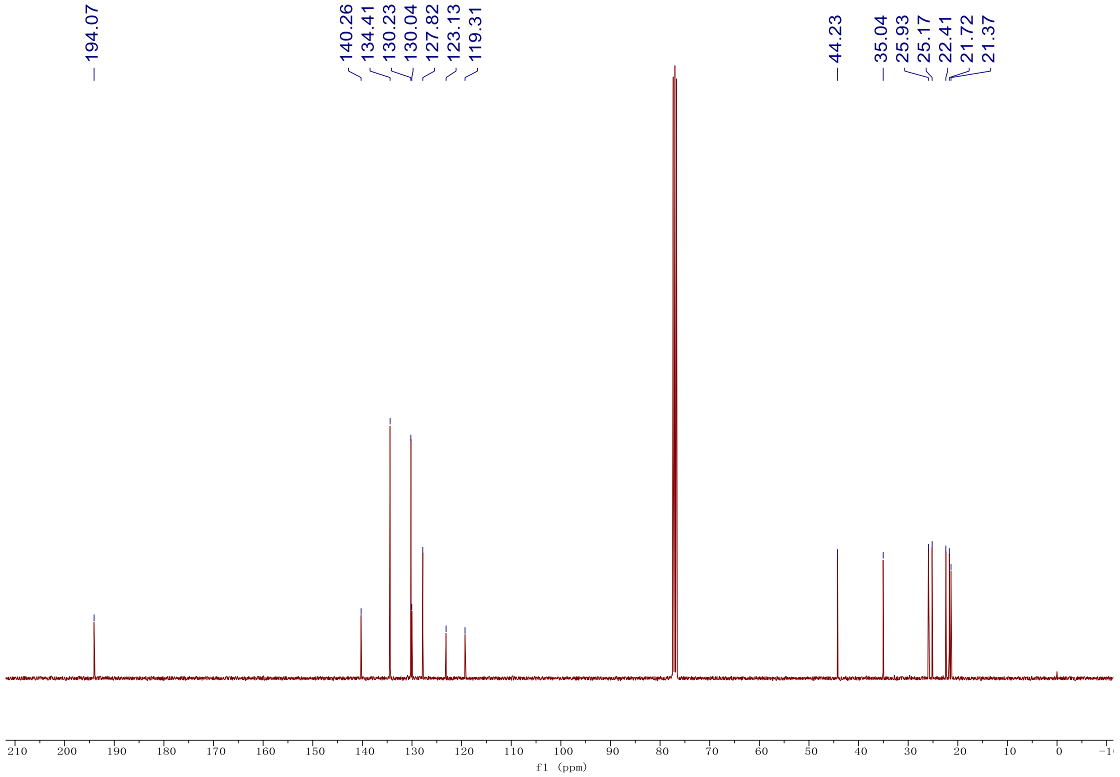

Supplementary Figure 105. ^{13}C NMR (101 MHz, $CDCl_3$)

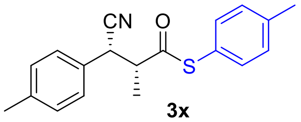

3x

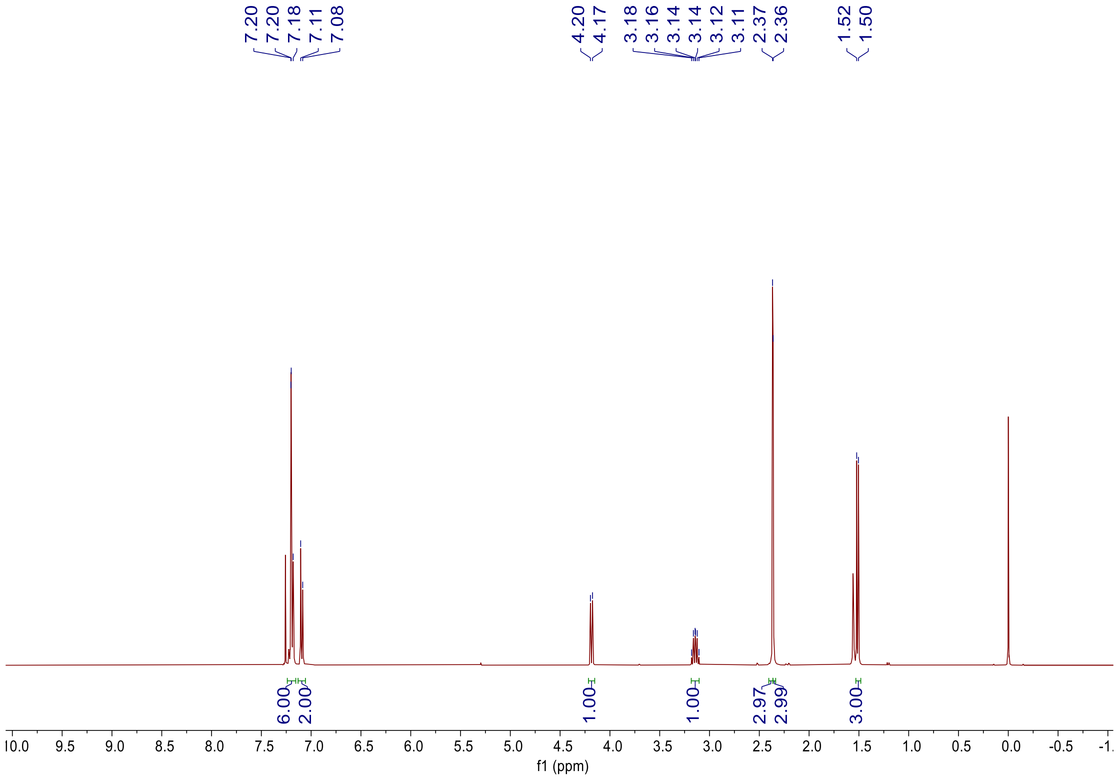

Supplementary Figure 106. ^{1}H NMR (400 MHz, $CDCl_3$)

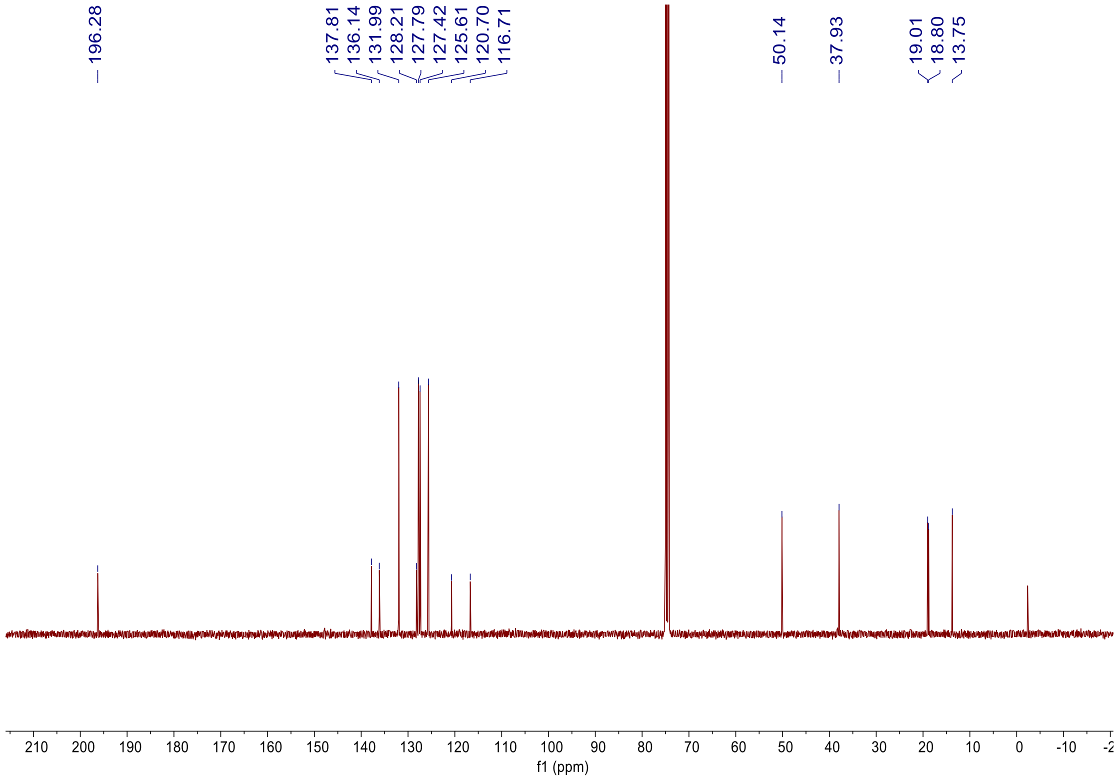

Supplementary Figure 107. ^{13}C NMR (101 MHz, $CDCl_3$)

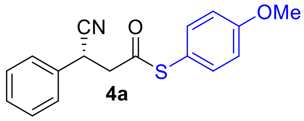

4a

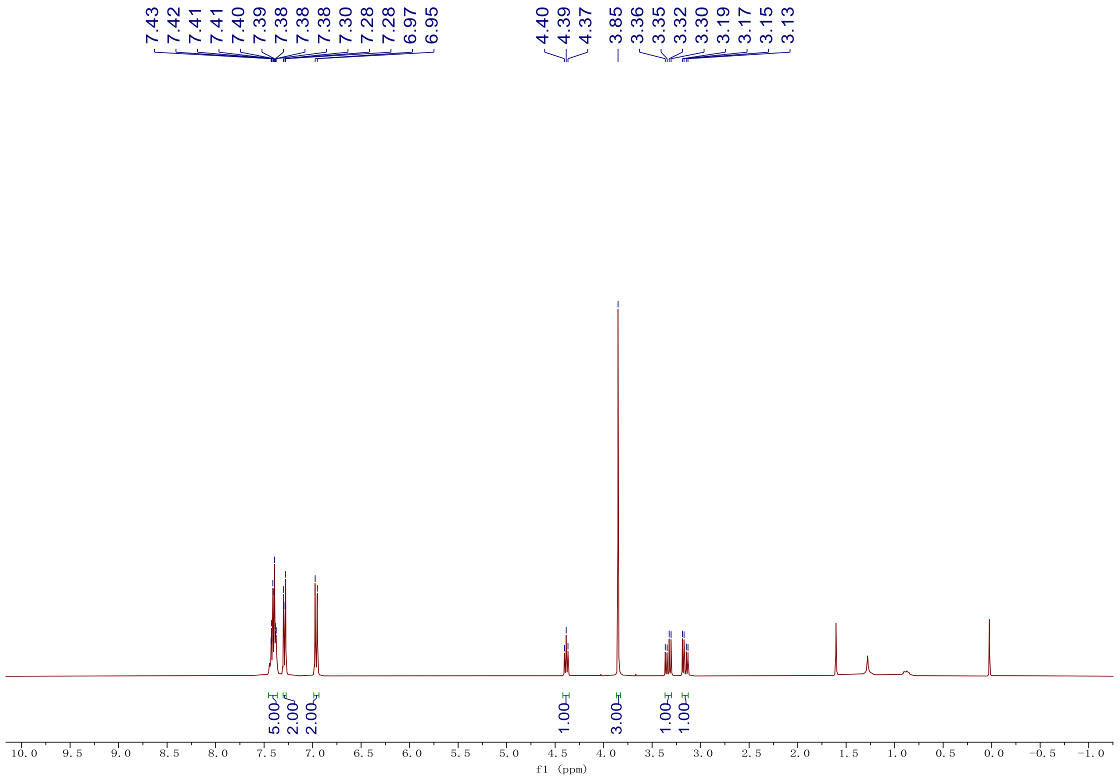

Supplementary Figure 108. ^{1}H NMR (400 MHz, CDCl$_3$)

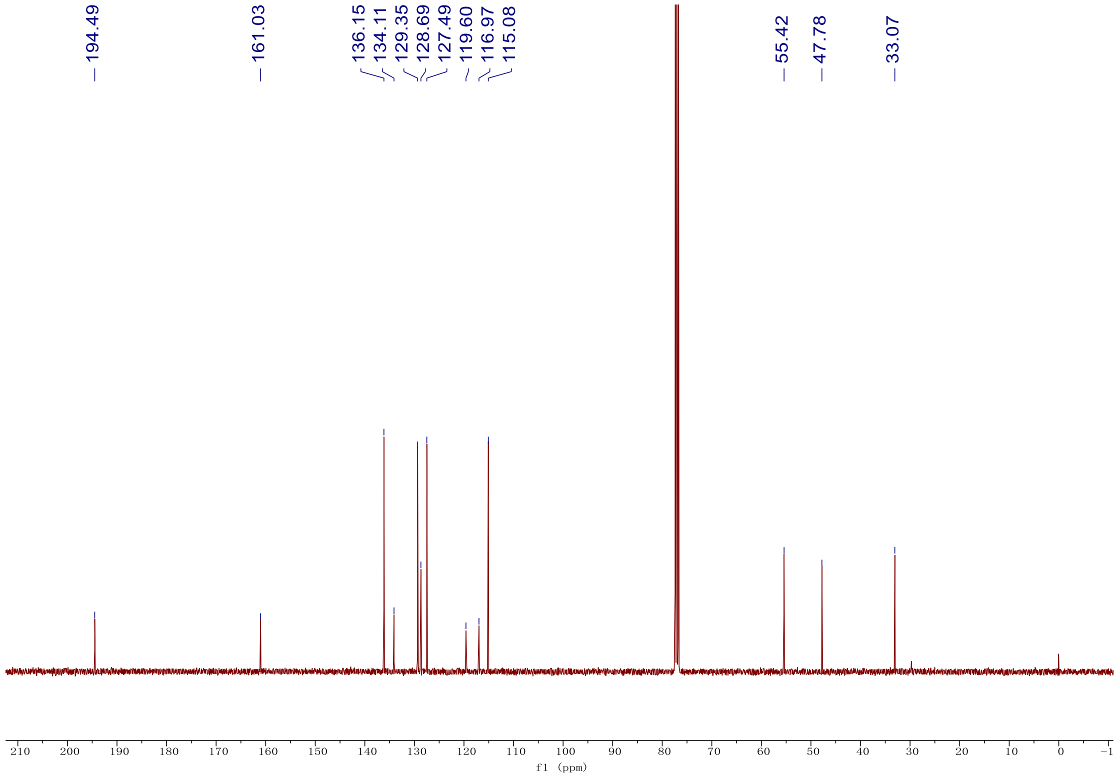

Supplementary Figure 109. ^{13}C NMR (101 MHz, CDCl$_3$)

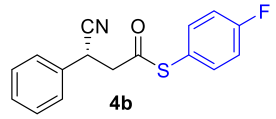

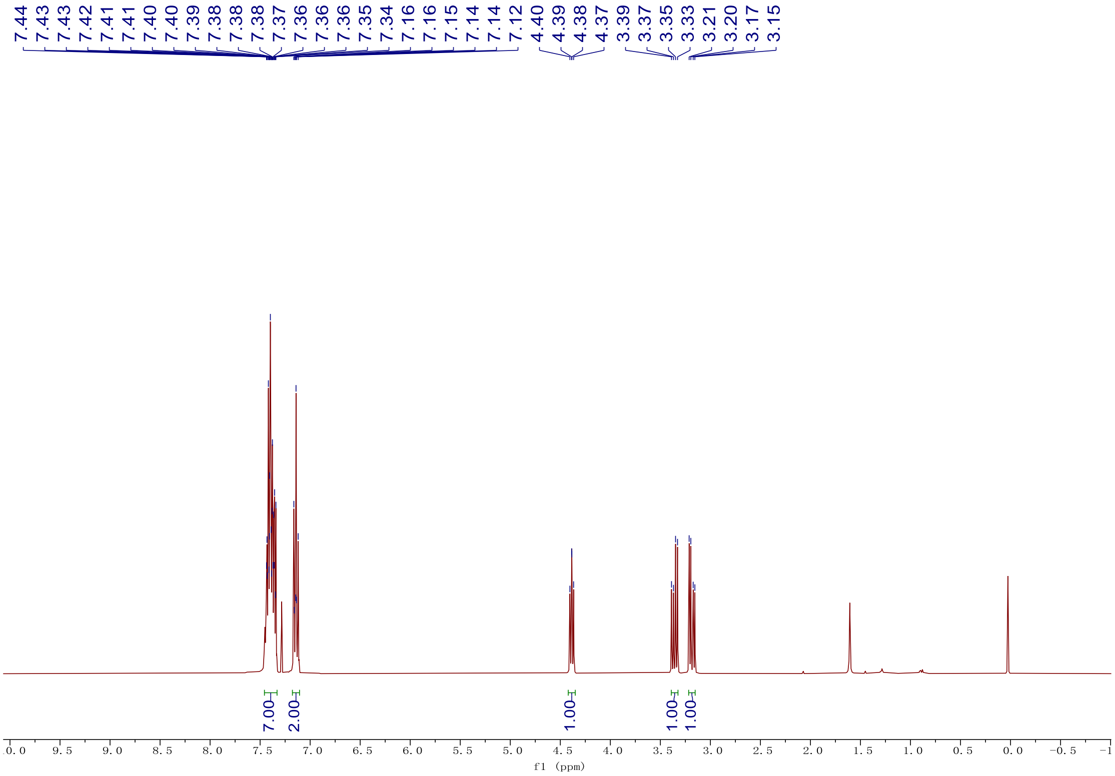

Supplementary Figure 110. ^{1}H NMR (400 MHz, $CDCl_3$)

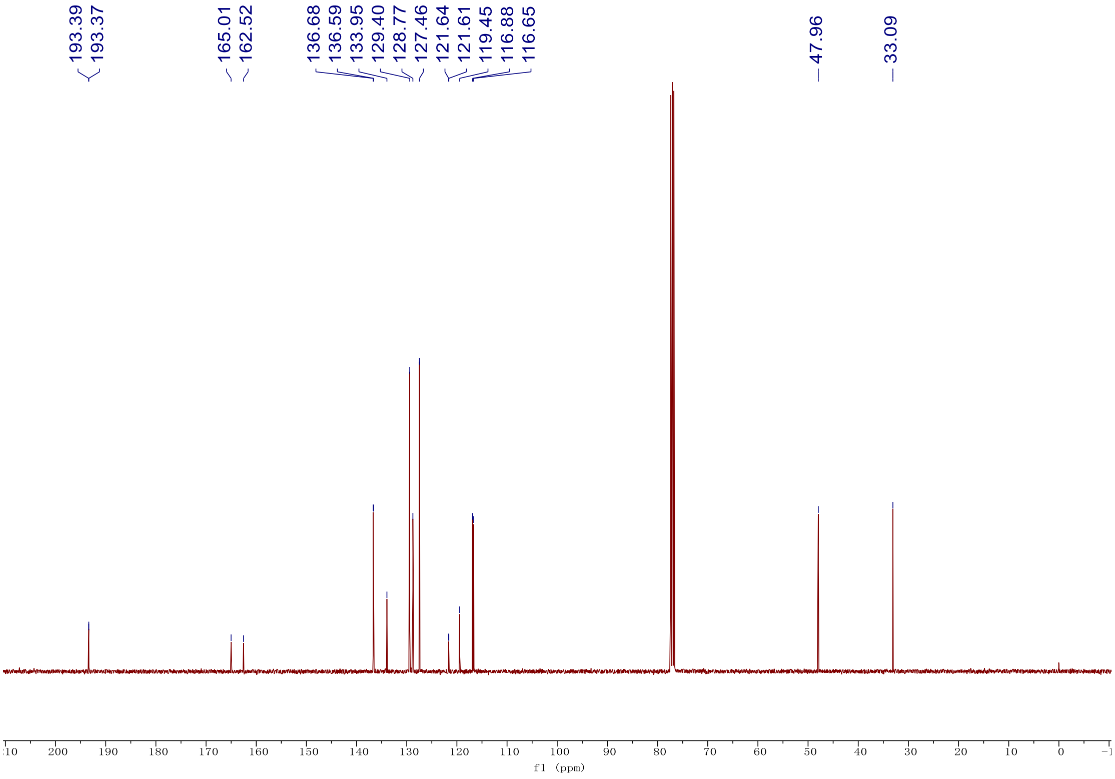

Supplementary Figure 111. ^{13}C NMR (101 MHz, $CDCl_3$)

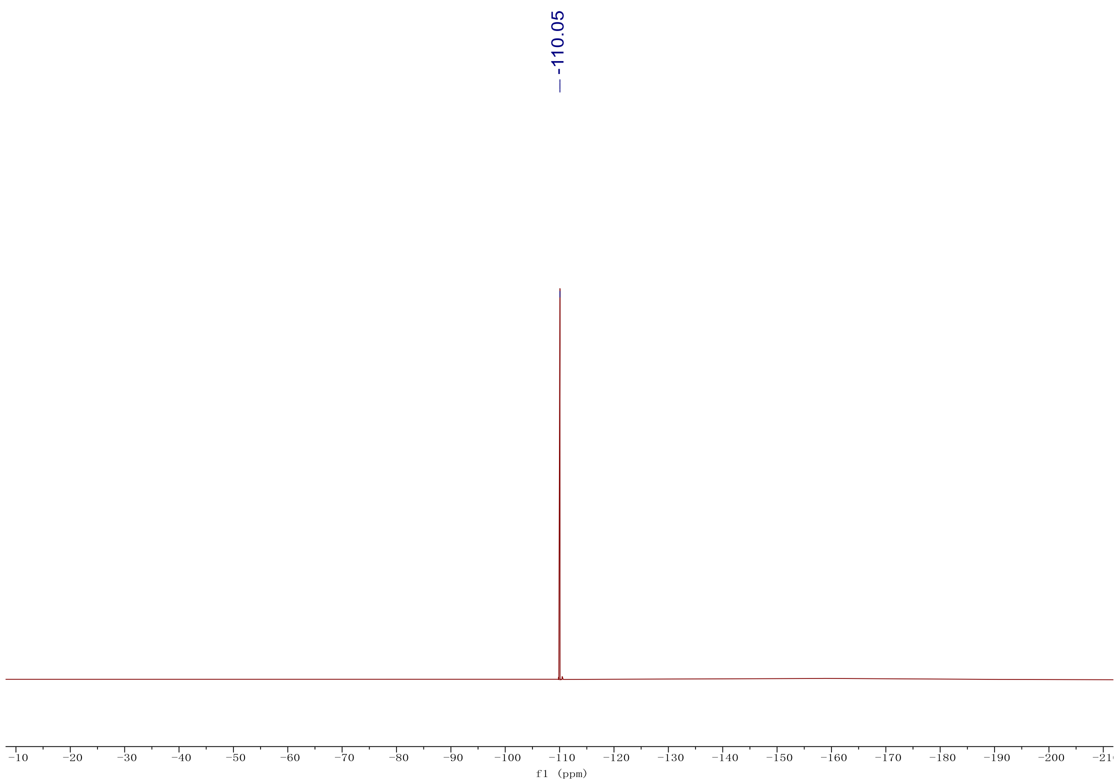

Supplementary Figure 112. ^{19}F NMR (376 MHz, $CDCl_3$)

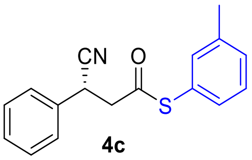

4c

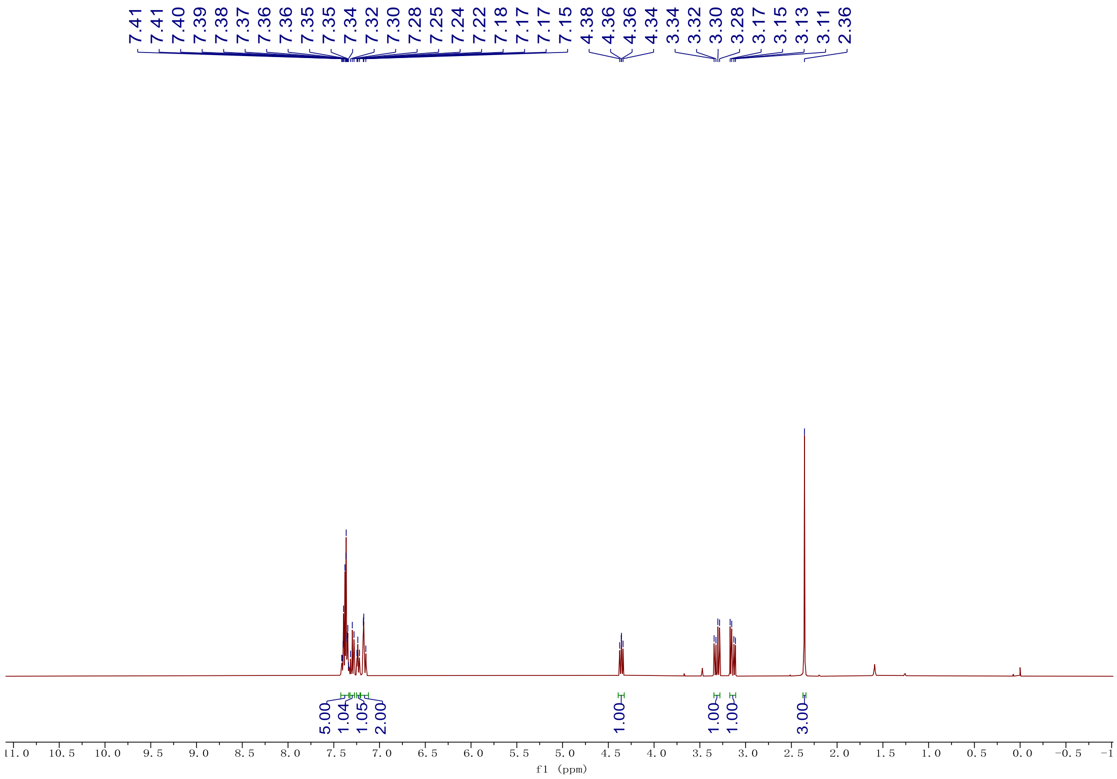

Supplementary Figure 113. ^{1}H NMR (400 MHz, $CDCl_3$)

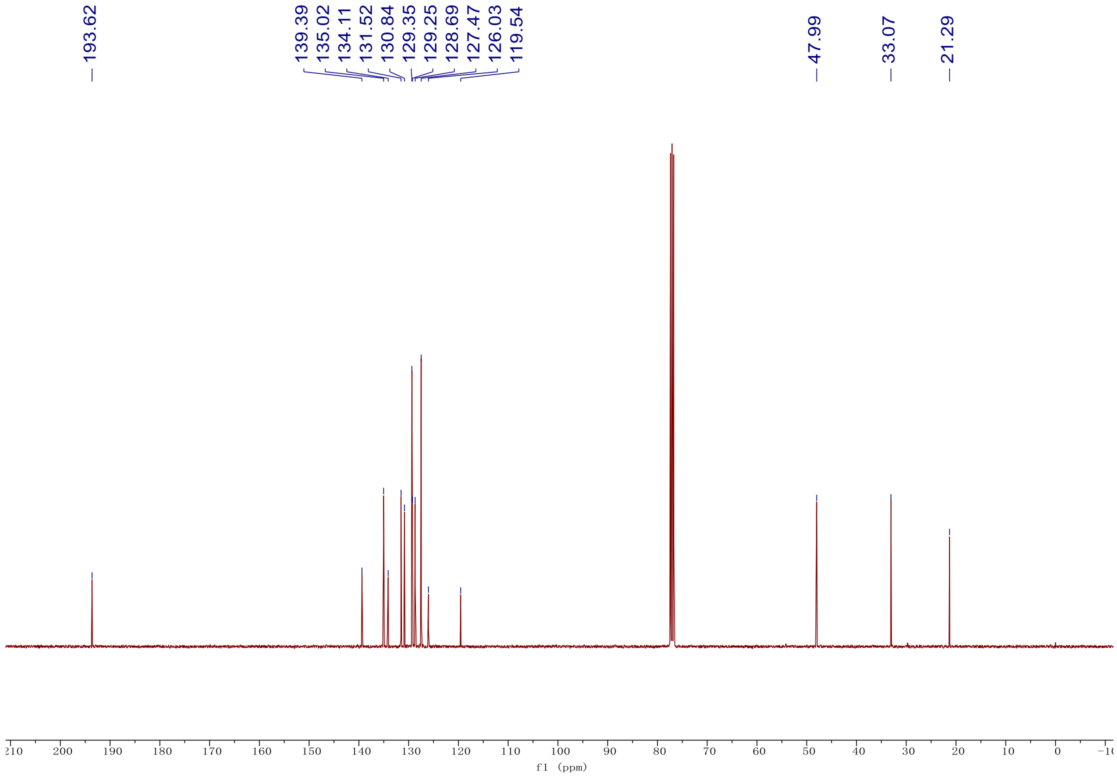

Supplementary Figure 114. ^{13}C NMR (101 MHz, $CDCl_3$)

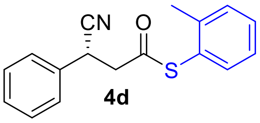

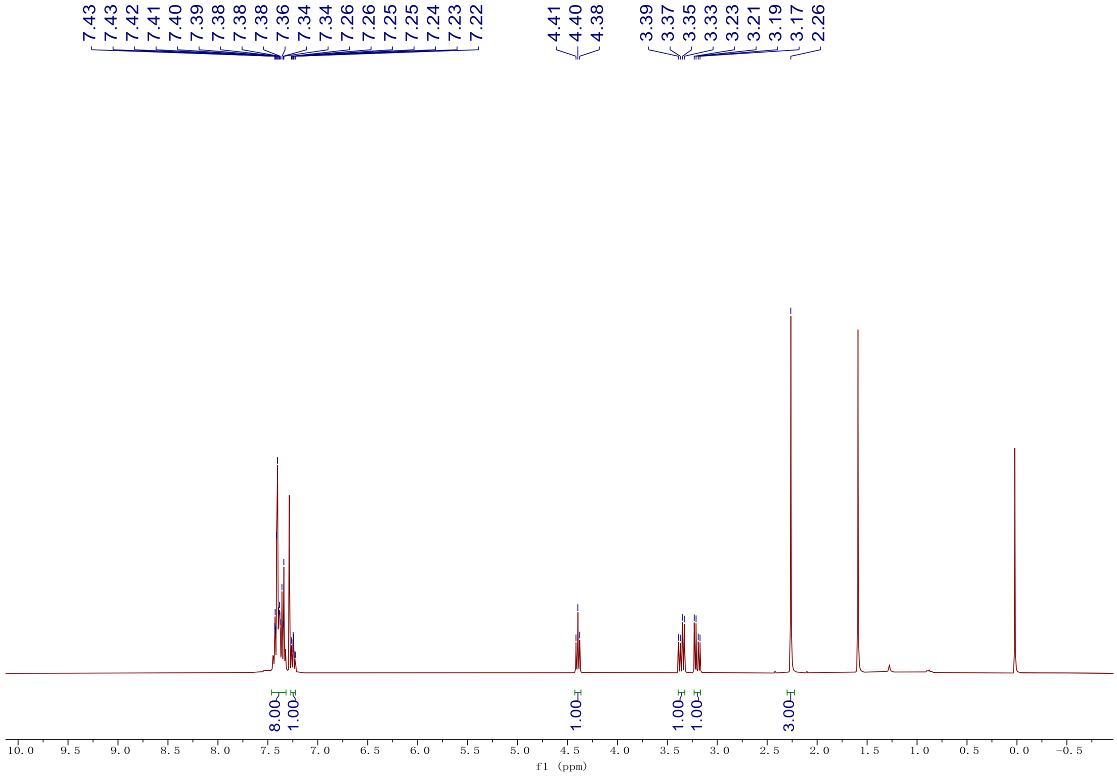

Supplementary Figure 115. ^{1}H NMR (400 MHz, $CDCl_3$)

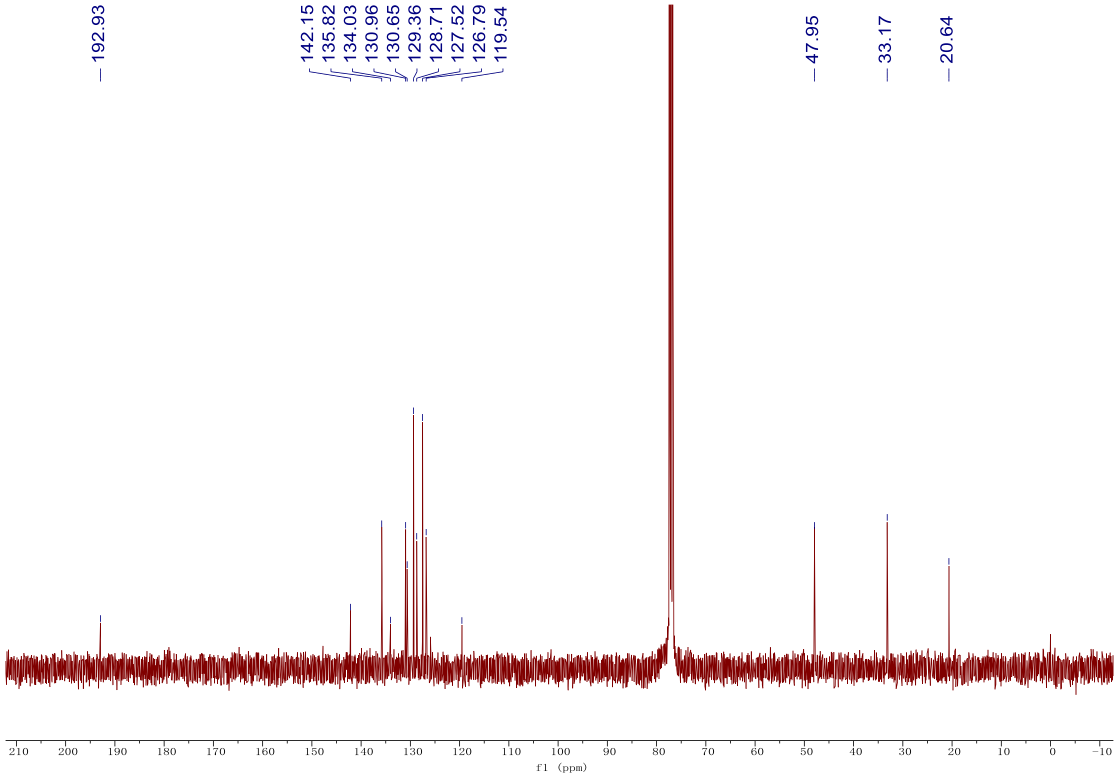

Supplementary Figure 116. ^{13}C NMR (101 MHz, $CDCl_3$)

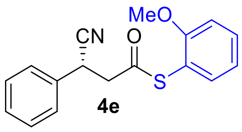

4e

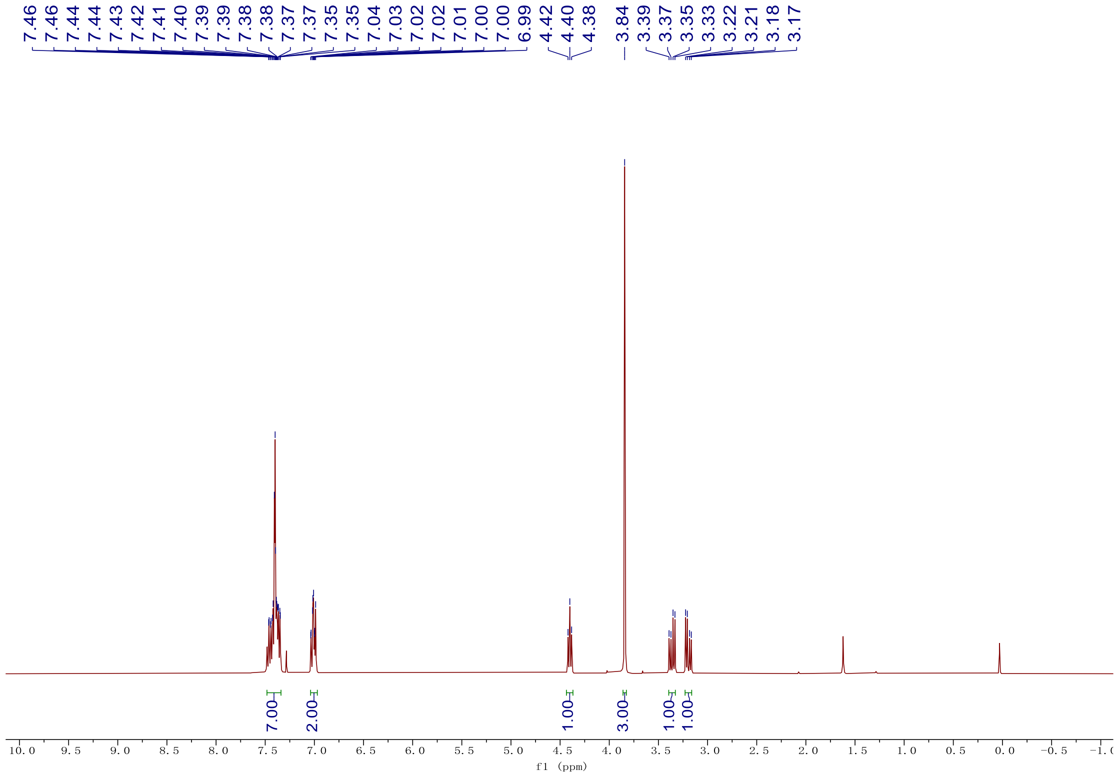

Supplementary Figure 117. ^{1}H NMR (400 MHz, $CDCl_3$)

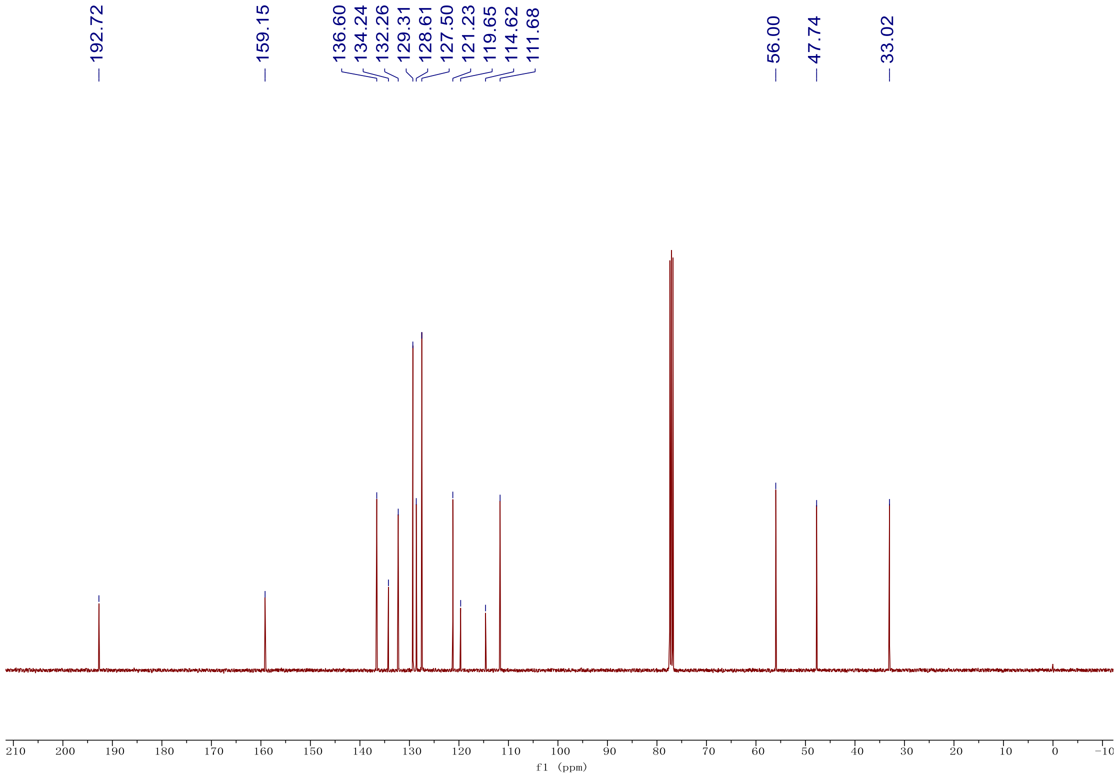

Supplementary Figure 118. ^{13}C NMR (101 MHz, $CDCl_3$)

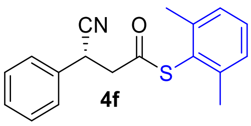

4f

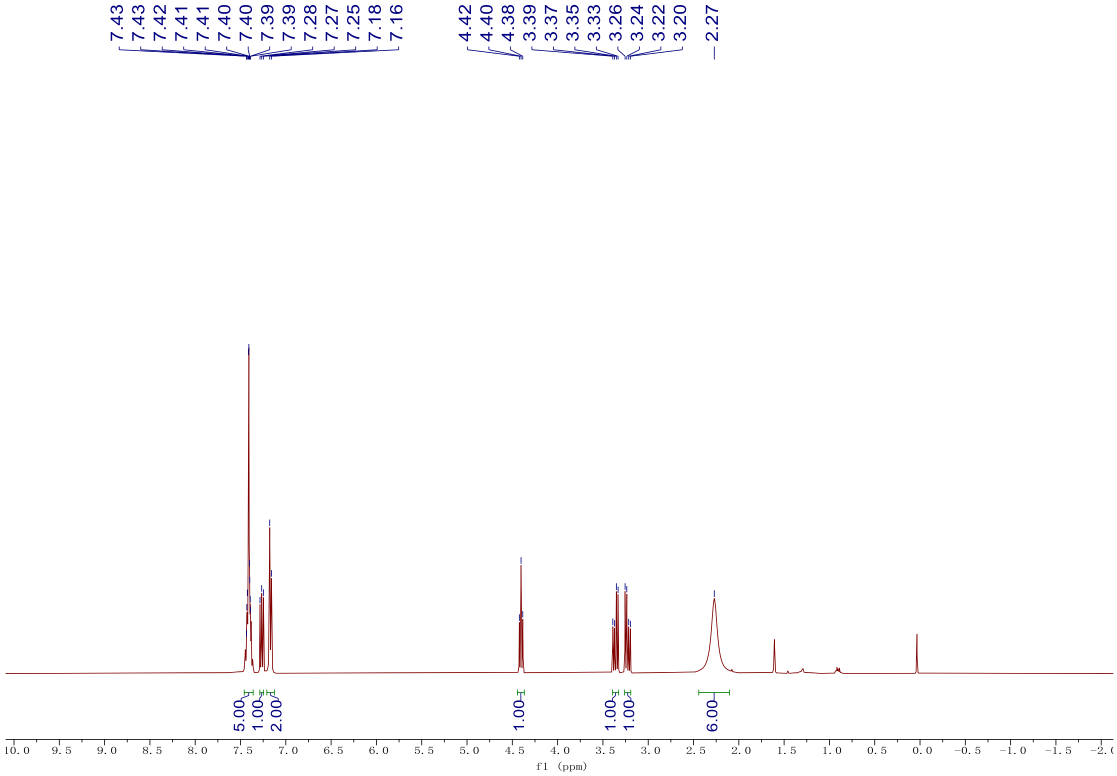

Supplementary Figure 119. ^{1}H NMR (400 MHz, $CDCl_3$)

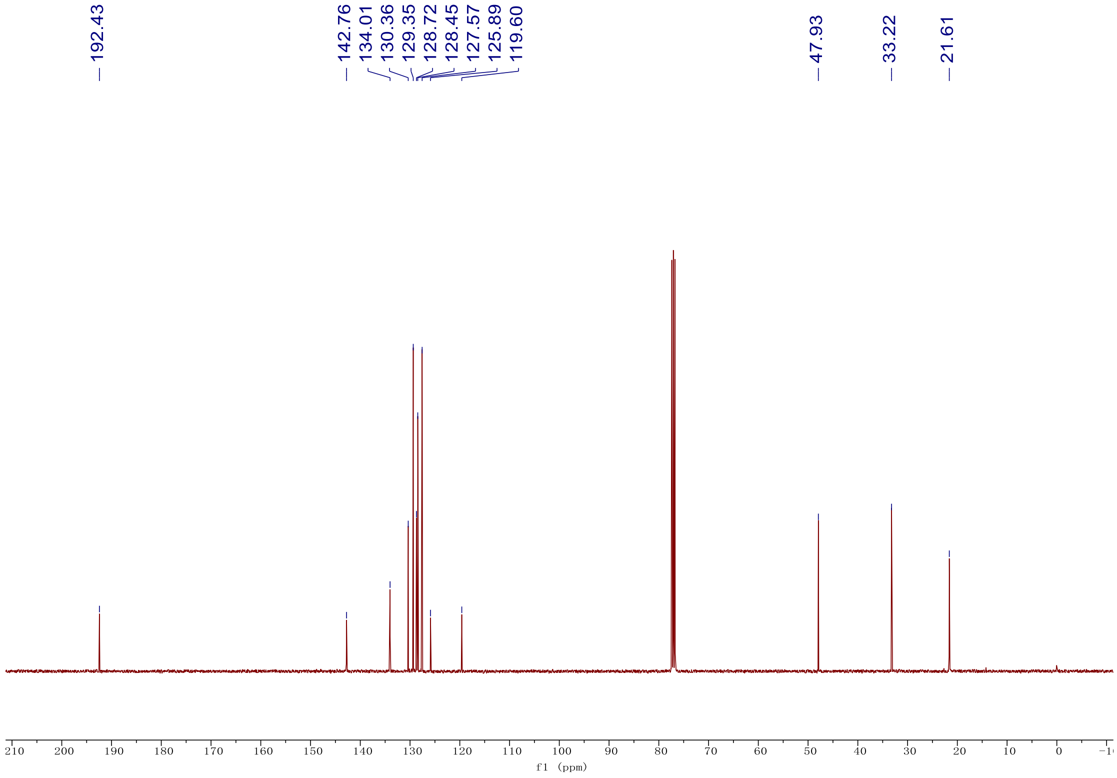

Supplementary Figure 120. ^{13}C NMR (101 MHz, $CDCl_3$)

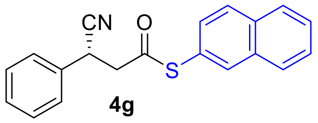

4g

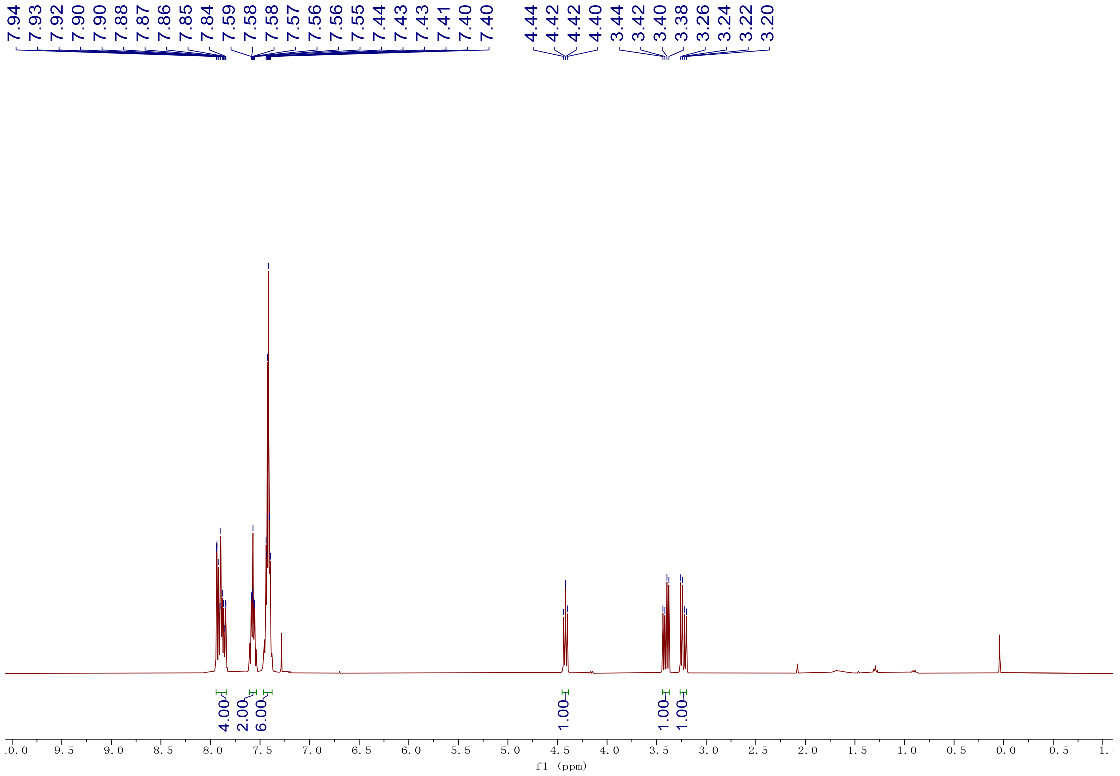

__Supplementary Figure 121. ¹H NMR (400 MHz, $CDCl_3$)__

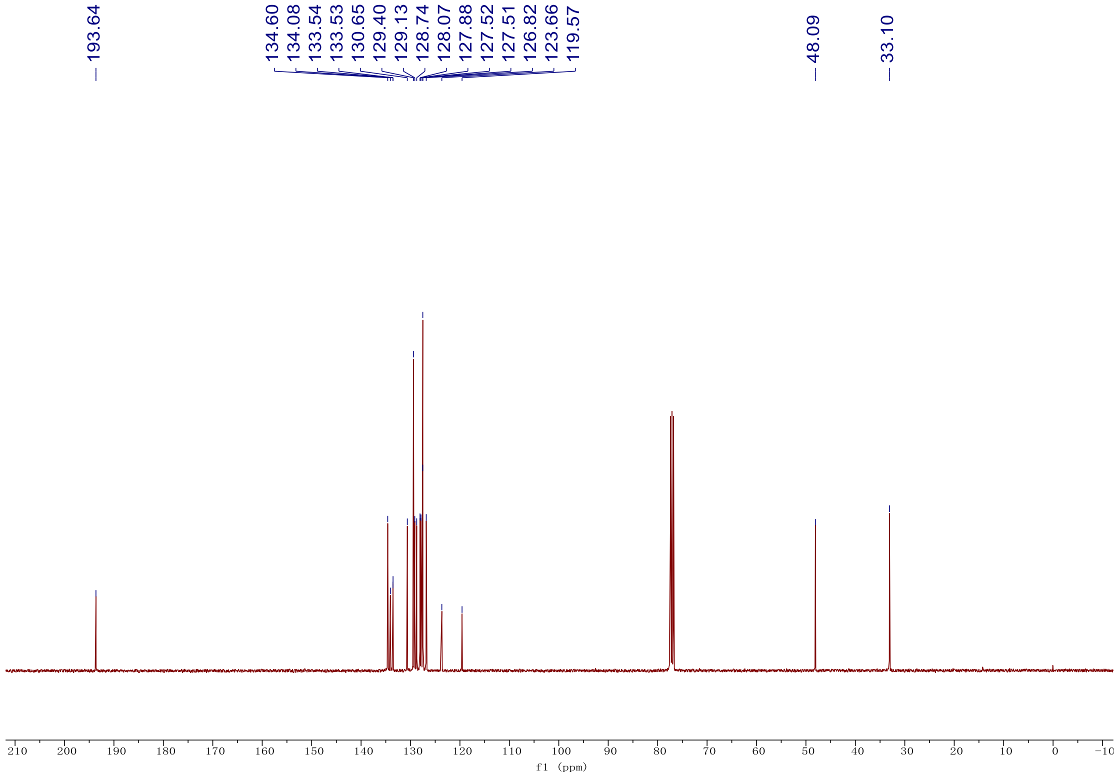

__Supplementary Figure 122. ¹³C NMR (101 MHz, $CDCl_3$)__

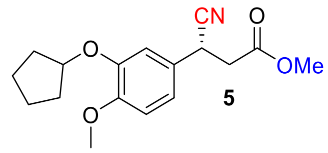

5

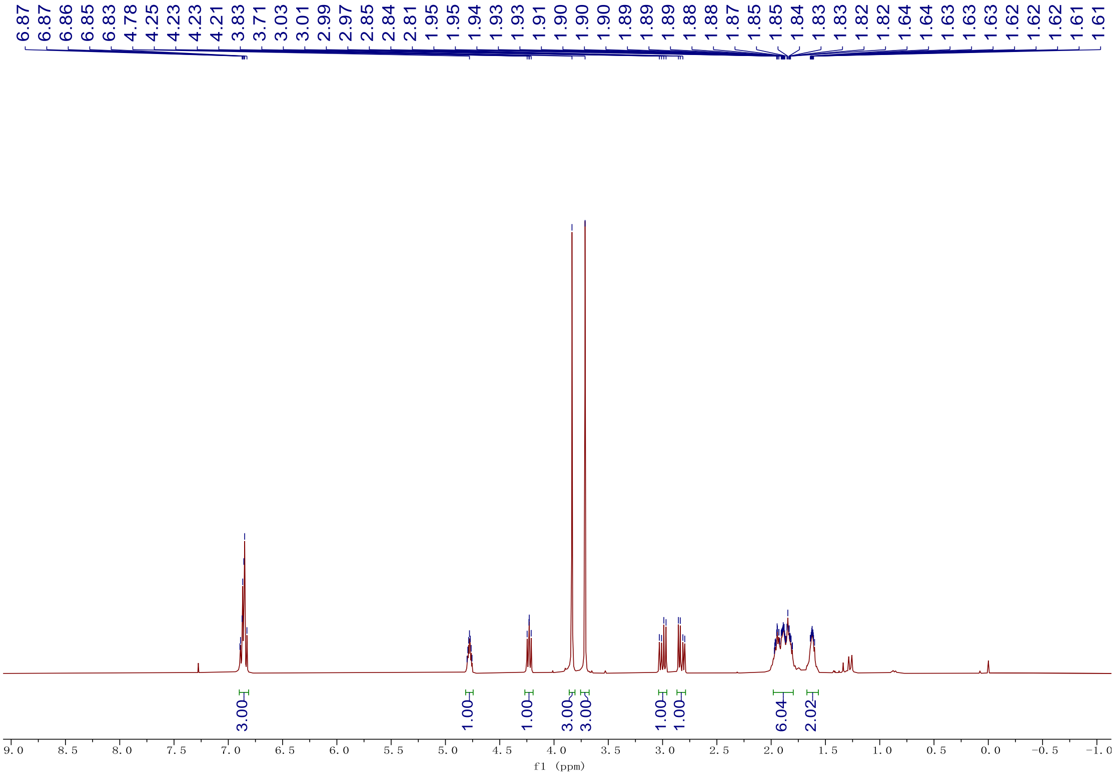

Supplementary Figure 123. ^{1}H NMR (400 MHz, $CDCl_3$)

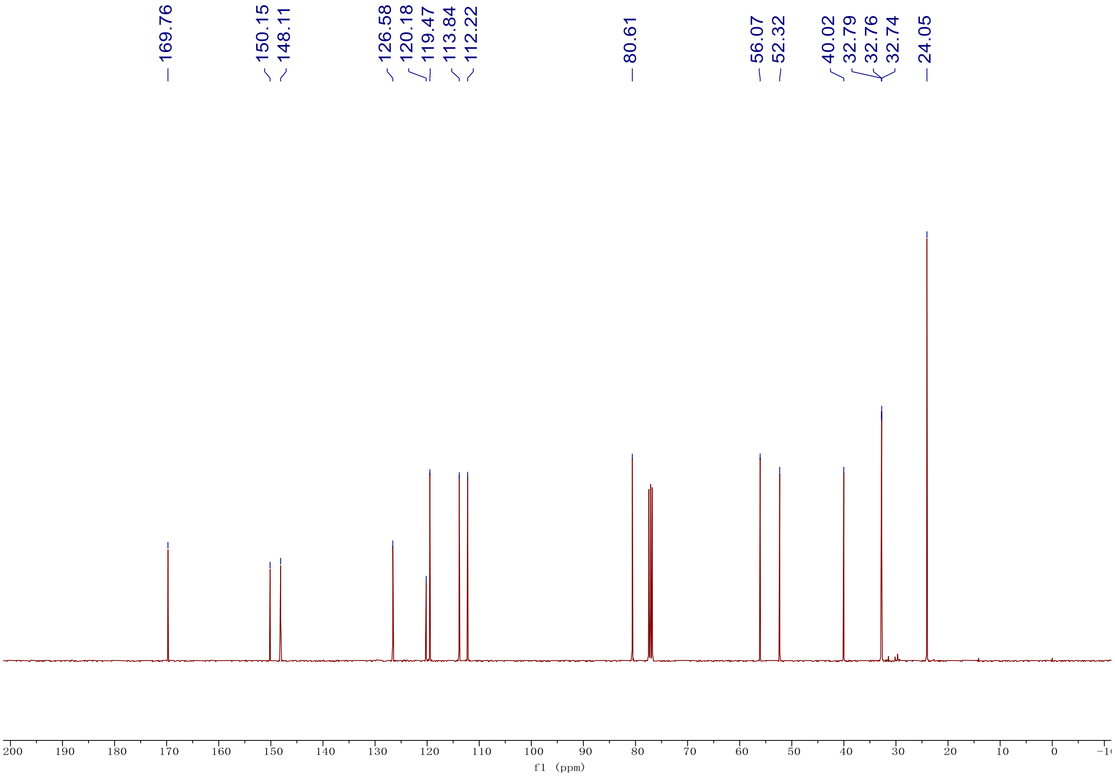

Supplementary Figure 124. ^{13}C NMR (101 MHz, $CDCl_3$)

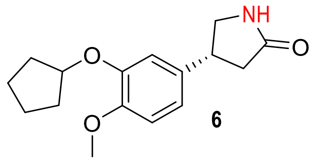

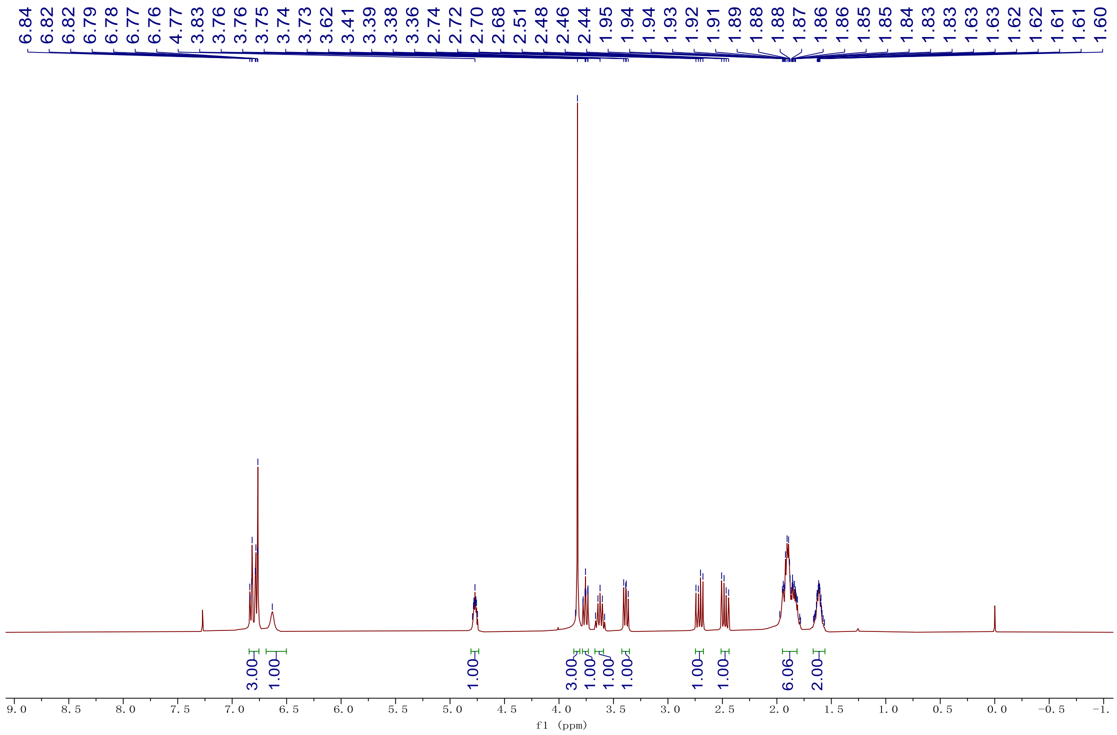

Supplementary Figure 125. ^{1}H NMR (400 MHz, $CDCl_3$)

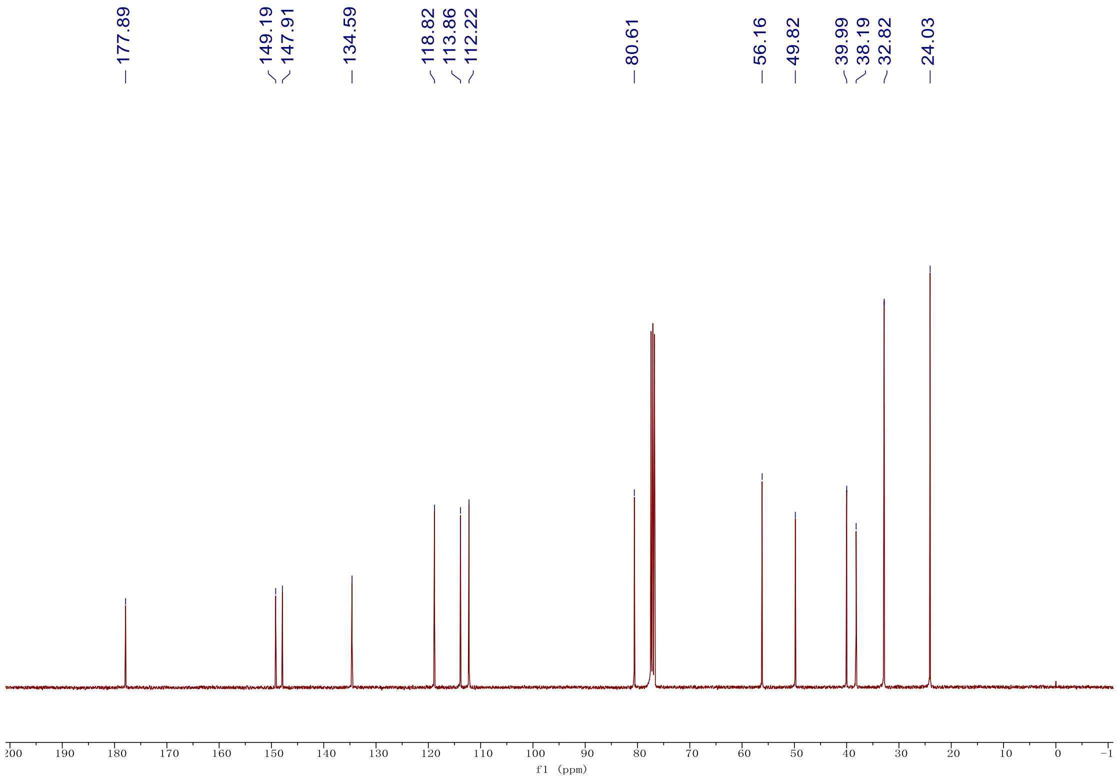

Supplementary Figure 126. ^{13}C NMR (101 MHz, $CDCl_3$)

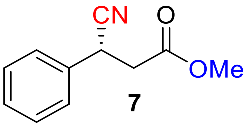

7

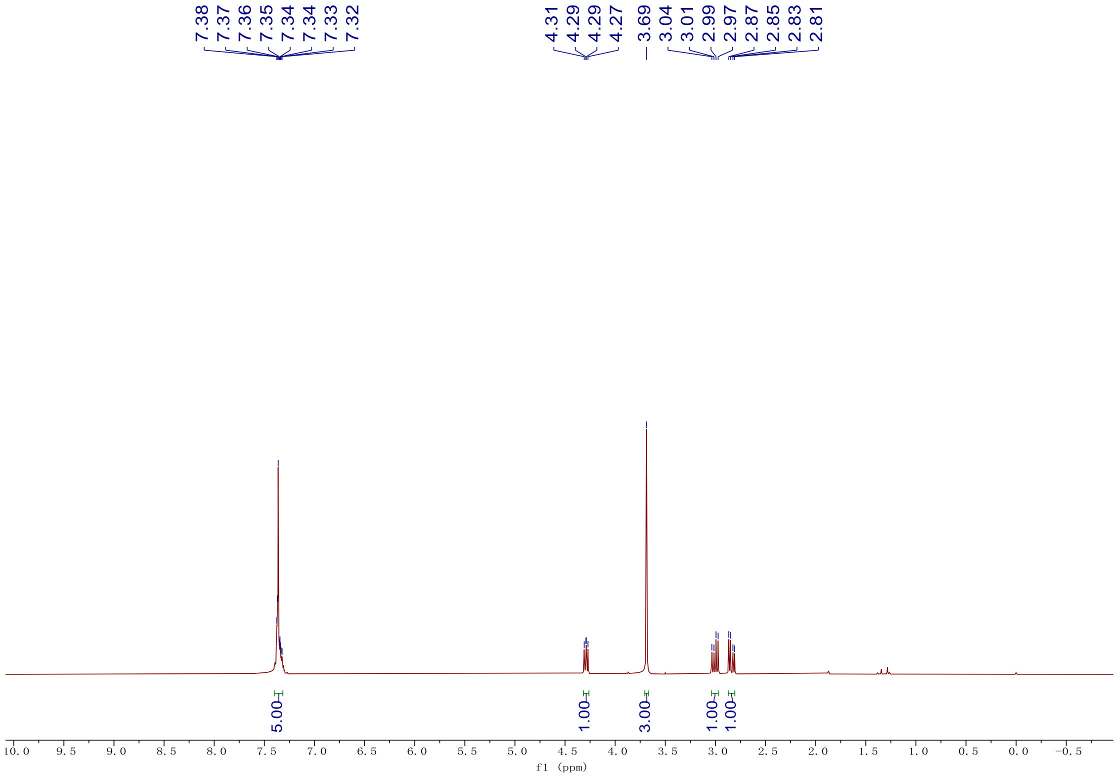

Supplementary Figure 127. ^{1}H NMR (400 MHz, $CDCl_3$)

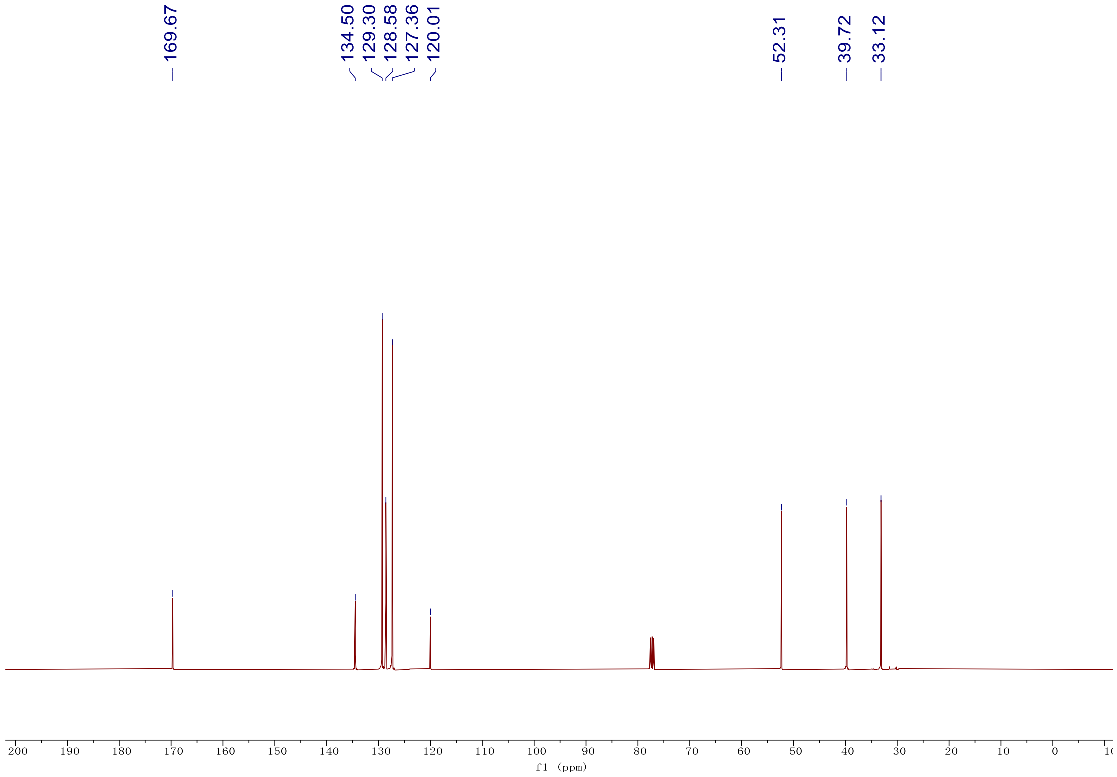

Supplementary Figure 128. ^{13}C NMR (101 MHz, $CDCl_3$)

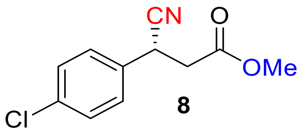

8

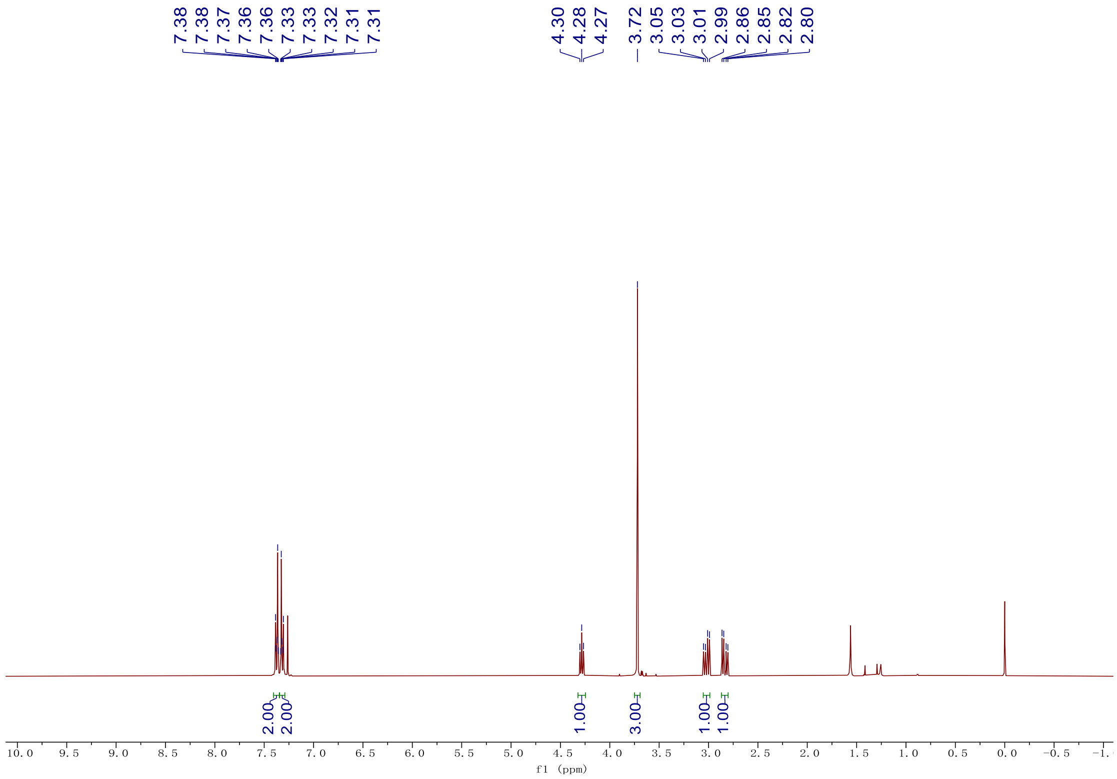

<u>Supplementary Figure 129. ^{1}H NMR (400 MHz, $CDCl_3$)</u>

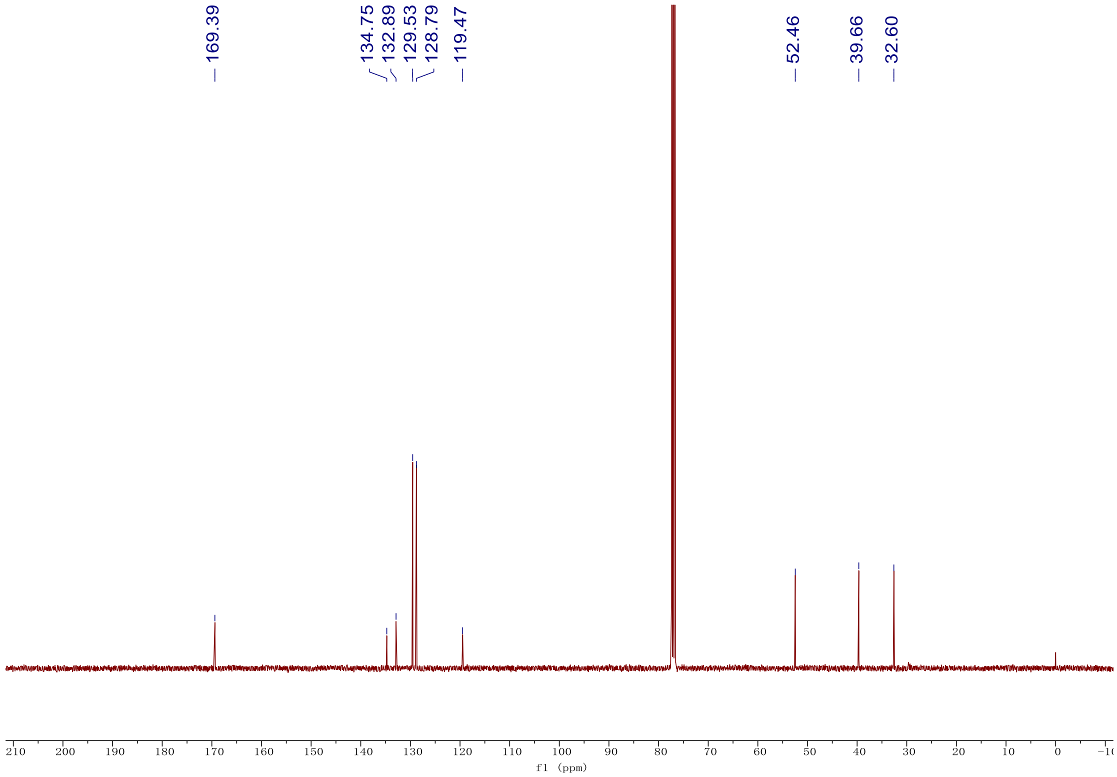

<u>Supplementary Figure 130. ^{13}C NMR (101 MHz, $CDCl_3$)</u>

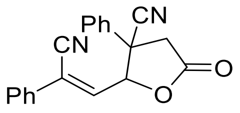

3a'

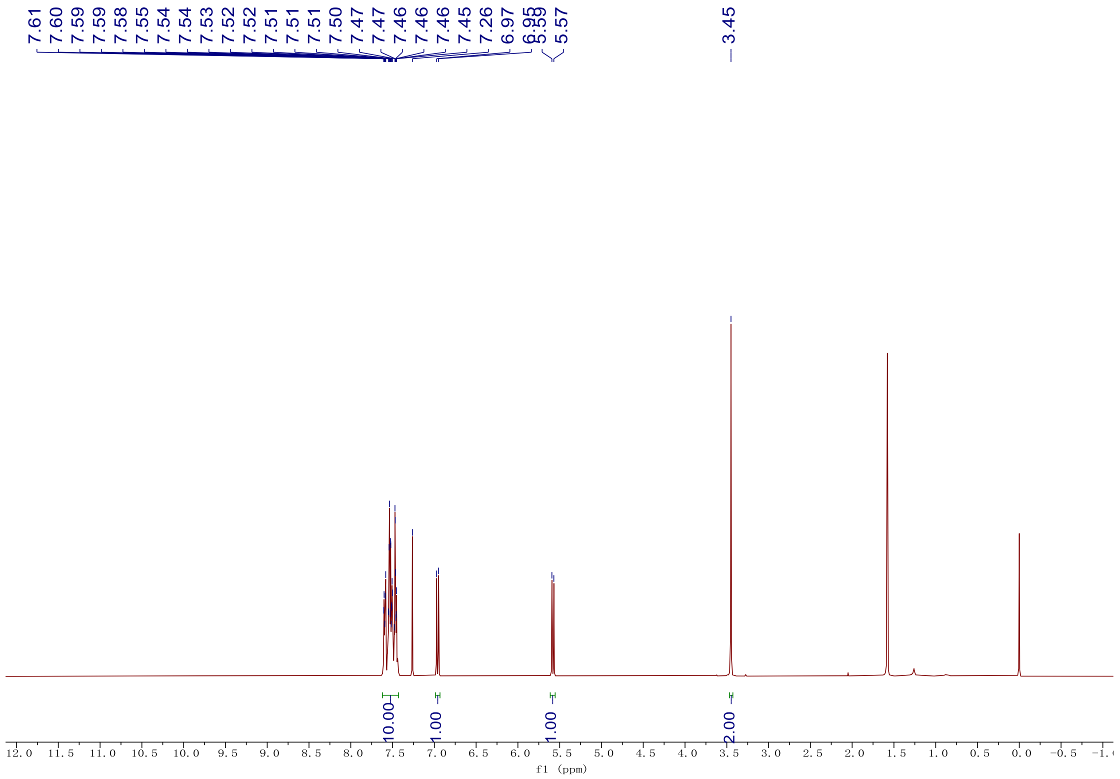

Supplementary Figure 131. ^{1}H NMR (400 MHz, $CDCl_3$)

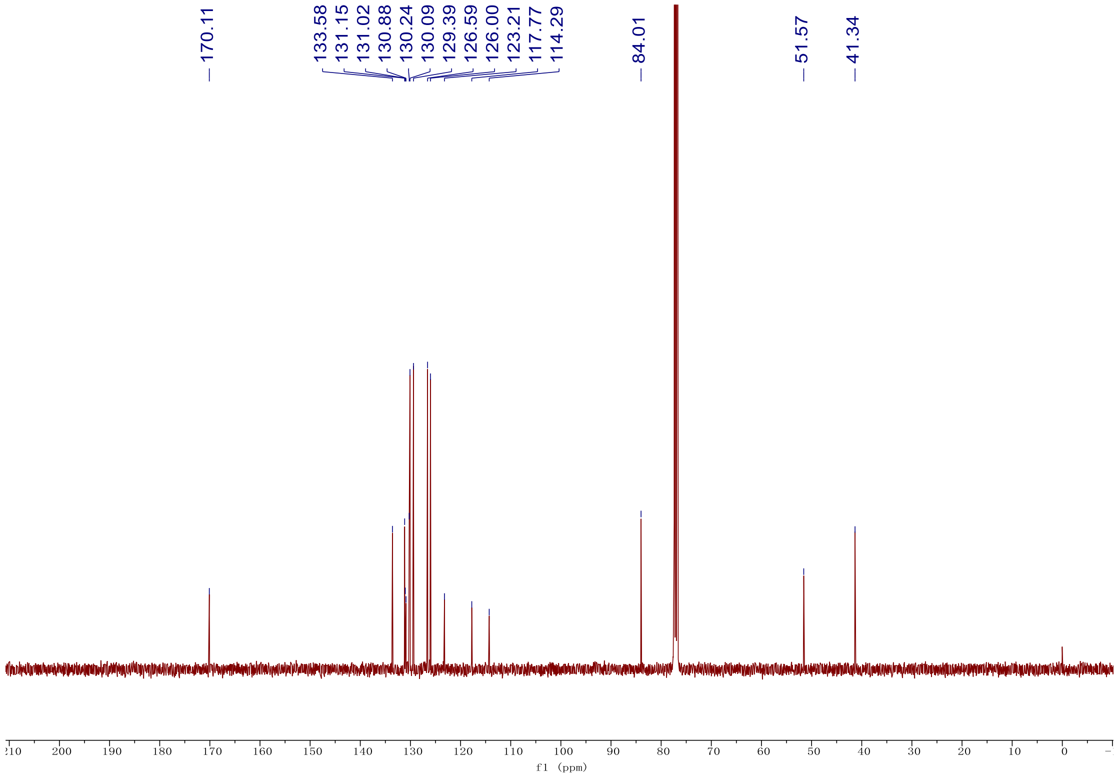

Supplementary Figure 132. ^{13}C NMR (101 MHz, $CDCl_3$)

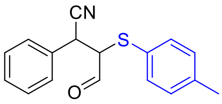

I

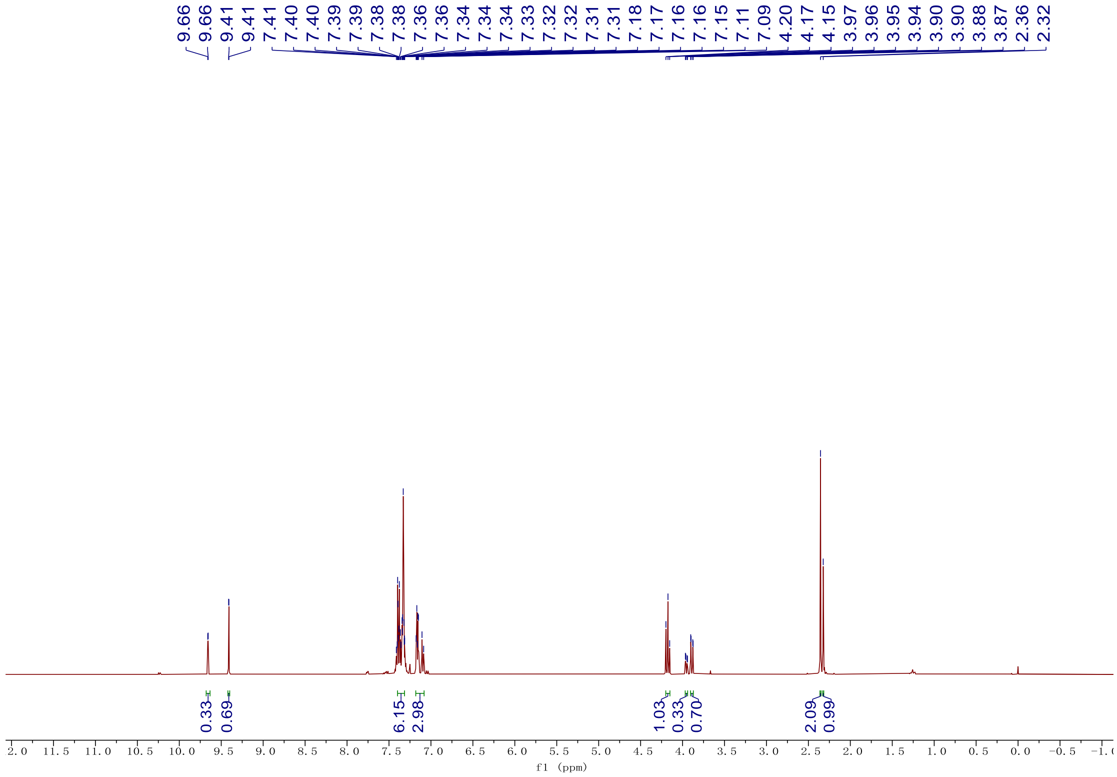

Supplementary Figure 133. ^{1}H NMR (400 MHz, $CDCl_3$)

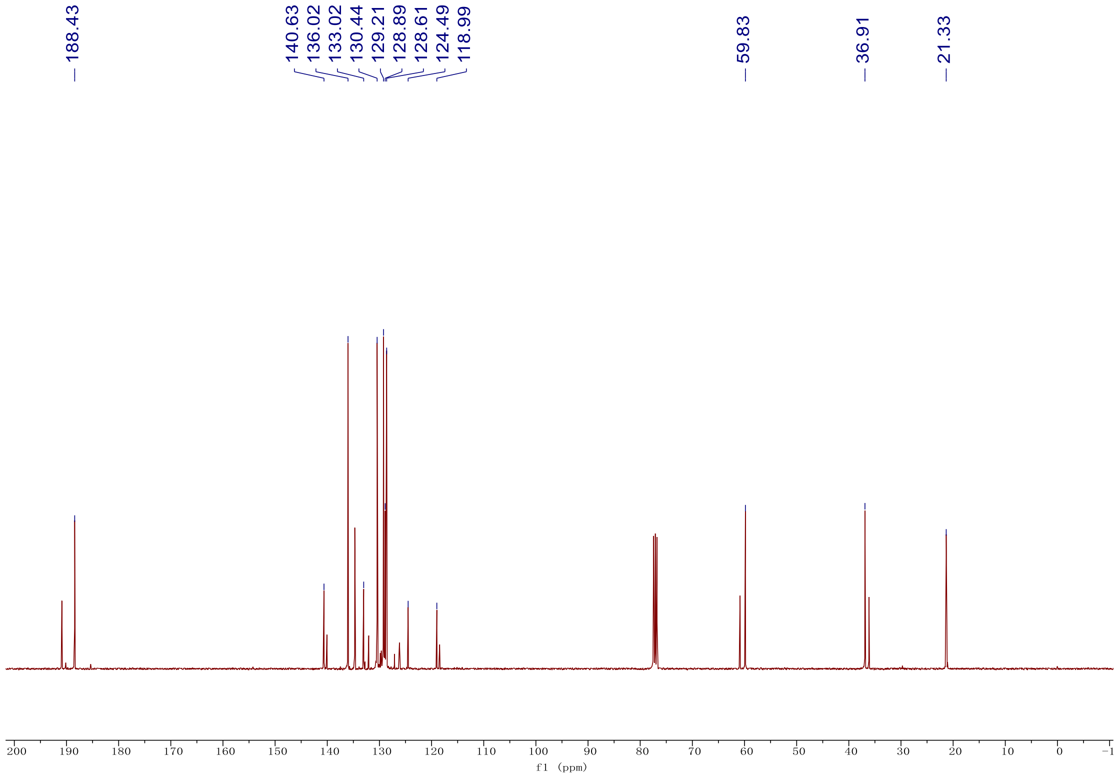

Supplementary Figure 134. ^{13}C NMR (101 MHz, $CDCl_3$)

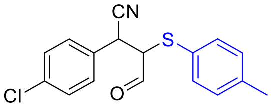

I'

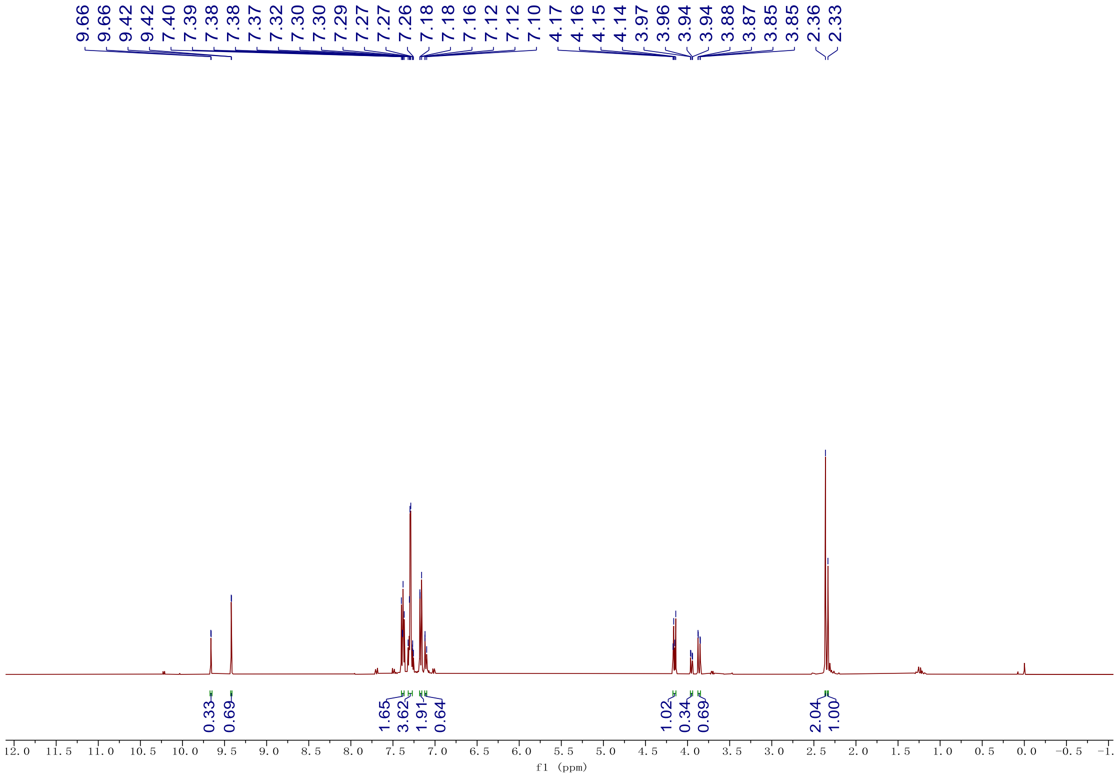

Supplementary Figure 135. ^{1}H NMR (400 MHz, $CDCl_3$)

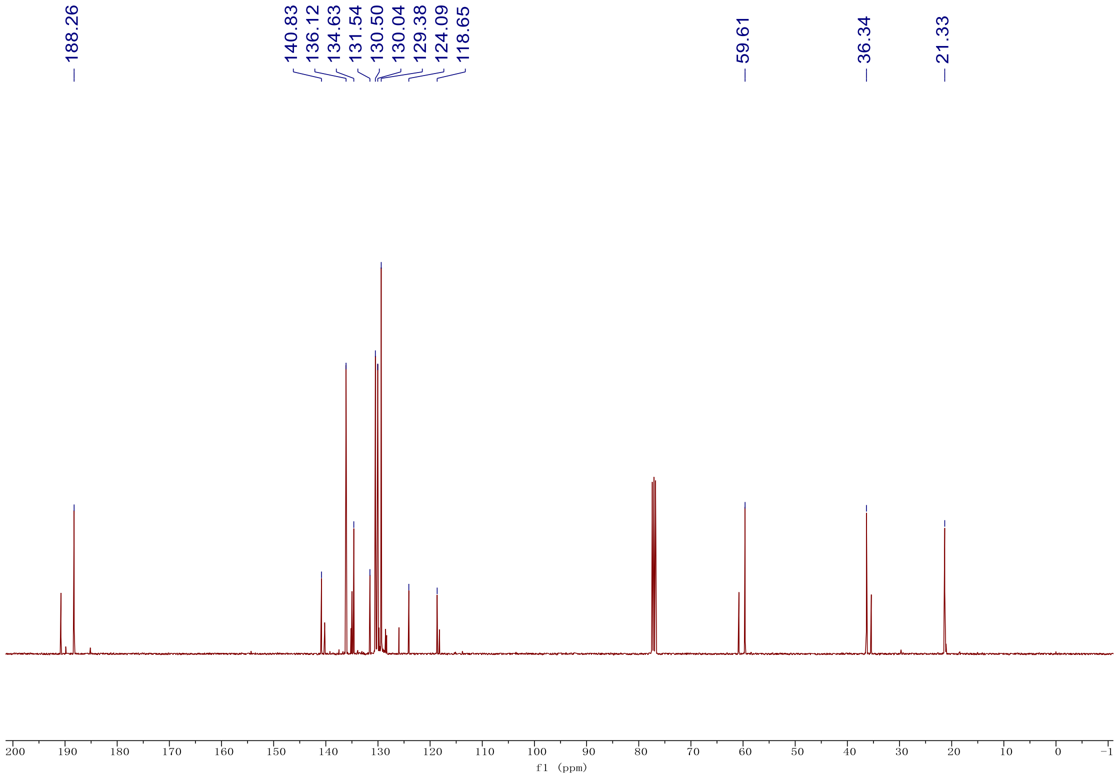

Supplementary Figure 136. ^{13}C NMR (101 MHz, $CDCl_3$)

HPLC spectra

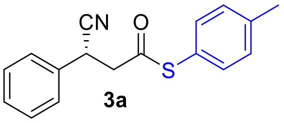

3a

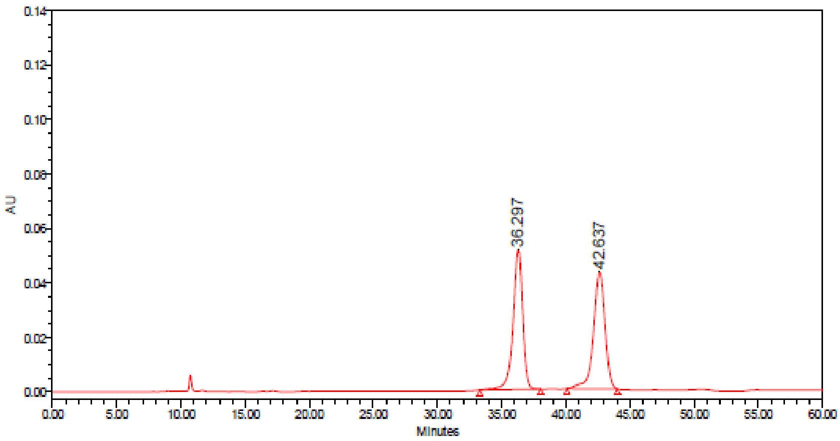

	RT	Area	% Area	Height
1	36.297	2669125	50.01	51336
2	42.637	2668192	49.99	42774

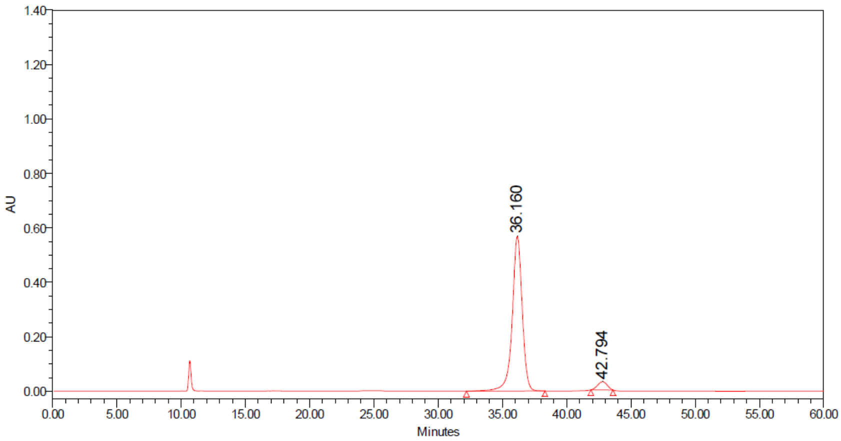

	RT	Area	% Area	Height
1	36.160	30742410	95.17	568402
2	42.794	1561412	4.83	30367

Supplementary Figure 137. HPLC spectra of 3a

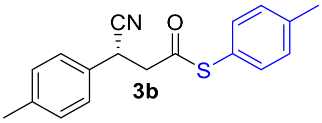

3b

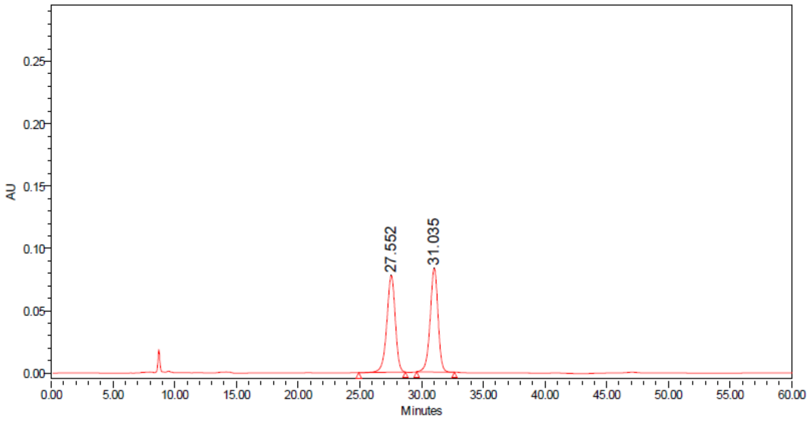

	RT	Area	% Area	Height	% Height
1	27.552	3702875	50.02	77652	48.16
2	31.035	3700399	49.98	83576	51.84

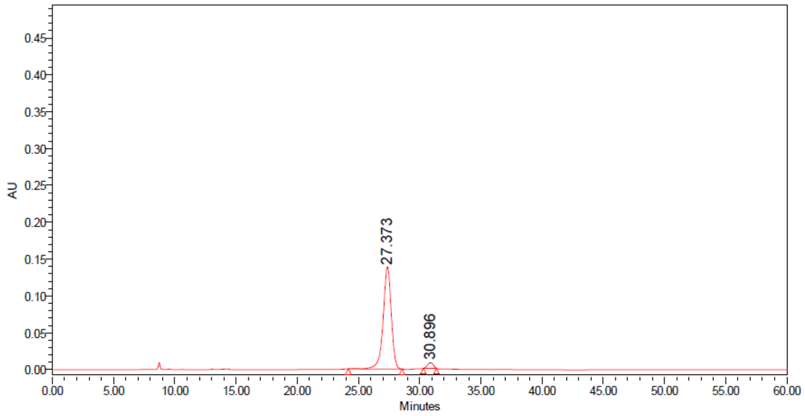

	RT	Area	% Area	Height	% Height
1	27.373	6430946	96.16	138560	94.68
2	30.896	256600	3.84	7778	5.32

Supplementary Figure 138. HPLC spectra of 3b

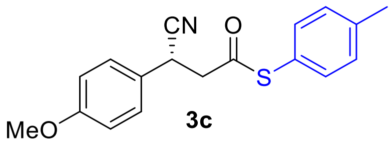

3c

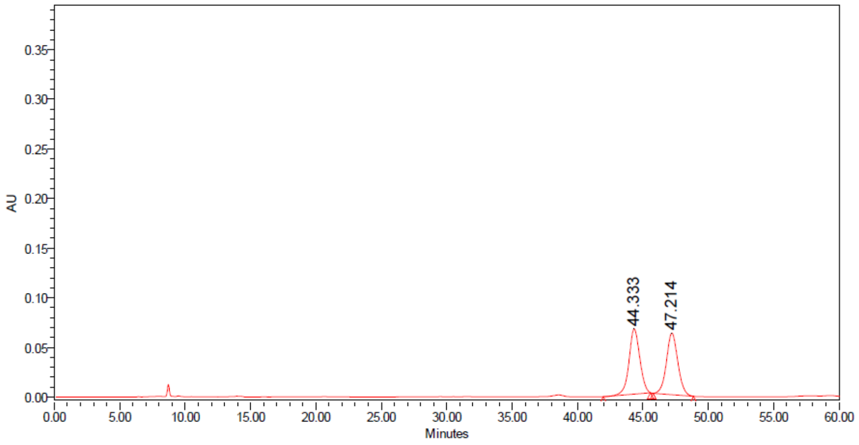

	RT	Area	% Area	Height	% Height
1	44.333	3777699	50.06	65681	51.44
2	47.214	3769168	49.94	62015	48.56

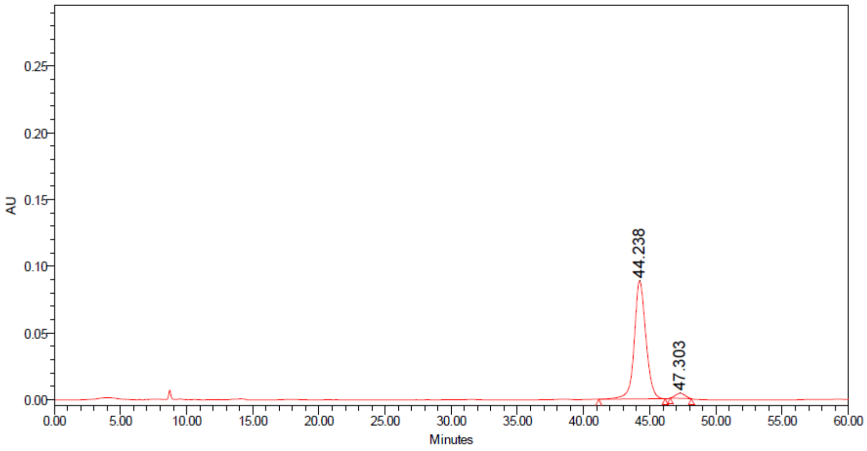

	RT	Area	% Area	Height	% Height
1	44.238	5366193	96.54	88435	95.80
2	47.303	192584	3.46	3875	4.20

Supplementary Figure 139. HPLC spectra of 3c

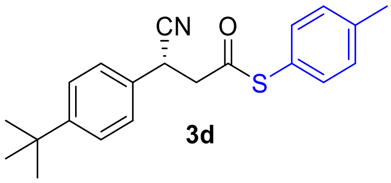

3d

<Chromatogram>

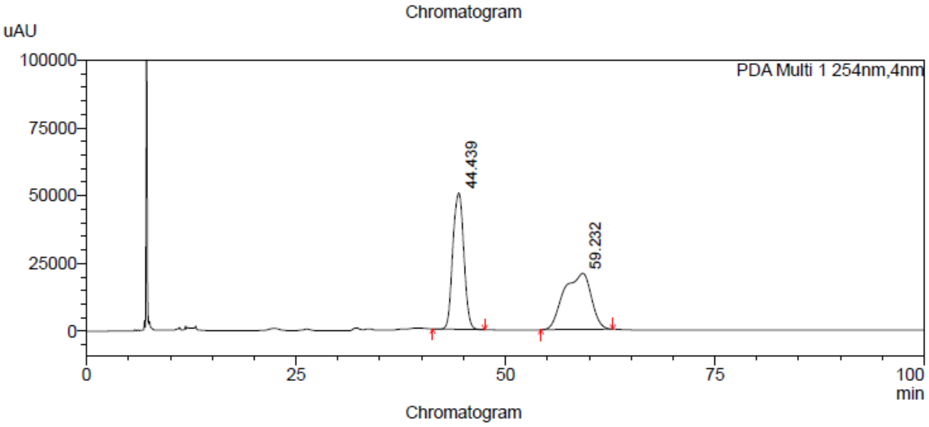

<Peak Table>

PDA Ch1 254nm

Peak#	Ret. Time	Area	Height	Area%	Height%
1	44.439	4605010	50227	49.576	70.804
2	59.232	4683864	20711	50.424	29.196
Total		9288874	70939	100.000	100.000

<Chromatogram>

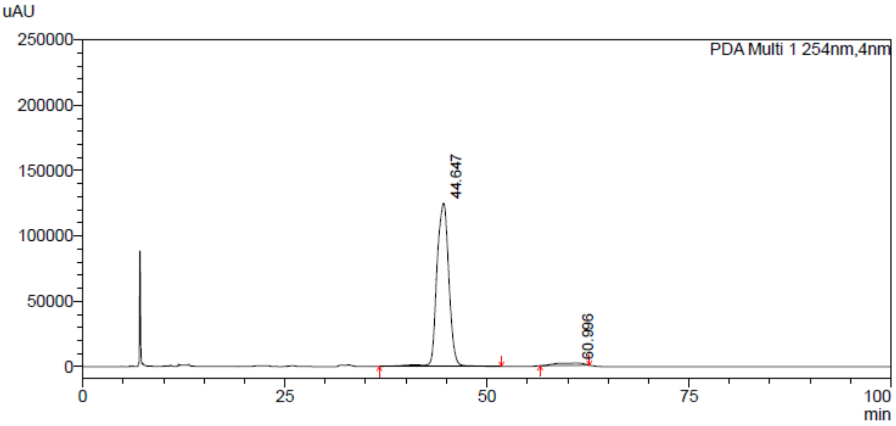

<Peak Table>

PDA Ch1 254nm

Peak#	Ret. Time	Area	Height	Area%	Height%
1	44.647	12861697	124962	96.740	98.614
2	60.996	433381	1756	3.260	1.386
Total		13295078	126717	100.000	100.000

Supplementary Figure 140. HPLC spectra of 3d

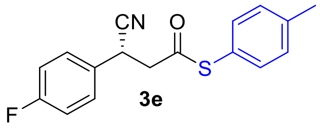

3e

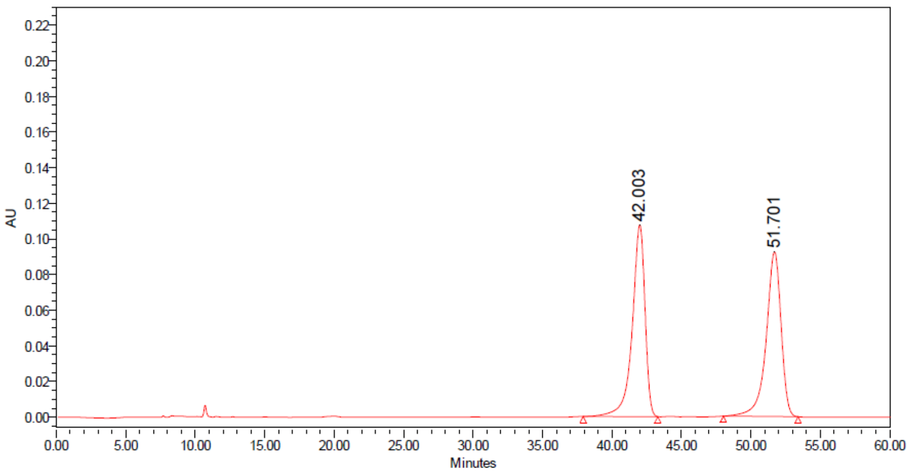

	RT	Area	% Area	Height	% Height
1	42.003	6896078	50.07	107588	53.78
2	51.701	6877237	49.93	92454	46.22

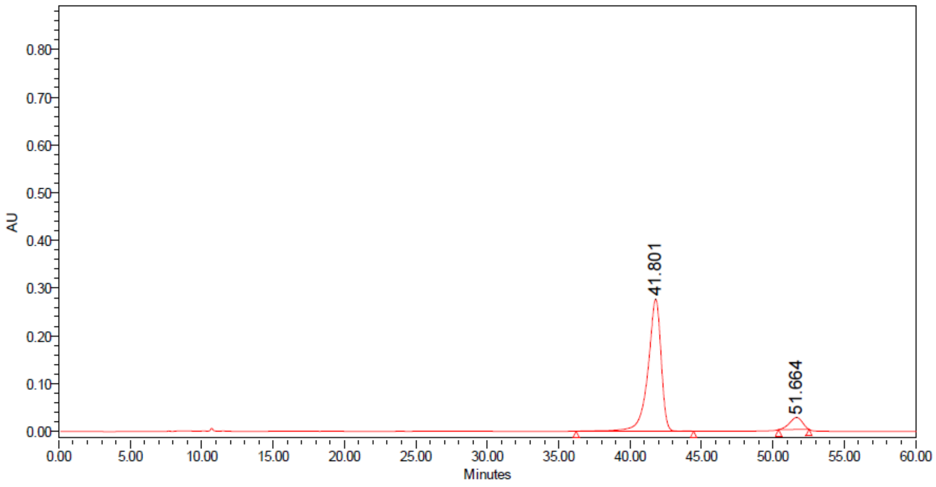

	RT	Area	% Area	Height	% Height
1	41.801	17640116	91.88	276886	91.65
2	51.664	1558281	8.12	25237	8.35

Supplementary Figure 141. HPLC spectra of 3e

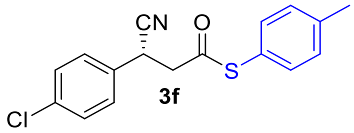

3f

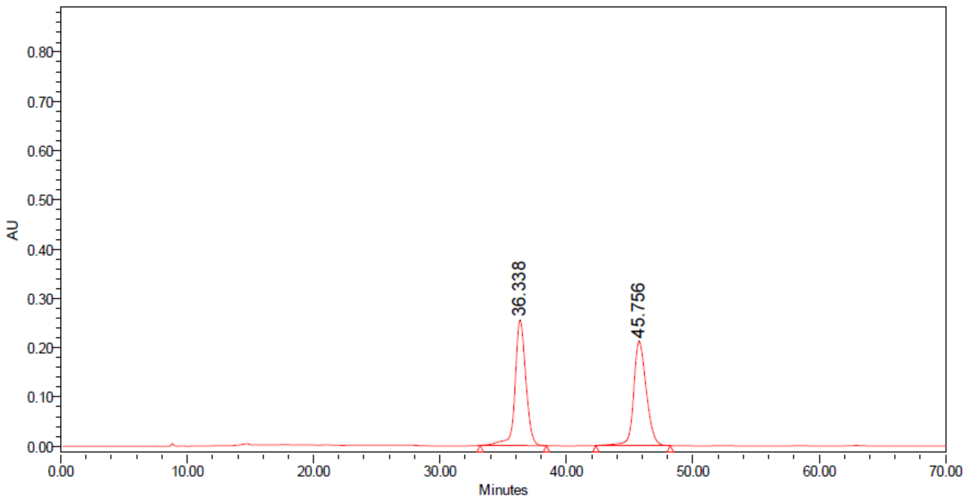

	RT	Area	% Area	Height	% Height
1	36.338	15022394	50.57	254349	54.60
2	45.756	14683098	49.43	211479	45.40

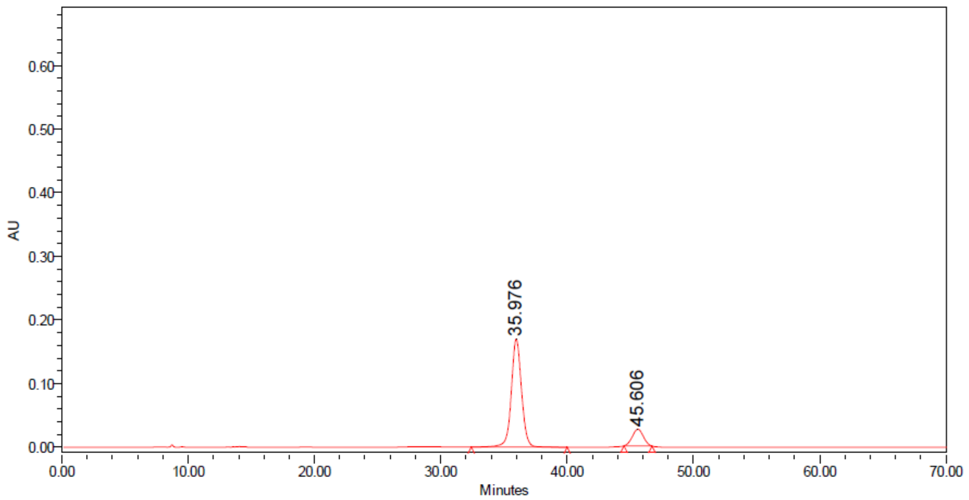

	RT	Area	% Area	Height	% Height
1	35.976	9571699	85.71	169802	86.56
2	45.606	1595576	14.29	26363	13.44

Supplementary Figure 142. HPLC spectra of 3f

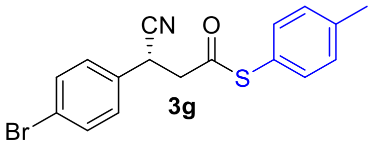

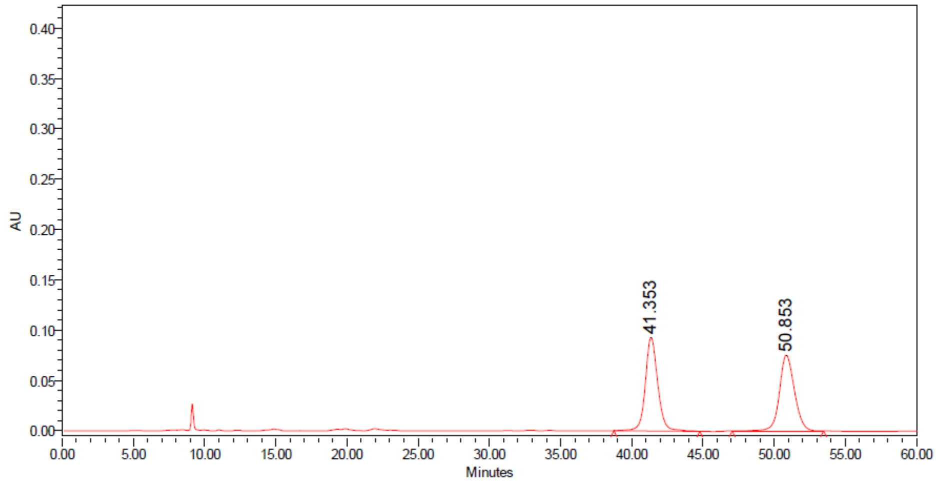

	RT	Area	% Area	Height	% Height
1	41.353	5618211	50.47	92944	55.26
2	50.853	5512826	49.53	75239	44.74

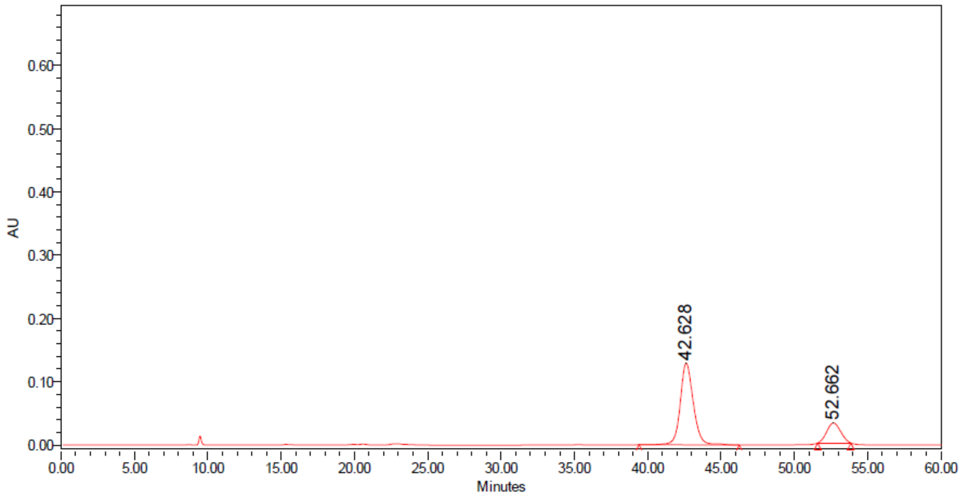

	RT	Area	% Area	Height	% Height
1	42.628	8020599	79.46	129230	80.28
2	52.662	2073209	20.54	31740	19.72

Supplementary Figure 143. HPLC spectra of 3g

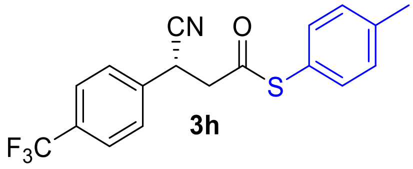

3h

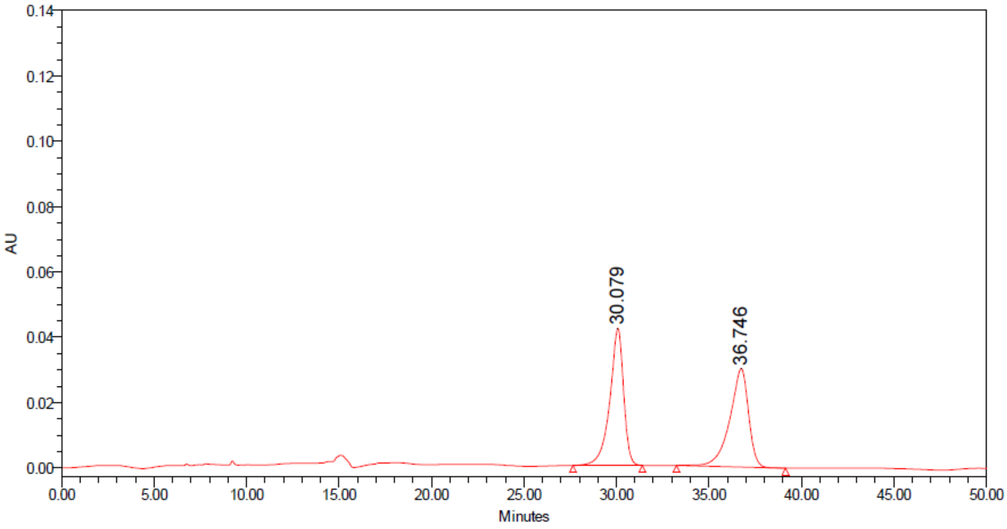

	RT	Area	% Area	Height	% Height
1	30.079	2173146	50.70	42000	58.23
2	36.746	2113540	49.30	30132	41.77

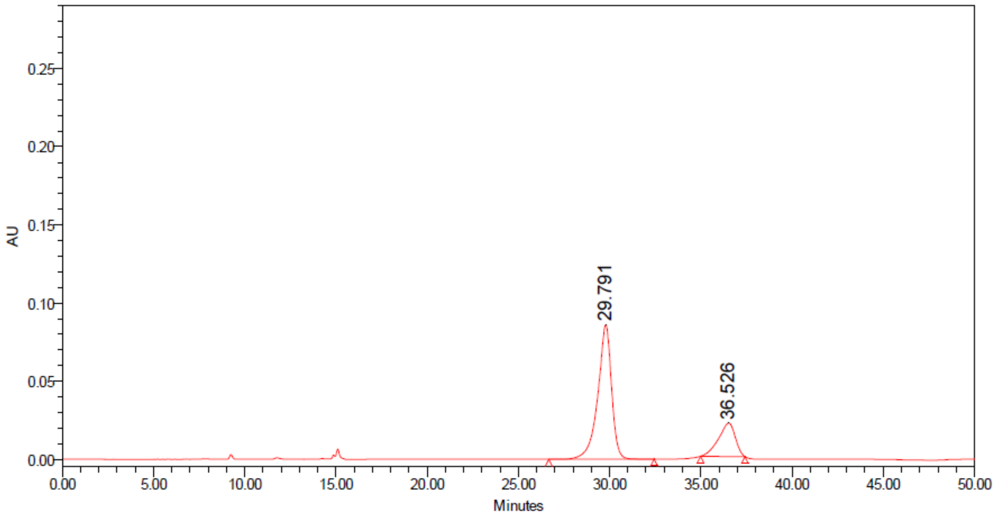

	RT	Area	% Area	Height	% Height
1	29.791	4493486	76.68	85993	80.04
2	36.526	1366224	23.32	21440	19.96

Supplementary Figure 144. HPLC spectra of 3h

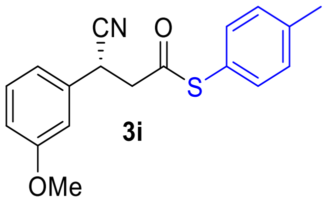

3i

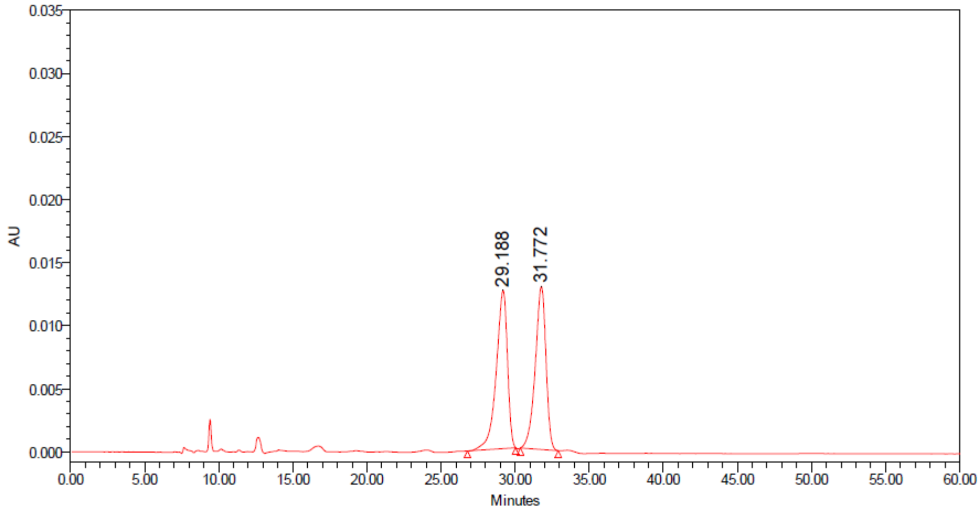

	RT	Area	% Area	Height	% Height
1	29.188	657333	50.30	12557	49.25
2	31.772	649567	49.70	12938	50.75

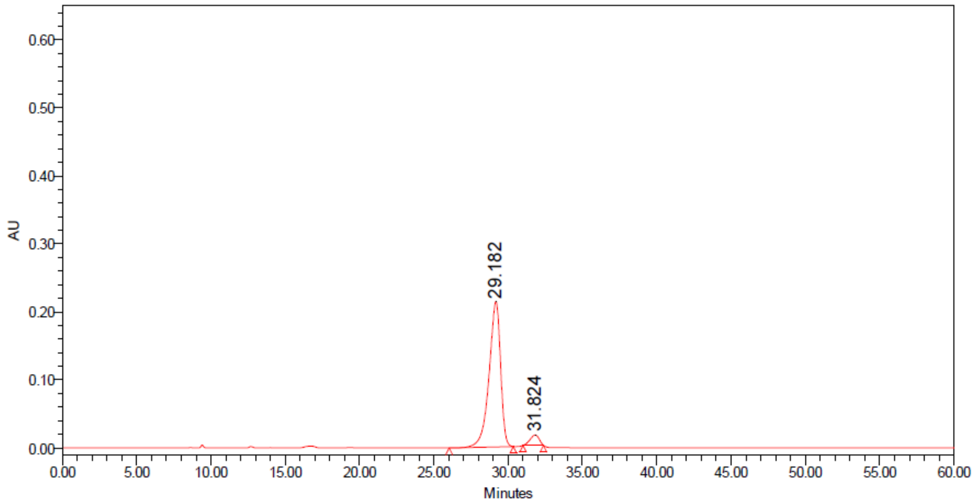

	RT	Area	% Area	Height	% Height
1	29.182	11476971	94.67	214128	93.31
2	31.824	646483	5.33	15362	6.69

Supplementary Figure 145. HPLC spectra of 3i

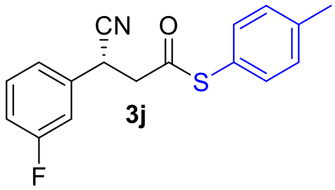

3j

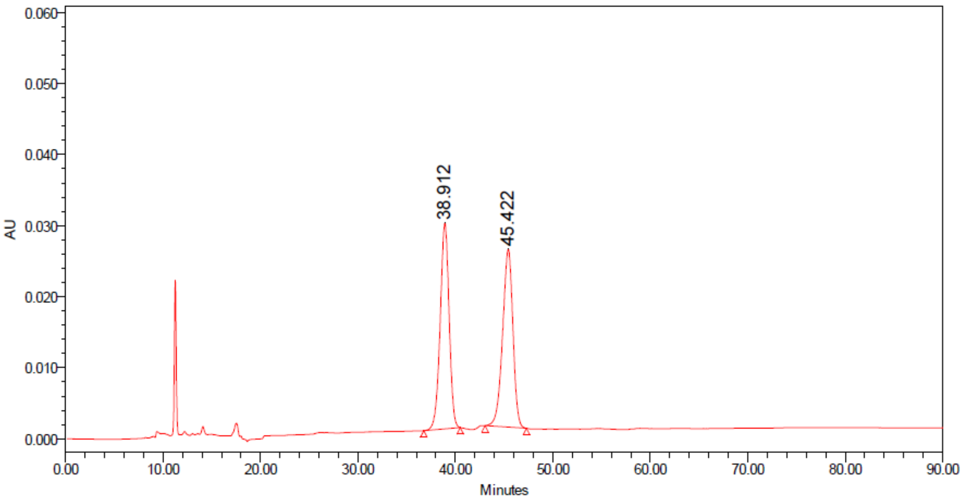

	RT	Area	% Area	Height	% Height
1	38.912	1844091	49.97	28958	53.62
2	45.422	1846369	50.03	25049	46.38

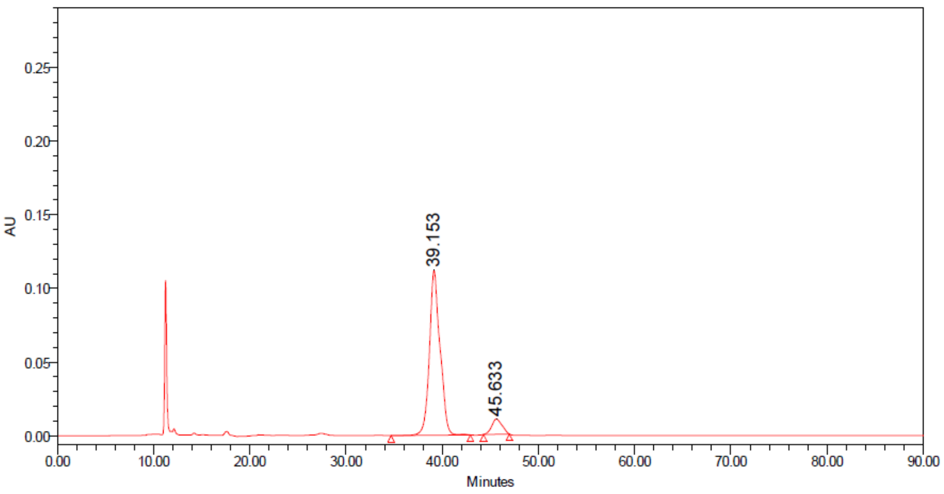

	RT	Area	% Area	Height	% Height
1	39.153	8585065	91.73	111758	91.45
2	45.633	774148	8.27	10445	8.55

Supplementary Figure 146. HPLC spectra of 3j

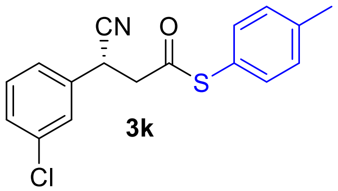

3k

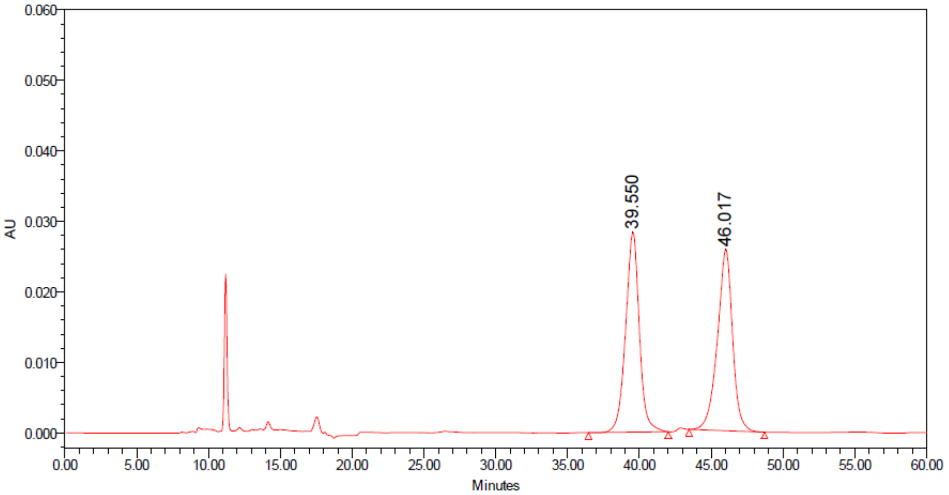

	RT	Area	% Area	Height	% Height
1	39.550	1882755	49.96	28377	52.46
2	46.017	1886103	50.04	25720	47.54

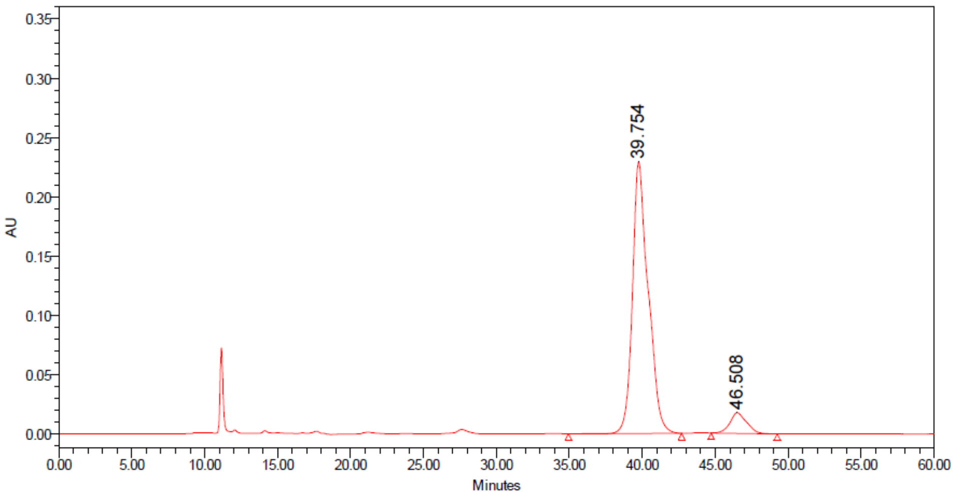

	RT	Area	% Area	Height	% Height
1	39.754	17166653	92.56	229090	92.91
2	46.508	1380253	7.44	17472	7.09

Supplementary Figure 147. HPLC spectra of 3k

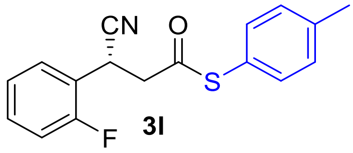

3l

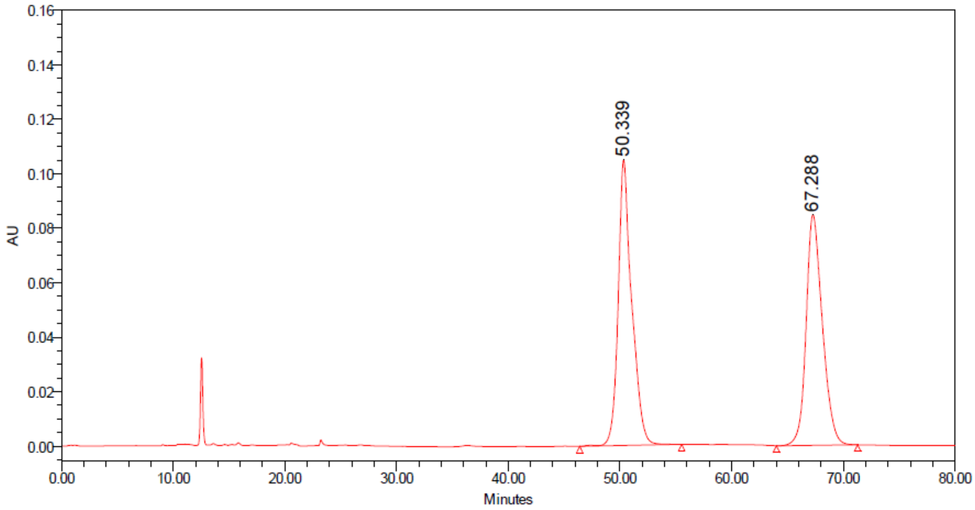

	RT	Area	% Area	Height	% Height
1	50.339	8866918	50.86	104878	55.30
2	67.288	8568217	49.14	84763	44.70

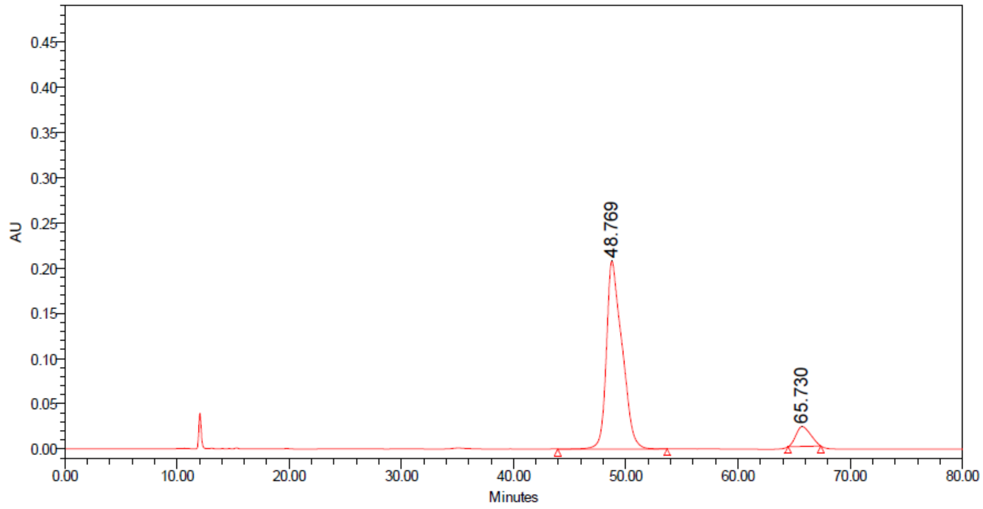

	RT	Area	% Area	Height	% Height
1	48.769	20077070	91.00	207914	90.40
2	65.730	1984544	9.00	22069	9.60

Supplementary Figure 148. HPLC spectra of 3l

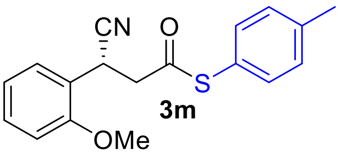

3m

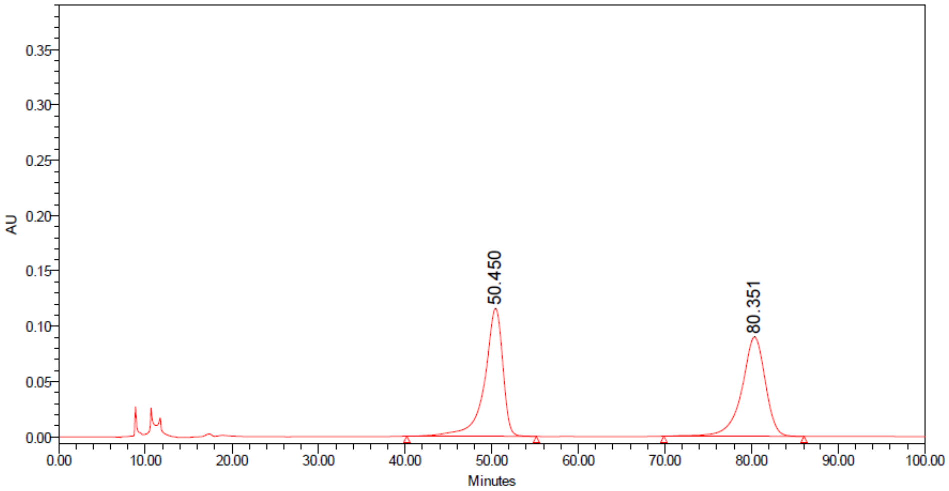

	RT	Area	% Area	Height	% Height
1	50.450	16904318	50.06	115640	56.30
2	80.351	16864284	49.94	89758	43.70

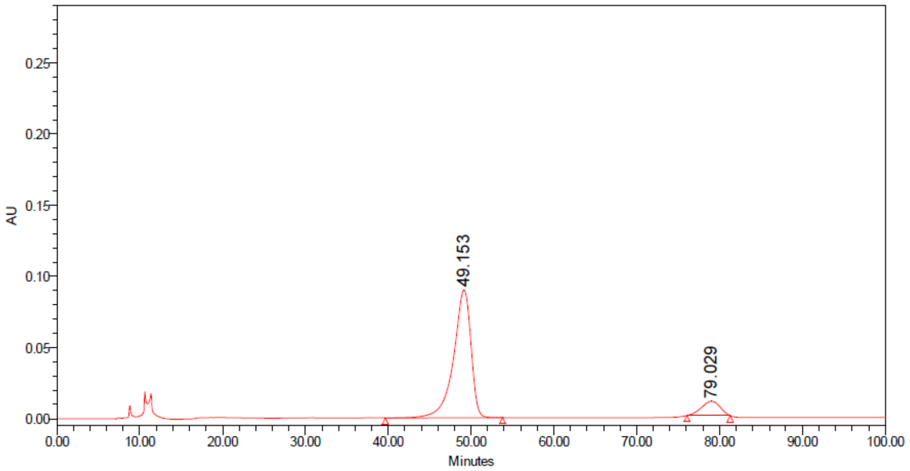

	RT	Area	% Area	Height	% Height
1	49.153	13117235	89.31	89718	89.83
2	79.029	1570305	10.69	10157	10.17

Supplementary Figure 149. HPLC spectra of 3m

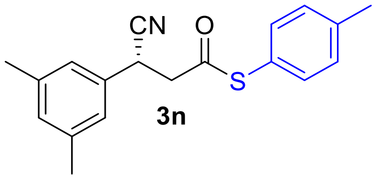

3n

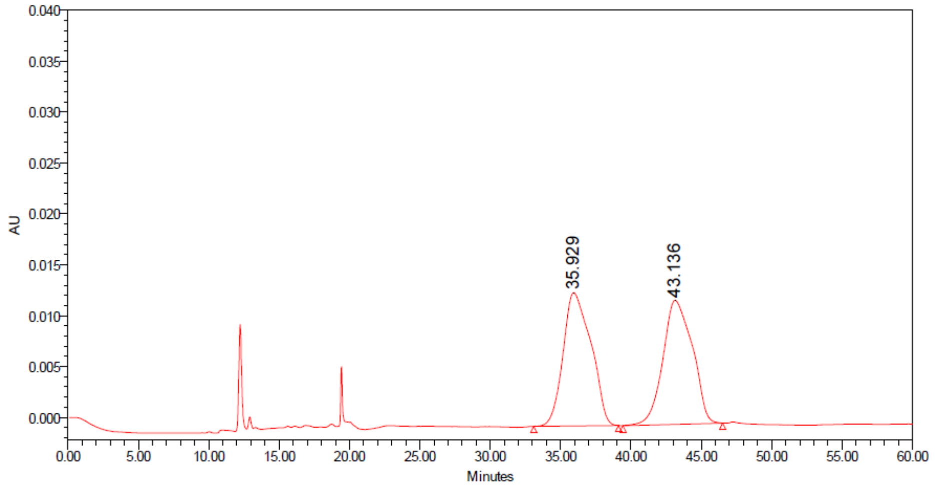

	RT	Area	% Area	Height	% Height
1	35.929	1807082	50.49	13104	51.83
2	43.136	1772327	49.51	12181	48.17

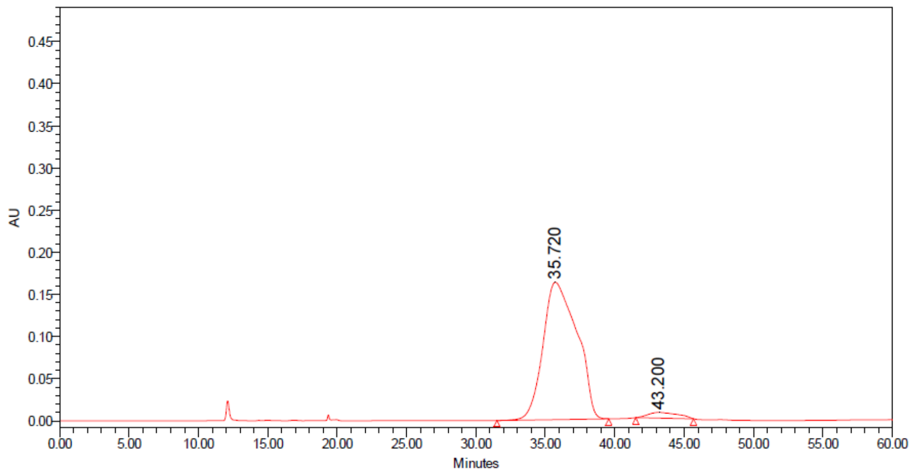

	RT	Area	% Area	Height	% Height
1	35.720	27743837	96.45	162912	96.04
2	43.200	1020560	3.55	6723	3.96

Supplementary Figure 150. HPLC spectra of 3n

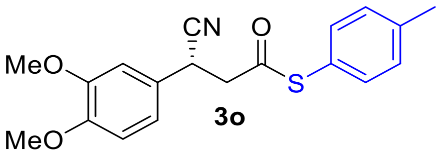

3o

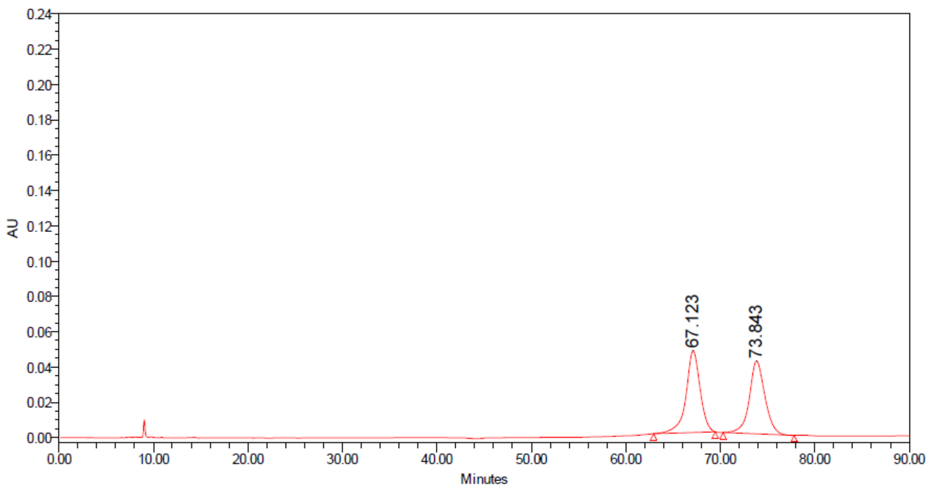

	RT	Area	% Area	Height
1	67.123	4806698	50.43	46285
2	73.843	4725034	49.57	41284

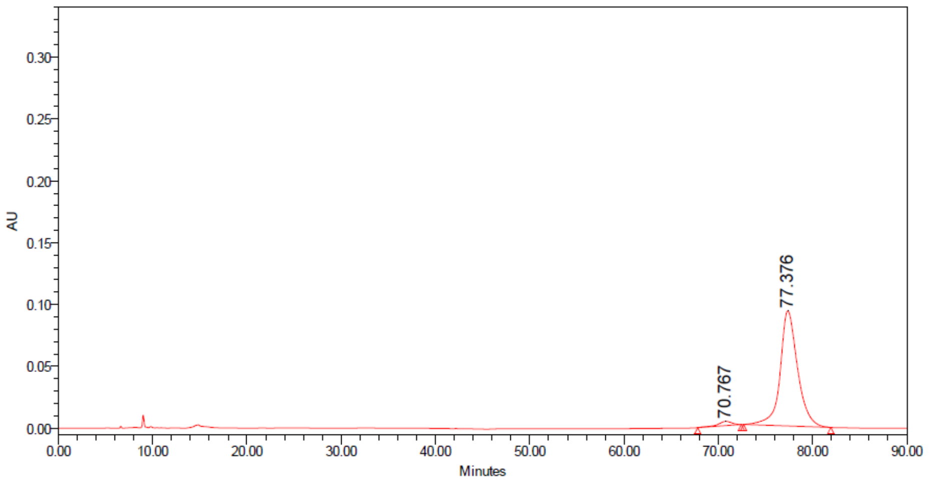

	RT	Area	% Area	Height
1	70.767	327864	2.67	3385
2	77.376	11961251	97.33	93002

Supplementary Figure 151. HPLC spectra of 3o

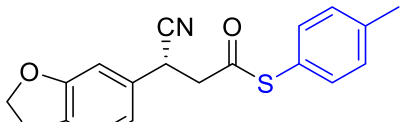

3p

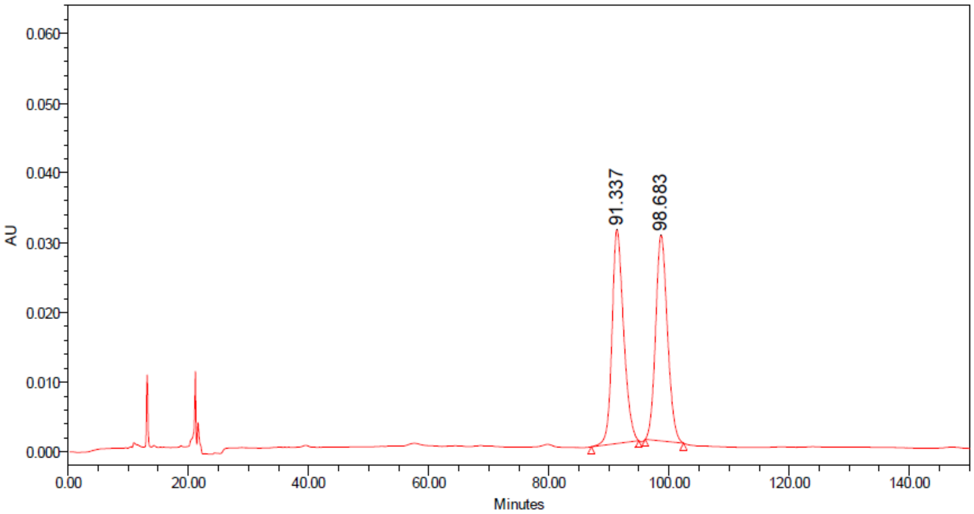

	RT	Area	% Area	Height	% Height
1	91.337	4115496	50.52	30722	50.96
2	98.683	4030363	49.48	29568	49.04

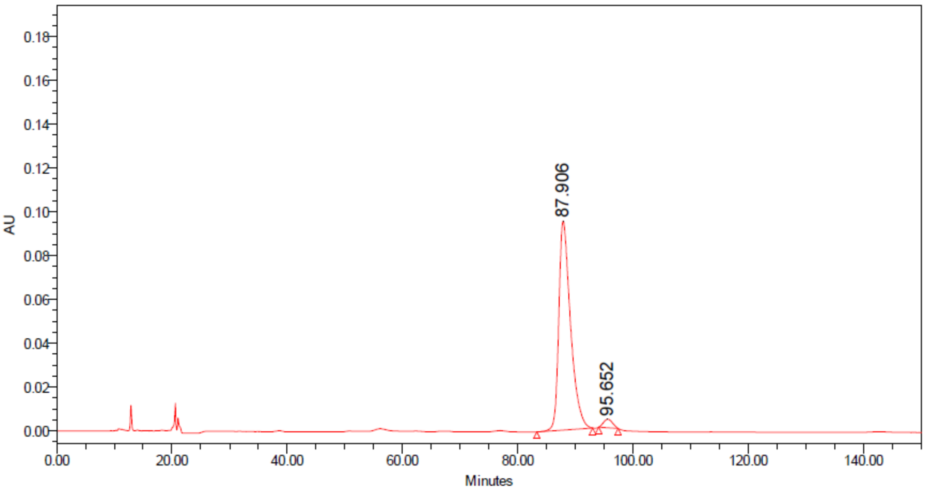

	RT	Area	% Area	Height	% Height
1	87.906	13432796	96.88	95479	96.02
2	95.652	432596	3.12	3958	3.98

Supplementary Figure 152. HPLC spectra of 3p

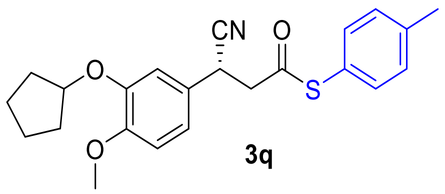

3q

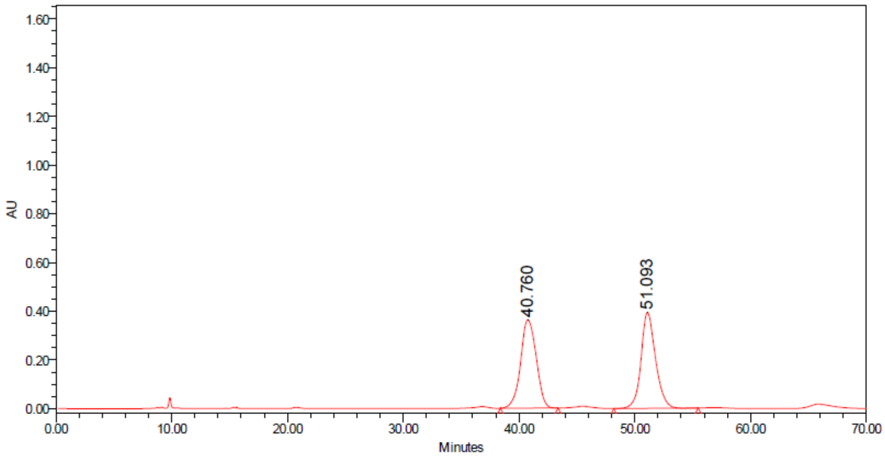

	RT	Area	% Area	Height	% Height
1	40.760	34073529	49.89	363122	47.99
2	51.093	34222207	50.11	393607	52.01

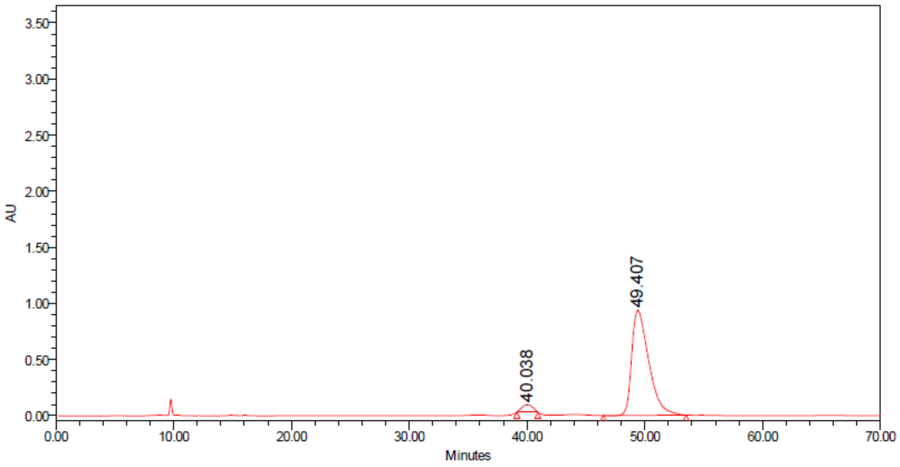

	RT	Area	% Area	Height	% Height
1	40.038	4302995	4.37	66568	6.66
2	49.407	94227223	95.63	932995	93.34

Supplementary Figure 153. HPLC spectra of 3q

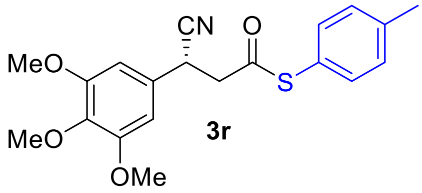

3r

<Chromatogram>

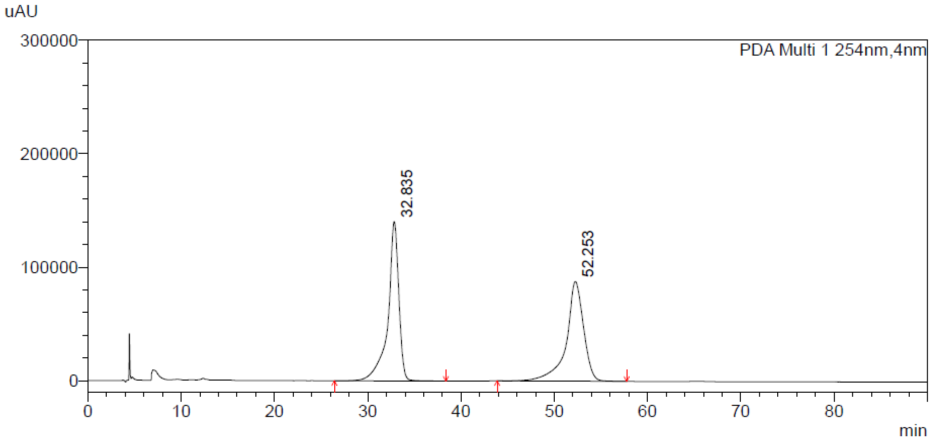

<Peak Table>

PDA Ch1 254nm

Peak#	Ret. Time	Area	Height	Area%	Height%
1	32.835	11473242	140536	50.421	61.546
2	52.253	11281757	87806	49.579	38.454
Total		22754999	228342	100.000	100.000

<Chromatogram>

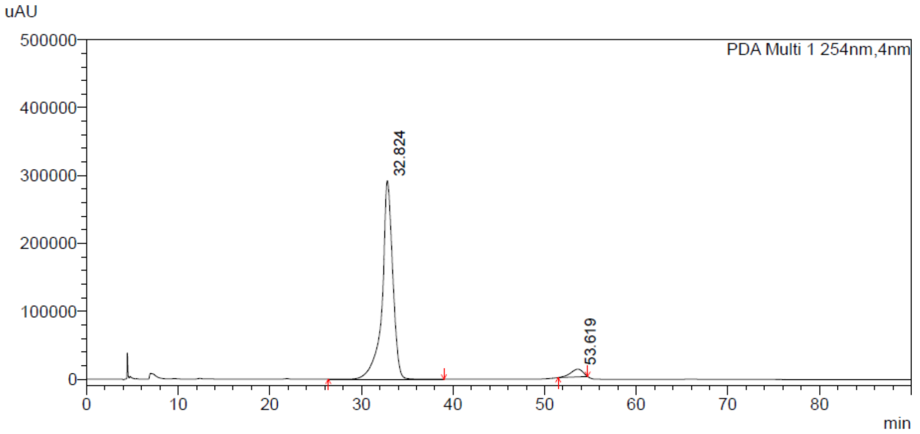

<Peak Table>

PDA Ch1 254nm

Peak#	Ret. Time	Area	Height	Area%	Height%
1	32.824	23579144	292368	95.785	96.249
2	53.619	1037587	11393	4.215	3.751
Total		24616731	303761	100.000	100.000

Supplementary Figure 154. HPLC spectra of 3r

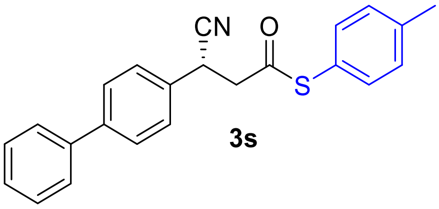

3s

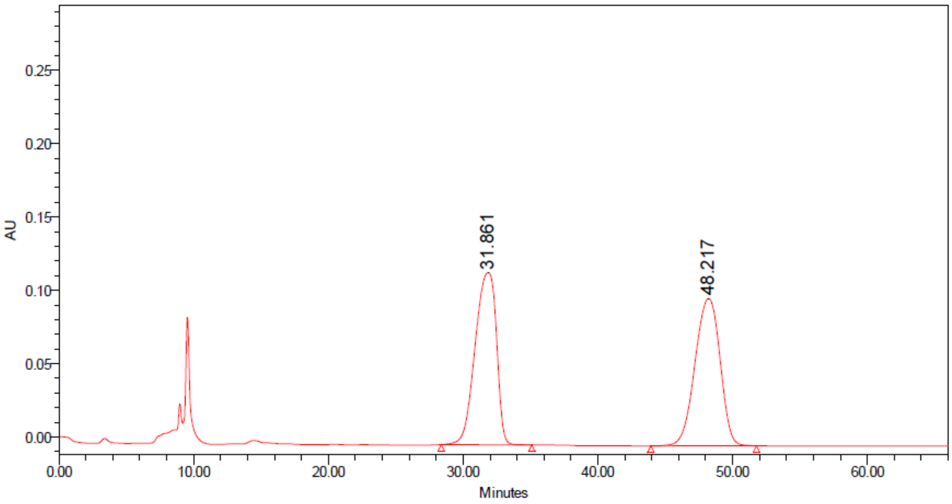

	RT	Area	% Area	Height	% Height
1	31.861	12928833	49.96	117432	53.92
2	48.217	12950859	50.04	100372	46.08

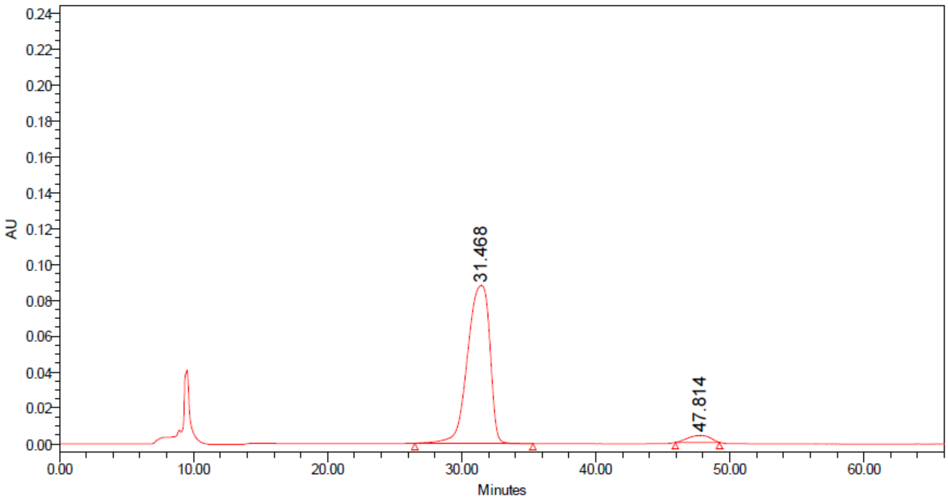

	RT	Area	% Area	Height	% Height
1	31.468	10123258	95.76	87932	95.76
2	47.814	447809	4.24	3891	4.24

Supplementary Figure 155. HPLC spectra of 3s

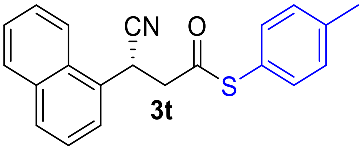

3t

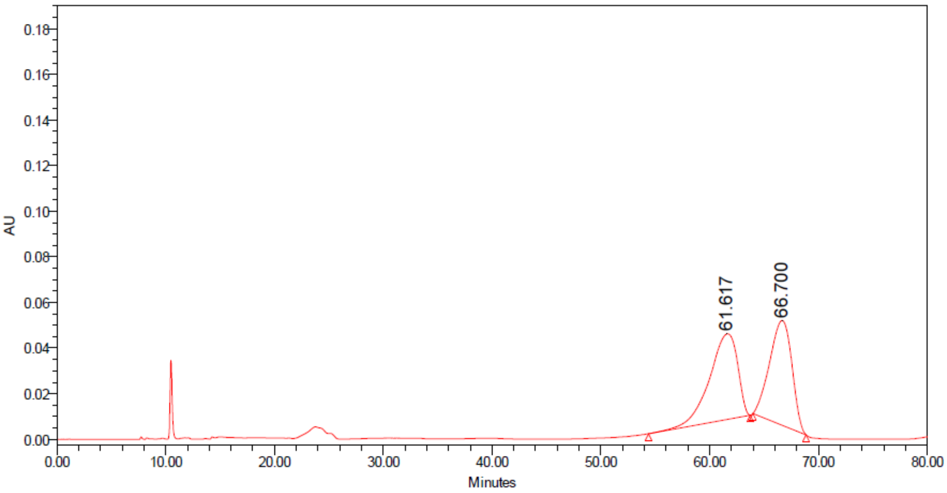

	RT	Area	% Area	Height	% Height
1	61.617	6706237	50.68	37467	44.91
2	66.700	6527023	49.32	45968	55.09

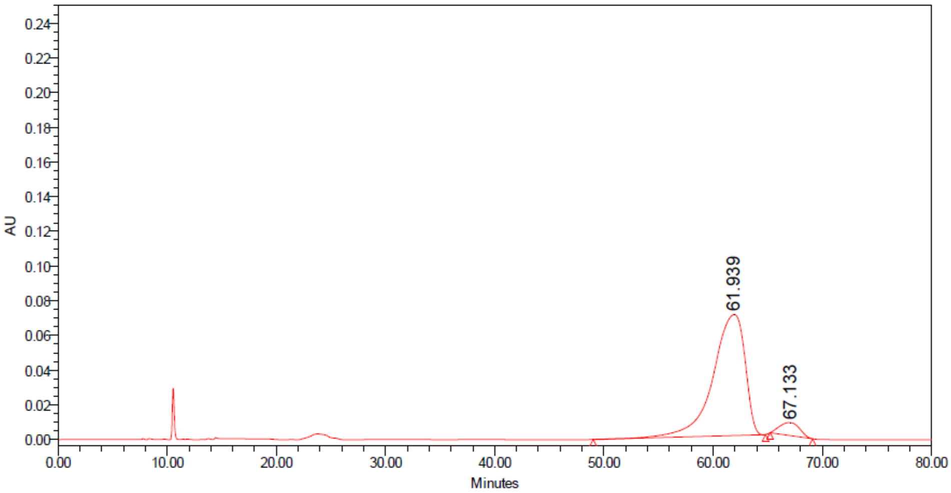

	RT	Area	% Area	Height	% Height
1	61.939	14207784	93.78	69731	90.26
2	67.133	942306	6.22	7522	9.74

Supplementary Figure 156. HPLC spectra of 3t

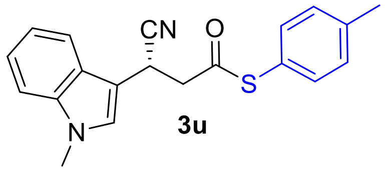

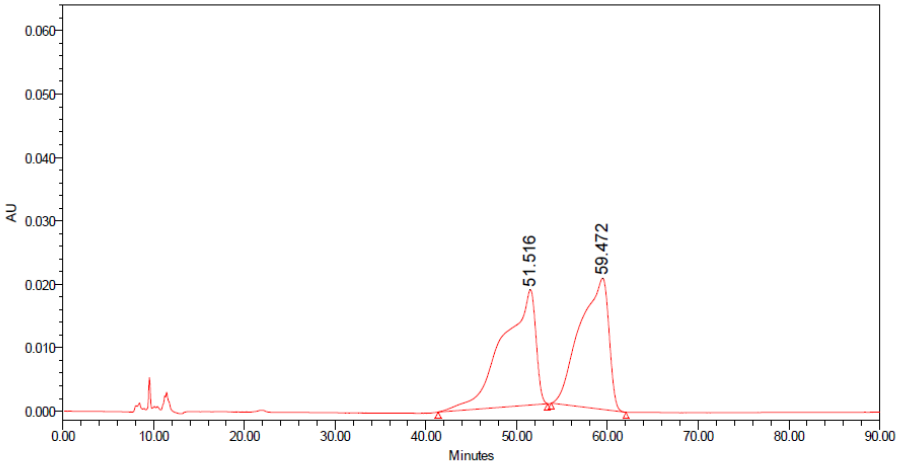

	RT	Area	% Area	Height	% Height
1	51.516	4576749	50.20	18184	46.74
2	59.472	4540588	49.80	20718	53.26

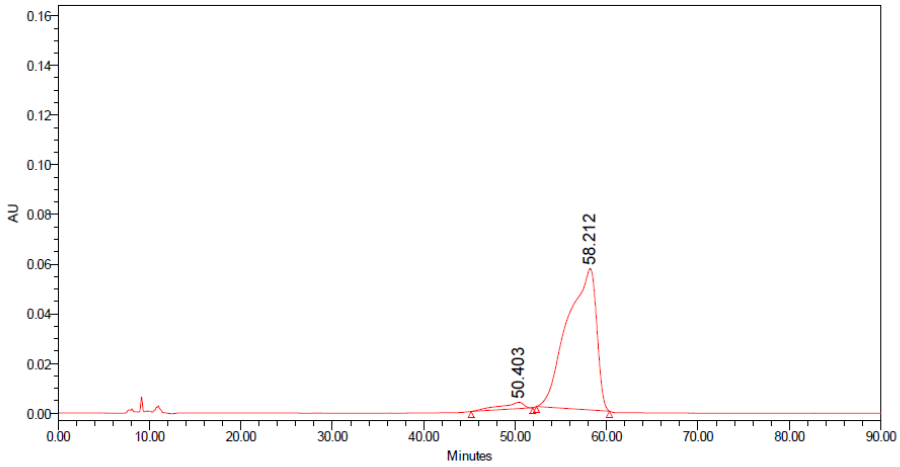

	RT	Area	% Area	Height	% Height
1	50.403	468448	3.60	2571	4.32
2	58.212	12559906	96.40	56910	95.68

Supplementary Figure 157. HPLC spectra of 3u

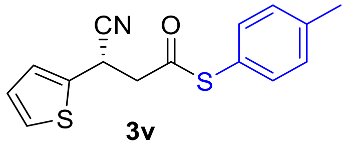

3v

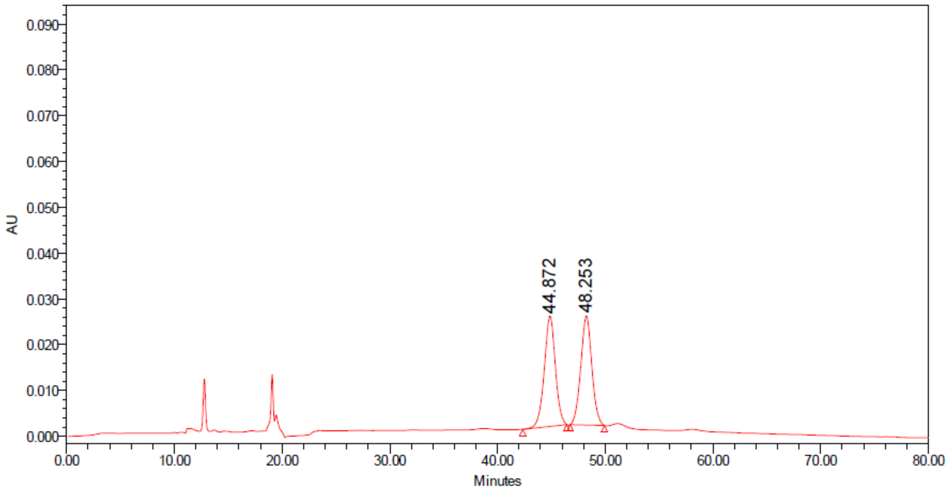

	RT	Area	% Area	Height	% Height
1	44.872	1723799	50.37	24049	50.28
2	48.253	1698608	49.63	23783	49.72

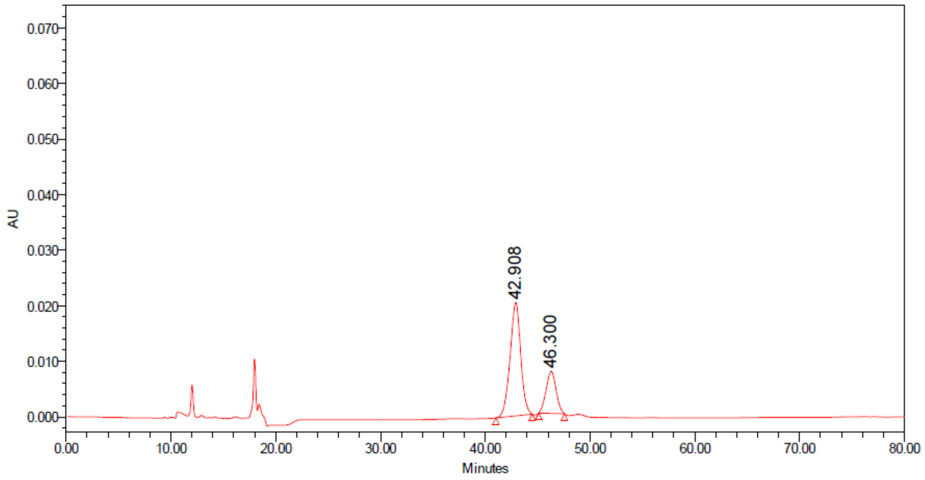

	RT	Area	% Area	Height	% Height
1	42.908	1439561	74.24	20368	72.88
2	46.300	499610	25.76	7579	27.12

Supplementary Figure 158. HPLC spectra of 3v

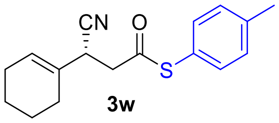

3w

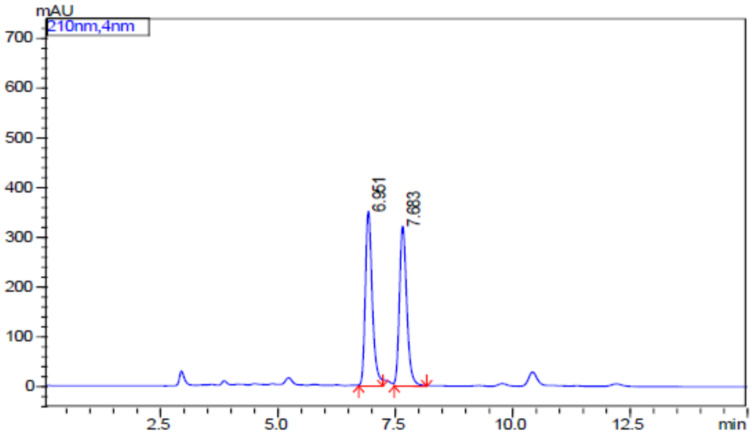

<Column Performance Report>

Peak No.	Time	Area	Area %	Plate number	Tailing	Resolution
1	6.951	3582720	50.925	10174	1.352	--
2	7.683	3452587	49.075	11083	1.238	2.579

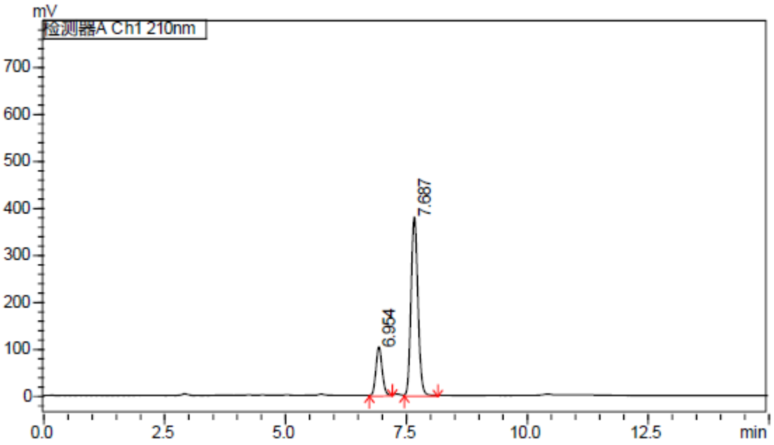

<Column Performance Report>

Peak No.	Time	Area	Area %	Plate number	Tailing	Resolution
1	6.954	912443	19.587	13041	1.104	--
2	7.687	3745916	80.413	13133	1.114	2.862

Supplementary Figure 159. HPLC spectra of 3w

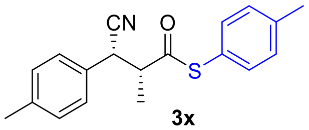

3x

< Chromatogram >

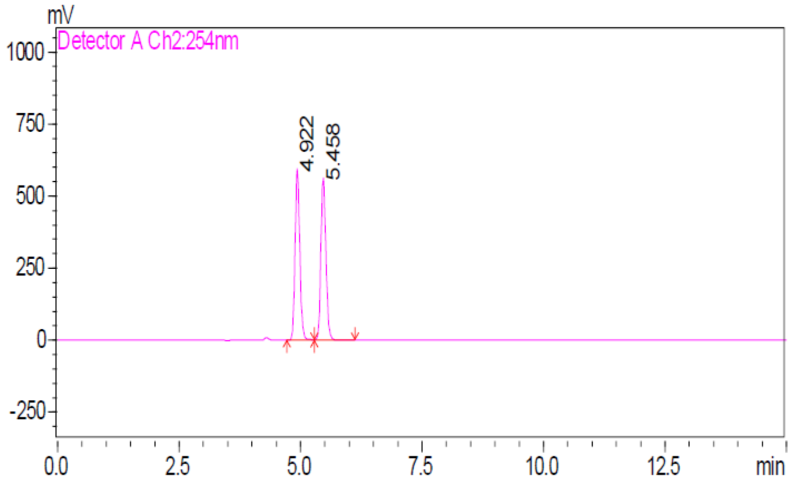

<Column Performance Report>

Peak No.	Time	Area	Area %	Plate number	Tailing	Resolution
1	4.922	4057783	49.8123	9401.629	1.285	--
2	5.458	4088364	50.1877	10419.522	1.235	2.569

< Chromatogram >

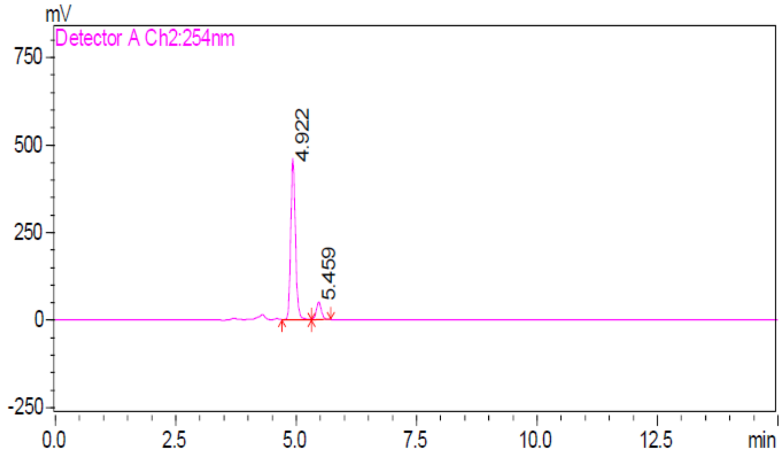

<Column Performance Report>

Peak No.	Time	Area	Area %	Plate number	Tailing	Resolution
1	4.922	3144758	89.6603	9481.568	1.297	--
2	5.459	362656	10.3397	9900.129	1.171	2.549

Supplementary Figure 160. HPLC spectra of 3x

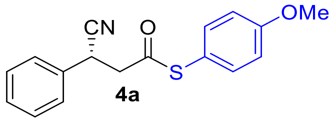

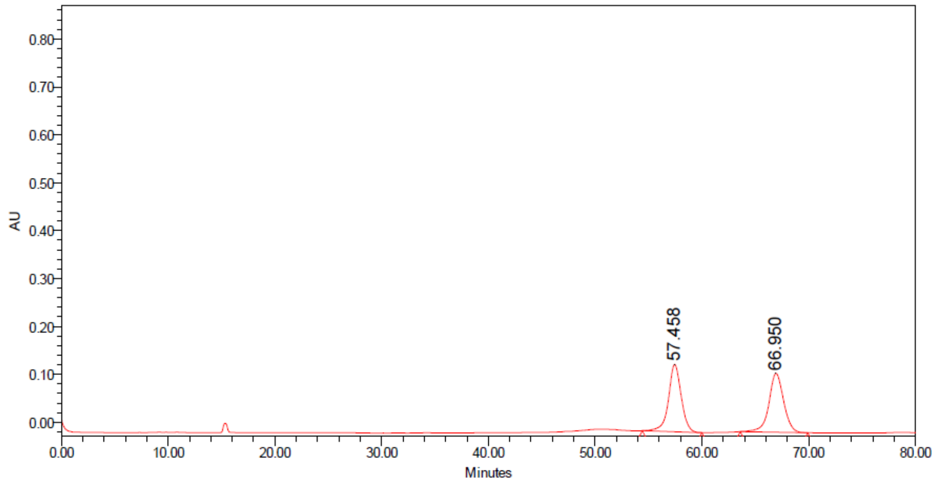

	RT	Area	% Area	Height	% Height
1	57.458	11625238	49.68	140639	53.37
2	66.950	11775456	50.32	122875	46.63

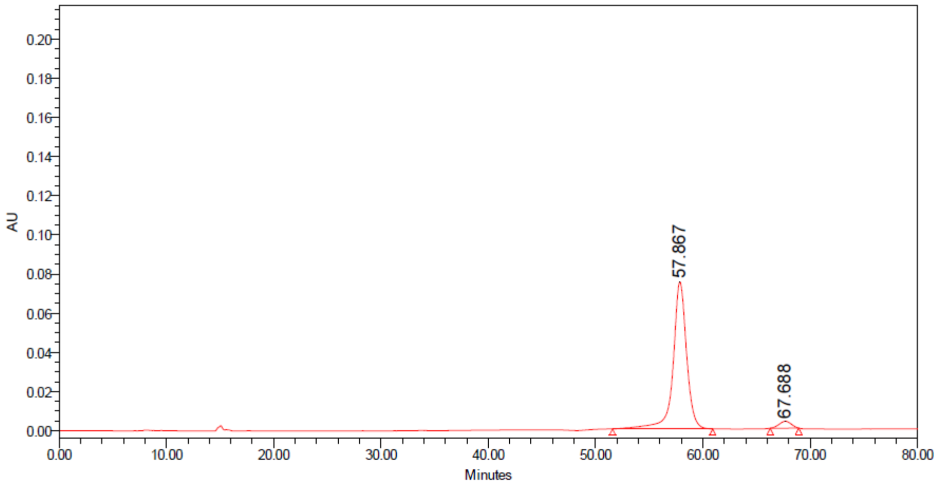

	RT	Area	% Area	Height	% Height
1	57.867	6586556	96.00	75138	95.53
2	67.688	274469	4.00	3517	4.47

Supplementary Figure 161. HPLC spectra of 4a

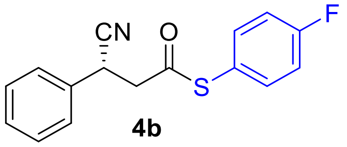

4b

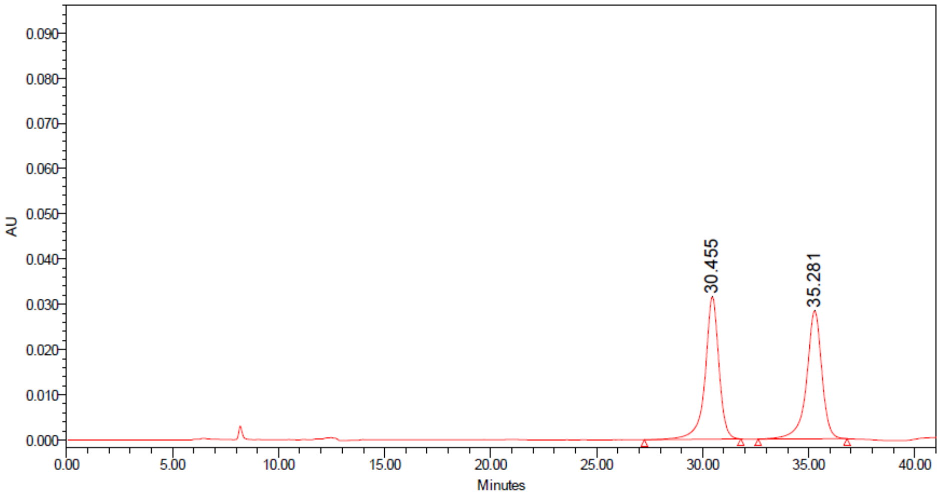

	RT	Area	% Area	Height	% Height
1	30.455	1426176	50.23	31463	52.63
2	35.281	1412893	49.77	28324	47.37

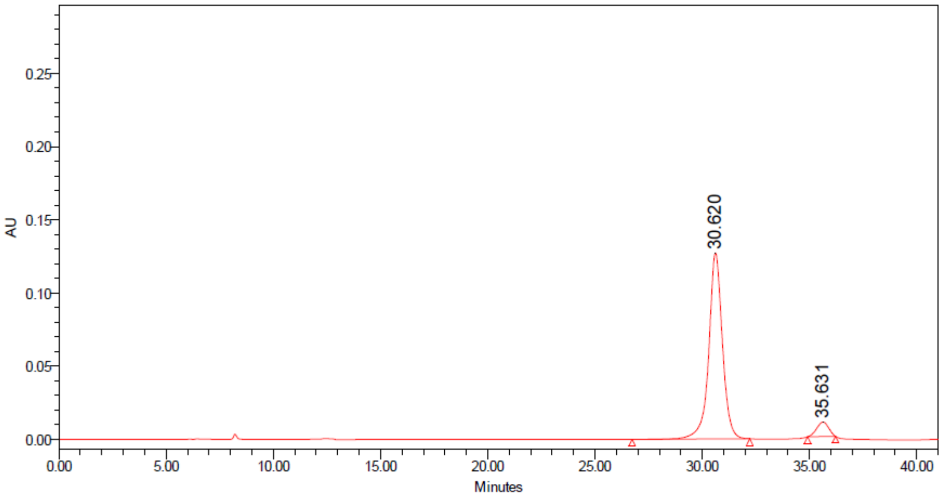

	RT	Area	% Area	Height	% Height
1	30.620	5625465	93.65	126981	92.77
2	35.631	381427	6.35	9891	7.23

Supplementary Figure 162. HPLC spectra of 4b

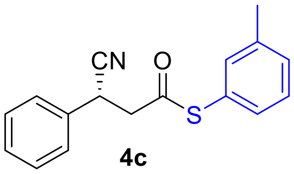

4c

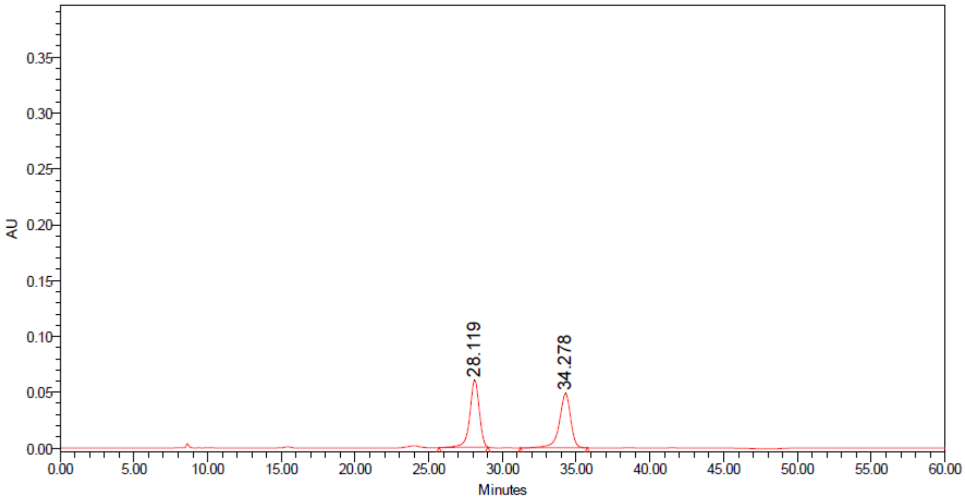

	RT	Area	% Area	Height	% Height
1	28.119	2659781	50.59	60006	55.25
2	34.278	2597518	49.41	48607	44.75

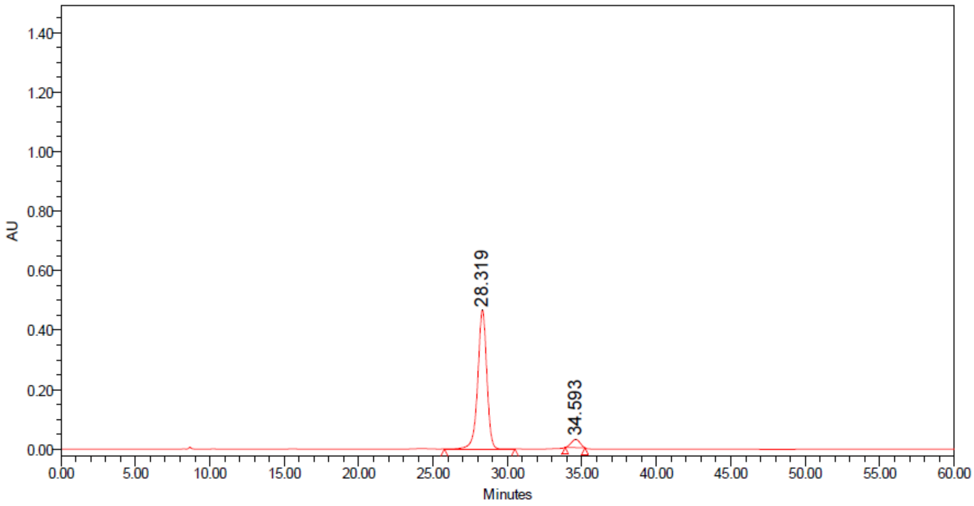

	RT	Area	% Area	Height	% Height
1	28.319	19710987	94.62	467492	94.42
2	34.593	1120936	5.38	27634	5.58

Supplementary Figure 163. HPLC spectra of 4c

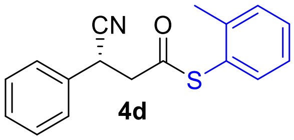

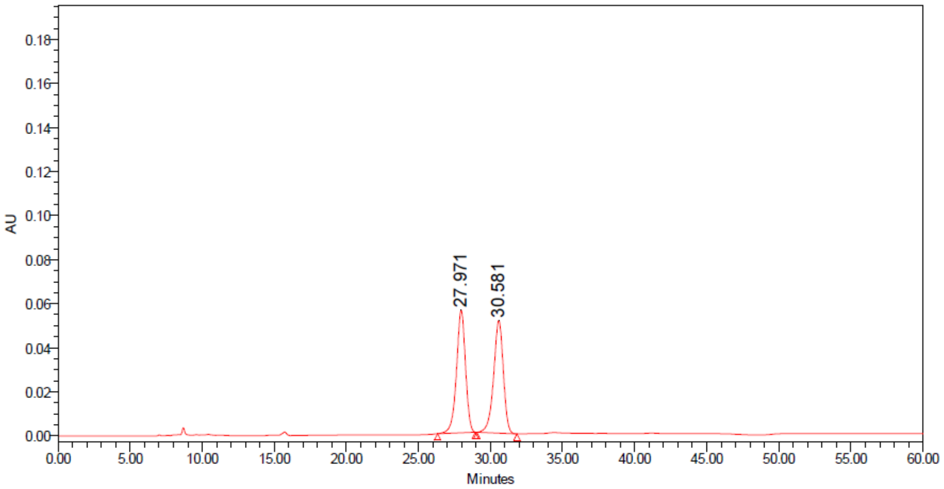

	RT	Area	% Area	Height	% Height
1	27.971	2415253	50.12	55662	52.09
2	30.581	2404083	49.88	51194	47.91

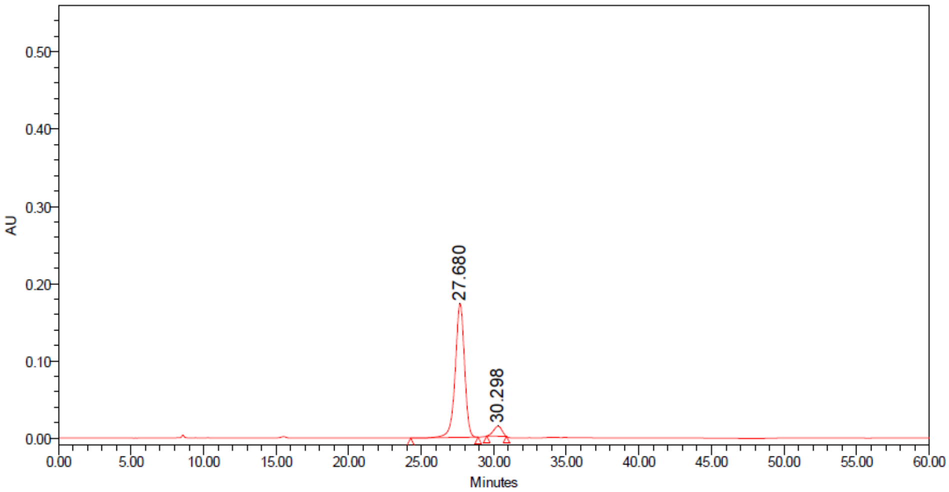

	RT	Area	% Area	Height	% Height
1	27.680	7713459	93.66	173524	92.99
2	30.298	521975	6.34	13086	7.01

Supplementary Figure 164. HPLC spectra of 4d

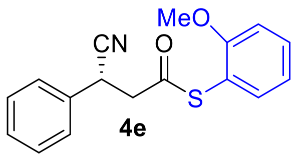

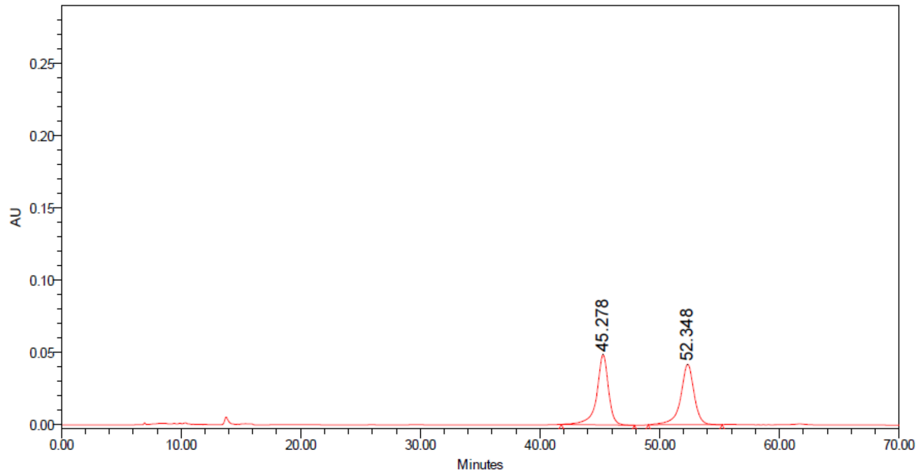

	RT	Area	% Area	Height	% Height
1	45.278	3307704	50.35	48625	53.78
2	52.348	3262252	49.65	41794	46.22

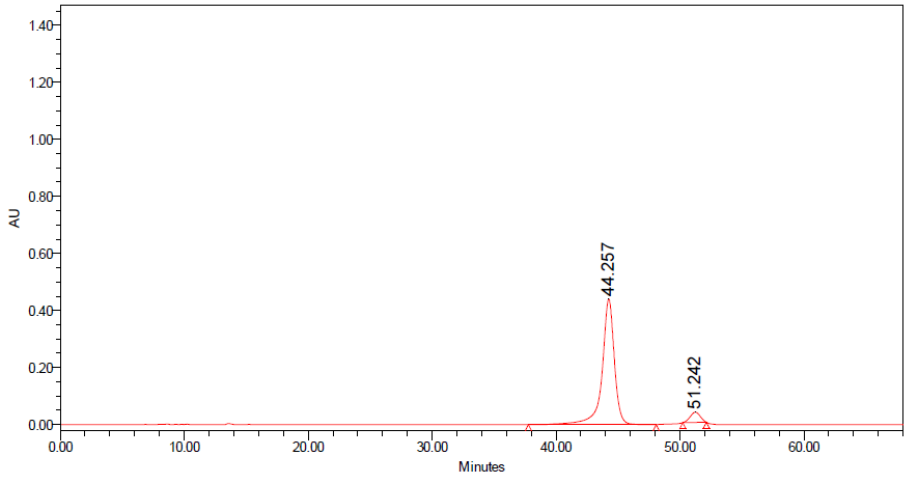

	RT	Area	% Area	Height	% Height
1	44.257	30170540	93.65	440641	92.41
2	51.242	2045411	6.35	36204	7.59

Supplementary Figure 165. HPLC spectra of 4e

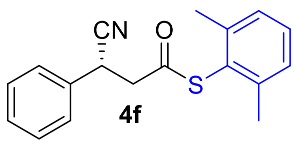

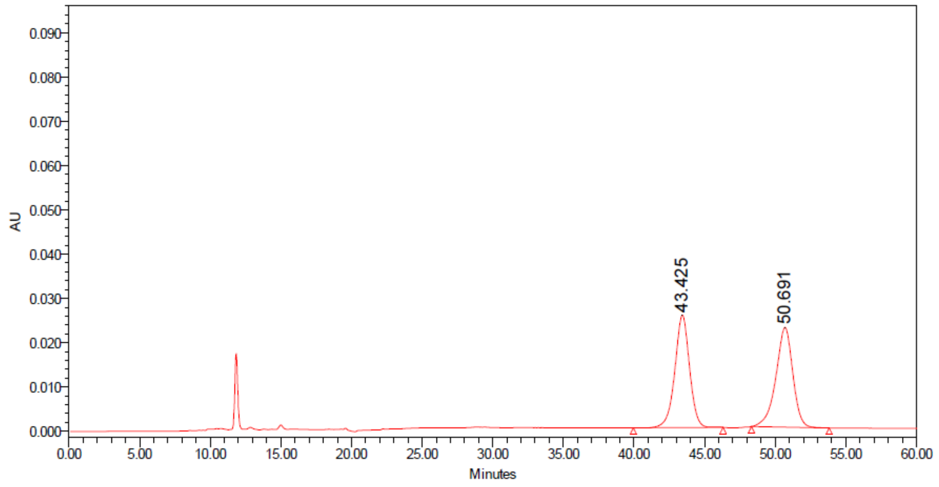

	RT	Area	% Area	Height	% Height
1	43.425	1836941	49.45	25396	52.98
2	50.691	1877921	50.55	22536	47.02

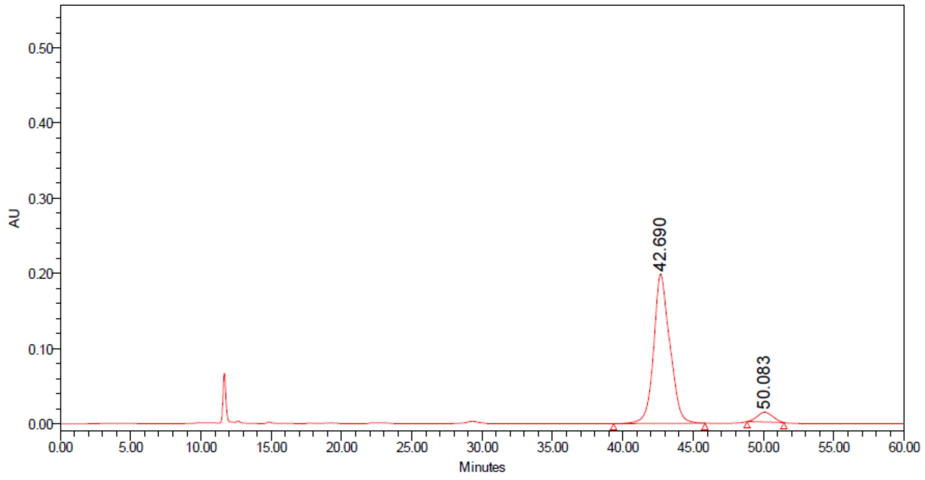

	RT	Area	% Area	Height	% Height
1	42.690	15877605	94.06	198472	93.78
2	50.083	1002522	5.94	13159	6.22

Supplementary Figure 166. HPLC spectra of 4f

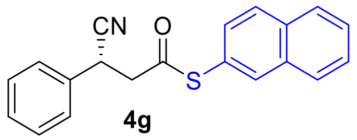

4g

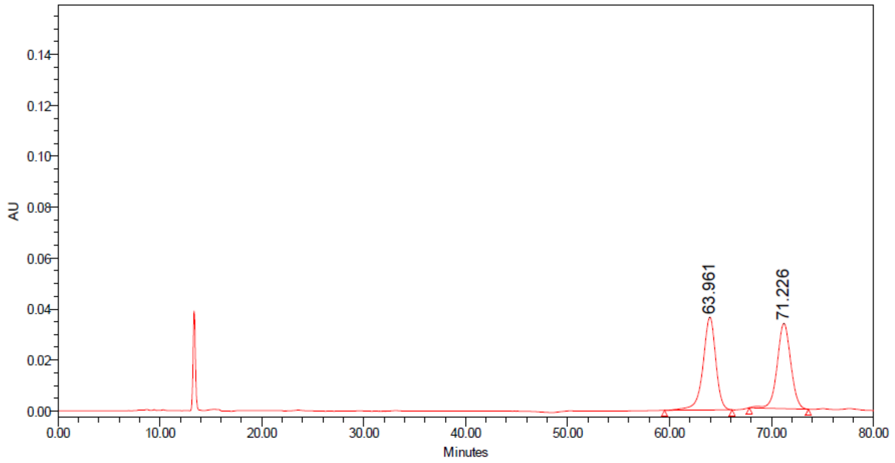

	RT	Area	% Area	Height	% Height
1	63.961	3182286	50.61	36494	52.14
2	71.226	3106176	49.39	33494	47.86

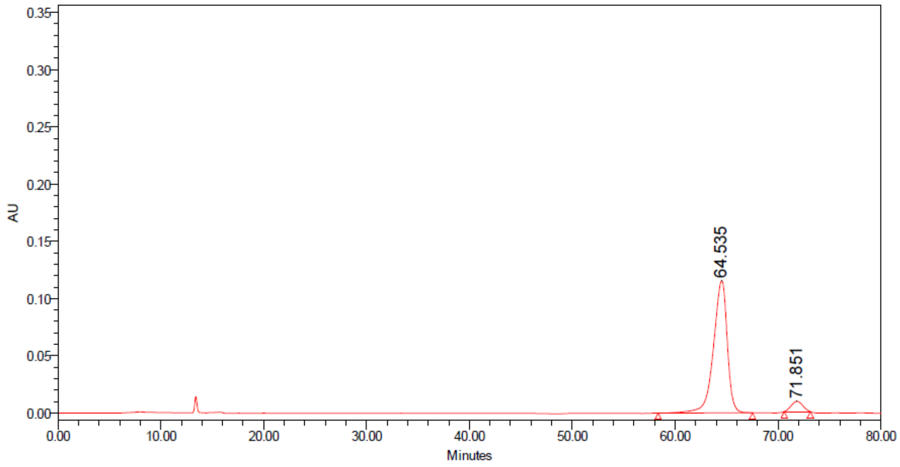

	RT	Area	% Area	Height	% Height
1	64.535	10748174	93.87	115662	92.75
2	71.851	701392	6.13	9042	7.25

Supplementary Figure 167. HPLC spectra of 4g

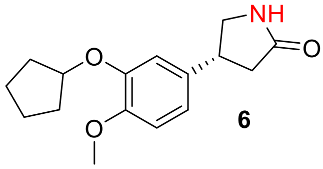

6

<Chromatogram>

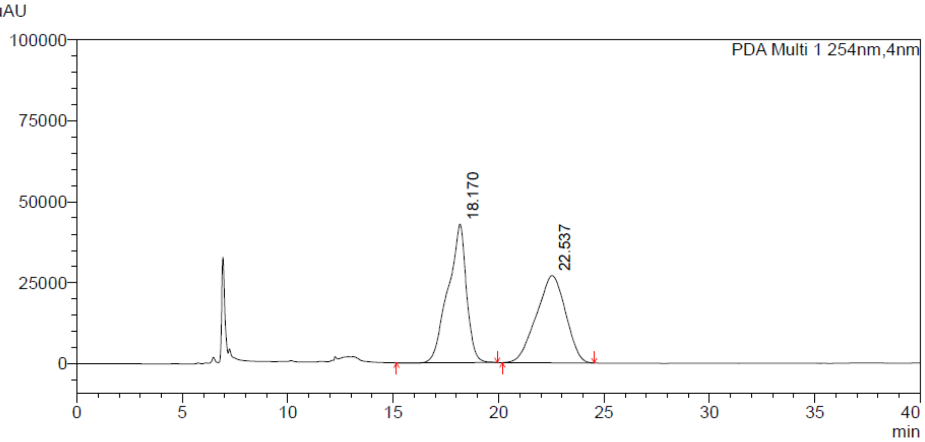

<Peak Table>

PDA Ch1 254nm

Peak#	Ret. Time	Area	Height	Area%	Height%
1	18.170	2594691	42745	49.951	61.343
2	22.537	2599767	26938	50.049	38.657
Total		5194458	69683	100.000	100.000

<Chromatogram>

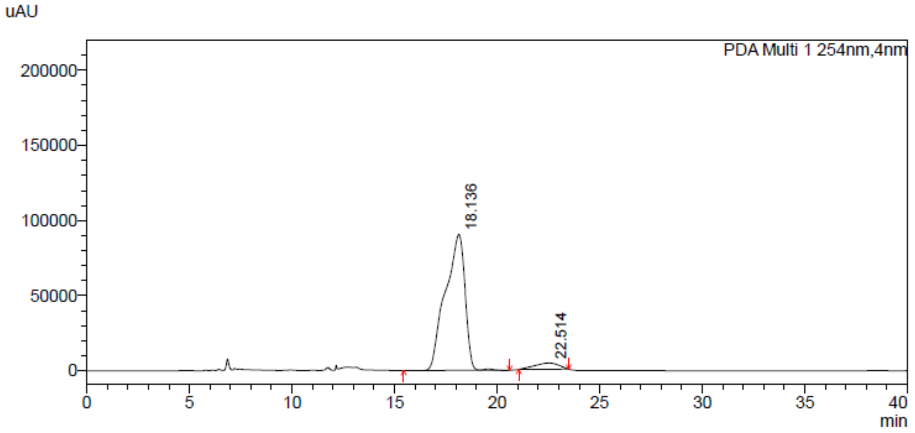

<Peak Table>

PDA Ch1 254nm

Peak#	Ret. Time	Area	Height	Area%	Height%
1	18.136	6041024	90622	94.682	95.558
2	22.514	339327	4213	5.318	4.442
Total		6380351	94835	100.000	100.000

Supplementary Figure 168. HPLC spectra of 6

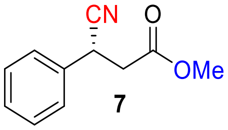

7

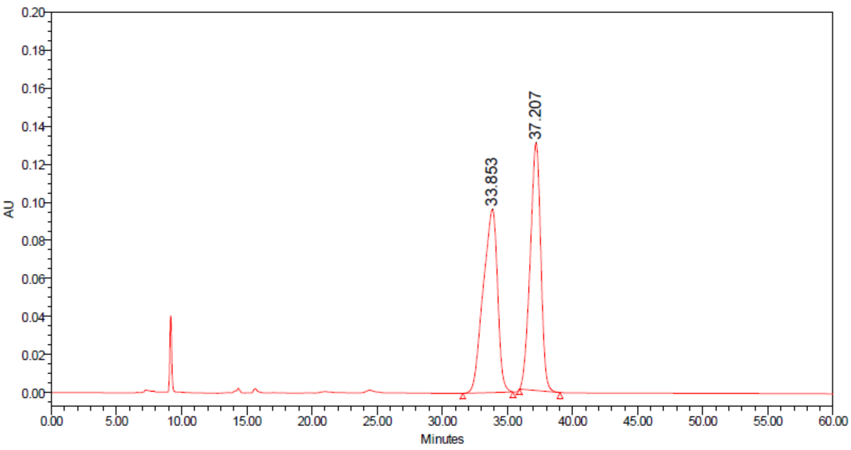

	RT	Area	% Area	Height	% Height
1	33.853	7385103	50.12	96390	42.54
2	37.207	7349326	49.88	130180	57.46

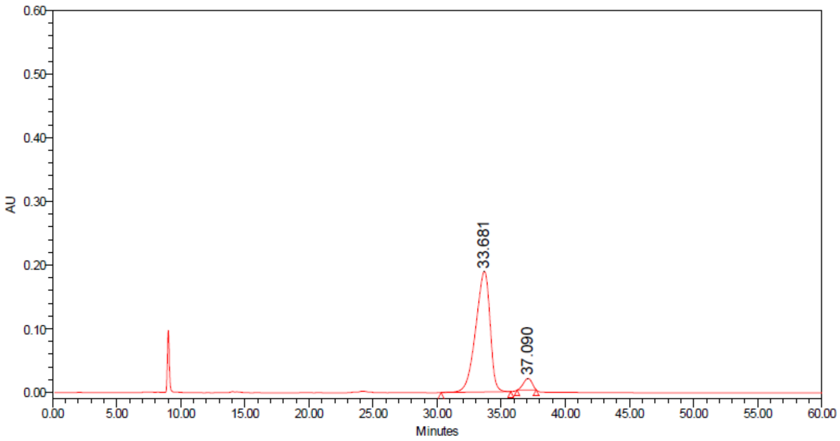

	RT	Area	% Area	Height	% Height
1	33.681	14657401	94.58	189378	91.24
2	37.090	839735	5.42	18184	8.76

Supplementary Figure 169. HPLC spectra of 7

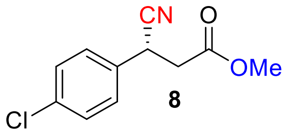

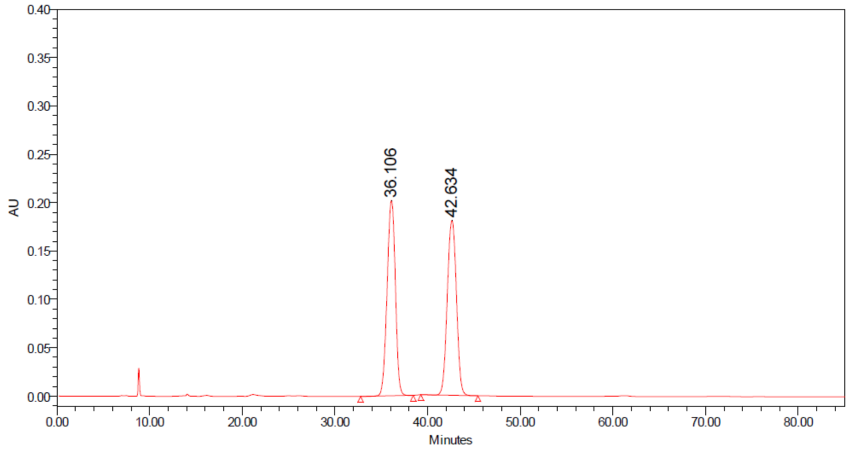

	RT	Area	% Area	Height	% Height
1	36.106	12515468	50.18	202102	52.77
2	42.634	12427052	49.82	180914	47.23

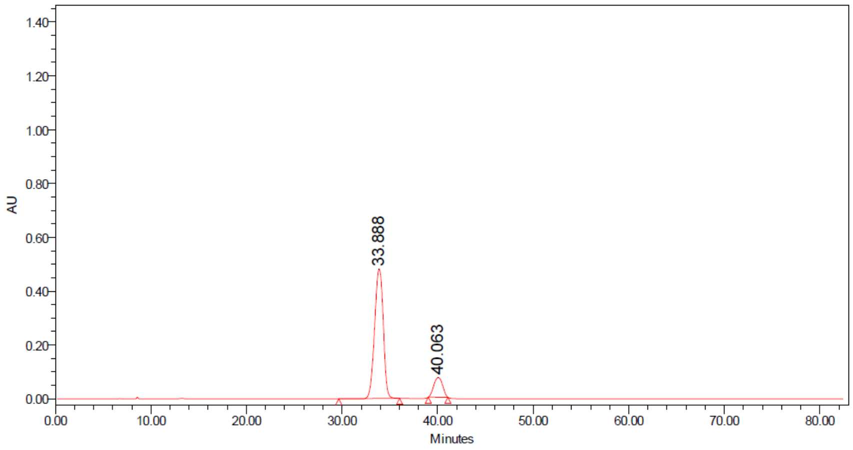

	RT	Area	% Area	Height	% Height
1	33.888	30283123	86.62	481475	86.82
2	40.063	4678868	13.38	73111	13.18

Supplementary Figure 170. HPLC spectra of 8

Scale-Up Synthesis of 3a.

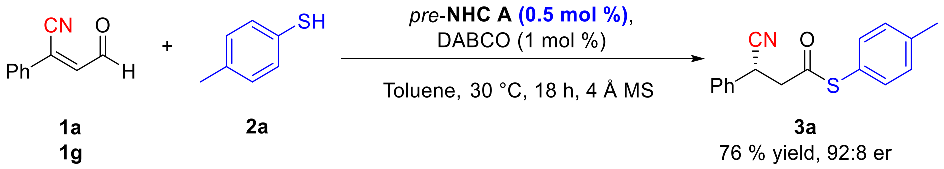

HPLC analysis: (Chiralcel IA; 25 °C, IPA/Hexane = 05/95, 0.5 mL/min, 254 nm), Rt_1 (major) = 36.5 min, Rt_2 (minor) = 42.3 min; 92:8 er.

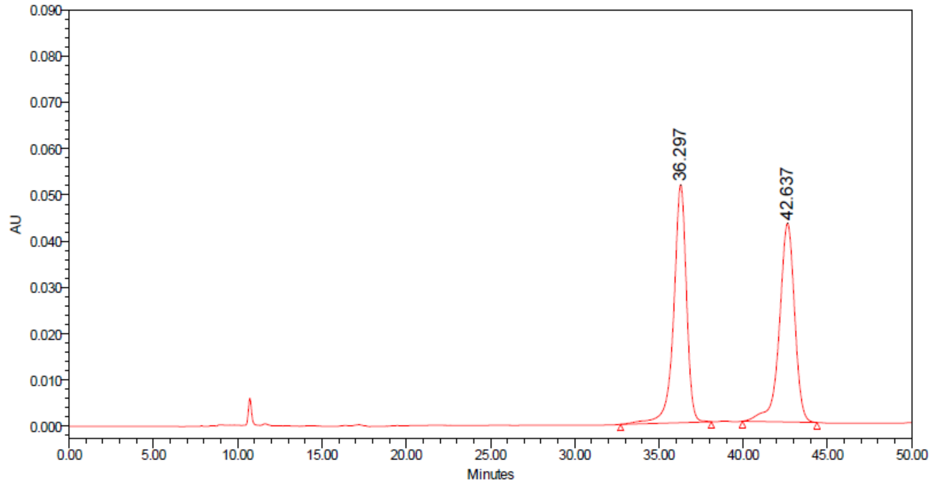

	RT	Area	% Area	Height
1	36.297	2708778	49.99	51437
2	42.637	2710386	50.01	42968

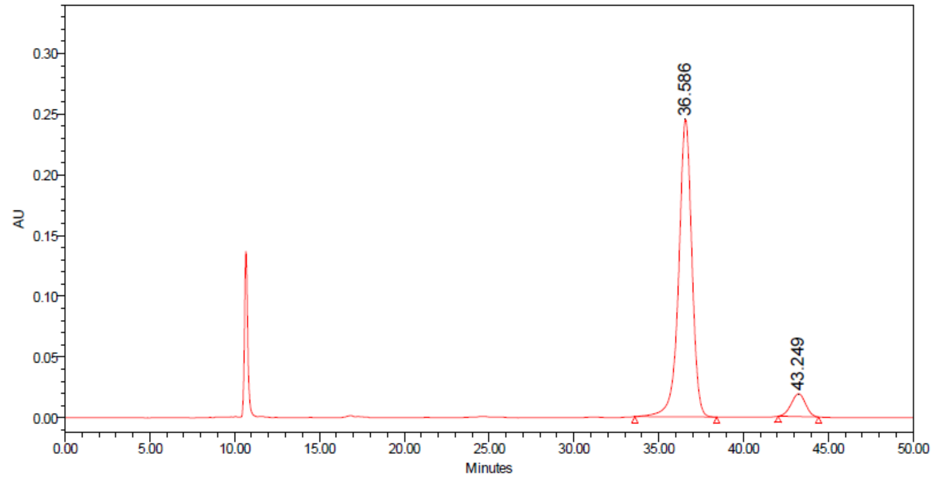

	RT	Area	% Area	Height
1	36.586	13215034	92.25	245081
2	43.249	1110420	7.75	18874

Supplementary Figure 171. HPLC spectra of 3a (scale-up)

Scale-Up Synthesis of 3q.

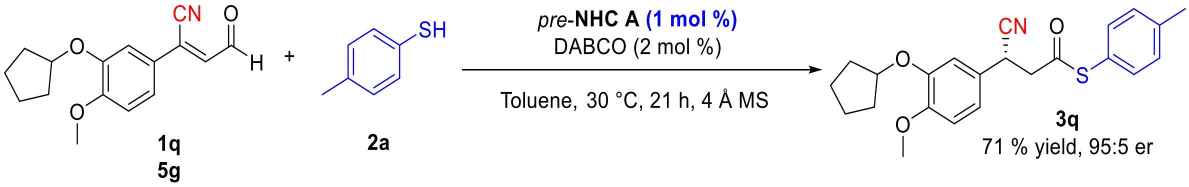

HPLC analysis: (Chiralcel IA; 25 ºC, IPA/Hexane = 05/95, 0.5 mL/min, 254 nm), Rt_1 (major) = 51.1 min, Rt_2 (minor) = 40.8 min; 95:5 er.

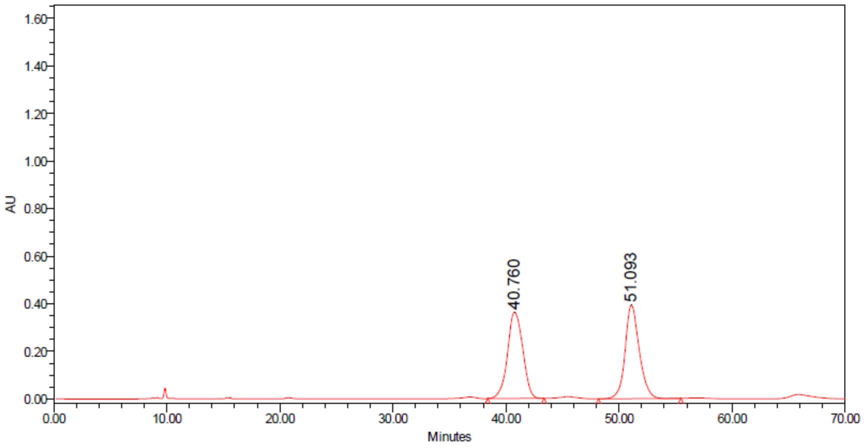

	RT	Area	% Area	Height	% Height
1	40.760	34073529	49.89	363122	47.99
2	51.093	34222207	50.11	393607	52.01

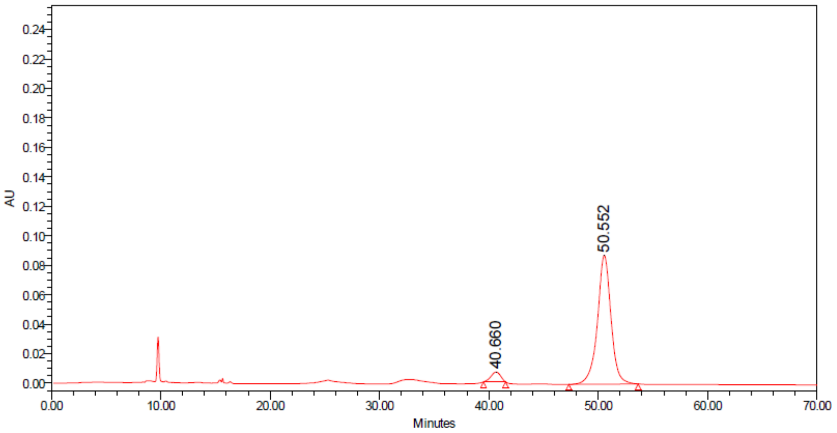

	RT	Area	% Area	Height	% Height
1	40.660	412733	5.20	6759	7.17
2	50.552	7523856	94.80	87452	92.83

Supplementary Figure 172. HPLC spectra of 3q (scale-up)

HRMS spectra

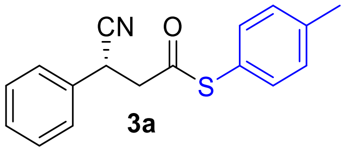

3a

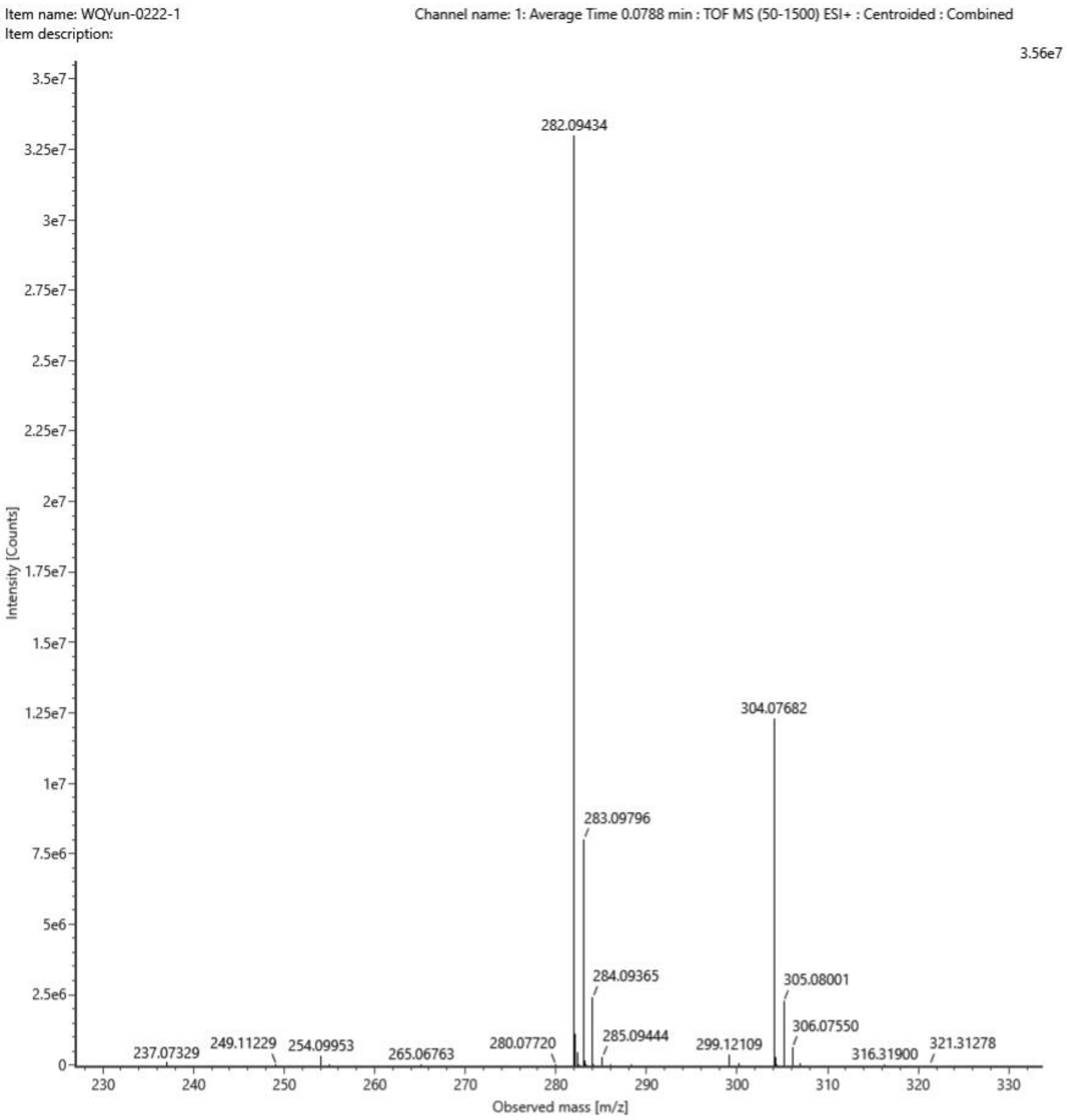

Add:H$^+$

Composition	i-FIT Confidence (%)	Predicted m/z	m/z error (PPM)
C17H15NOS	100.000000	282.094711	-1.320908

Supplementary Figure 173. HRMS spectra of 3a

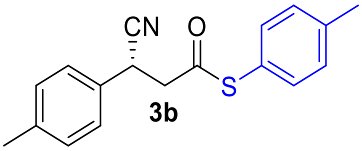

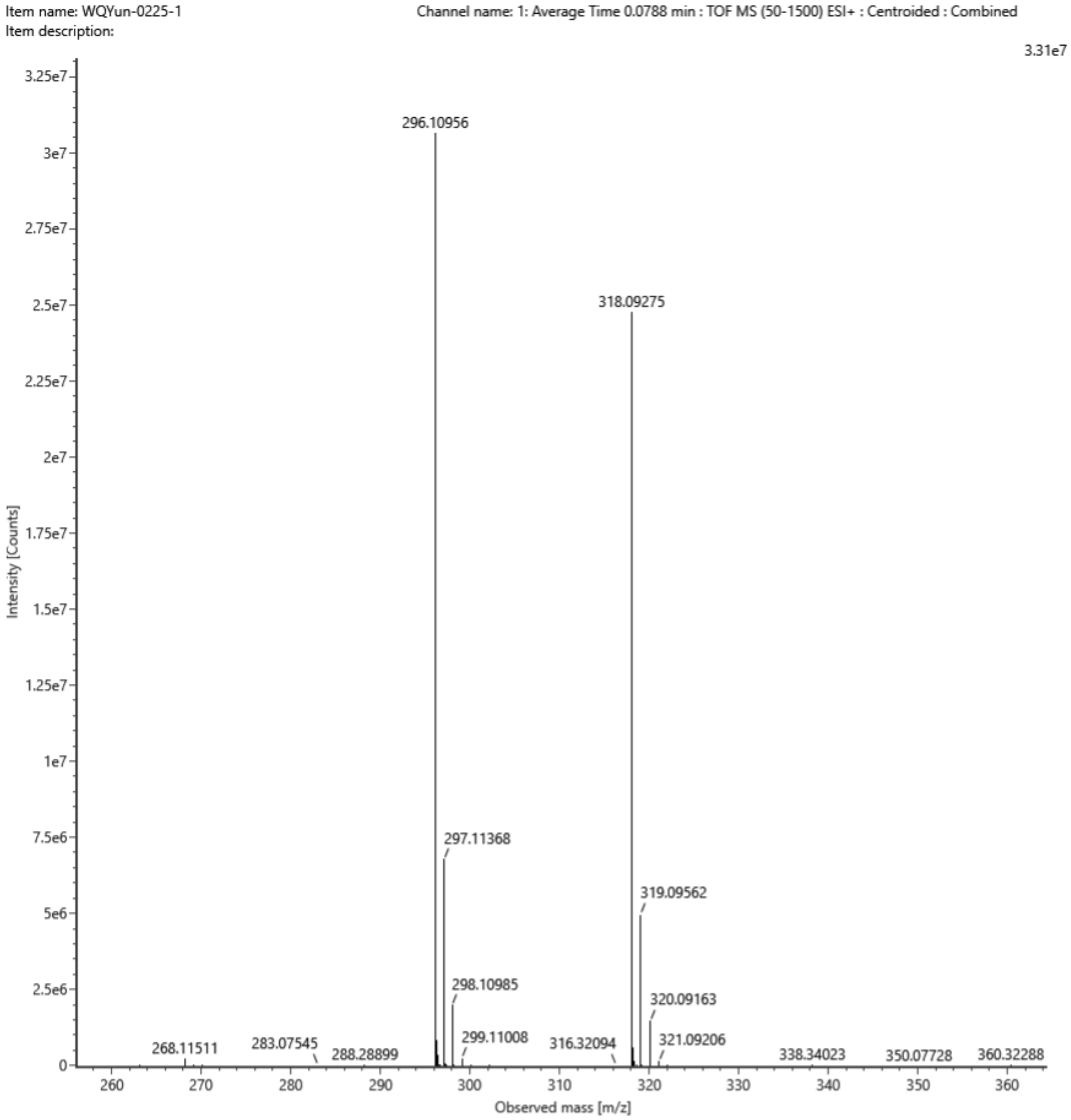

Add:H^+

Composition	i-FIT Confidence (%)	Predicted m/z	m/z error (PPM)
C18H17NOS	100.000000	296.110361	-2.715513

Supplementary Figure 174. HRMS spectra of 3b

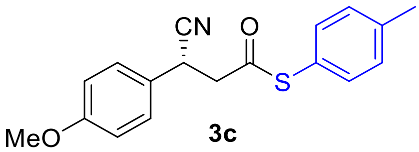

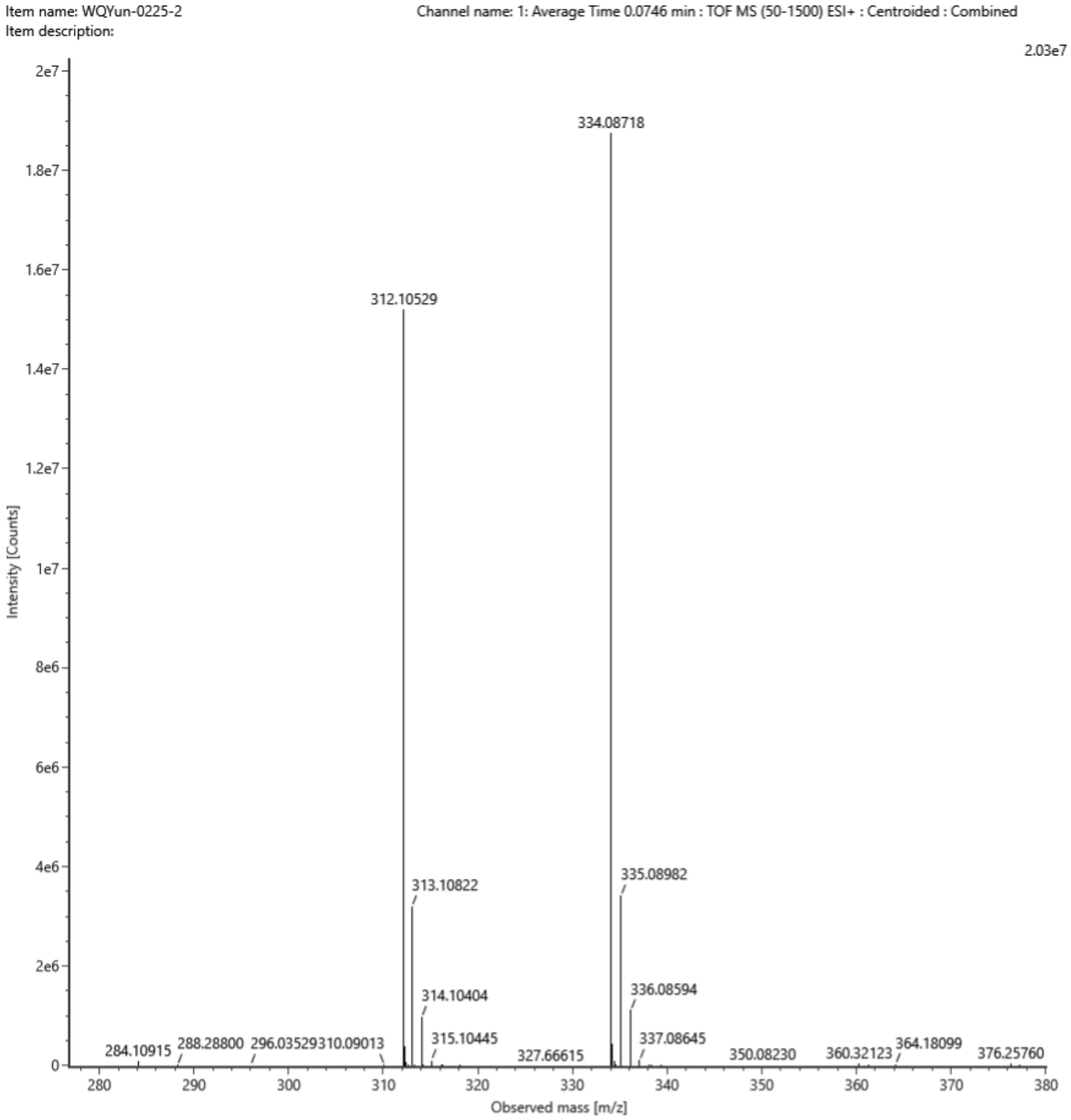

Add:Na^+

Composition	i-FIT Confidence (%)	Predicted m/z	m/z error (PPM)
C18H17NO2S	100.000000	334.087221	-0.121906

Supplementary Figure 175. HRMS spectra of 3c

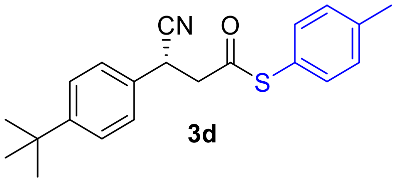

3d

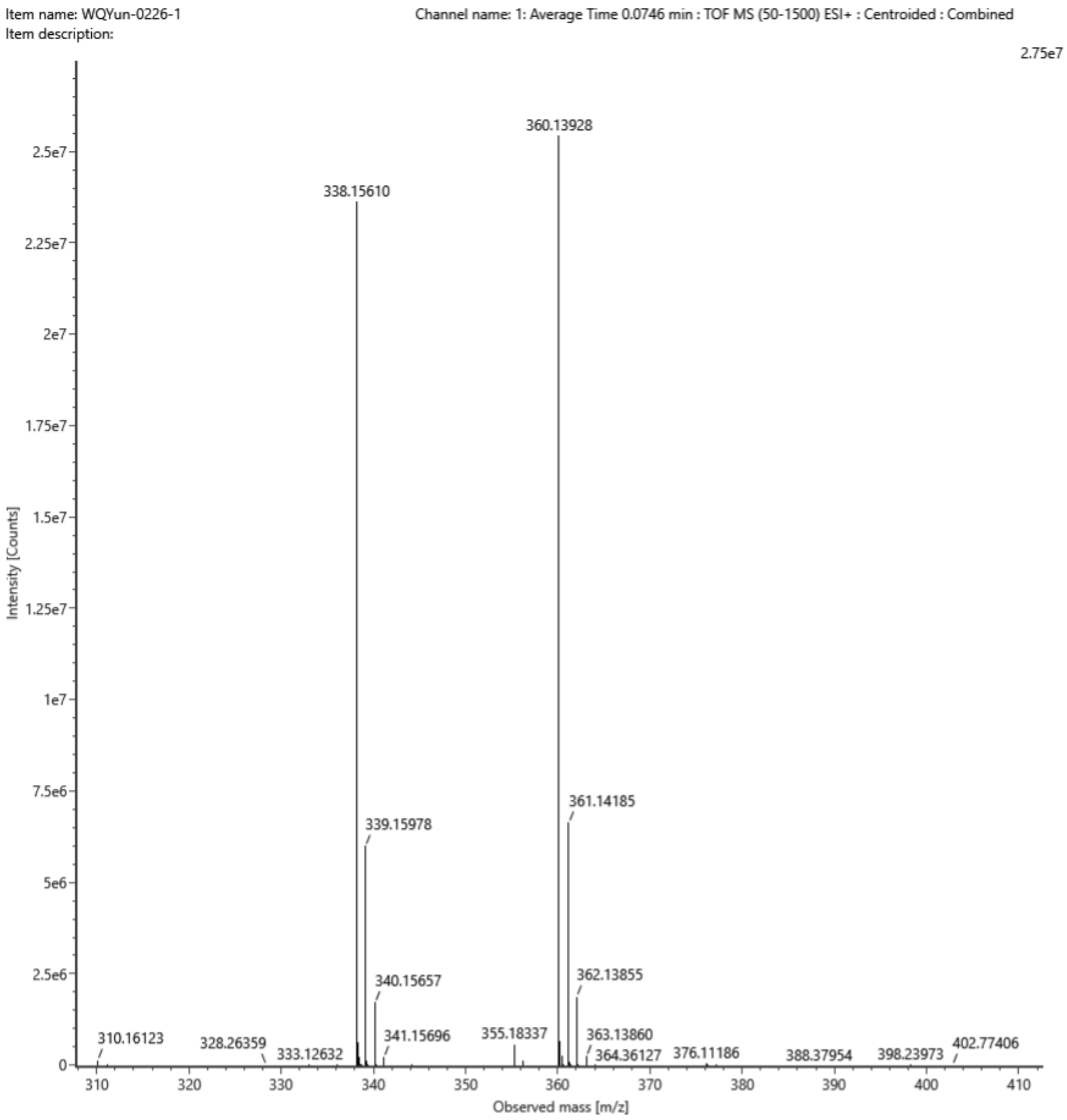

Add:Na^+

Composition	i-FIT Confidence (%)	Predicted m/z	m/z error (PPM)
C21H23NOS	100.000000	360.139256	0.066345

Supplementary Figure 176. HRMS spectra of 3d

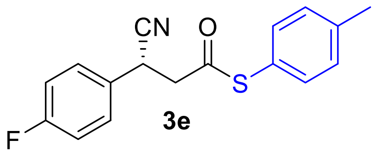

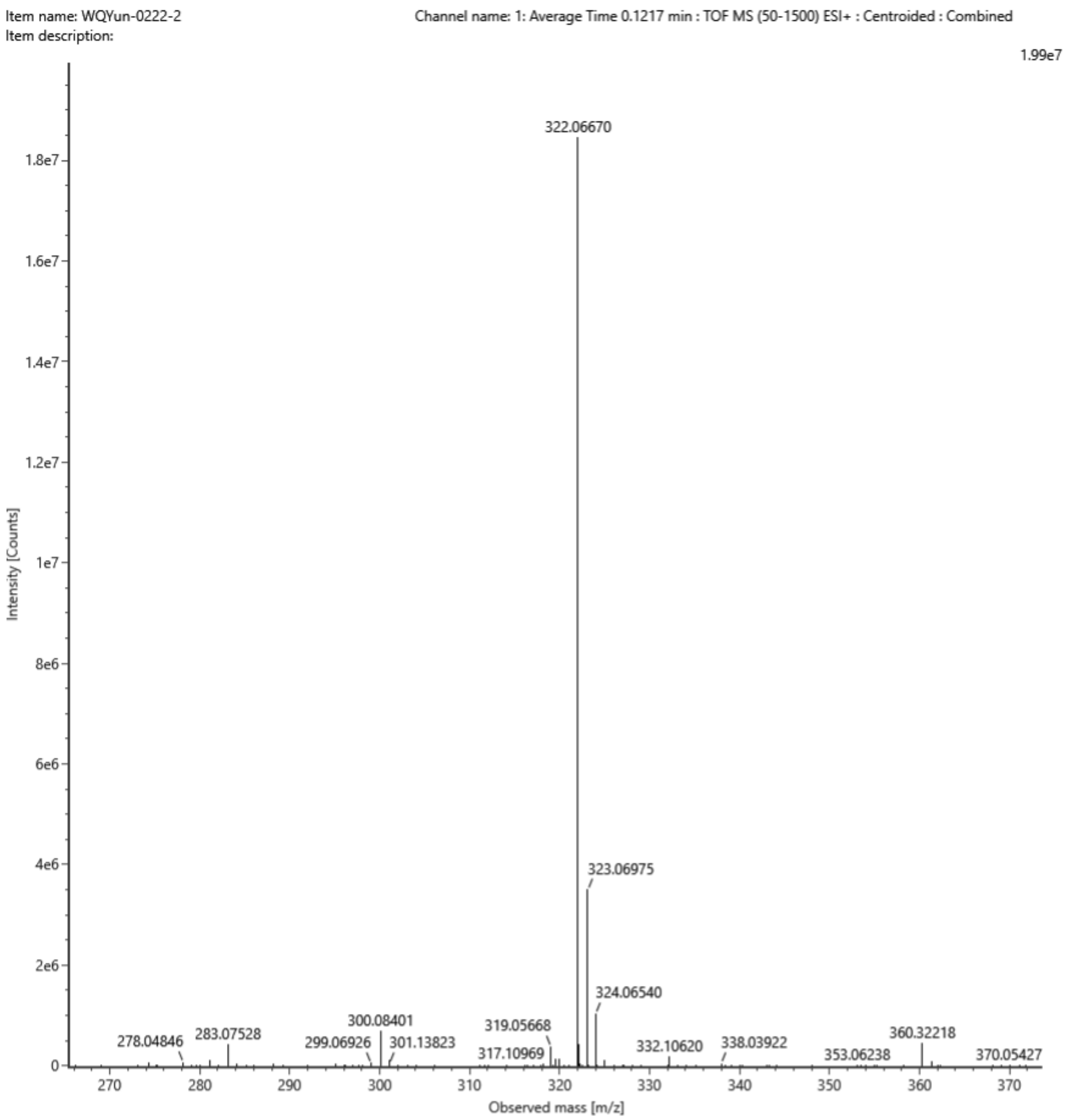

Add:Na^+

Composition	i-FIT Confidence (%)	Predicted m/z	m/z error (PPM)
C17H14FNOS	100.000000	322.067234	-1.663513

Supplementary Figure 177. HRMS spectra of 3e

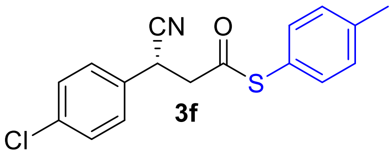

3f

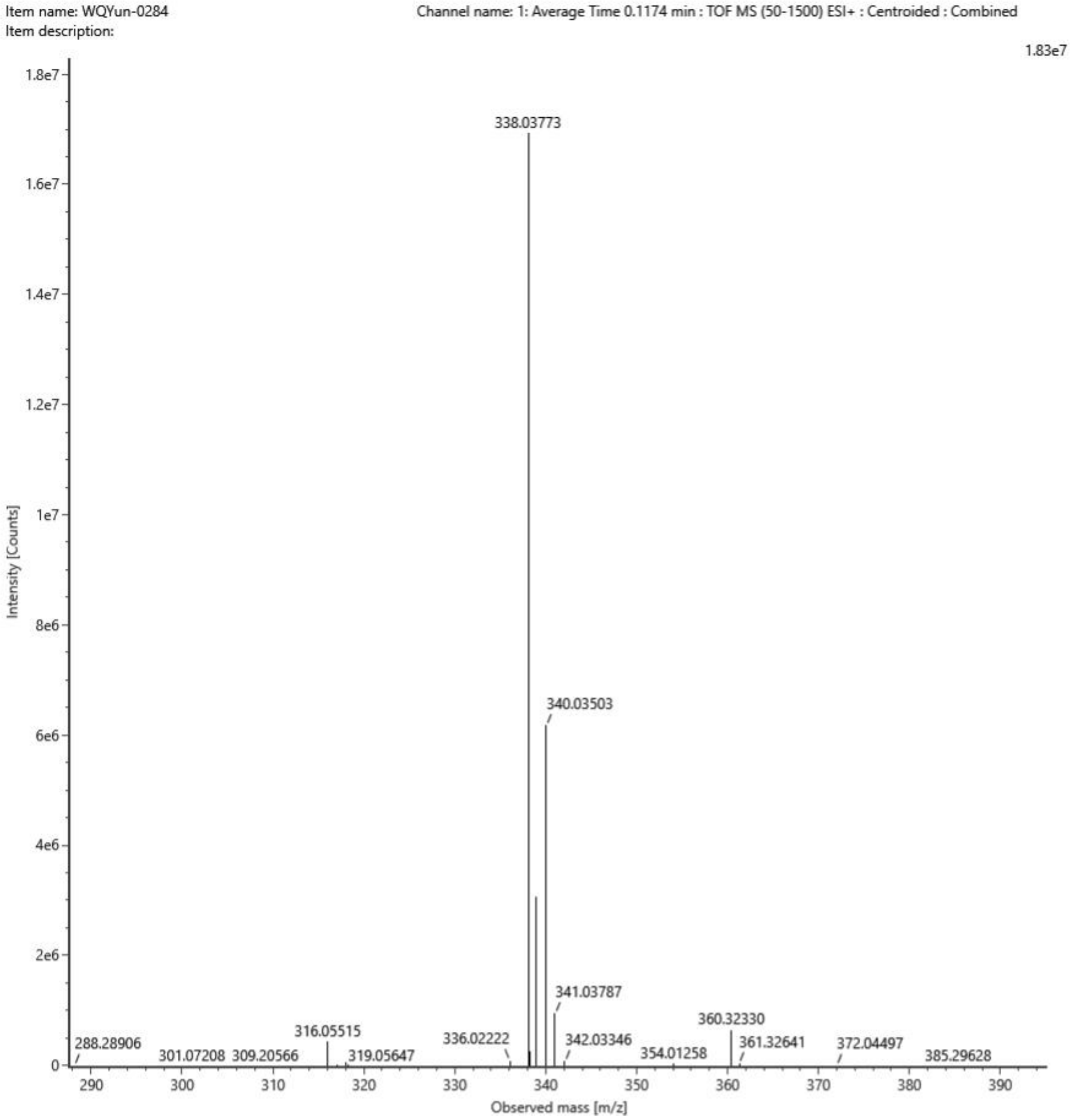

Add:Na^+

Composition	i-FIT Confidence (%)	Predicted m/z	m/z error (PPM)
C17H14ClNOS	100.000000	338.037684	0.137684

Supplementary Figure 178. HRMS spectra of 3f

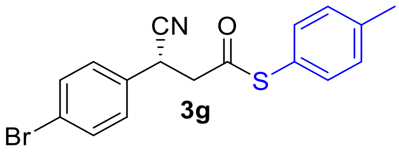

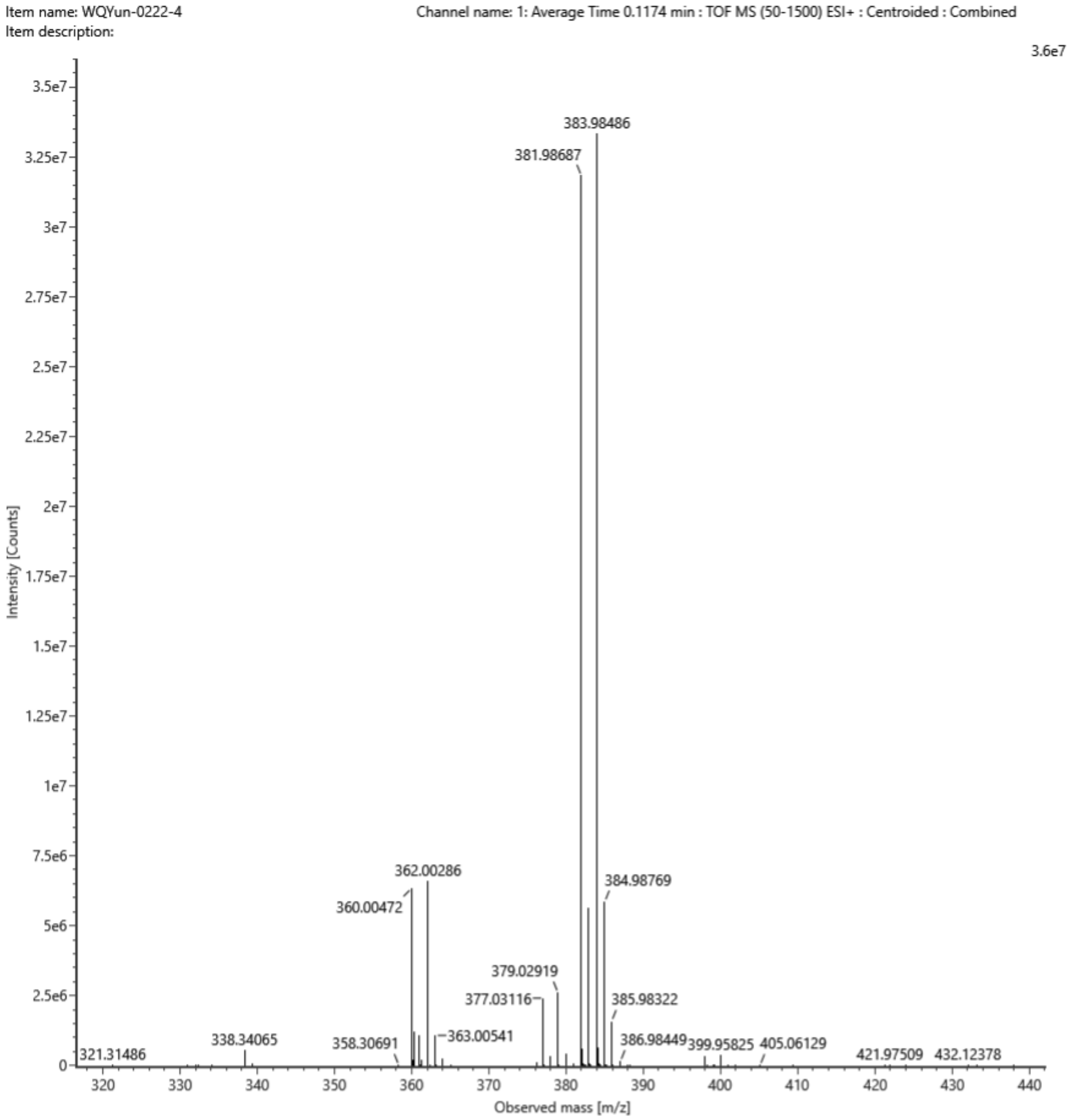

Add:Na^+

Composition	i-FIT Confidence (%)	Predicted m/z	m/z error (PPM)
C17H14BrNOS	100.000000	381.987169	-0.832280

Supplementary Figure 179. HRMS spectra of 3g

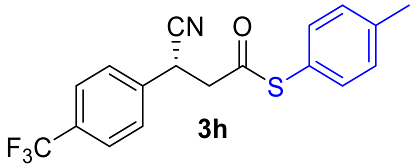

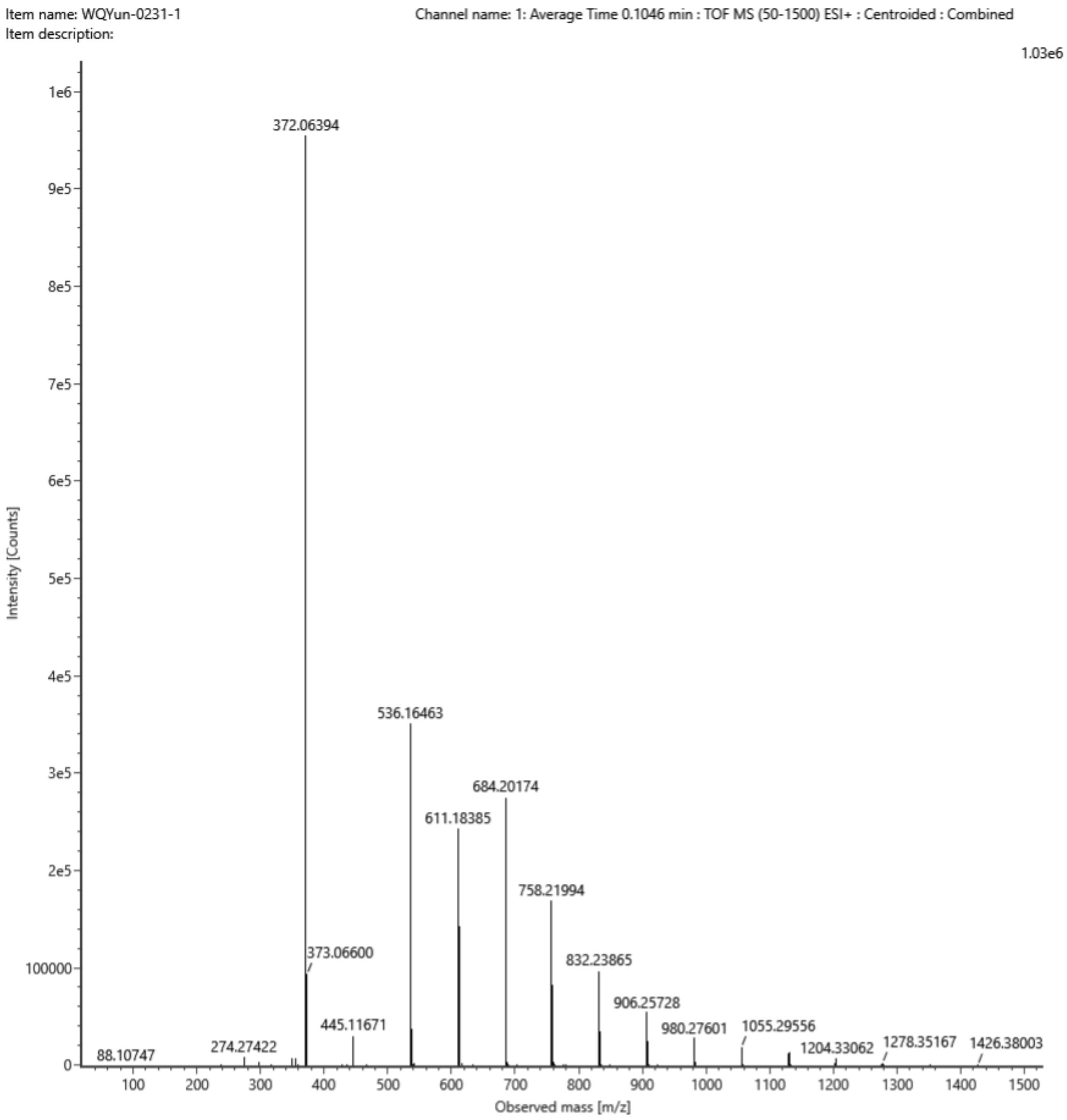

Add:Na+

Composition	i-FIT Confidence (%)	Predicted m/z	m/z error (PPM)
C18H14F3NOS	100.000000	372.064040	-0.270811

Supplementary Figure 180. HRMS spectra of 3h

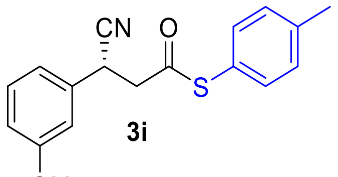

3i

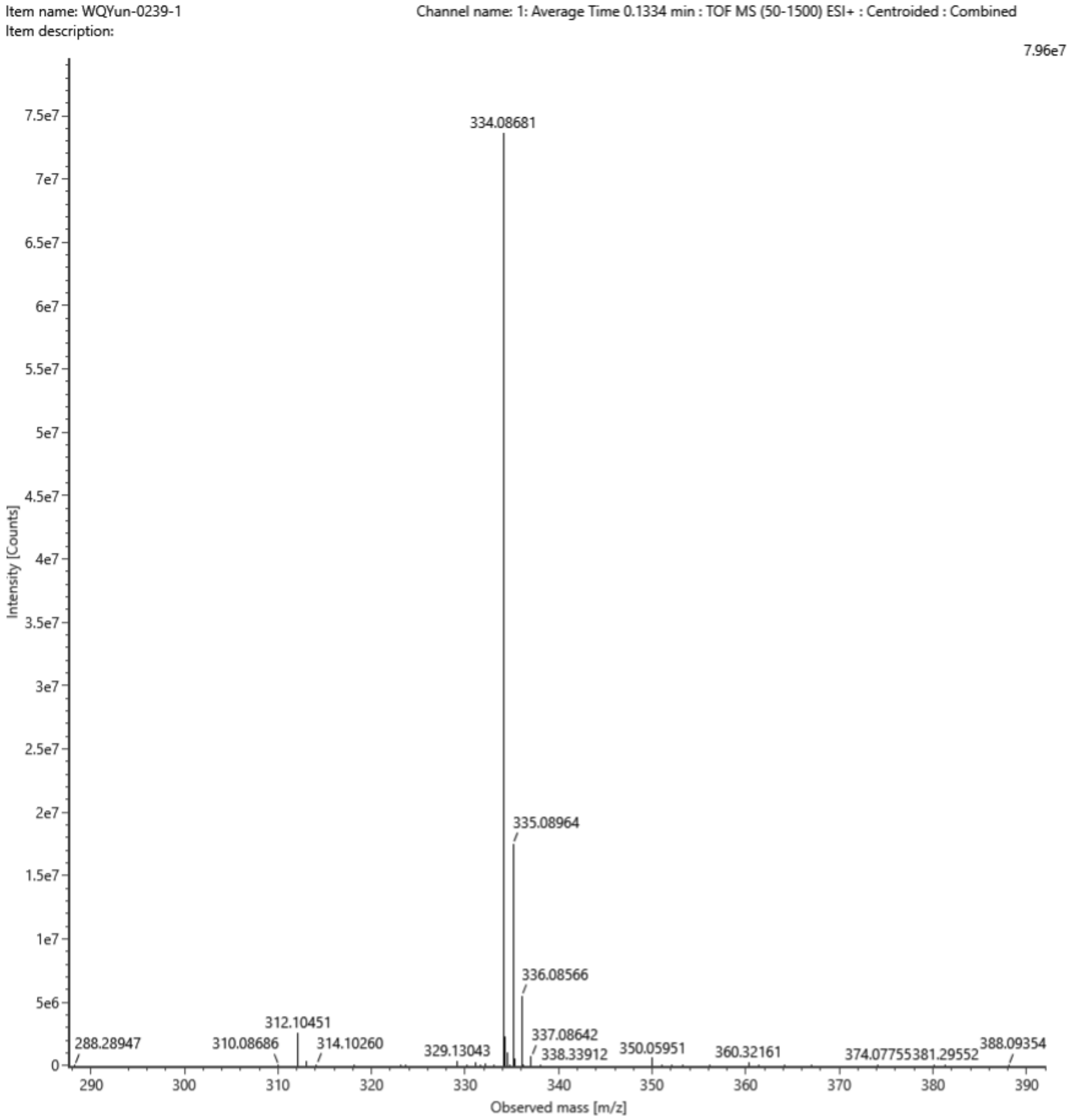

Add:Na^+

Composition	i-FIT Confidence (%)	Predicted m/z	m/z error (PPM)
C18H17NO2S	99.999999	334.087221	-1.232752

Supplementary Figure 181. HRMS spectra of 3i

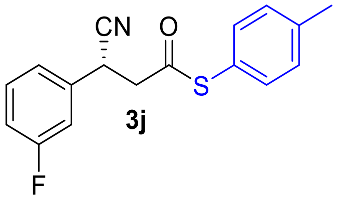

3j

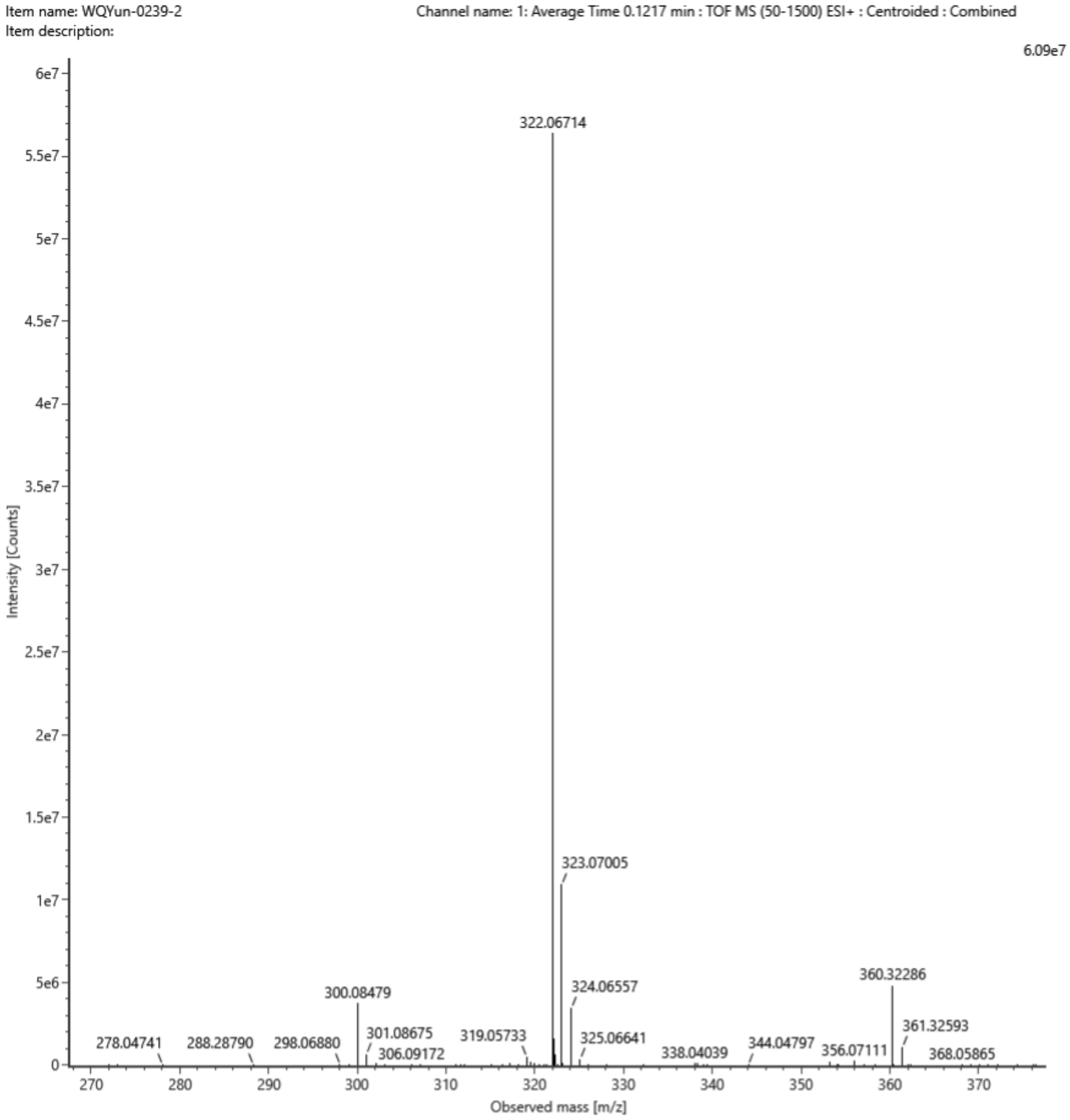

Add:Na^+

Composition	i-FIT Confidence (%)	Predicted m/z	m/z error (PPM)
C17H14FNOS	100.000000	322.067234	-0.293049

Supplementary Figure 182. HRMS spectra of 3j

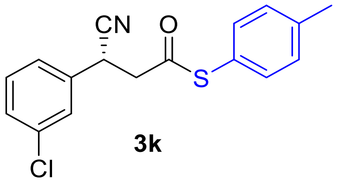

3k

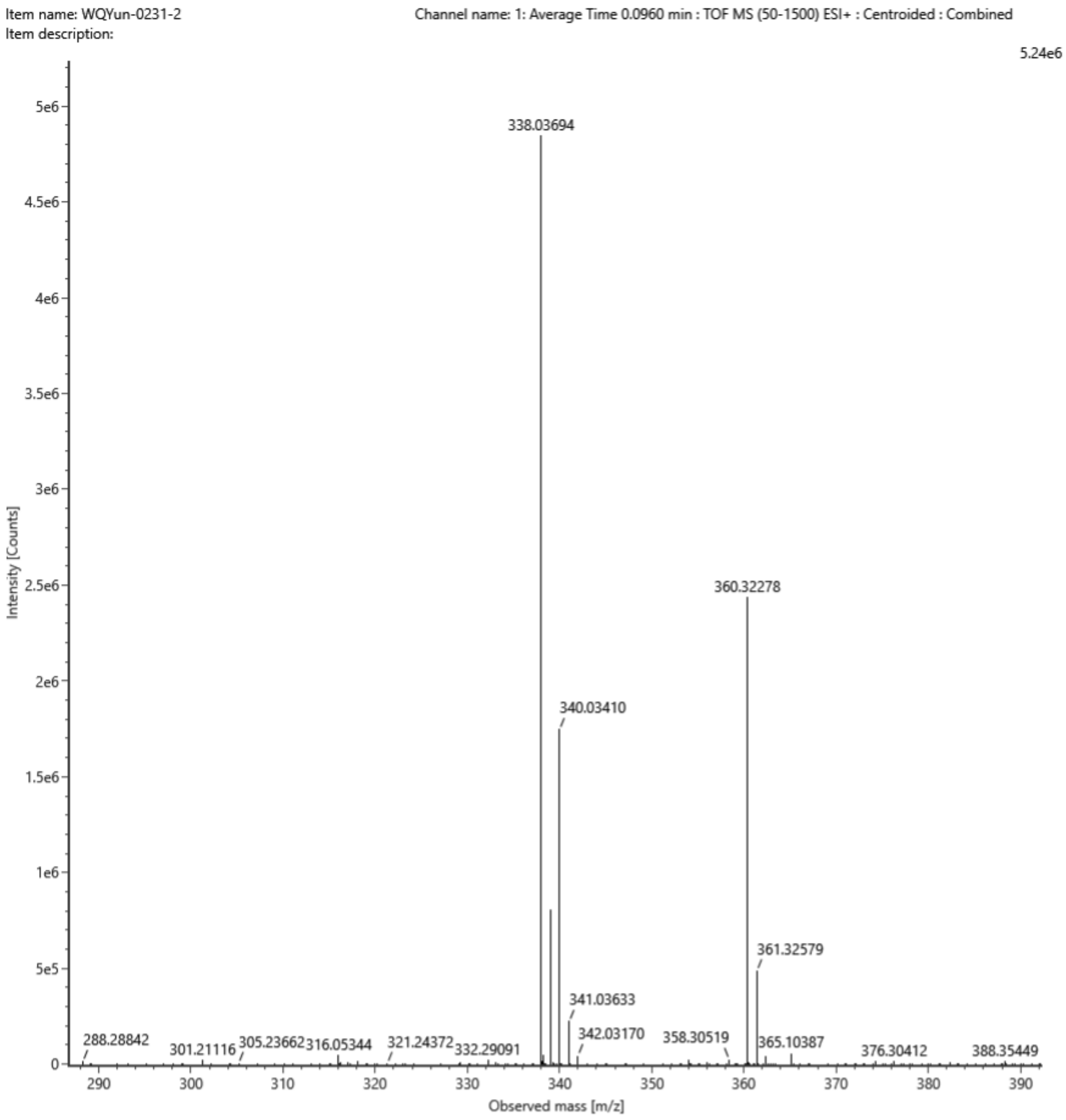

Add:Na^{+}

Composition	i-FIT Confidence (%)	Predicted m/z	m/z error (PPM)
C17H14ClNOS	100.000000	338.037684	-2.206323

Supplementary Figure 183. HRMS spectra of 3k

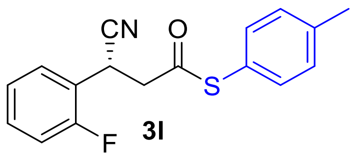

3l

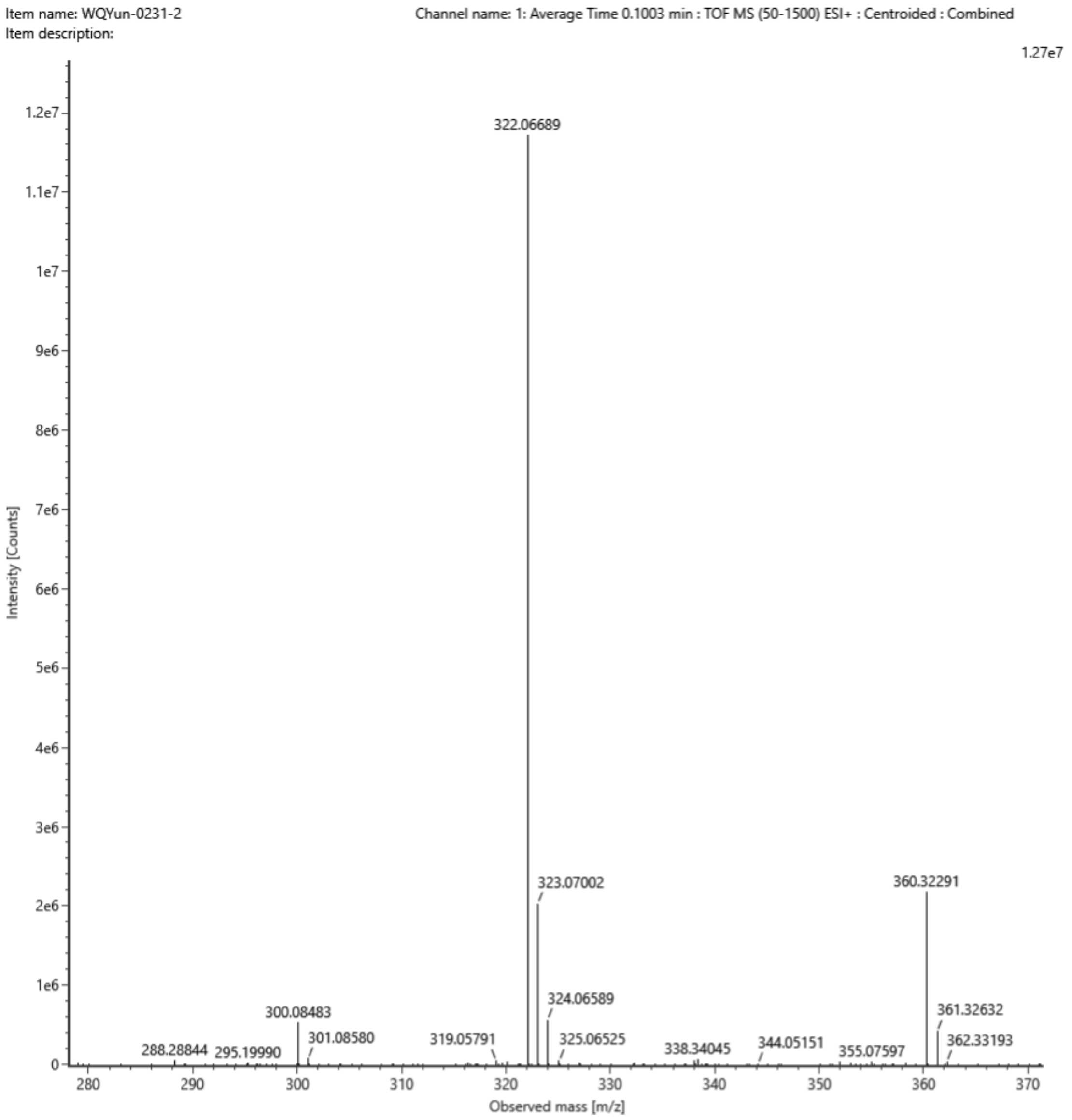

Add:Na^+

Composition	i-FIT Confidence (%)	Predicted m/z	m/z error (PPM)
C17H14FNOS	99.951274	322.067234	-1.071721

Supplementary Figure 184. HRMS spectra of 3l

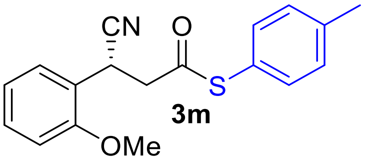

3m

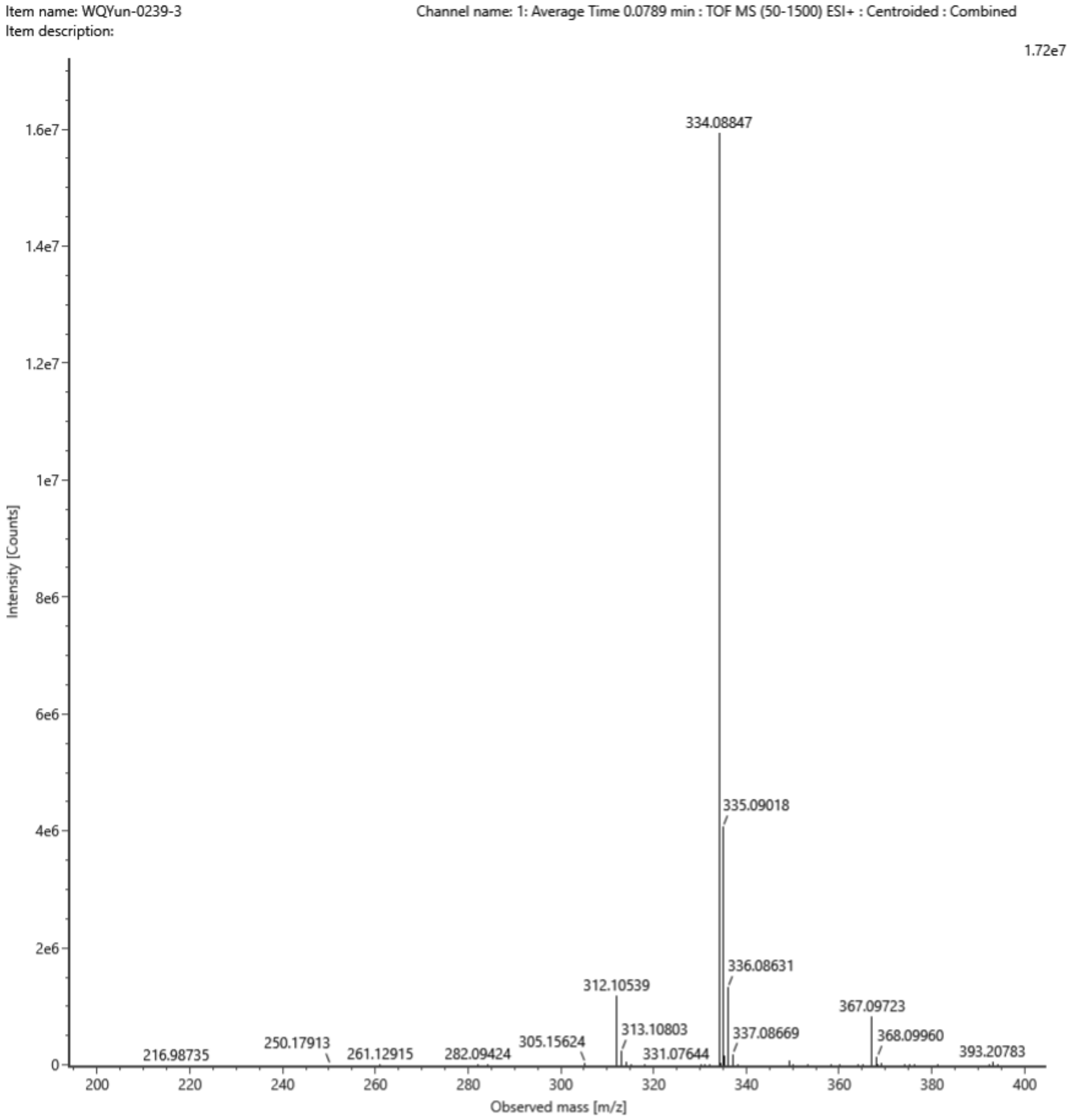

Add:Na^{+}

Composition	i-FIT Confidence (%)	Predicted m/z	m/z RMS (PPM)
C18H17NO2S	95.181724	334.087221	3.316037

Supplementary Figure 185. HRMS spectra of 3m

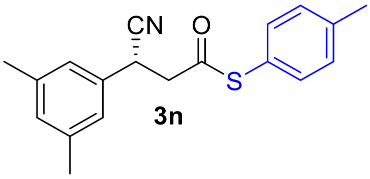

3n

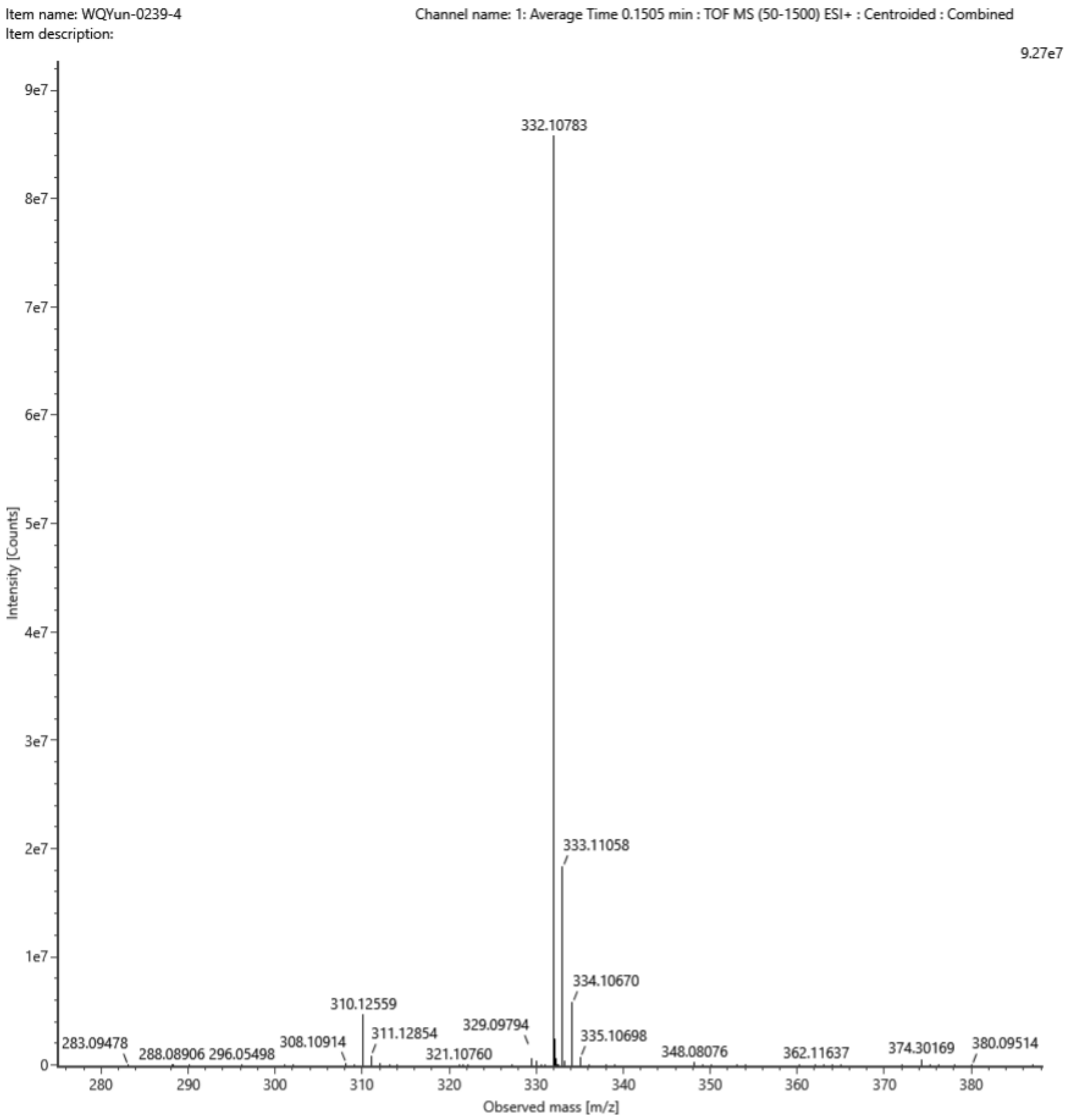

Add:Na^+

Composition	i-FIT Confidence (%)	Predicted m/z	m/z error (PPM)
C19H19NOS	100.000000	332.107956	-0.380688

Supplementary Figure 186. HRMS spectra of 3n

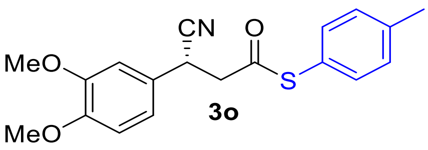

3o

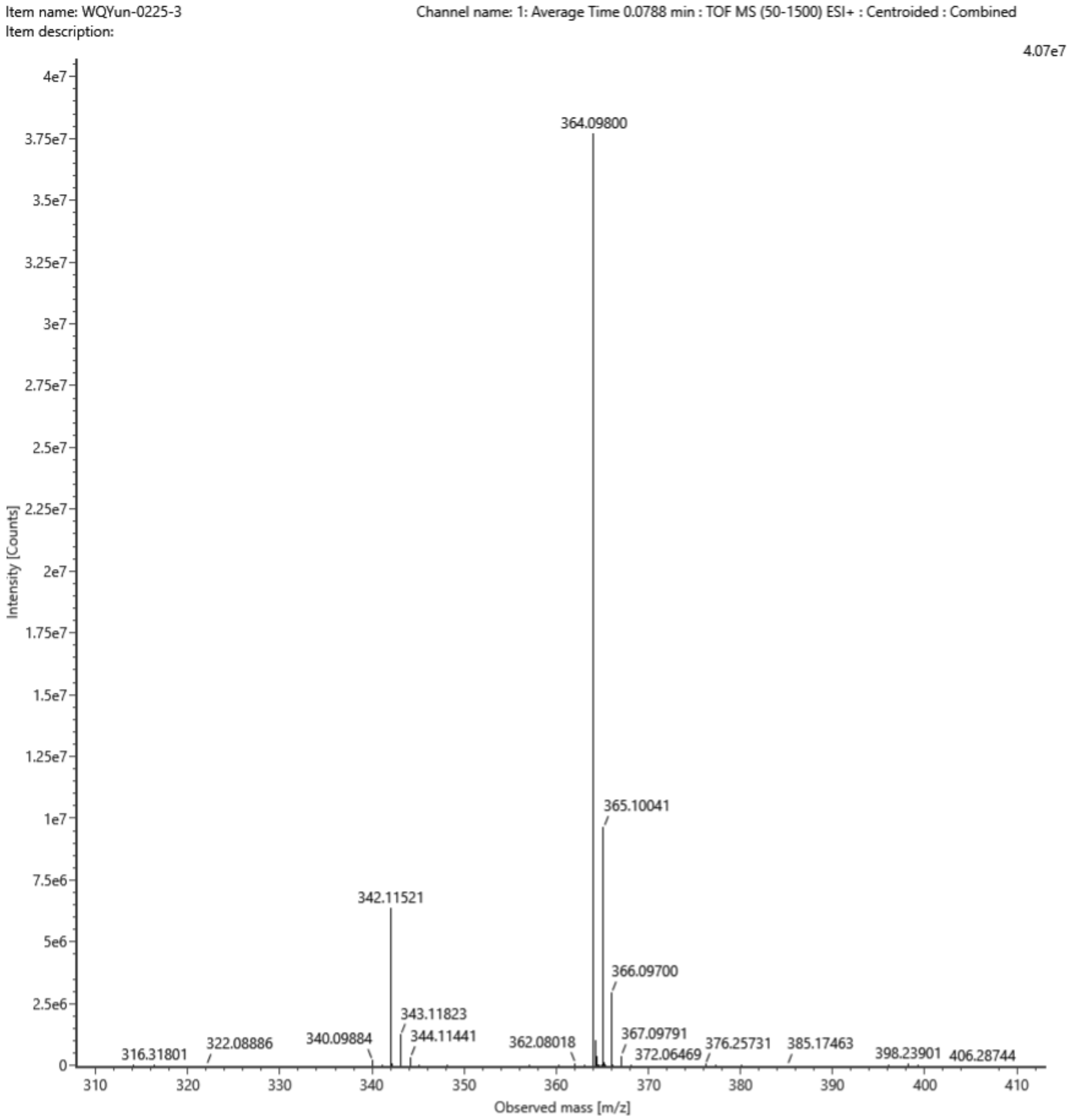

Add:Na^{+}

Composition	i-FIT Confidence (%)	Predicted m/z	m/z error (PPM)
C19H19NO3S	100.000000	364.097785	0.591338

Supplementary Figure 187. HRMS spectra of 3o

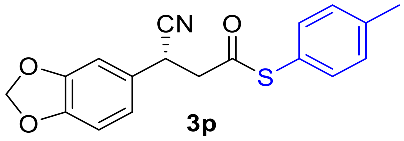

3p

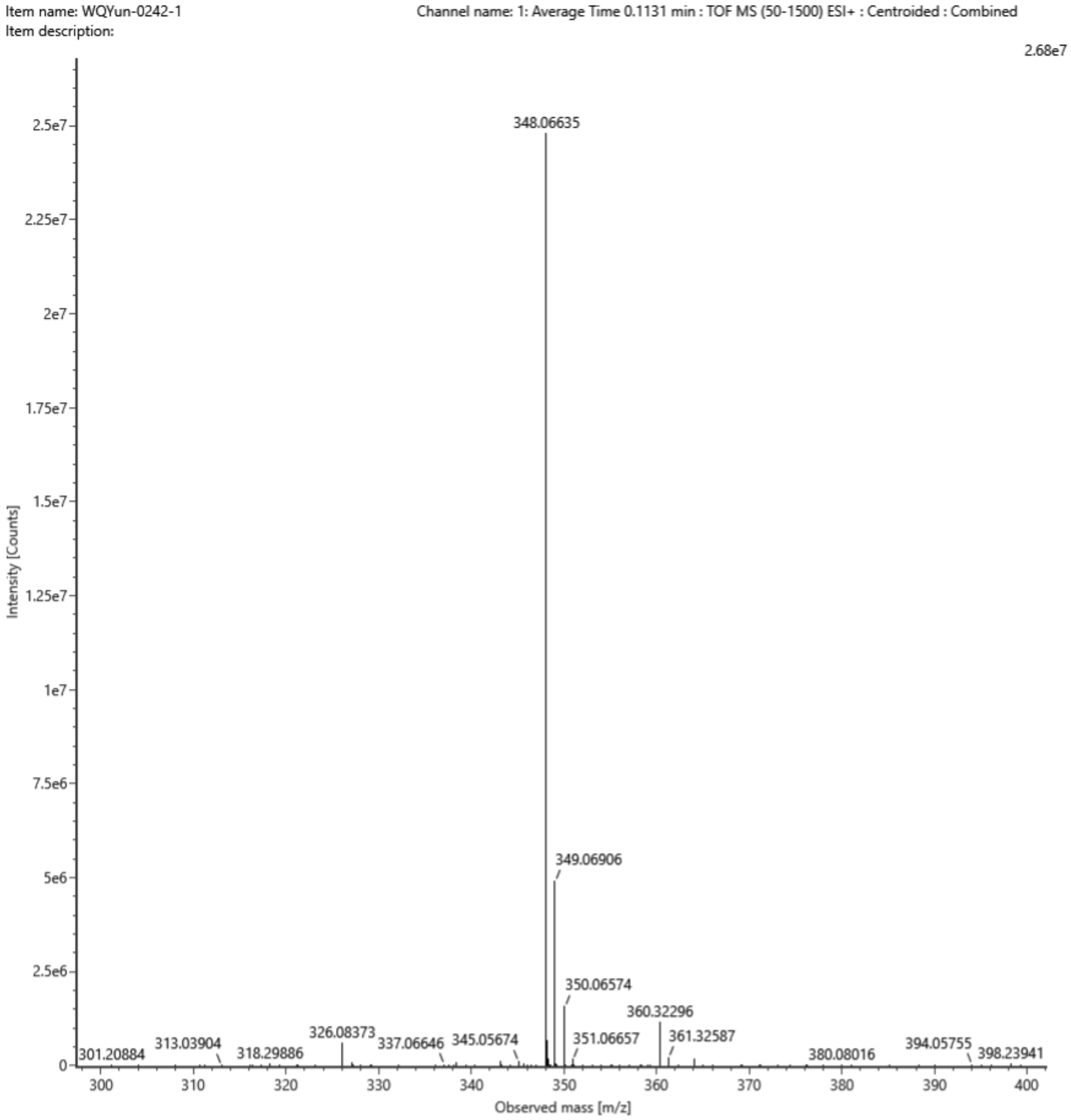

Add:Na$^+$

Composition	i-FIT Confidence (%)	Predicted m/z	m/z error (PPM)
C18H15NO3S	99.998276	348.066485	-0.415787

Supplementary Figure 188. HRMS spectra of 3p

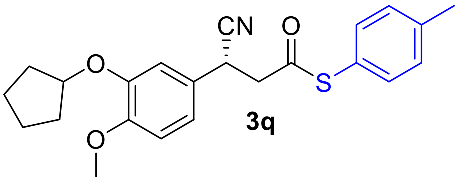

3q

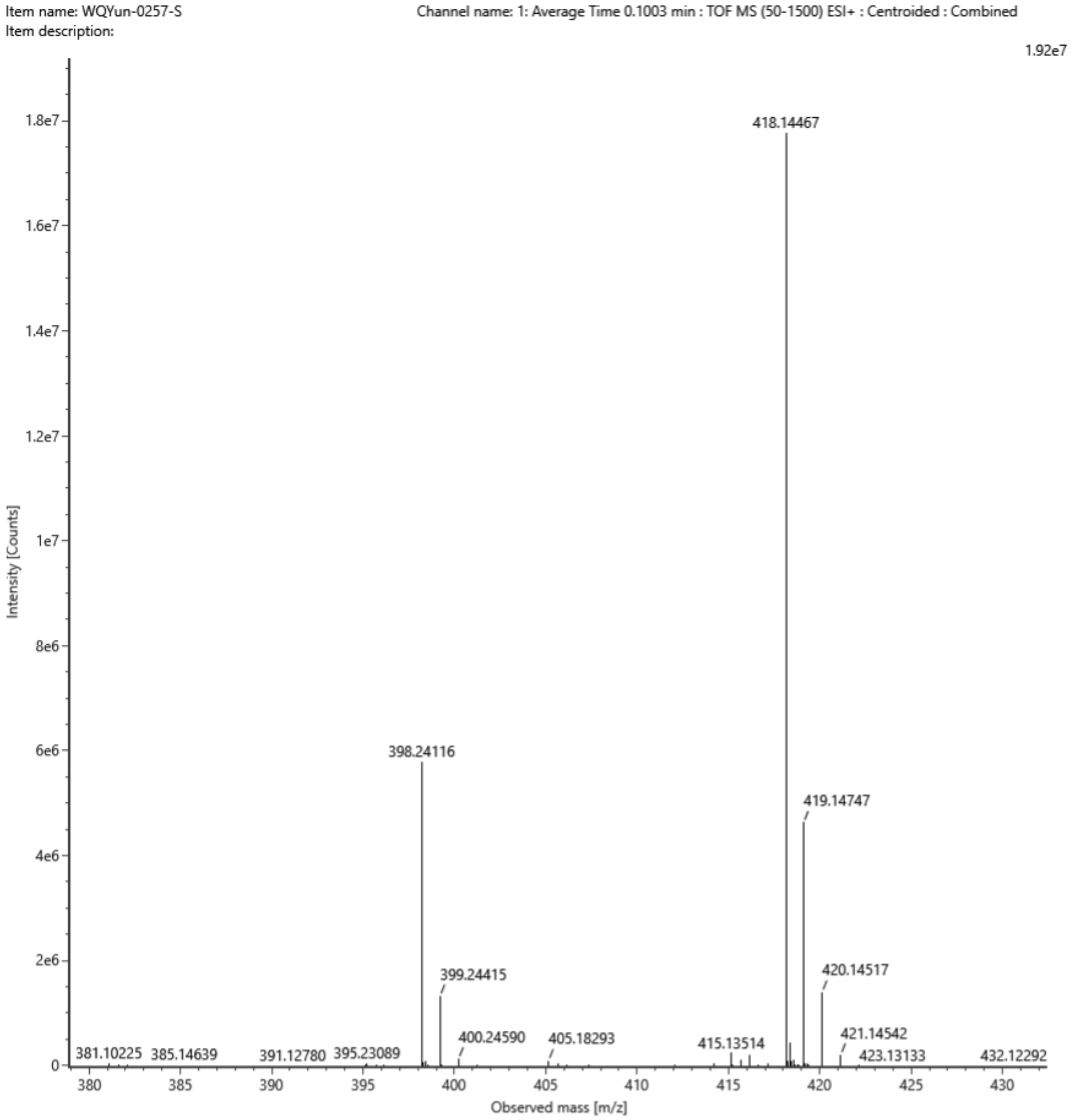

Add: Na^+

Composition	i-FIT Confidence (%)	Predicted m/z	m/z error (PPM)
C23H25NO3S	99.999991	418.144735	-0.156979

Supplementary Figure 189. HRMS spectra of 3q

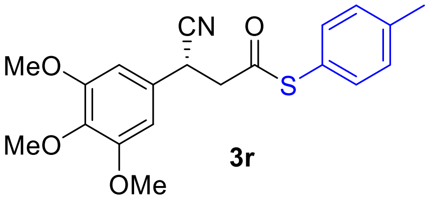

3r

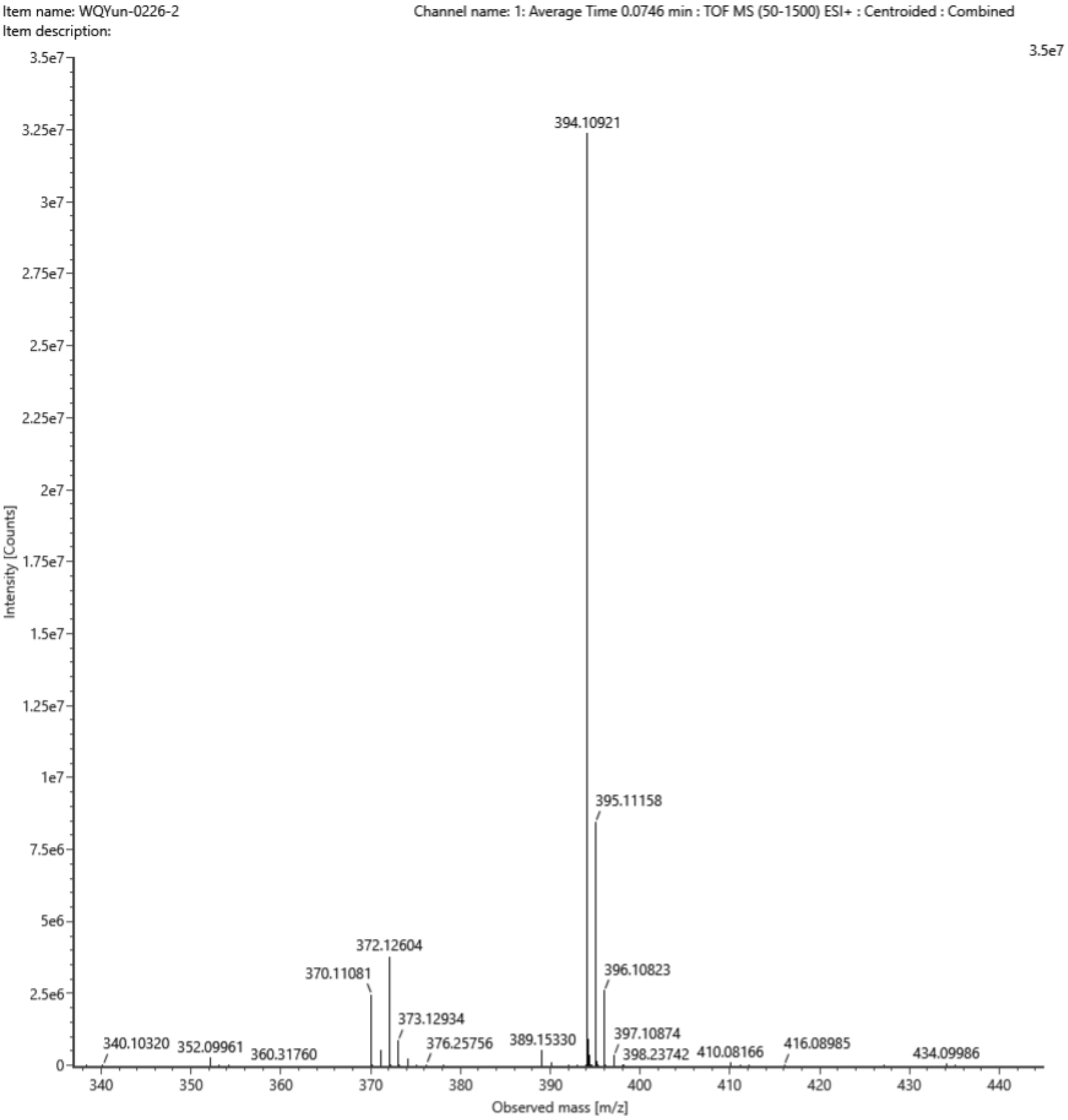

Add:Na^+

Composition	i-FIT Confidence (%)	Predicted m/z	m/z error (PPM)
C20H20NO4S	100.000000	394.108350	2.187787

Supplementary Figure 190. HRMS spectra of 3r

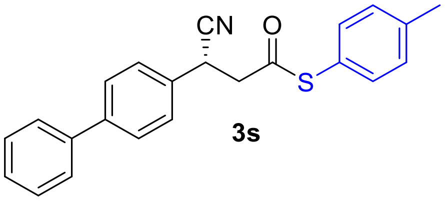

3s

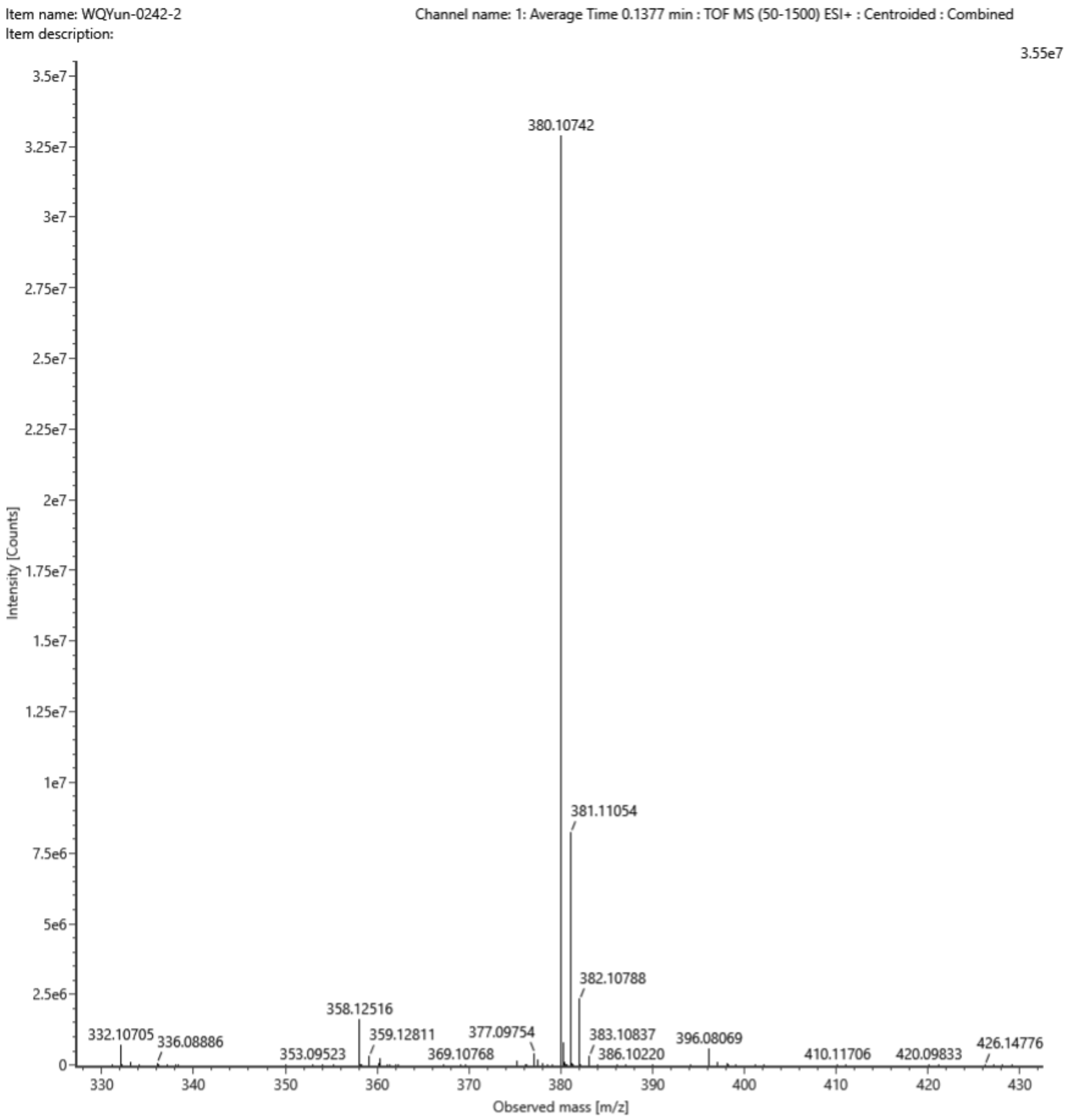

Add:Na^+

Composition	i-FIT Confidence (%)	Predicted m/z	m/z error (PPM)
C23H19NOS	100.000000	380.107956	-1.413995

Supplementary Figure 191. HRMS spectra of 3s

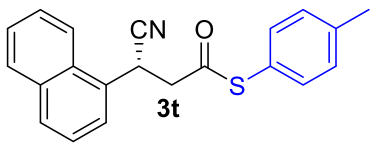

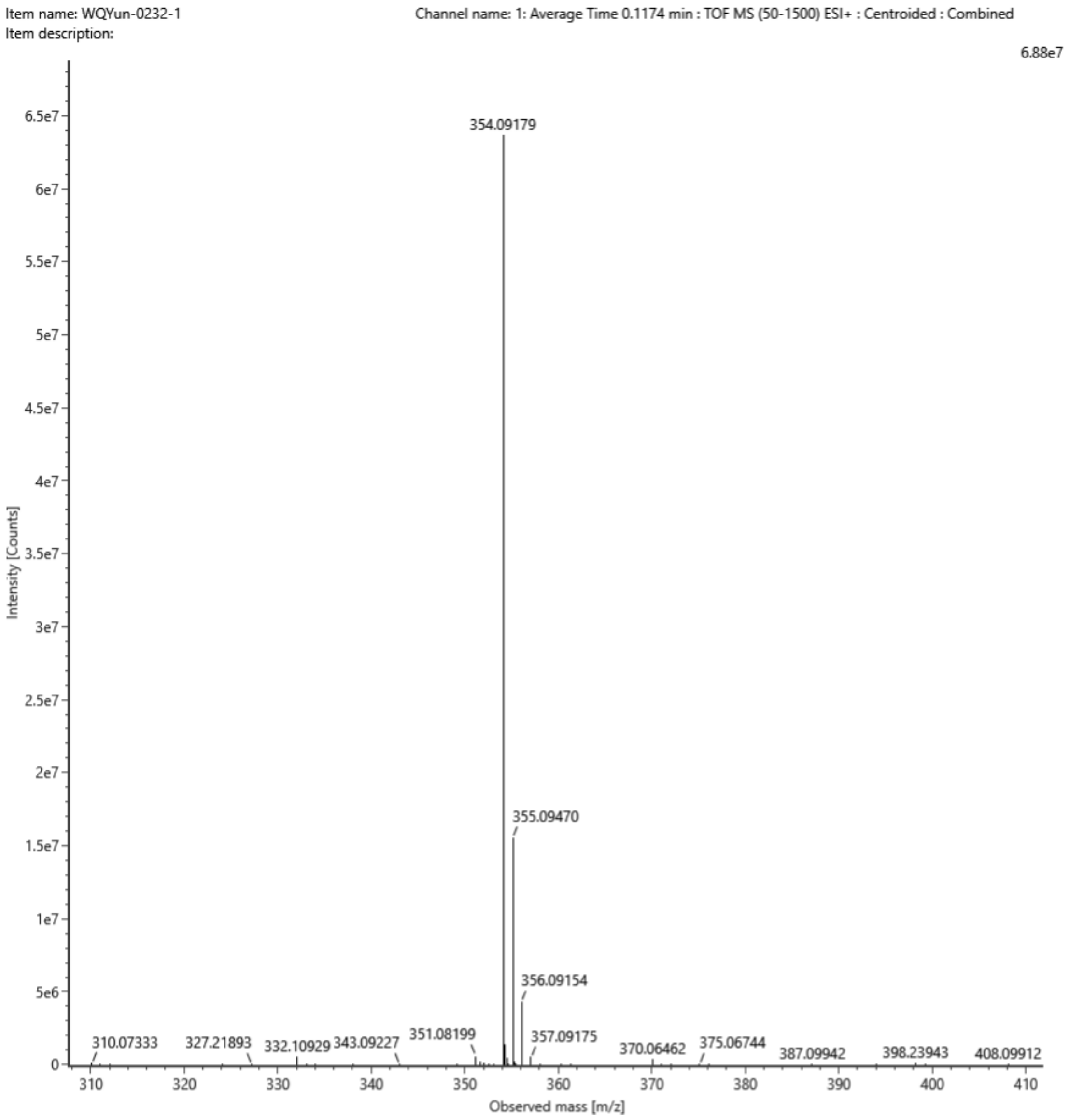

Add:Na^+

Composition	i-FIT Confidence (%)	Predicted m/z	m/z error (PPM)
C21H17NOS	99.999999	354.092306	-1.558375

Supplementary Figure 192. HRMS spectra of 3t

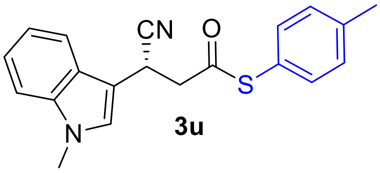

3u

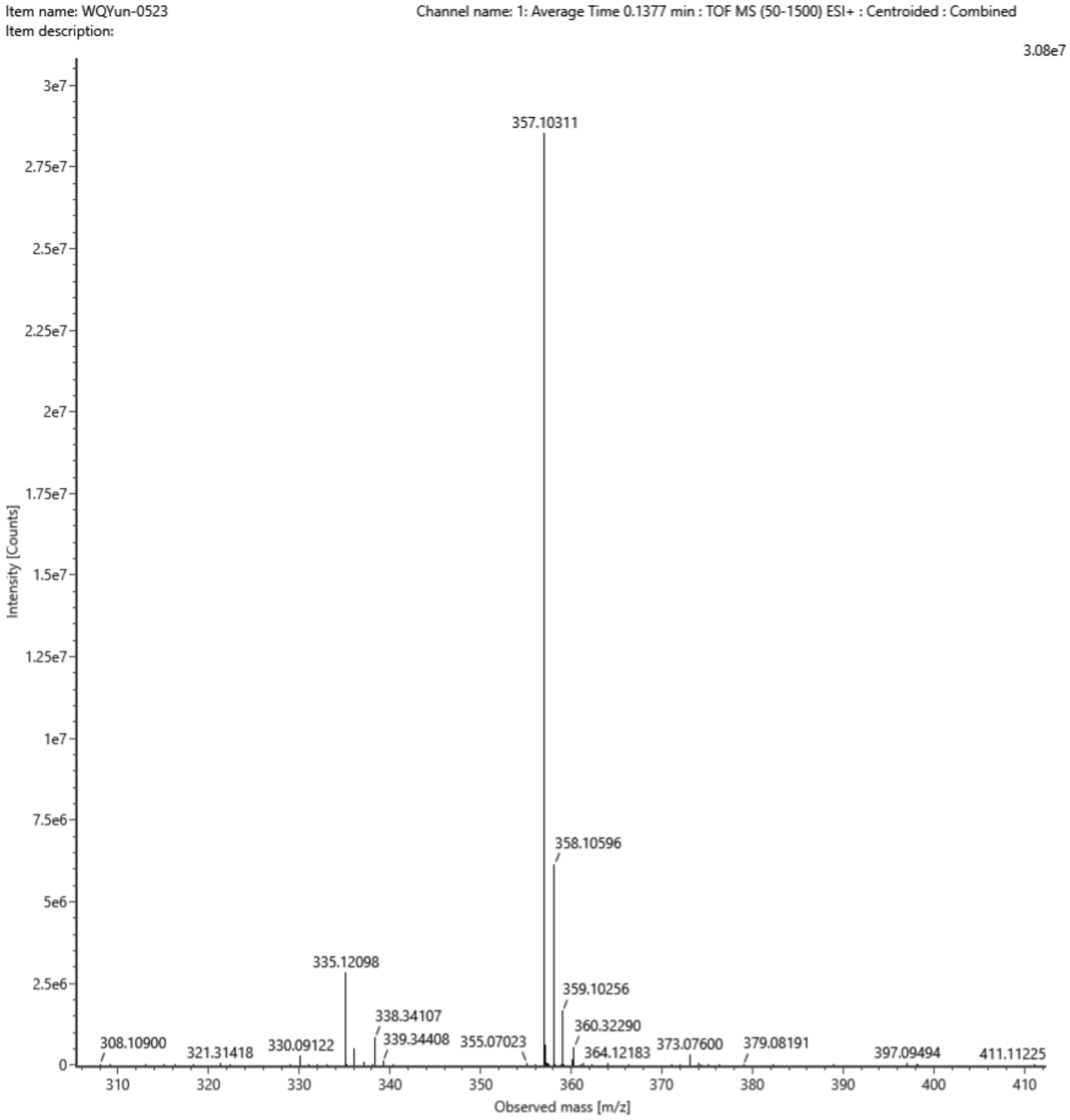

Add:Na^+

Composition	i-FIT Confidence (%)	Predicted m/z	m/z error (PPM)
C20H18N2OS	100.000000	357.103205	-0.266842

Supplementary Figure 193. HRMS spectra of 3u

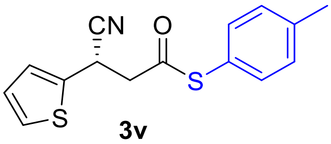

3v

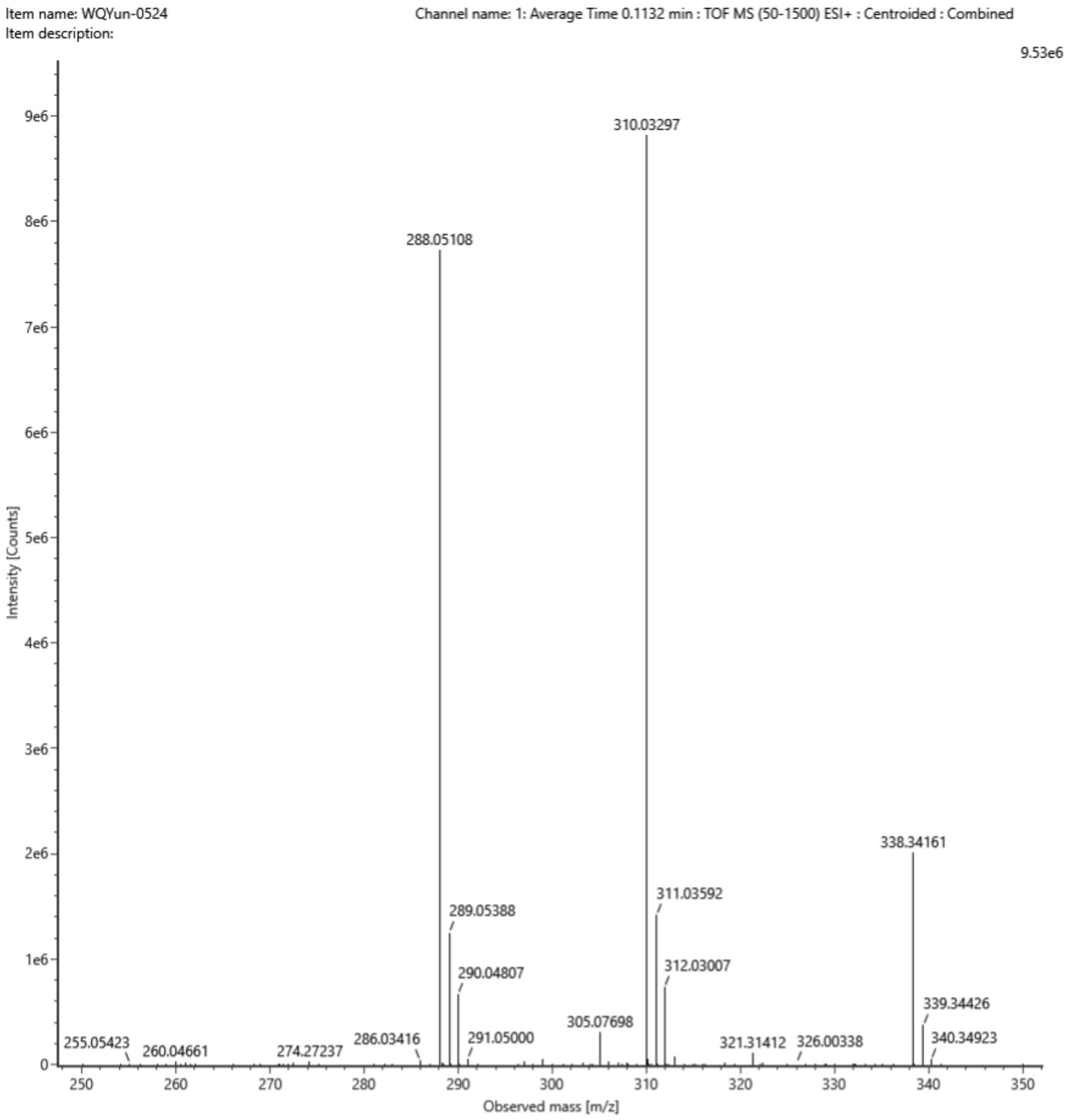

Add:Na^+

Composition	i-FIT Confidence (%)	Predicted m/z	m/z error (PPM)
C15H13NOS2	100.000000	310.033077	-0.344905

Supplementary Figure 194. HRMS spectra of 3v

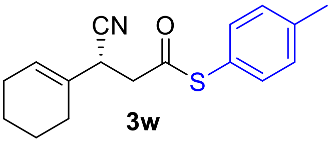

3w

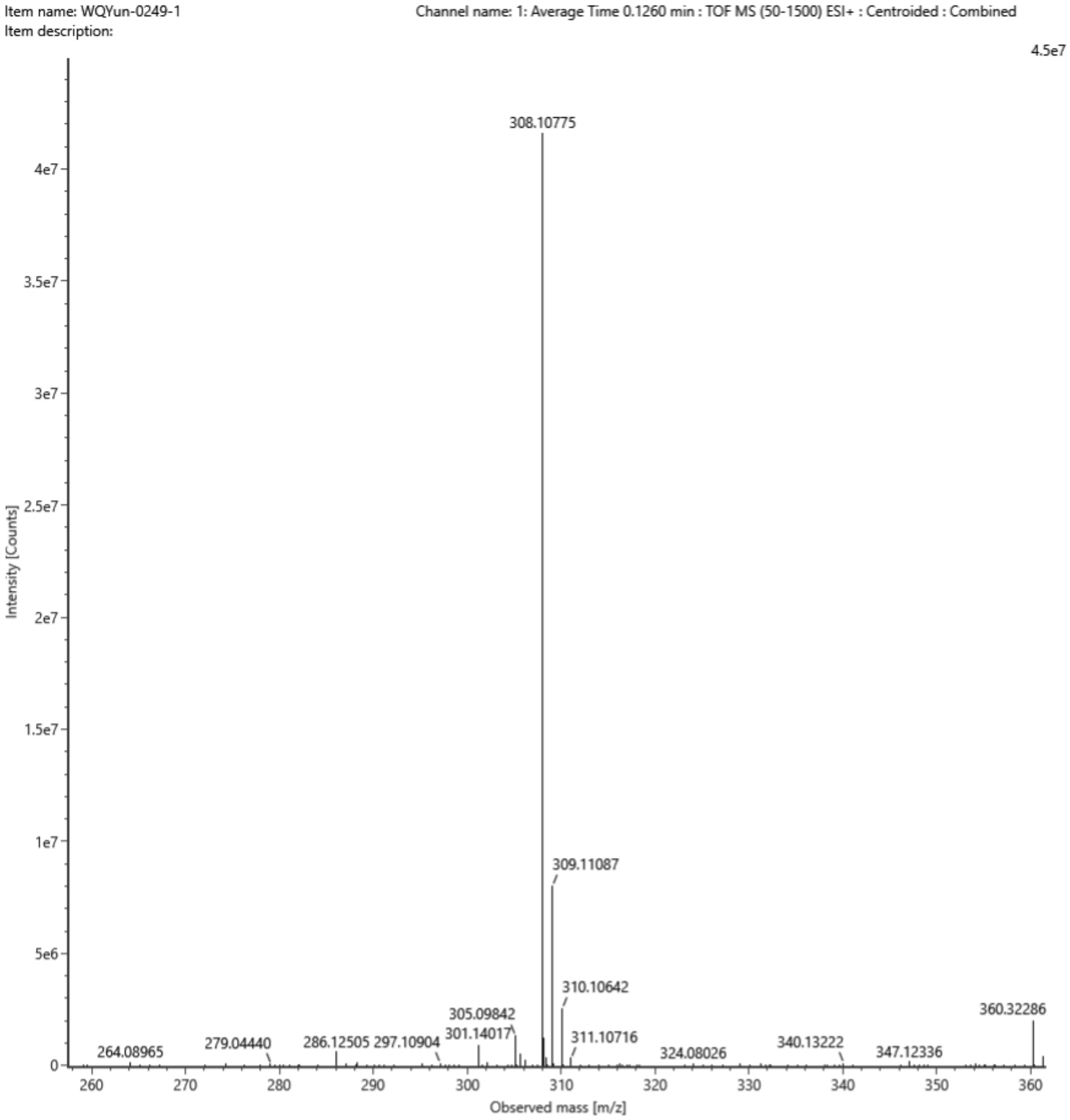

Add:Na$^+$

Composition	i-FIT Confidence (%)	Predicted m/z	m/z error (PPM)
C17H19NOS	100.000000	308.107956	-0.722667

Supplementary Figure 195. HRMS spectra of 3w

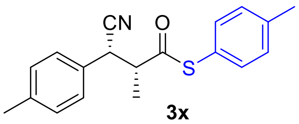

3x

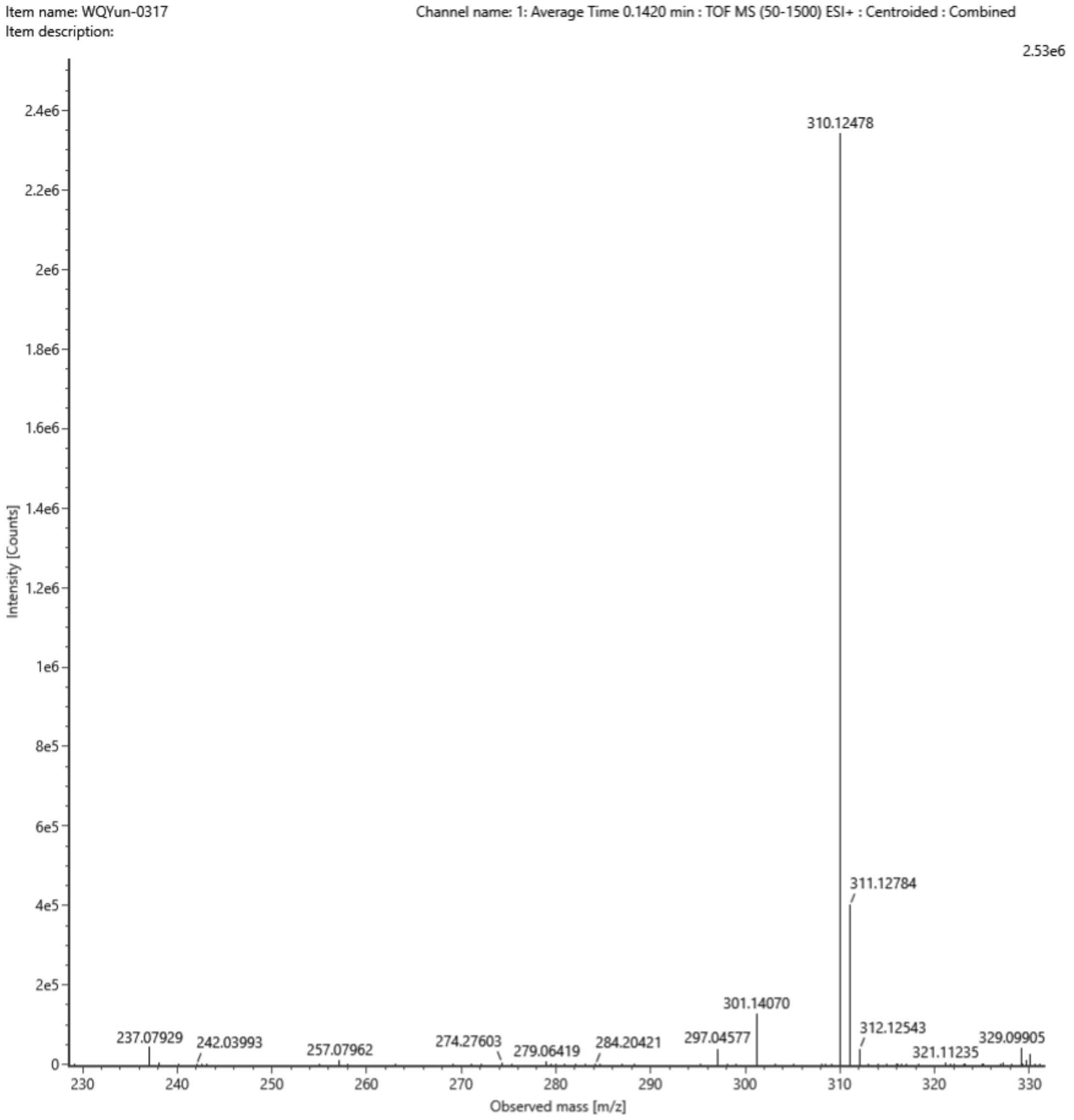

Add:H+

Composition	i-FIT Confidence (%)	Predicted m/z	m/z error (PPM)
C19H19NOS	100.000000	309.118731	1.952017

Supplementary Figure 196. HRMS spectra of 3x

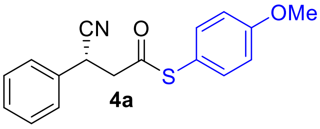

4a

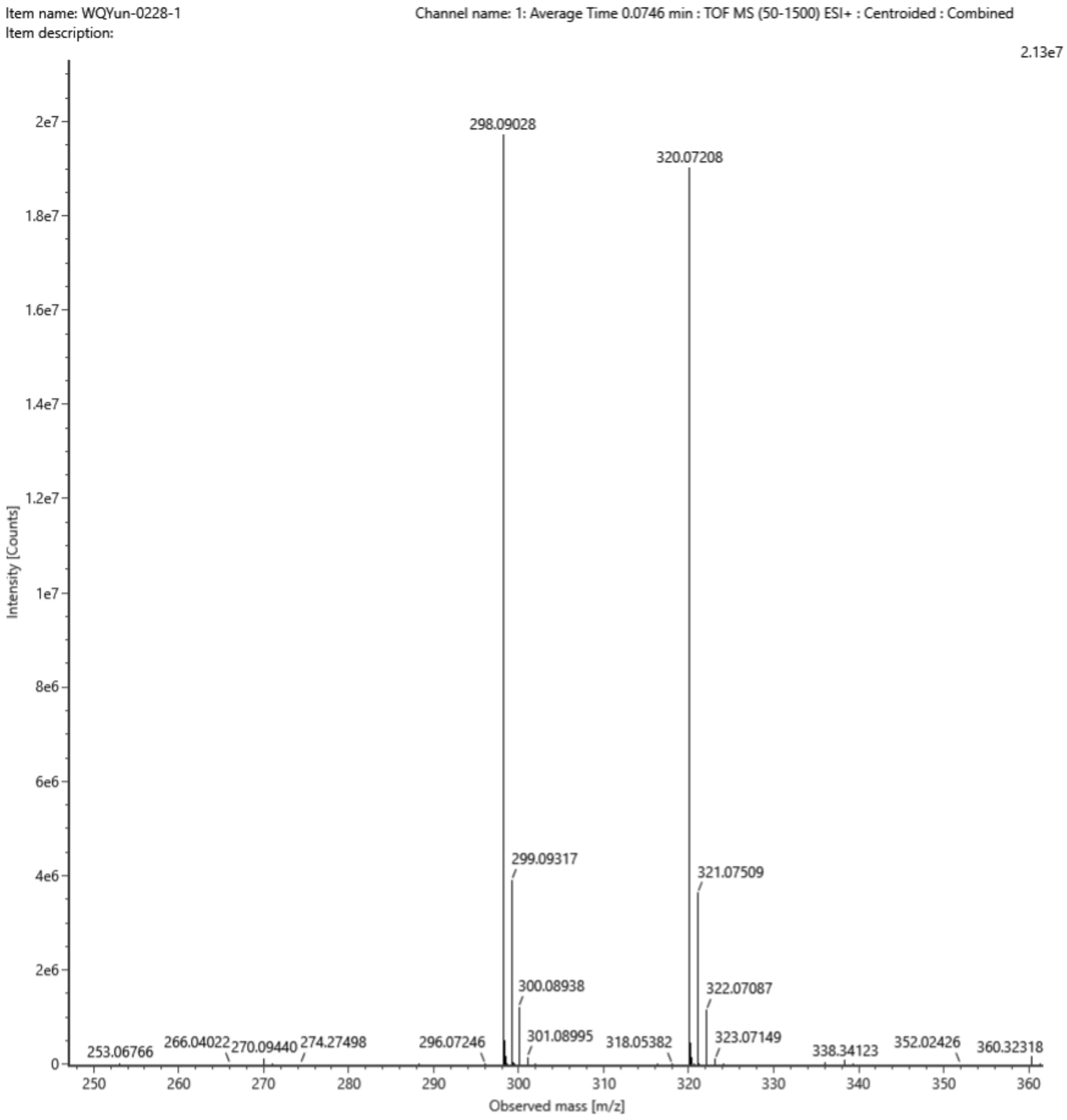

Add:H^+

Composition	i-FIT Confidence (%)	Predicted m/z	m/z error (PPM)
C17H15NO2S	100.000000	298.089626	2.201700

Supplementary Figure 197. HRMS spectra of 4a

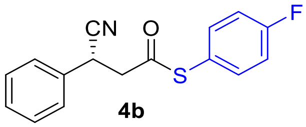

4b

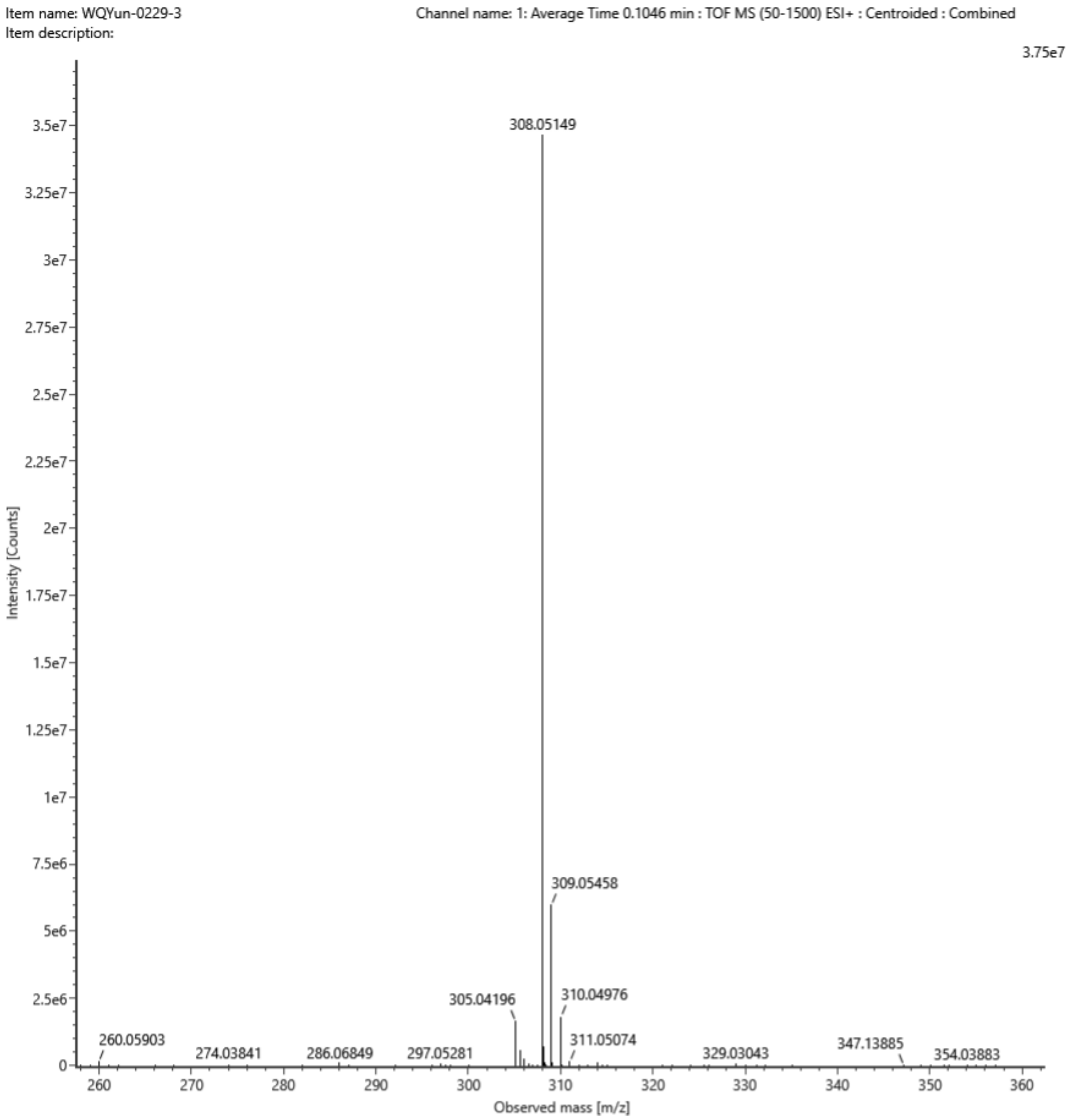

Add:Na^{+}

Composition	i-FIT Confidence (%)	Predicted m/z	m/z error (PPM)
C16H12FNOS	100.000000	308.051584	-0.306218

Supplementary Figure 198. HRMS spectra of 4b

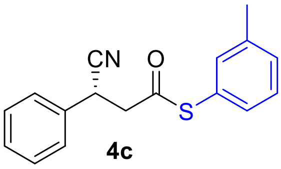

4c

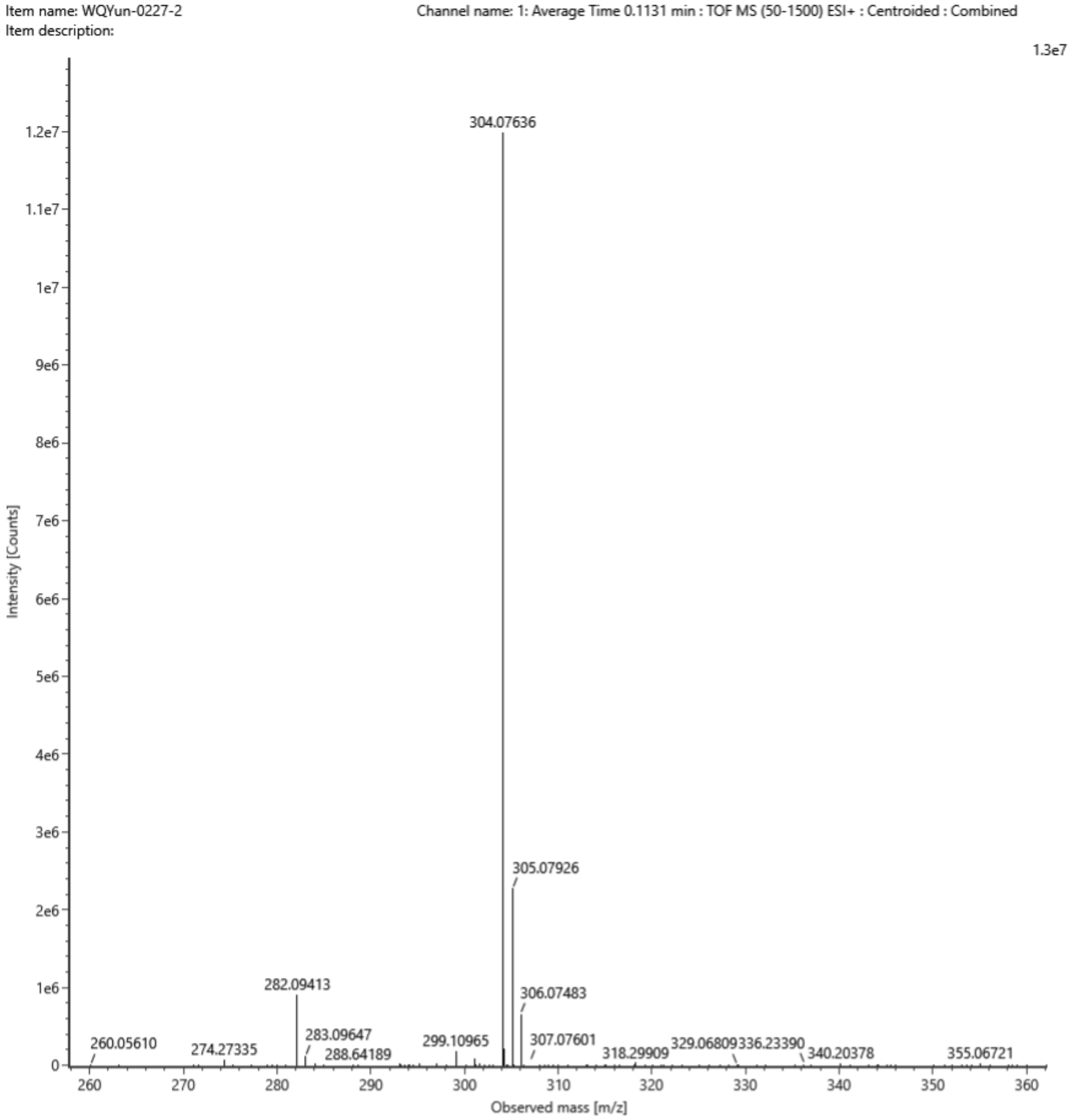

Add:Na^+

Composition	i-FIT Confidence (%)	Predicted m/z	m/z error (PPM)
C17H15NOS	100.000000	304.076656	-0.976405

Supplementary Figure 199. HRMS spectra of 4c

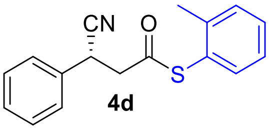

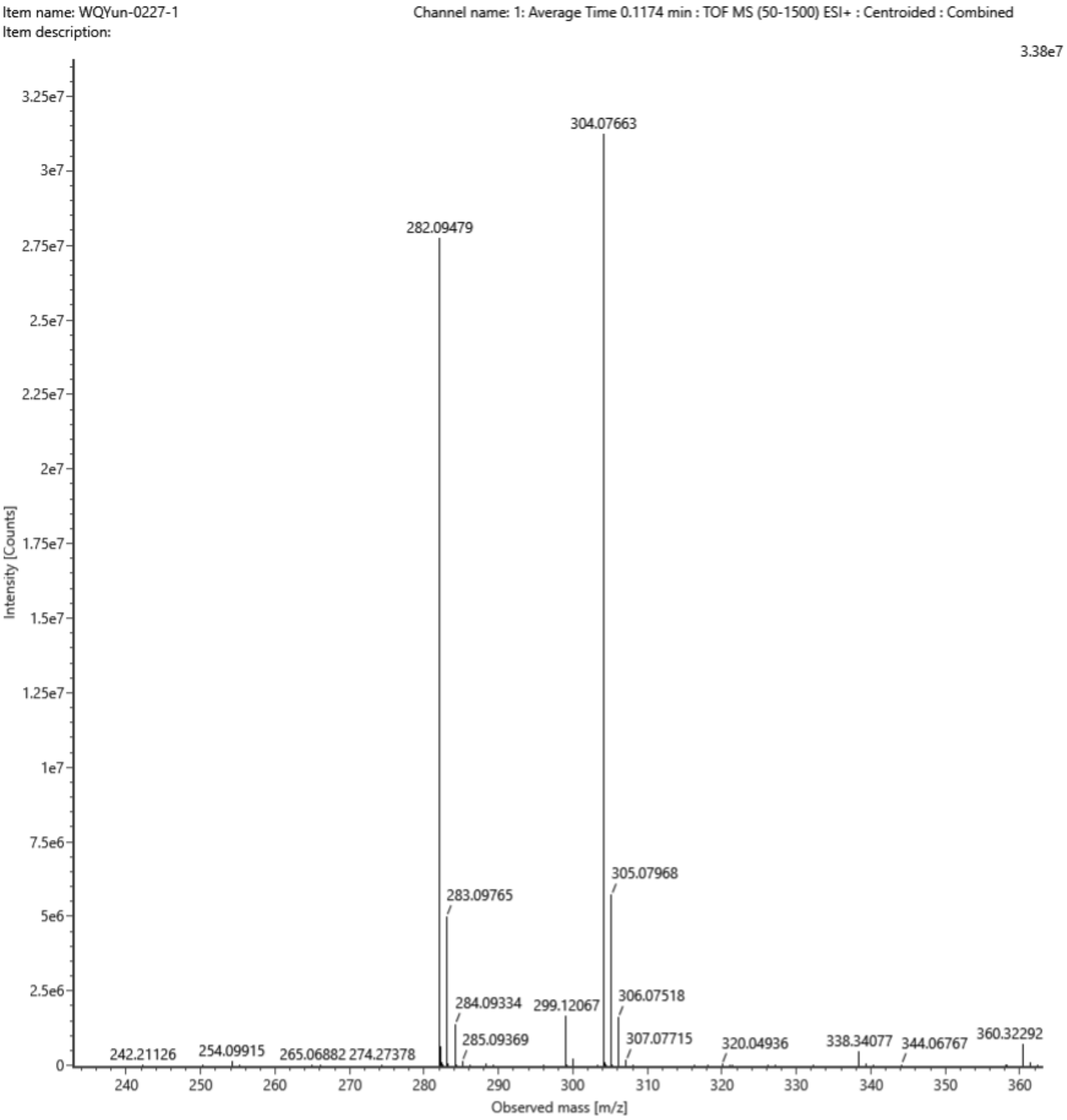

Add:Na^+

Composition	i-FIT Confidence (%)	Predicted m/z	m/z error (PPM)
C17H15NOS	100.000000	304.076656	-0.085519

Supplementary Figure 200. HRMS spectra of 4d

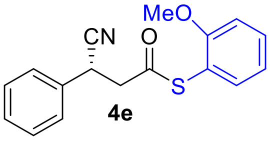

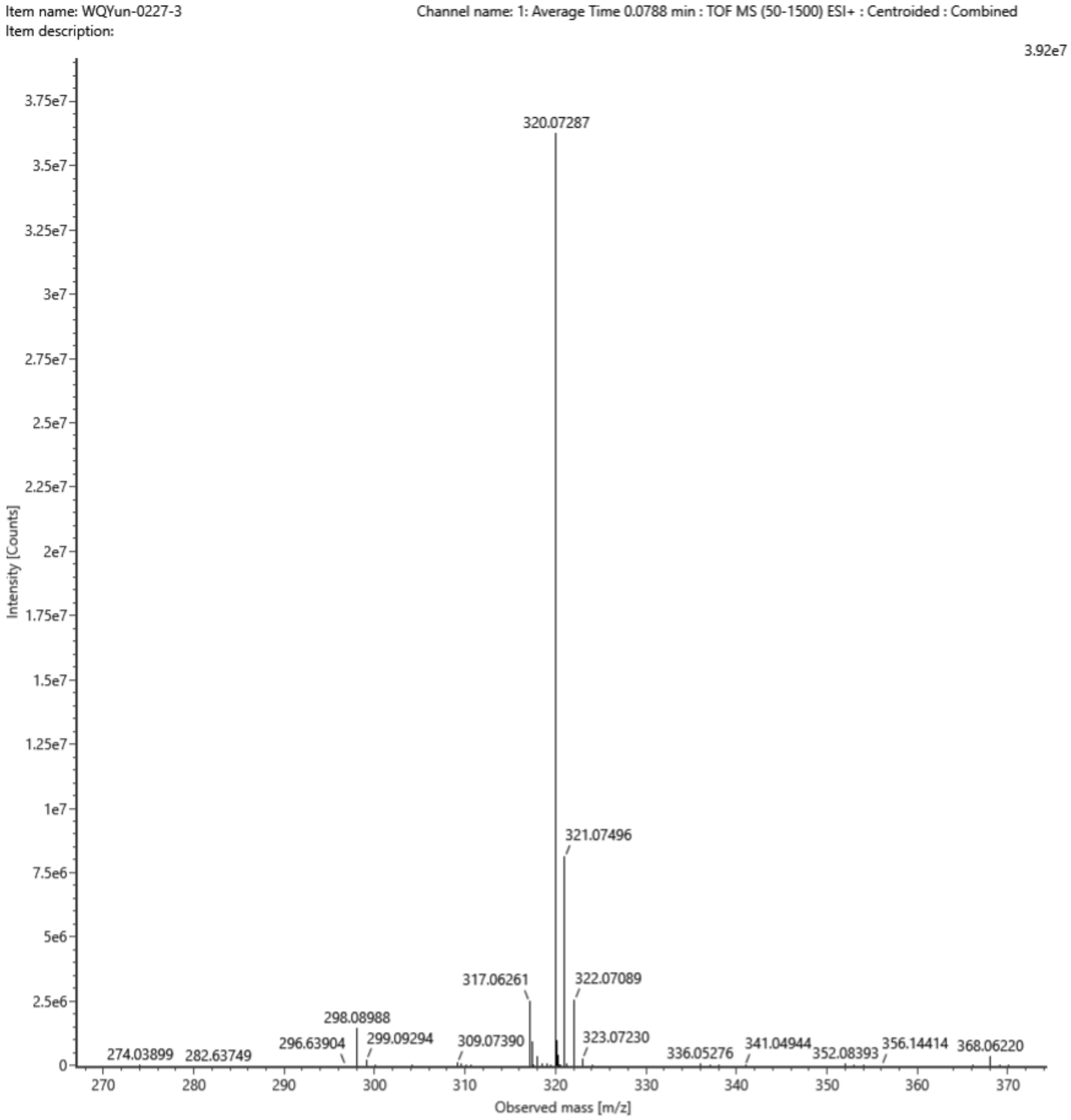

Add:Na$^+$

Composition	i-FIT Confidence (%)	Predicted m/z	m/z error (PPM)
C17H15NO2S	100.000000	320.071571	4.072703

Supplementary Figure 201. HRMS spectra of 4e

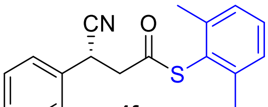

4f

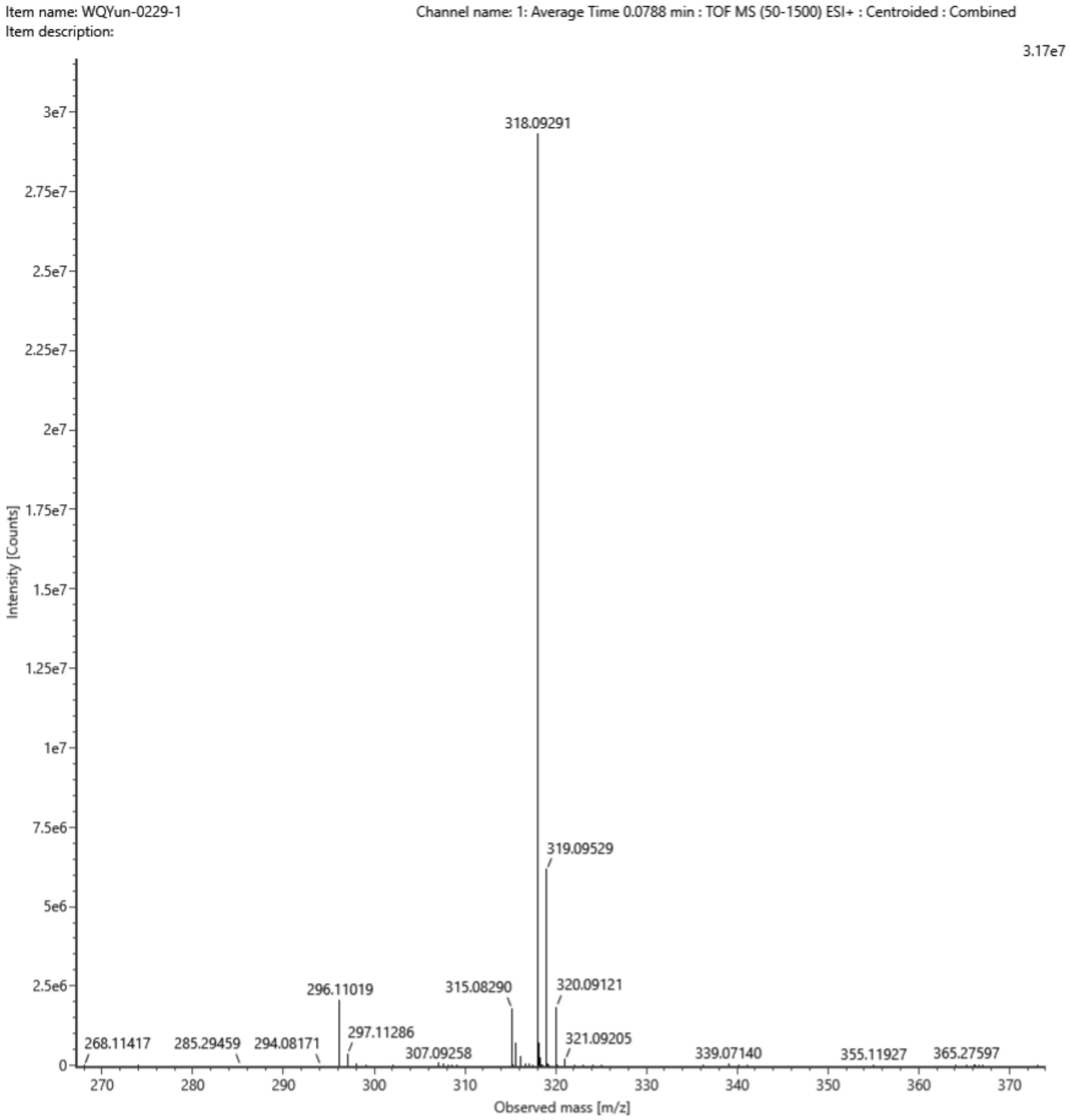

Add:Na^{+}

Composition	i-FIT Confidence (%)	Predicted m/z	m/z error (PPM)
C18H17NOS	100.000000	318.092306	1.904905

Supplementary Figure 202. HRMS spectra of 4f

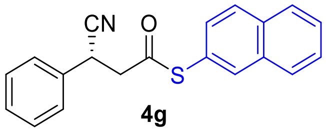

4g

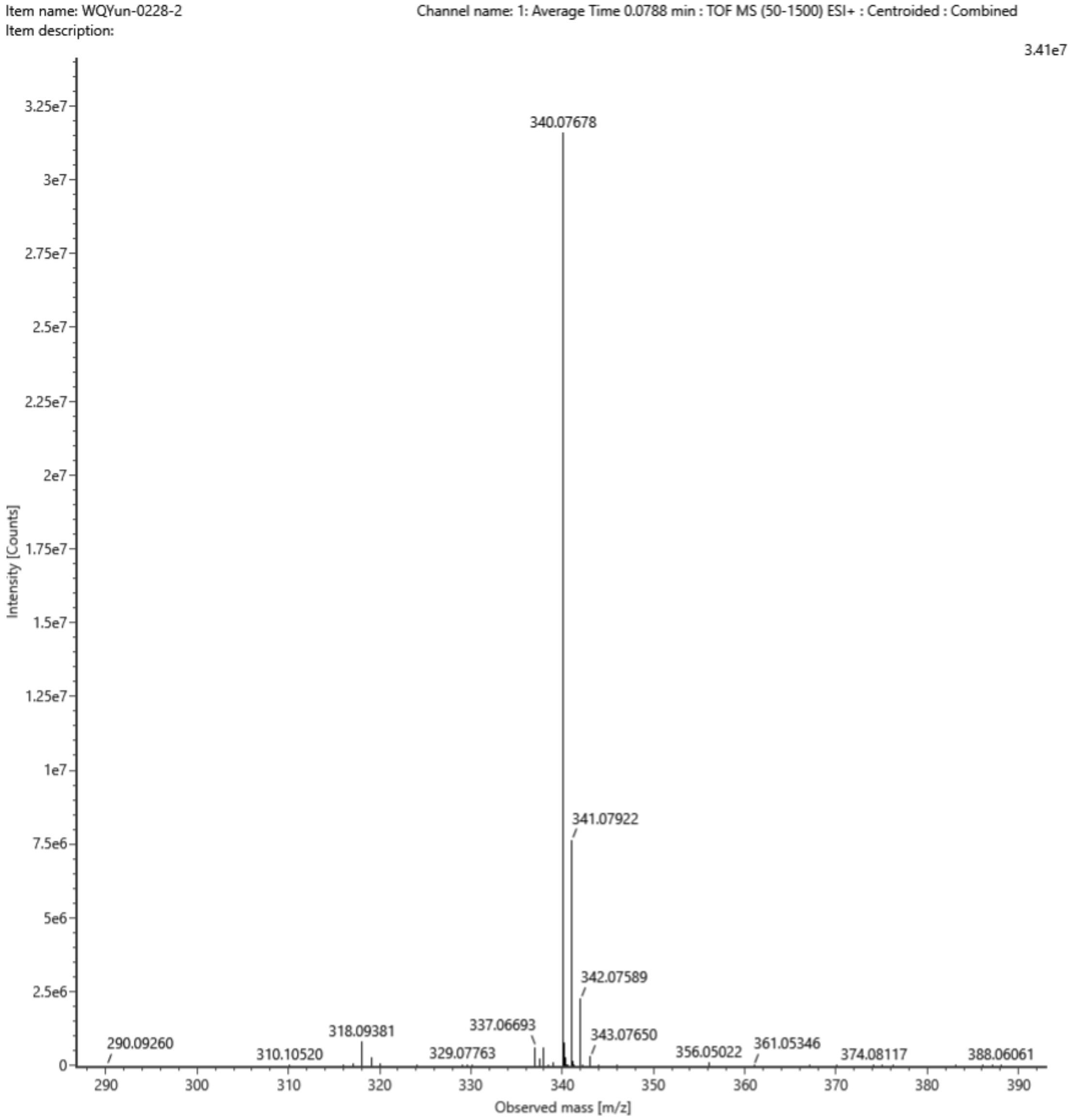

Add:Na^+

Composition	i-FIT Confidence (%)	Predicted m/z	m/z error (PPM)
C20H15NOS	100.000000	340.076656	0.365948

Supplementary Figure 203. HRMS spectra of 4g

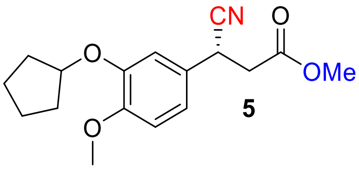

5

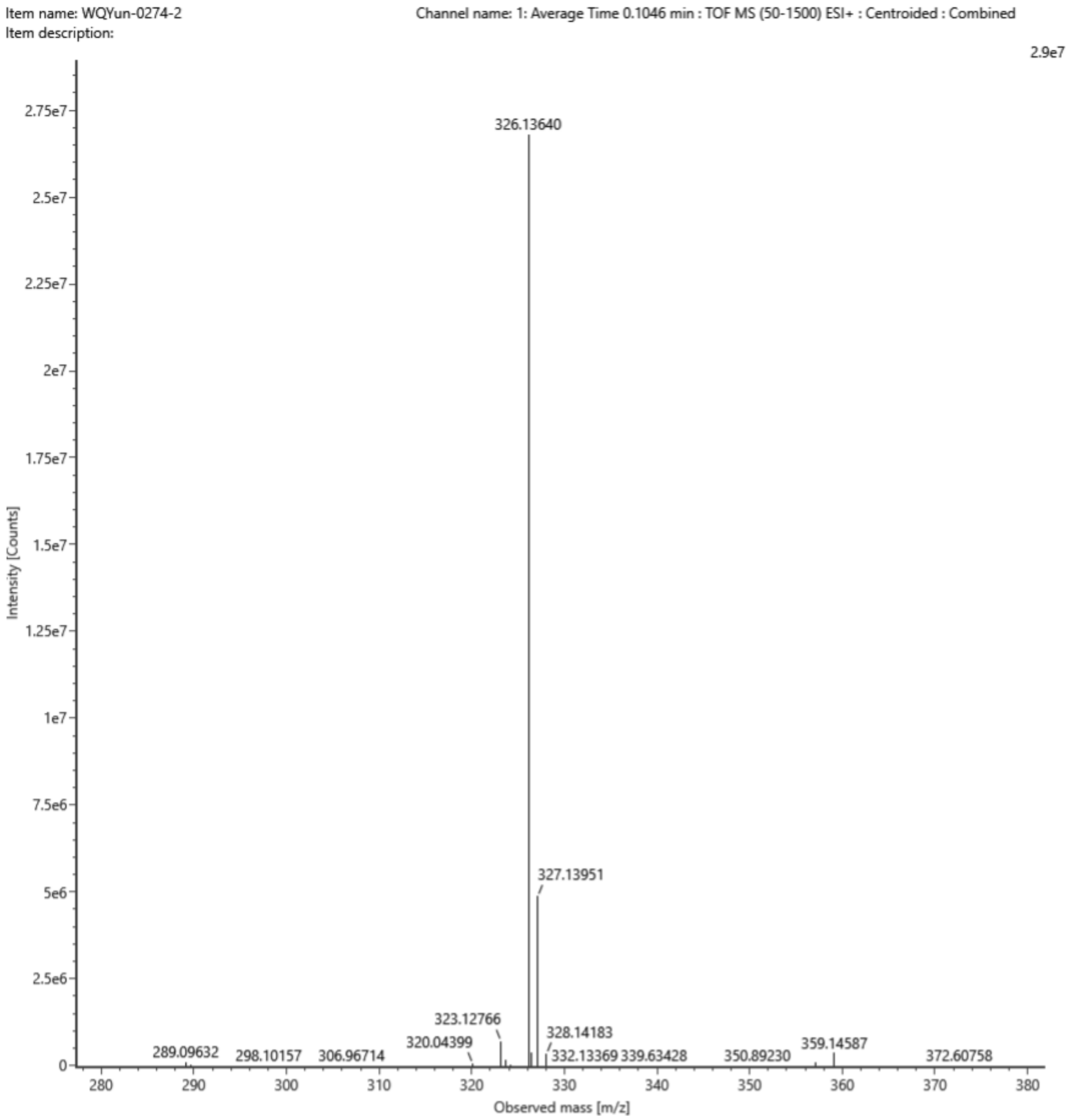

Add:Na^+

Composition	i-FIT Confidence (%)	Predicted m/z	m/z error (PPM)
C17H21NO4	100.000000	326.136279	0.371401

Supplementary Figure 204. HRMS spectra of 5

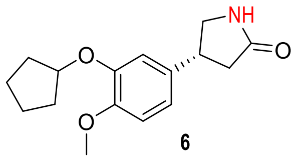

6

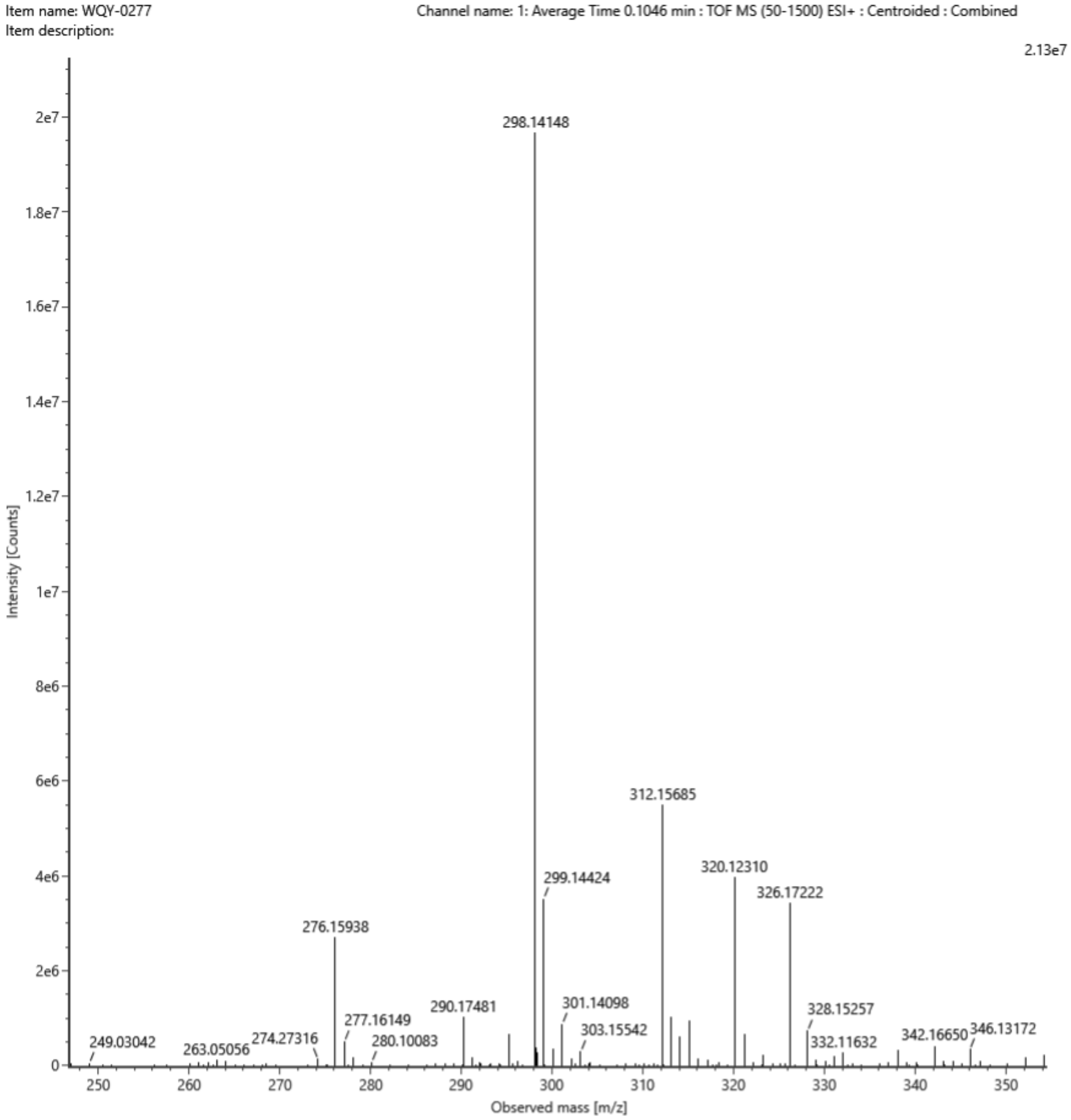

Add:Na^+

Composition	i-FIT Confidence (%)	Predicted m/z	m/z error (PPM)
C16H21NO3	100.000000	298.141365	0.388295

Supplementary Figure 205. HRMS spectra of 6

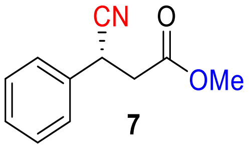

7

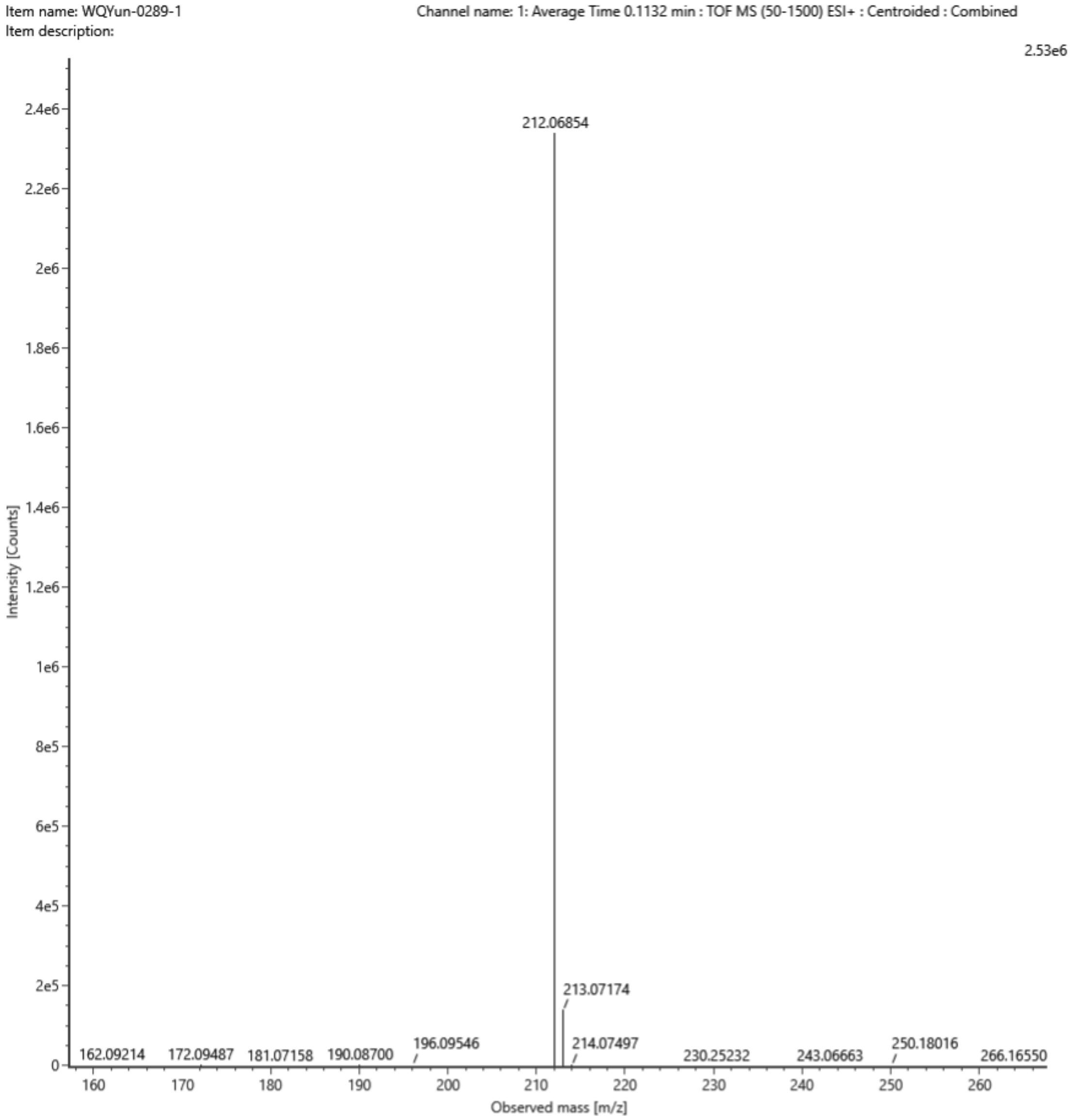

Add:Na^+

Composition	i-FIT Confidence (%)	Predicted m/z	m/z error (PPM)
C11H11NO2	100.000000	212.068200	1.612409

Supplementary Figure 206. HRMS spectra of 7

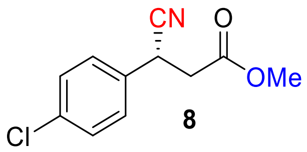

8

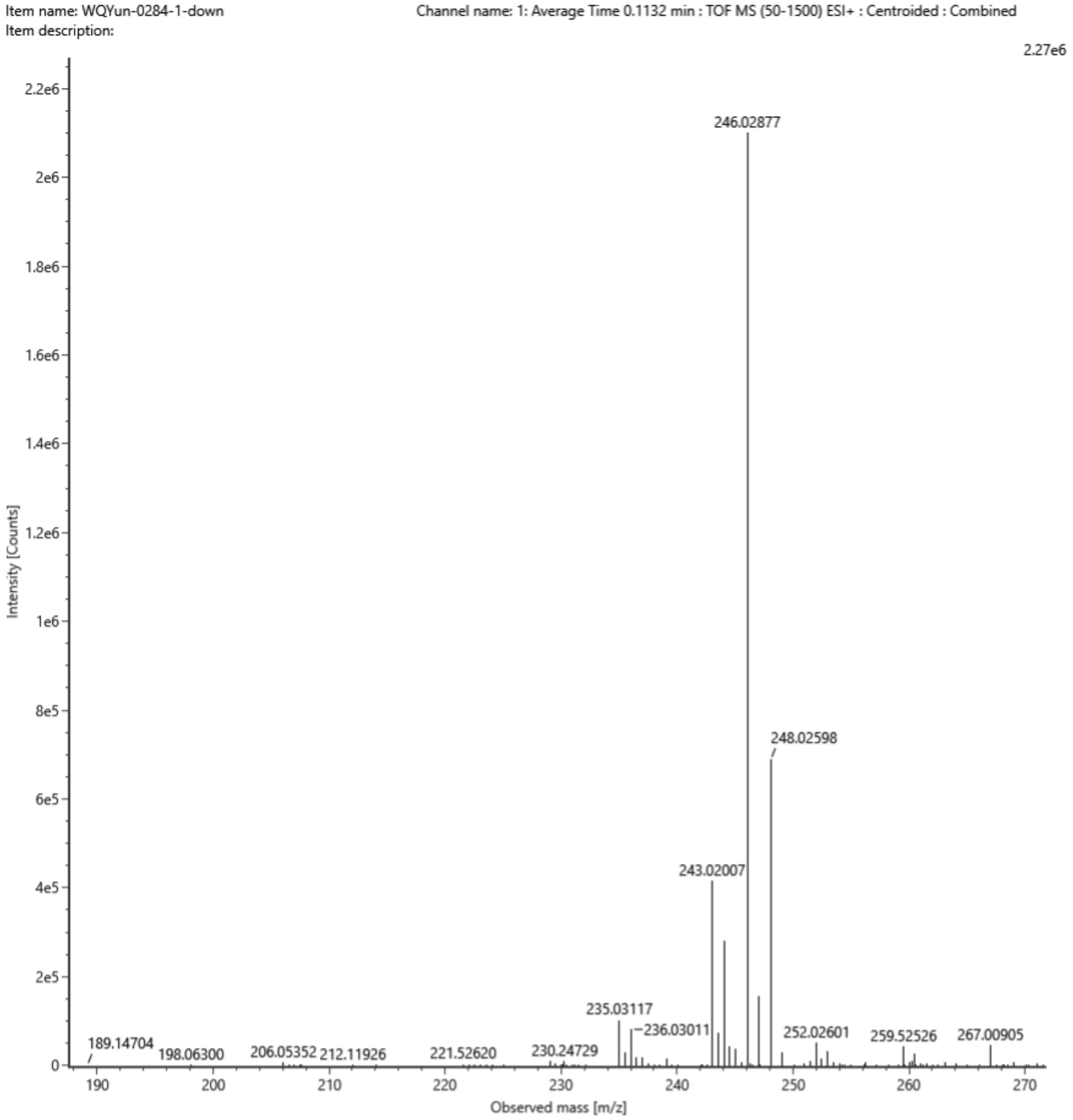

Add:Na^+

Composition	i-FIT Confidence (%)	Predicted m/z	m/z error (PPM)
C11H10ClNO2	99.999994	246.029227	-1.866616

Supplementary Figure 207. HRMS spectra of 8

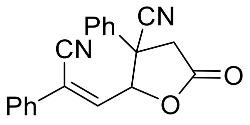

3a'

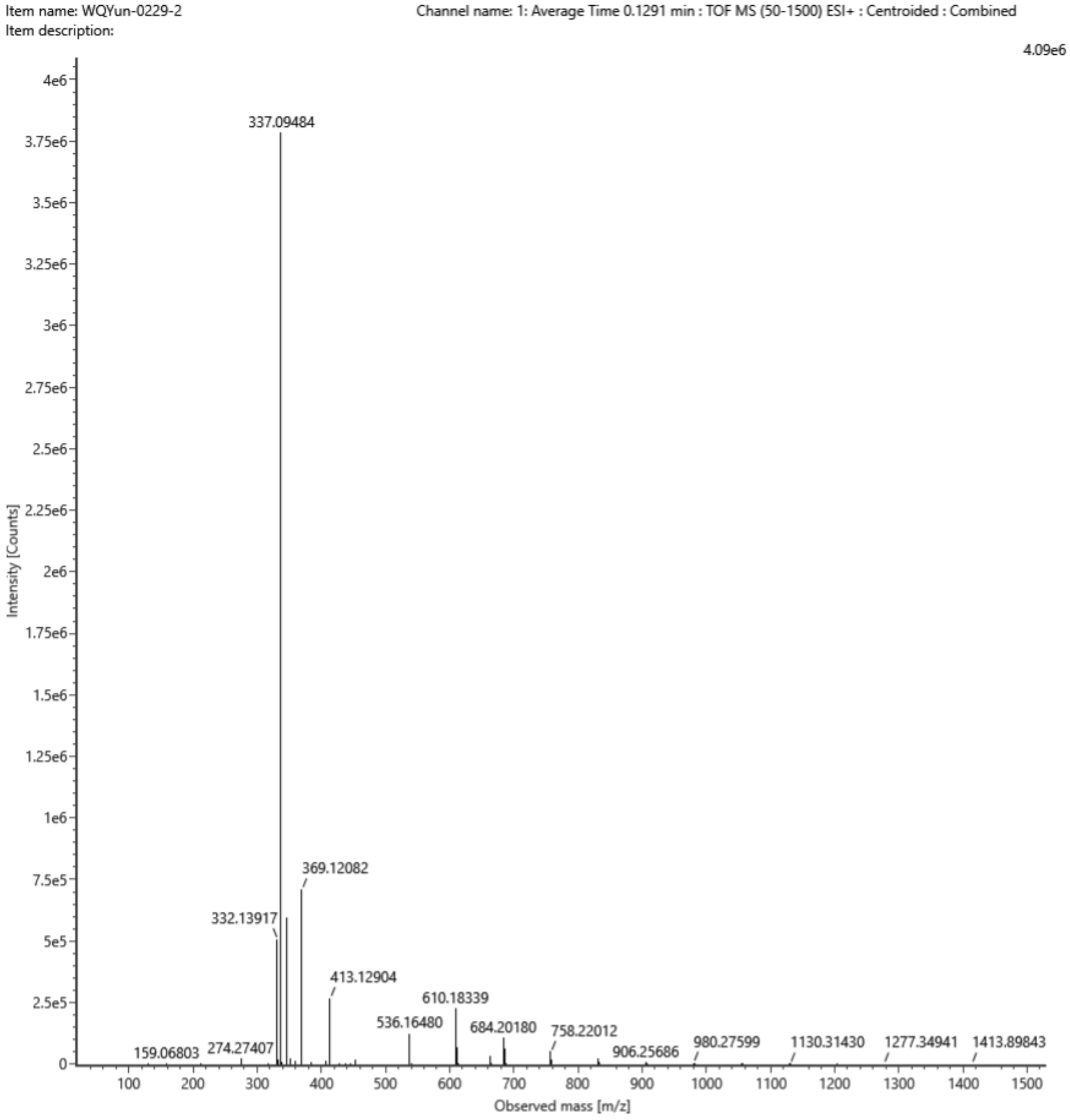

Add:Na^+

Composition	i-FIT Confidence (%)	Predicted m/z	m/z error (PPM)
C20H14N2O2	100.000000	337.094749	0.271399

Supplementary Figure 208. HRMS spectra of 3a'

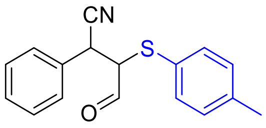

I

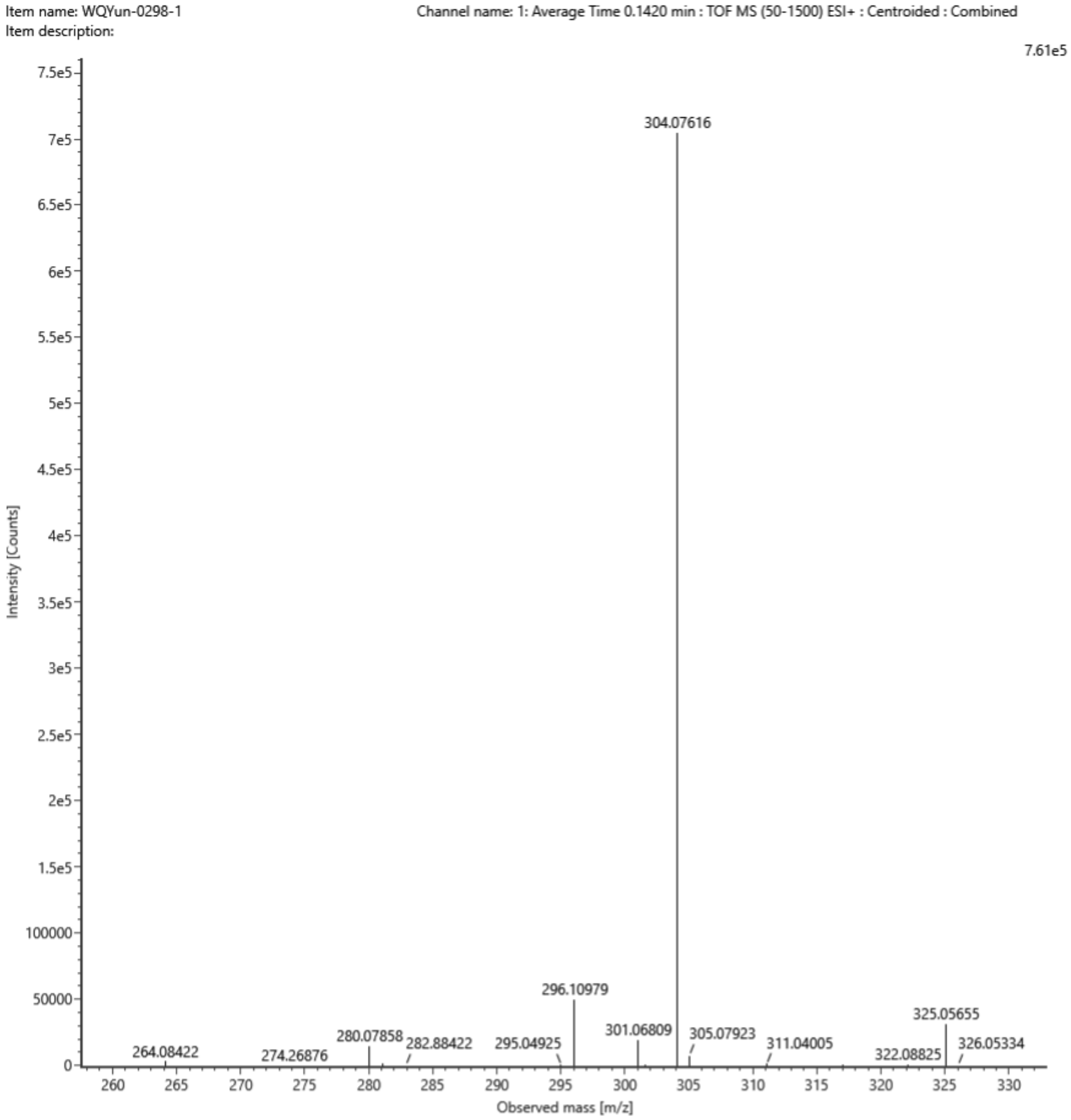

Add:Na^+

Composition	i-FIT Confidence (%)	Predicted m/z	m/z error (PPM)
C17H15NOS	100.000000	304.076656	-1.636322

Supplementary Figure 209. HRMS spectra of I

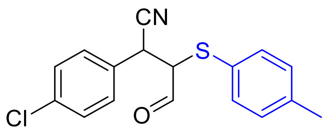

I'

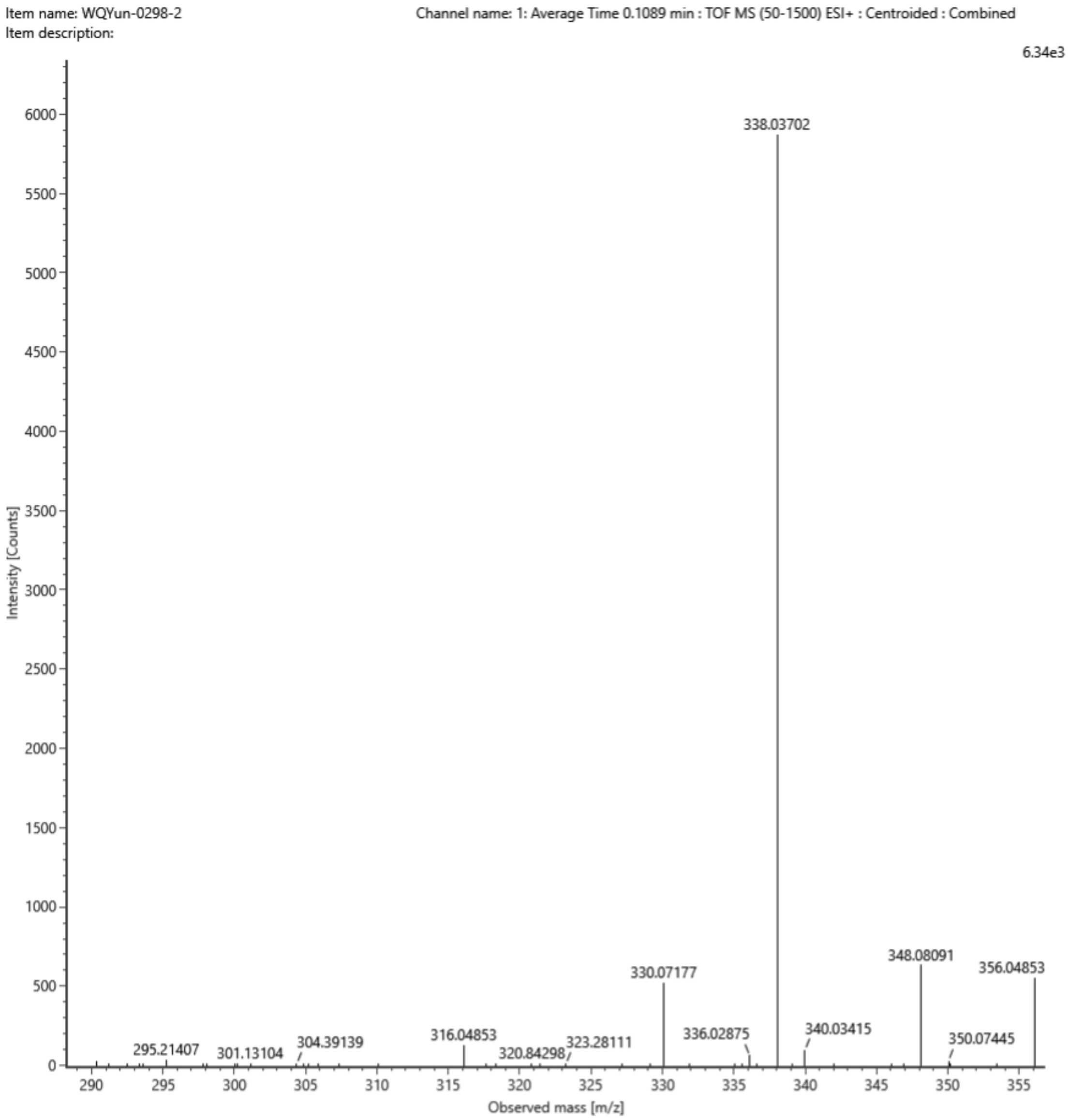

Add:Na+

Composition	i-FIT Confidence (%)	Predicted m/z	m/z error (PPM)
C17H14ClNOS	100.000000	338.037684	-1.968955

Supplementary Figure 210. HRMS spectra of I’

X-Ray crystallography

Colorless block crystal of **1k** was obtained by vaporization of a petroleum ether / ethyl acetate solution. The colorless block crystals of compounds **3a**, **3x** and **I'** were obtained by vaporization of a petroleum ether / dichloromethane solution / ethanol solution. The absolute stereochemistry was determined by the X-ray diffraction. These crystals were deposited in the Cambridge Crystallographic Data Centre and assigned.
www.ccdc.cam.ac.uk/data request/cif.

Supplementary Table 3. X-ray crystallographic analysis of 1k.

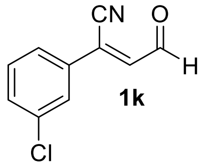 **1k**	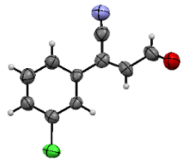 CCDC: 2220606

Bond precision: C-C = 0.0129 A Wavelength=0.71073

Cell:	a=11.5027(11)	b=29.695(3)	c=7.6829(7)
	alpha=90	beta=92.342(3)	gamma=90
Temperature:	298 K		

	Calculated	Reported
Volume	2622.1(4)	2622.1(4)
Space group	C c	C 1 c 1
Hall group	C -2yc	C -2yc
Moiety formula	C10 H6 Cl N O	C10 H6 Cl N O
Sum formula	C10 H6 Cl N O	C10 H6 Cl N O
Mr	191.61	191.61
Dx,g cm-3	1.456	1.456
Z	12	12
Mu (mm-1)	0.388	0.388
F000	1176.0	1176.0
F000'	1178.21	
h,k,lmax	13,35,9	13,35,9
Nref	4607[2312]	3871
Tmin,Tmax	0.920,0.955	0.845,0.955
Tmin'	0.840	

Correction method= # Reported T Limits: Tmin=0.845 Tmax=0.955
AbsCorr = MULTI-SCAN

Data completeness= 1.67/0.84 Theta(max)= 25.017

R(reflections)= 0.0601(2633) wR2(reflections)= 0.1513(3871)

S = 1.076 Npar=352

Supplementary Table 4. X-ray crystallographic analysis of 3a.

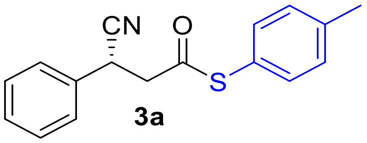

3a

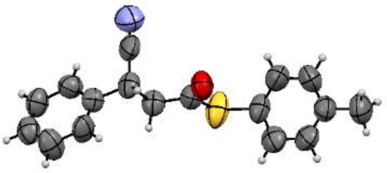

CCDC: 2220572

Bond precision: C-C = 0.0138 A   Wavelength=0.71073

Cell:	a=7.6254(7)	b=12.5274(14)	c=32.086(3)
	alpha=90	beta=90	gamma=90
Temperature:	298 K		

	Calculated		Reported
Volume	3065.1(5)		3065.1(5)
Space group	P 21 21 21		P 21 21 21
Hall group	P 2ac 2ab	O S	P 2ac 2ab
Moiety formula	C17 H15 N		C17 H15 N O S
Sum formula	C17 H15 N	O S	C17 H15 N O S
Mr	281.36		281.36
Dx,g cm-3	1.219		1.219
Z	8		8
Mu (mm-1)	0.206		0.206
F000	1184.0		1184.0
F000’	1185.42		
h,k,lmax	9,14,38		9,14,38
Nref	5404[3098]		5394
Tmin,Tmax	0.961,0.980		0.917,0.980
Tmin’	0.915		

Correction method= # Reported T Limits: Tmin=0.917
Tmax=0.980 AbsCorr = MULTI-SCAN

Data completeness= 1.74/1.00   Theta(max)= 25.018

R(reflections)= 0.0567(1735)   wR2(reflections)= 0.1318(5394)

S = 0.821   Npar=363

Supplementary Table 5. X-ray crystallographic analysis of 3x.

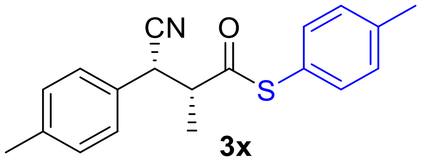

3x

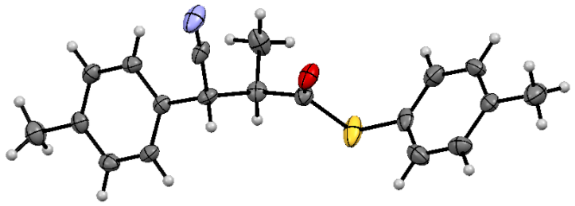

CCDC: 2277728

Bond precision: C-C = 0.0035 A   Wavelength=1.54184

Cell:	a=9.0019(8)	b=9.7374(5)	c=19.3454(11)
	alpha=90	beta=90	gamma=90
Temperature:	116 K		

	Calculated		Reported
Volume	1695.7(2)		1695.7(2)
Space group	P 21 21 21		P 21 21 21
Hall group	P 2ac 2ab	O S	P 2ac 2ab
Moiety formula	C19 H19 N		C19 H19 N O S
Sum formula	C19 H19 N	O S	C19 H19 N O S
Mr	309.41		309.41
Dx,g cm-3	1.212		1.212
Z	4		4
Mu (mm-1)	1.691		1.691
F000	656.0		656.0
F000’	658.95		
h,k,lmax	10,11,22		10,11,22
Nref	2979[1728]		2904
Tmin,Tmax	0.601,0.689		0.847,1.000
Tmin’	0.545		

Correction method= # Reported T Limits: Tmin=0.847 Tmax=1.000

AbsCorr = MULTI-SCAN

Data completeness= 1.68/0.97   Theta(max)= 66.210

R(reflections)= 0.0418(2691)   wR2(reflections)= 0.1053(2904)

S = 1.023   Npar=202

Supplementary Table 7. X-ray crystallographic analysis of I’.

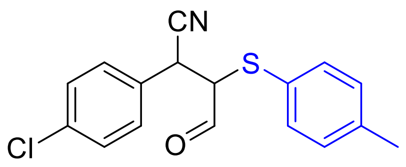

I'

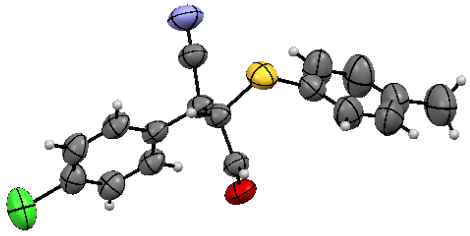

CCDC：2221058

Bond precision: C-C = 0.0038 A   Wavelength=1.54178

Cell:	a=14.3651(7)	b=5.5757(3)	c=20.1055(10)
	alpha=90	beta=96.250(2)	gamma=90
Temperature:	273 K		

	Calculated	Reported
Volume	1600.79(14)	1600.79(14)
Space group	P 2/c	P 1 2/c 1
Hall group	-P 2yc	-P 2yc
Moiety formula	C17 H14 Cl N O S	C17 H14 Cl N O S
Sum formula	C17 H14 Cl N O S	C16 H0.25 Cl N2 O
S Mr	315.80	303.94
Dx,g cm-3	1.310	1.261
Z	4	4
Mu (mm-1)	3.304	3.323
F000	656.0	605.0
F000’	660.29	
h,k,lmax	17,6,24	17,6,24
Nref	3056	3018
Tmin,Tmax		
Tmin’		

Correction method= Not given

Data completeness= 0.988   Theta(max)= 70.182

R(reflections)= 0.0536(2682)   wR2(reflections)= 0.1607(3018)

S = 1.139   Npar=191

VI. Supplementary References

(1) Wu, Q. et al. NHC-catalyzed enantioselective synthesis of dihydropyran-4-carbonitriles bearing all-carbon quaternary centers. *Org. Chem. Front.* **4**, 2323-2326 (2017).

(2) Soli, E. D. et al. Azide and cyanide displacements via hypervalent silicate intermediates. *J. Org. Chem.* **64**, 3171-3177 (1999).

(3) Zhang, X. et al. An enantioconvergent halogenophilic nucleophilic substitution (S_N2X) reaction. *Science* **363**, 400-404 (2019).

(4) Li, X. et al. Rhodium-catalyzed asymmetric hydrogenation of β-cyanocinnamic esters with the assistance of a single hydrogen bond in a precise position. *Chem. Sci.* **9**, 1919-1924 (2018).

(5) Langlois, N., Dahuron, N. & Wang, H. S. Enantioselective syntheses of (R)-3-phenyl gaba, (R)-baclofen and 4-arylpyrrolidin-2-ones. *Tetrahedron* **52**, 15117-15126 (1996).

(6) Brenna, E. et al. Opposite enantioselectivity in the bioreduction of (Z)-β-aryl-β-cyanoacrylates mediated by the tryptophan 116 mutants of old yellow enzyme 1: Synthetic approach to (R)- and (S)-β-aryl-γ-lactams. *Adv. Synth. Catal.* **357**, 1849-1860 (2015).
